# Widespread associations between behavioral metrics and brain microstructure in ASD suggest age mediates subtypes of ASD

**DOI:** 10.1162/IMAG.a.144

**Published:** 2025-09-10

**Authors:** Haylee J. Ressa, Benjamin T. Newman, Zachary Jacokes, James C. McPartland, Natalia M. Kleinhans, T. Jason Druzgal, Kevin A. Pelphrey, John Darrell Van Horn

**Affiliations:** Department of Psychology, University of Virginia, Gilmer Hall, Charlottesville, VA, United States; Department of Radiology and Medical Imaging, University of Virginia, School of Medicine, Gilmer Hall, Charlottesville, VA, United States; School of Data Science, University of Virginia, Elson Building, Charlottesville, VA, United States; Yale Child Study Center and the Yale Center for Brain and Mind Health, Yale School of Medicine, Sterling Hall of Medicine, New Haven, CT, United States; University of Washington Integrated Brain Imaging Center, Eunice Kennedy Shriver Intellectual and Developmental Disabilities Research Center, Seattle, WA, United States

**Keywords:** autism spectrum disorder, neurodevelopment, diffusion-weighted imaging, white matter microstructure, behavior, age

## Abstract

Autism spectrum disorder (ASD) is a neurodevelopmental disorder characterized by differences in social communication and repetitive behaviors. Our laboratory has previously found that g-ratio, the proportion of axon width to myelin diameter, and axonal conduction velocity, which is associated with the capacity of an axon to carry information, are both decreased throughout the adolescent brain in ASD. By associating these differences with performance on cognitive and behavioral tests, this study aims to first associate a broad array of behavioral metrics with neuroimaging markers of ASD, and to explore the prevalence of ASD subtypes using a neuroimaging driven perspective. Analyzing 273 participants (148 with ASD) ages 8 to 17 years through an NIH-sponsored Autism Centers of Excellence network (MH100028), we observe widespread associations between behavioral and cognitive evaluations of autism and between behavioral and microstructural metrics, alongside different directional correlations between different behavioral metrics. Stronger associations with individual subcategories from each test rather than summary scores suggest that different neuronal profiles may be masked by composite test scores. Machine learning cluster analyses applied to neuroimaging data reinforce the association between neuroimaging and behavioral metrics and suggest that age-related maturation of brain metrics may drive changes in ASD behavior. This suggests that if ASD can be definitively subtyped, these subtypes may show different behavioral trajectories across the developmental period. Clustering identified a pattern of restrictive and repetitive behavior in some participants and a second group that was defined by high sensory sensitivity and language performance.

## 1 Introduction

Autism spectrum disorder (ASD) is a neurodevelopmental disorder with a prevalence of approximately 1 in 36 children (Maenner et al., 2023). In a clinical setting, a diagnosis of ASD is made by a clinician according to criteria established by the Diagnostic and Statistical Manual of Mental Disorders (DSM-5) that describe persistent social or communication differences, and restricted or repetitive behaviors or interests, which can include atypical response to sensory information (*Diagnostic and Statistical Manual of Mental Disorders*, 2013). Recently, to counteract variability in clinical decision making, there has been an increased emphasis on assessing performance on one or more behavioral and cognitive tests (Constantino & Charman, 2016). Several tests have been developed and normed, but the relationship between different test metrics and the neural structure underlying ASD is still unclear. The highly heterogeneous nature of behaviors in ASD suggests that individuals exist on a spectrum from neurotypical to those with high support needs due to severe autism. Concurrently research may suggest that there is not a singular form of autism but rather multiple forms of “autisms” (Masi et al., 2017). Large datasets have played a critical role in advancing autism research by offering a comprehensive means to study variability across diverse aspects, including genetic factors, brain structure, and behavioral traits (Lombardo et al., 2019).

### 1.1 ASD and the brain

Research highlighting altered functional connectivity in individuals with ASD has given rise to the underconnectivity theory, suggesting that individuals with ASD experience reduced functional connectivity in both frontal and posterior brain regions, attributed to limited cortical bandwidth (Just et al., 2012). However recent studies have challenged this theory by demonstrating that patterns of atypical neural connectivity in autism cannot be consistently explained by distance or connectivity (Picci et al., 2016). Furthermore, idiosyncratic connectivity patterns in which individuals with ASD show greater inter-individual variability have been observed (Hong et al., 2017). Underlying cellular differences, such as changes in neuronal microstructure, may contribute to these connectivity variations. Diffusion MRI provides a means to analyze axonal characteristics such as diameter and anisotropy at a sub-voxel level (Afzali et al., 2021). Deficiencies in axonal structure can impair the speed or efficiency of action potentials. The thickness of the myelin sheath surrounding white matter in the cortex is associated with the inner diameter of axons, which influences conduction velocity (Liewald et al., 2014). Additionally, effective long-range signaling depends on axons with larger diameters to support signal transmission across greater distances (Das & Gilbert, 1995).

### 1.2 Diffusion microstructure and ASD

Structural differences in the axons of ASD participants have been observed in previous diffusion studies. In particular, ASD individuals exhibited altered white matter microstructural patterns in the splenium of the corpus callosum (Zhao et al., 2022). Findings suggest ASD participants have lower fractional anisotropy and higher diffusivity in white matter tracts associated with behaviors commonly disrupted in ASD individuals. Fixel-based analysis of white matter tracts demonstrated that ASD individuals have lower fiber density in the splenium, corresponding with greater social impairments (Dimond et al., 2019). Alternatively, neurite orientation dispersion and density imagining (NODDI) has found that ASD participants had higher extracellular free-water levels and lower neurite density. These differences were mainly present in long-range association tracts that guide ASD behaviors (Andica et al., 2021).

### 1.3 Conduction velocity, g-ratio, and extracellular water

A recent paper (Newman et al., 2024) demonstrated architectural differences in the axonal microstructure of ASD participants. g-ratio is a proportion of axon to myelin diameter with an optimal range of around 0.6–0.7 (Rushton, 1951). As axon and myelination thickness play a role in signal transduction speed and efficiency, g-ratio is related to conduction velocity, a measure associated with an axon’s capacity to carry information sensitive to myelin and axonal development differences. This study found that in the same adolescent sample utilized in this study, ASD participants were observed to have increased levels of extracellular water, altered g-ratio, and decreased conduction velocity, compared with non-ASD participants. These microstructural differences were present throughout the cortex, subcortex, and white matter skeleton. Decreases in conduction velocity and g-ratio, in particular, suggest deficits in long-range connections that rely on larger axon and myelination diameters (Newman et al., 2024).

To continue this work, we leveraged a uniquely rich dataset on a cohort of autistic and non-autistic individuals from the NIH-sponsored (MH100028) Autism Centers of Excellence (ACE) cohort. This cohort represents a very large and carefully evaluated group of age, sex, and diagnosis-matched individuals that enabled us to explore the relationship between brain microstructure and behavioral patterns across a spectrum of development. By focusing on microstructural features, we aimed to uncover how variability in brain organization relates to the diverse symptom presentations observed in ASD. One of the central goals of this work is to better understand the neural correlates of ASD subtypes. Rather than attempting to divide autism into rigid subcategories, we adopted a spectrum-based view, recognizing that microstructural features and their behavioral correlates may not align neatly with traditional diagnostic boundaries. Instead, these features may represent dimensions of variability that are shared across typical and atypical development, offering a more nuanced framework for interpreting heterogeneity in autism. Ultimately, this study seeks to address the critical challenge of ASD variability by identifying brain-based markers that could predict symptom patterns and inform future classifications of autism. This approach holds the potential to bridge the gap between brain and behavior, advancing our understanding of ASD heterogeneity and paving the way for more personalized interventions and predictive biomarkers. To achieve this aim, this study takes an exploratory approach to study and characterize ASD in a rich and highly detailed behavioral and neuroimaging dataset.

## 2 Methods

### 2.1 Participants

Two-hundred seventy-three (mean age = 154.3 months ± 35.21 S.D., age range = 96-216 months; 133 female [49%]) participants from Wave 1 of an NIH-sponsored Autism Centers of Excellence Network were included in this study. Written informed consent was obtained from all participants for being included in the study at each recruitment site and the study was approved by the IRB at each participating site (Harvard University, Yale University, Seattle Children’s Hospital with the University of Washington, and the University of California, Los Angeles). During recruitment, participants were excluded from the study cohort if their score on the Differential Ability Scales (DAS) general ability was below 70. Participants were screened and excluded from participation if they had any reported intellectually disabling comorbidities. The study cohort included 148 individuals diagnosed with ASD (mean age = 150.8 months ± 34.31 S.D., 70 female [47%]) and 124 neurotypical participants (mean age = 154.3 months ± 35.21 S.D., 62 female [50%]). Full demographic information is available in Table 1. Data used in this study are available from the NIH National Database for Autism Research (NDAR).

**Table 1.** Demographic information and summary for participants in this study.

	Total	ASD	Non-ASD
Mean age (months)	154.3114 (±35.14514)	150.7703 (±34.19298)	158.9516 (±35.58448)
Percentage female	49%	47%	50%
Mean intracranial volume (mm^3^)	1532466 (±161768.6)	1531830 (±170275.8)	1534959 (±152678.8)
Mean IQ	107.0586 (±18.03812)	106.9926 (±19.2718)	107.0508 (±16.60533)

### 2.2 Behavioral and cognitive assessments

Participants were administered the battery of behavioral and cognitive tests summarized in Table 1 at each participating site (Harvard University, Yale University, Seattle Children’s Hospital with the University of Washington, and the University of California, Los Angeles). All participants within the ASD cohort were validated via the administration of the ADI-R and ADOS-2, including separate social and behavioral subcomponents, by a clinician (Lord et al., 1994). Participants’ family members were also asked about age of language acquisition. The social, behavioral, overall, and composite ADOS-2 scores were included for ASD participants only. Participants not meeting ASD criteria in the ASD cohort were excluded from the study. Participants in the final ASD cohort were paired using age- and sex-matched non-autistic participants. The behavioral tests utilized in this study are as follows:

The Clinical Evaluation of Language Fundamentals—Fourth Edition (CELF-4) is a test given to participants of ages 5 through 21 years to evaluate language ability through expressive and receptive language-based subtests that measure phonology, morphology, syntax, semantics, and working memory. CELF-4 is administered by a professional, typically a speech pathologist. Language skills vary greatly among children with ASD (Salem et al., 2021). While children with ASD tend to have impairments in both expressive and receptive language skills, differences in receptive language are usually more significant (Mody et al., 2012).

The Behavior Rating Inventory of Executive Functions (BRIEF) is a behavior rating scale used to screen for executive function in children of ages 5 to 18 years. Parents and teachers complete the BRIEF questionnaire and ask how the child behaves in everyday situations, particularly those that require problem solving. Children with ASD have been found to have significantly elevated BRIEF scores compared with neurotypical children, corresponding to executive function ability (Blijd-Hoogewys et al., 2014).

The Autism Diagnostic Observation Schedule (ADOS), used by many clinicians as the gold standard of ASD diagnosis, is a semi-structured assessment of social communication, social interaction, and repetitive behaviors in individuals suspected to have autism spectrum disorder (ASD). It is administered by trained clinicians who select the appropriate module based on the individual’s developmental and language level, with metrics targeting areas such as quality of social responses, reciprocity, and restricted or repetitive behaviors (Kamp-Becker et al., 2018).

Autism Diagnostic Interview-Revised (ADI-R), a caregiver interview conducted by clinicians, assesses behaviors associated with autism. ADI-R measures social communication, restricted and repetitive behaviors, and developmental milestones, providing information on symptoms through detailed, structured questions (Snow et al., 2009).

The Repetitive Behavior Scale-Revised (RBS-R) evaluates an array of restrictive, repetitive behaviors (RRBs) an individual with ASD may exhibit. RRBs, a common presentation of ASD, are divided into subscales of stereotypic behaviors, self-injurious behaviors, compulsions, ritualized behaviors, insistence on sameness, and restricted interests (Lam & Aman, 2007). Caregivers self-report the RBS-R questionnaire. As RRBs are a hallmark of ASD, children with ASD are expected to score higher on subscales of the RBS-R.

The Adolescent/Adult Sensory Profile (AASP) measures sensory processing and individual sensory preferences that lead to behaviors in participants 11 years and older. AASP is a self-reported questionnaire that scores components such as sensory sensitivity and avoidance for the five senses. Children with ASD have been found to have altered sensory processing behaviors that match in intensity but differ in processing patterns from neurotypical children (Crane et al., 2009).

The Child Behavior Checklist (CBCL) component of the Achenbach System of Empirically Based Assessment assesses a range of behavioral and emotional syndromes, including anxiety, depression, aggression, and defiant behavior problems. The syndromes are grouped into internally and externally focused behaviors and emotions. The CBCL questionnaire is administered to parents of children of ages 6 to 18 years. Children with ASD have been found to have higher scores on CBCL subscales for depression, social problems, thought problems, and attention problems than neurotypical children (Arias et al., 2022).

The Social Responsiveness Scale version 2 (SRS-II) is a rating scale measuring behavioral associated with ASD and can be completed by raters with at least 1 month of experience with the rated individual. Different rating forms are available for age groups, including a self-report form for individuals aged 19 years and up. The SRS-2 focuses on social differences and each item is responded with a 4-point Likert scale rating (Bruni, 2014; Constantino & Gruber, 2012).

The Vineland Adaptive Behavior Scales—II (Vineland-II) is utilized for the assessment of social and adaptive functions, also termed social competency. Vineland-II is sometimes used as a substitute for traditional intelligence testing in situations where the participant’s verbal ability is inadequate or there is active psychopathology due to the shortness and ease of administration (Doll, 1935).

The Differential Ability Scales (DAS) is a cognitive battery designed to test a range of abilities with a narrower and more specific domain than general intelligence tests. The DAS is intended to provide a profile of specific cognitive strengths and weaknesses to educators in order to tailor interventions. The various subtests are altered depending on participant age and ability and subdivisions cover verbal and nonverbal domains (Elliott et al., 1990, 2007) (Table 2).

**Table 2.** Description of all behavioral scales and subscales utilized in this study along with shorthand labels used to refer to the metrics throughout the rest of the figures.

Test name	Focus	Administrator	Subscales	Labels	Standardized or raw score
The Clinical Evaluation of Language Fundamentals—Fourth Edition (CELF-4)	Receptive language-skills including phonology, morphology, syntax, semantics, and working memory	Clinician	Recalling sentences	celf4_rs_scld_scr	Standardized
			Formulating sentences	celf4_fs_scld_scr	Standardized
			Concepts and following directions	celf4_cfd_scld_scr	Standardized
			Word classes (receptive)	celf4_wc2r_scld_scr	Standardized
			Word classes (expressive)	celf4_wc2e_scld_scr	Standardized
			Word classes (total)	celf4_wc2t_scld_scr	Standardized
			Word definition	celf4_wd_scld_scr	Standardized
			Word structure	celf4_ws_scld_scr	Standardized
			Core language	celf4_cl_ss	Standardized
The Behavior Rating Inventory of Executive Functions (BRIEF)	Executive function	Caregiver	Planning and organizing	brief_p_po	Standardized
			Organization of materials	brief_p_om	Standardized
			Monitor	brief_p_monitor	Standardized
			Inhibit	brief_p_inhibit	Standardized
			Shift	brief_p_shift	Standardized
			Emotional control	brief_p_ec	Standardized
			Initiate	brief_p_iniitiate	Standardized
			Working memory	brief_p_wm	Standardized
			Behavioral regulation	brief_p_bri	Standardized
			Metacognition	brief_p_mi	Standardized
			Global executive	brief_p_gec	Standardized
The Autism Diagnostic Observation Schedule Revised (ADOS-2)	Social and behavioral features associated with ASD	Clinician	Social affect	Socialaffect	Sum of raw scores
			Social comparison	Scoresumm_compscore	Sum of raw scores
			Behavioral profile	behavioraltotal	Sum of raw scores
			ADOS total score	Scoresumm_overaltotal	Sum of raw scores
Autism Diagnostic Interview-Revised (ADI-R)	Social and behavioral features associated with ASD	Clinician	Language acquisition (age in months of first word)	acqorlossoflang_aword	Neither
			Language acquisition (age in months of first phrase)	acqorlossoflang_aphrase	Neither
			Loss of language after acquisition (binary)	loslang	Neither
			Communication profile	dbaes_atotal	Sum of raw scores
			Social behavior profile	dbaes_bvtotal	Sum of raw scores
The Repetitive Behavior Scale-Revised(RBS-R)	Specifically examines repetitive behavior	Caregiver	Stereotyped behavior	rbsr_stereo_total	Sum of raw scores
			Self-injurious behavior	rbsr_self_injurous_total	Sum of raw scores
			Compulsive behavior	rbsr_compulsive_total	Sum of raw scores
			Ritualistic behavior	rbsr_ritualistic_total	Sum of raw scores
			Sameness behavior	rbsr_sameness_total	Sum of raw scores
			Restricted behavior	rbsr_restricted_total	Sum of raw scores
			RBS-R total score	rbsr_overall_total	Sum of raw scores
The Adolescent/Adult Sensory Profile (AASP)	Sensory processing and preferences	Both—see notes	Sensory seeking	sensory_c_fg1 (caregiver)	Standardized
			Emotionally reactive	sensory_c_fg2 (caregiver)	Standardized
			Low endurance/tone	sensory_c_fg3 (caregiver)	Standardized
			Oral sensory sensitivity	sensory_c_fg4 (caregiver)	Standardized
			Inattention/distractibility	sensory_c_fg5 (caregiver)	Standardized
			Poor registration	sensory_c_fg6 (caregiver)	Standardized
			Sensory sensitivity	sensory_c_fg7 (caregiver)	Standardized
			Sedentary	sensory_c_fg8 (caregiver)	Standardized
			Fine motor/perceptual	sensory_c_fg9 (caregiver)	Standardized
			Low registration	sensory_a_qg1 (self)	Standardized
			Sensation seeking	sensory_a_qg2 (self)	Standardized
			Sensory sensitivity	sensory_a_qg3 (self)	Standardized
			Sensation avoiding	sensory_a_qg4 (self)	Standardized
The Child Behavior Checklist (CBCL)	Behavioral and emotional syndromes, including anxiety, depression, aggression, and defiant behavior problems	Caregiver	Anxious depressed	cbcl_anxious	Standardized
			Somatic complaints	cbcl_somatic_c	Standardized
			Withdrawn	cbcl_withdrawn	Standardized
			Attention problems	cbcl_attention	Standardized
			Aggressive behavior	cbcl_aggressive	Standardized
			Internalizing problems	cbcl_internal	Standardized
			Externalizing problems	cbcl_external	Standardized
			Affective problems	cbcl_affective	Standardized
			Anxiety problems	cbcl_anxiety	Standardized
			Attention deficit/hyperactivity	cbcl_adhd	Standardized
			Oppositional defiant problems	cbcl_oppositional	Standardized
			Social problems	cbcl_social_p	Standardized
			Thought problems	cbcl_thought	Standardized
			Rule-breaking behavior	cbcl_rulebreak	Standardized
			Somatic problems	cbcl_somatic_p	Standardized
			Conduct problems	cbcl_conduct	Standardized
			CBCL total score	cbcl_total	Standardized
Social Responsiveness Scale (SRS-II)	Social behaviors associated with ASD	Caregiver	Social awareness	awr_raw	Raw
			Social cognition	cog_raw	Raw
			Social communication	com_raw	Raw
			Social motivation	mot_raw	Raw
			Restricted interest and repetitive behavior	rrb_raw	Raw
			Social awareness (T-scored)	awr_tscore	Standardized
			Social cognition (T-scored)	cog_tscore	Standardized
			Social communication (T-scored)	com_tscore	Standardized
			Social motivation (T-scored)	mot_tscore	Standardized
			Restricted interest and repetitive behavior (T-scored)	rrb_tscore	Standardized
			SRS total score	srs2_rawscore	Raw
			SRS total score (T-scored)	srs2_tscore	Standardized
Vineland Adaptive Behavior Scales—II (Vineland-II)	Measurement of adaptive behavior skills.	Caregiver	Communication	communicationdomain_totalb	Standardized
			Living skills	livingskillsdomain_totalb	Standardized
			Socialization	socializationdomain_totalb	Standardized
			Adaptive behavior	composite_totalb	Standardized
Differential Ability Scales (DAS)	Primary focus on cognitive abilities	Clinician	Verbal reasoning	verbal_ss	Standardized
			Nonverbal reasoning	nvr_ss	Standardized
			Spatial reasoning	spatial_ss	Standardized
			General conceptual ability	gca_ss	Standardized
			DAS summary score	snc_ss	Standardized

### 2.3 Statistical analysis

All behavioral and cognitive test results were compared with the mean microstructural values in each ROI using general linear models. All models featured sex-as-assigned at birth, participant age, full scale IQ, scanner/evaluation site, and intracranial volume as control terms. All resulting aggregate g-ratio, conduction velocity, and brain–behavior interaction p-values were corrected for multiple comparisons using the Benjamini & Hochberg (1995) method across all 214 ROIs. Test scales were not batch altered or z-scored for linear model testing as several tests are highly bimodally distributed in scoring by design, with non-autistic individuals frequently scoring at or near 0. There was no directional encoding performed as not all of the behavioral metrics have clear or distinct relationships to impairment, or are not ASD specific, with some subscales showing no difference between ASD and non-ASD participants or ASD participants scoring higher or lower than non-ASD participants in different subscales.

### 2.4 Image acquisition

Diffusion, T1-weighted, and T2-weighted images were acquired from each subject. Diffusion images were acquired with an isotropic voxel size of 2 x 2 x 2mm^3^, 64 non-colinear gradient directions at b = 1000 s/mm^2^, and 1 b = 0, TR = 7300 ms, TE = 74 ms. T1-weighted MPRAGE images with an FOV of 176 x 256 x 256 and an isotropic voxel size of 1 x 1 x 1mm^3^, TE = 3.3; T2-weighted images were acquired with an FOV of 128 x 128 x 34 with a voxel size of 1.5 x 1.5 x 4mm^3^, TE = 35.

### 2.5 Image data processing

All image data were processed per the protocol described in Newman et al. (2024) to generate aggregate g-ratio and aggregate conduction velocity maps. In brief, preprocessing was performed following prior work (Newman, Dhollander, et al., 2020); diffusion images were denoised (Veraart et al., 2016), corrected for Gibbs ringing artifacts (Kellner et al., 2016), and corrected for inhomogeneity fields using FSL’s *topup* and *eddy* commands utilizing outlier detection and replacement (Andersson et al., 2003, 2016; Andersson & Sotiropoulos, 2016), The final preprocessed diffusion images were up-sampled to an isotropic voxel size of 1.3 x 1.3 x 1.3 mm^3^ (Greenspan, 2009). WM, GM, and CSF tissue response functions were generated using the Dhollander algorithm (Dhollander et al., 2016), and single-shell 3-tissue-constrained spherical deconvolution was used to generate the WM fiber orientation distribution (FOD) and GM and CSF representations. 3-Tissue Constrained Spherical Deconvolution (Dhollander et al., 2017; Kelly et al., 2022; Mito et al., 2018, 2020) was used to calculate the voxel-wise maps of the fraction of signal arising from each of three compartments: an intracellular anisotropic, intracellular isotropic, and extracellular isotropic freely diffusing water compartment by setting the sum of all FOD coefficients equal to unity. WM-FODs were then used to create a cohort-specific template with a subset of 40 individuals counterbalanced between sex and diagnosis (D. Raffelt et al., 2012). All subject’s WM-FODs were registered to this template using an affine non-linear transform warp, and then the template was registered to a b-value matched template in stereotaxic MNI space (Hsu et al., 2015; Newman, Untaroiu, et al., 2020). A fixel-based morphometry (FBM) (D. Raffelt et al., 2012; D. A. Raffelt et al., 2017) approach was used to estimate the intra-axonal cross-sectional area within each voxel to be used as an apparent axonal volume fraction (AVF). Each subject’s AVF maps were then registered to MNI space using the ANTs SyN nonlinear registration technique by aligning each to the 11-year-old adolescent template developed by Richards et al. Note that a template approximately 1 standard deviation below the mean age of this study was used to better register the comparatively smaller younger subjects (Richards & Xie, 2015; Richards et al., 2016). T1w and T2w images were processed as described in the MICA-MNI pipeline (Cruces et al., 2022), including N4-bias correction (Avants et al., 2009), rescaling both images from 0 to 100, co-registration using a rigid transform, and subsequently non-linear ANTs SyN registration to the same Richards et al., template as the diffusion-based images (Avants et al., 2014; Richards et al., 2016). While there are noted shortcomings to using T1w/T2w ratio to measure myelin in white matter regions (Sandrone et al., 2023), the method has also been shown to correlate well with myelin in the cortex (Glasser & Van Essen, 2011; Sandrone et al., 2023). No calibration or adjustments were performed because g-ratio values are generally not well established in adolescents, and there is a desire not to alter or introduce additional error to g-ratio measurements before proceeding to aggregate conduction velocity calculation.

### 2.6 Code accessibility

All work was performed using publicly available software, and additional code for calculating g-ratio and conduction velocity from WM FODs and T1w/T2w ratio images can be found here: https://github.com/btn6sb/Conduction_Velocity

### 2.7 Aggregate g-ratio and conduction velocity calculation

Both metrics were calculated according to previously published methods (Newman et al., 2024). The aggregate g-ratio was calculated on a voxel-wise basis according to Stikov et al. and was used according to Mohammadi & Callaghan, as displayed in Equation 1 (Campbell et al., 2018; Mohammadi & Callaghan, 2021; Stikov et al., 2011, 2015). As a measure of intra-axonal volume, the fiber density cross section was used as the AVF (D. Raffelt et al., 2012). As a metric of myelin density, the T1w/T2w ratio was used as the myelin volume fraction (MVF). These metrics represent the total sums of each respective compartment across the volume of the voxel and are a volume-based equivalent to the original formulation of g as the ratio of axon diameter (d) to fiber diameter (D).

$$g=\;\frac{d}{D}=\sqrt{1-\frac{MVF}{MVF+AVF}}$$ (1)

Aggregate conduction velocity was calculated based on the calculations of Rushton (1951) and Berman et al. (2019), reiterating Rushton’s calculation that conduction velocity (θ) is proportional to the length of each fiber segment (l) and that this is roughly proportional to *D,* which in turn can be defined as the ratio between *d* and the g-ratio (g). Furthering the considerations of Rushton, Berman et al. show that a value proportional to conduction velocity can be calculated using axon diameter and the g-ratio as in Equation 2 (Berman et al., 2019):

$$\theta \;\propto l\;\propto Dg\sqrt{-\ln\left(g\right)}\propto d\sqrt{-\ln\left(g\right)}.$$ (2)

Aggregate g-ratio and conduction velocity were averaged across 214 ROIs from the JHU-ICBM WM atlas (48 ROIs) (Mori et al., 2005) and the Destrieux Cortical Atlas (164 ROIs) (Destrieux et al., 2010). Additionally, two composite ROIs were included, 1 of all 48 JHU ROIs and 1 of 150 neocortical regions from the Destrieux Atlas.

### 2.8 Cluster analyses

To further investigate the relationship between the behavioral and cognitive metrics and brain microstructure beyond the ROI level, we employed two separate machine learning cluster analysis techniques in an exploratory manner. These clustering techniques allow for examining the relationship between participants and brain imaging metrics by grouping participants, brain areas, and behavioral and cognitive metrics based on similarity across metrics. In performing these analyses, we aimed to explore whether groups or clusters of participants features exist that describe subtypes or groups within the cohort. We also demonstrated the utility of cluster analysis for analyzing structural neuroimaging data. We applied Clustering Hierarchy Optimization by Iterative Random Forests (CHOIR) to examine similarities across participants. This clustering analysis was performed using the respective R packages for each method.

CHOIR is a repeated iterative random forest and statistical permutation method based on distinctive features for clustering data. Initially designed for single-cell analysis, where, for example, a cluster might denote a specific biologically distinct cell type or population, here we substitute participants for cells and use the mean aggregate g-ratio and conduction velocity in each ROI as features. CHOIR generates robust clusters through an iterative random permutation testing procedure that merges clusters that fail the prediction testing (Petersen et al., 2024). After the final clusters are generated, we can recover the original identity of participants and observe whether the final clusters correspond with demographic, behavioral, or cognitive variables of interest. As the clustering is performed using only neuroimaging metrics, the correspondence of clusters to other variables suggests that particular groupings of neuroimaging results across ROIs are associated with these variables.

## 3 Results

### 3.1 Behavioral metrics

**Fig. 1.**
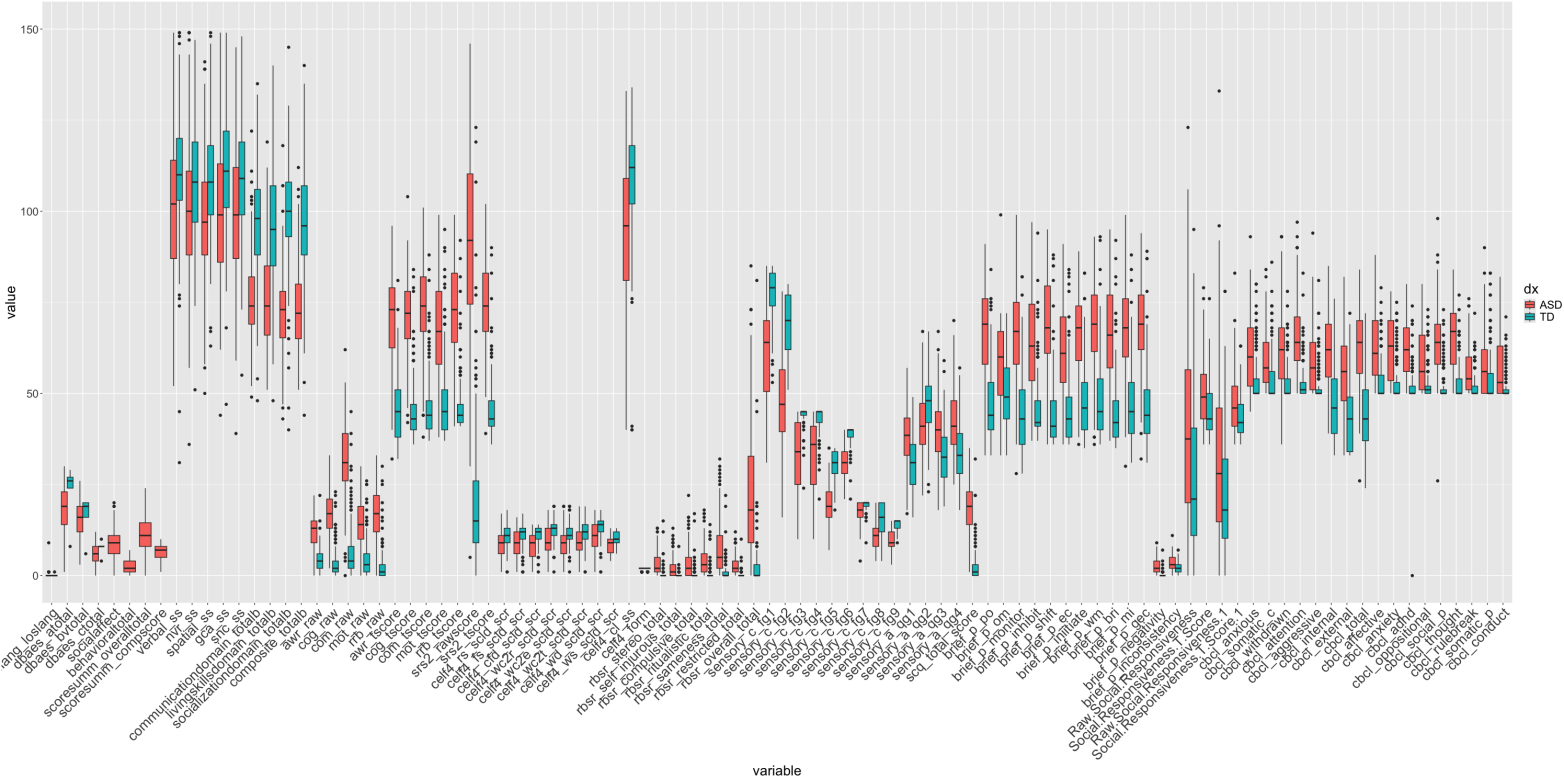
Boxplots with median, upper, and lower quartile (75^th^ & 25^th^ percentile scores, respectively), lines denoting 1.5x the inter-quartile range, and individual scores beyond that range, for each subscale metric and each test administered in this study. ASD participants typically had a higher median score than non-ASD participants with the exception of the CELF and Vineland-II tests. Most tests reliably scored either ASD or non-ASD subjects higher or lower at the group level, with the exception of the AASP.

Figure 1 presents the distribution of summary scores and individual subtest results across different measures for individuals with ASD (in red) and neurotypical individuals (in blue). These results represent one of the most comprehensive cognitive and behavioral batteries ever assembled for assessing autism spectrum disorder (ASD), including multiple commonly applied child behavior assessments, providing an analysis of the relationships between diverse domains such as sensory processing, executive function, language, and social behaviors. We present data from all subscales assessed as well as summary total scores. Compared with individual subscale scores, the summary scores exhibit narrower interquartile ranges and smaller variances than the individual subtest scores, which show a broader spread and higher variability. This contrast is consistent across most measures, with subtest results reflecting a greater range of values within each diagnostic group. On the BRIEF, CBCL, RBS-R, SRS-2, the ASD participants had higher mean scores on each metric than the non-ASD participants, while on the Vineland-II and CELF, the ASD participants had lower mean scores on each metric than the non-ASD participants. The AASP was mixed with the ASD participants having a higher score in 10 metrics and non-ASD participants having a higher mean score in 3 metrics.

The variation in sub-scales that measure unique behavior patterns is potentially masked by summary scores that determine diagnostic criteria. Individual subtest scores demonstrate greater variability than the more narrowly distributed summary scores, suggesting that the nuances in specific behaviors—such as those measured by the BRIEF, CBCL, RBS-R, SRS-2, Vineland-II, CELF, and AASP—may not be fully represented by aggregate measures often used in diagnostic contexts. Similarly, the mixed results in the AASP indicate that even within a single diagnostic group, there is considerable variability in response to specific sensory-related items. This complexity is potentially lost when only summary scores are used, as they may conceal these behavior-specific variations, resulting in a less specific understanding of the individual’s profile.

### 3.2 Correlation analysis

**Fig. 2.**
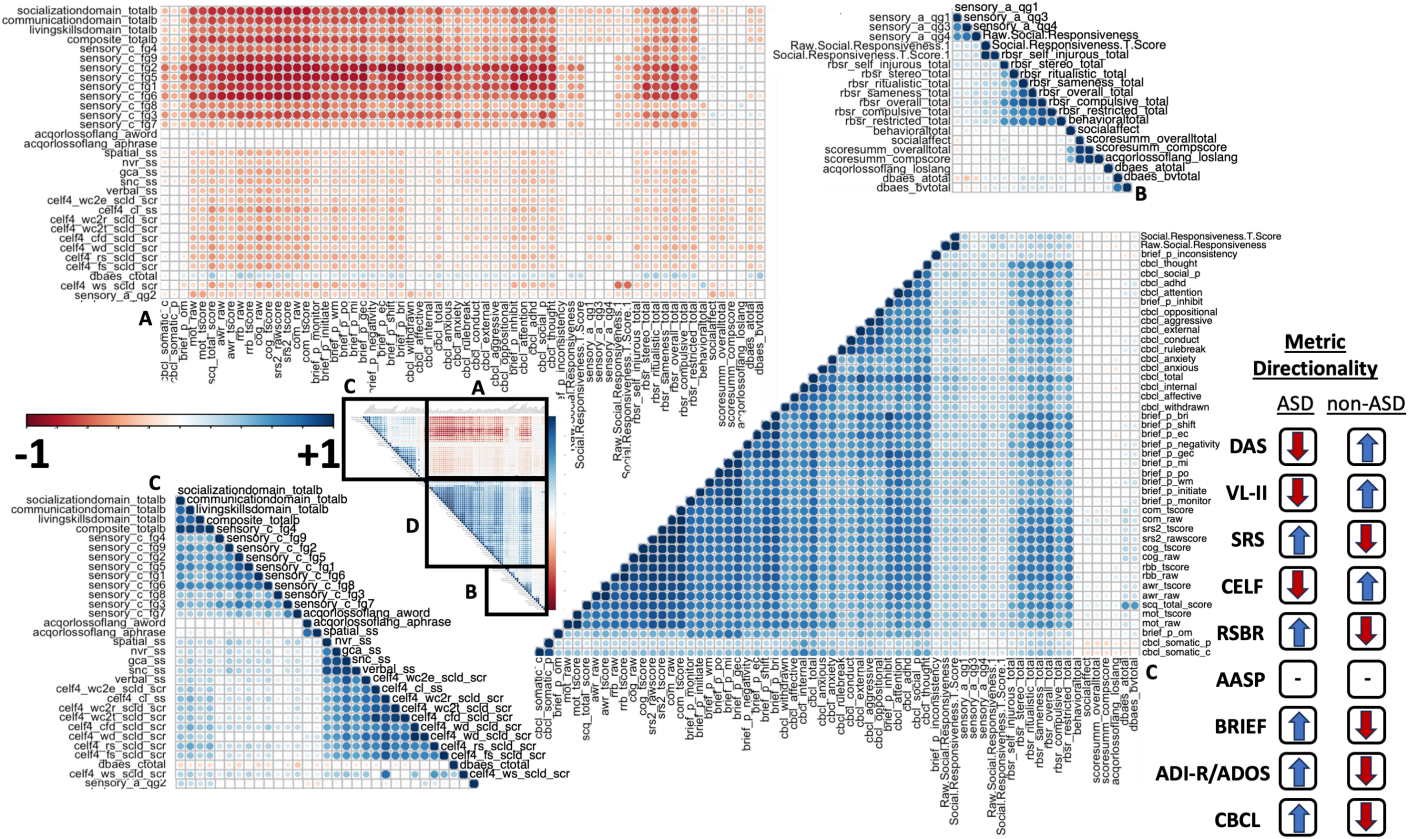
Correlation plot showing the Pearson correlation coefficient between each of the cognitive and behavioral test subdomains as well as composite/total scores. The directionality of each metric is displayed in the legend to the right, based on the results presented in Figure 1. To assist with interpreting metric directionality, higher mean values in ASD subjects or non-ASD subjects are indicated in an upward blue arrow or downward facing red arrow for each test. The AASP was the only test with mixed mean results between ASD and non-ASD subjects, with some subdomains scoring higher in ASD participants and some scoring higher in non-ASD participants. Subdomains of tests were generally highly positively correlated within tests, but there was significant negative correlations between tests, particularly the AASP/CELF and behavioral metrics. Tests measuring behavioral and social functioning tended to be highly positively correlated. Behavioral metrics were divided into four groups based on hierarchical clustering. The primary cluster (bottom left) included the CELF and AASP subscale C, the secondary cluster (bottom right) included the highly correlated BRIEF and CBCL, and the final cluster (top right) included the ADOS and RBSR. The underlying data composing column labels, p-values, multiple-comparison adjusted p-values, and correlation coefficients are listed in Supplementary Table S1.

Results from all tests across all subjects were analyzed using a simple Pearson correlation analysis to observe cross-test associations (Fig. 2). There were widespread significant correlations between individual subscales, particularly within tests (for column labels, p-values, and correlation coefficients that compose this figure, see Supplementary Table S1). Interestingly there was widespread positive correlations between the CBCL, RBS-R, and BRIEF, and a second group of less positive correlations between the AASP and CELF-4 with these two groups significantly negatively correlated from each other, suggesting a split between sensory and language processing and behavioral and social domains.

The Adolescent/Adult Sensory Profile (AASP) as well as the Clinical Evaluation of Language Fundamentals (CELF) displayed strong internal correlations among their own submetrics. However, the CELF did not show significant correlations with any other metrics, including the BRIEF (Behavior Rating Inventory of Executive Function). This lack of correlation suggests that the CELF captures unique information about language abilities that is not aligned with the constructs measured by other assessments. The subscales measuring social metrics, such as those within the AAS (Autism Spectrum-related Social Metrics), were poorly correlated with other domains, indicating a distinct construct that may not overlap substantially with sensory, executive function, or language domains. Interestingly, the summary scores of the ADOS (Autism Diagnostic Observation Schedule), considered the gold standard for autism diagnosis, did not correlate significantly with any other measures in the analysis, suggesting that the ADOS integrates individuals with several different behavioral profiles that may not share scores on individual domains, further supporting that ASD subtypes may appear in the behavioral results.

### 3.3 Linear modeling

**Fig. 3.**
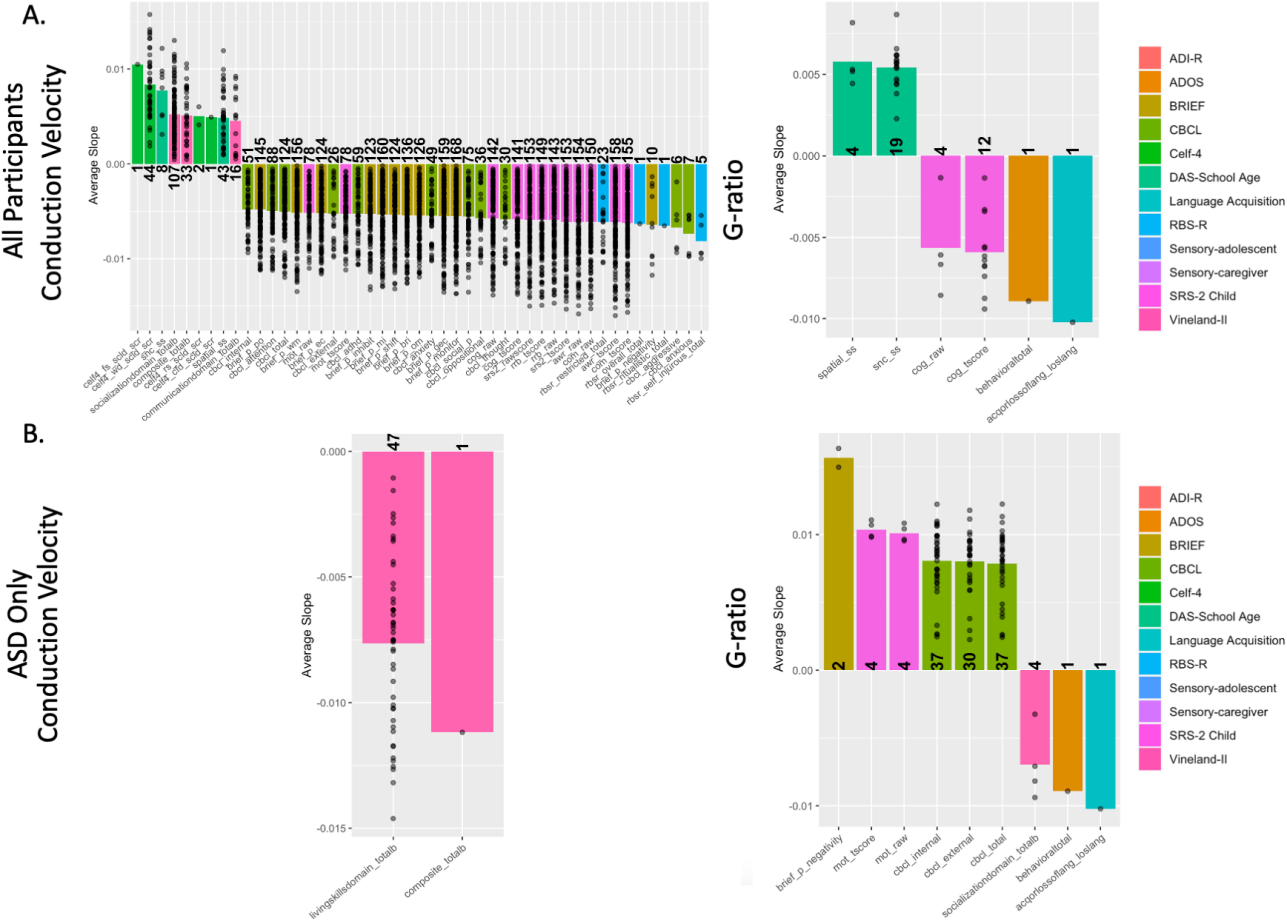
Bar charts showing the mean slope of ROIs significantly associated with each subscale metric, colored by parent metric and separated by conduction velocity or g-ratio. The number on the bar specifies the number of ROIs significantly associated with each metric after multiple comparison corrections, points represent the slope of individual ROIs, all significant ROIs were either entirely positive or negative for each behavioral metric. Charts show relationships between brain cellular microstructure from a sample that includes all autistic and non-autistic participants (A) or exclusively participants diagnosed with ASD (B).

**Fig. 4.**
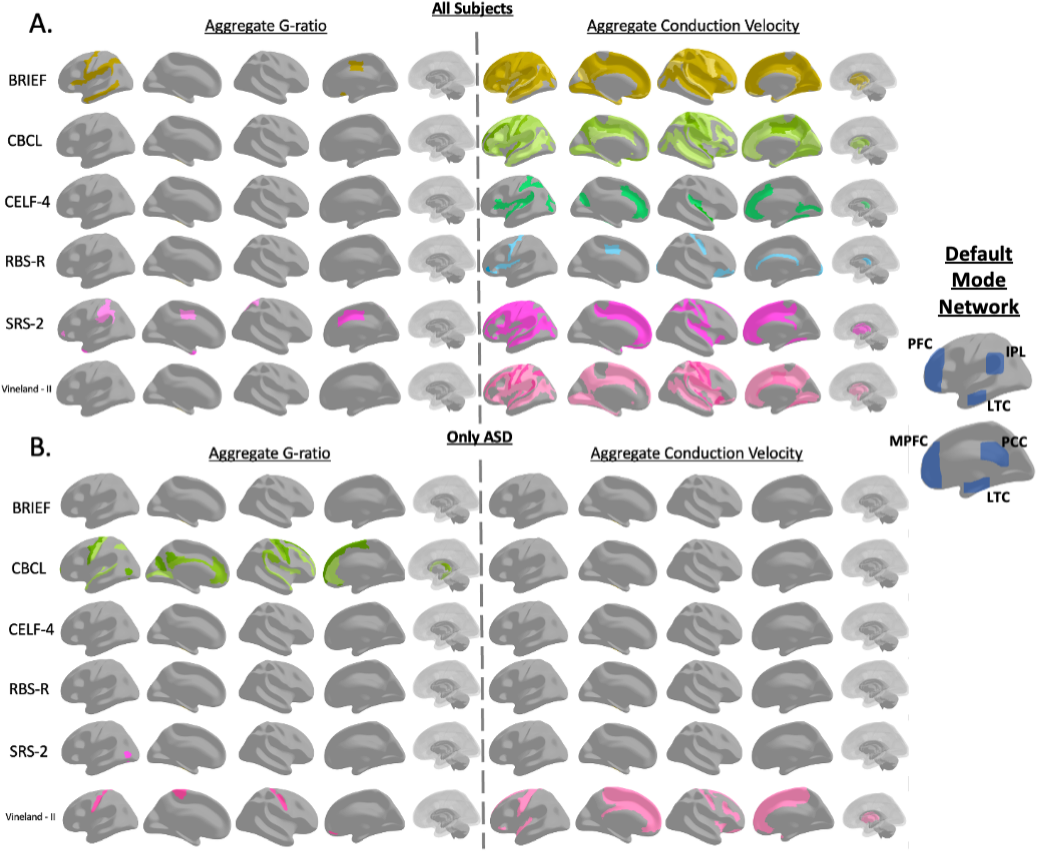
Illustrations showing the location of ROIs significantly associated with each significant parent evaluation. Color is consistent with the bar charts in Fig. 3 and is darker if the region is associated with more subscales within the parent evaluation. Illustrations show relationships between brain cellular microstructure from a sample that includes all autistic and non-autistic participants (A) or exclusively participants diagnosed with ASD (B). The ROIs involved in the DFN are displayed to right to better contextualize the ROI results. The prefrontal cortex (PFC), medial prefrontal cortex (MPFC), inferior parietal cortex (IPC), lateral temporal cortex (LTC), and posterior cingulate cortex (PCC) are functionally active in the DMN. DMN inset adapted from Buckner (2013).

When examining data from all participants, conduction velocity was significantly associated with 47 different subscales in at least 1 ROI (for full list of number of ROIs and average slope of relationship, see Supplementary Table S2. For all other linear model results in all ROIs, see Supplementary Tables S3–S6). Subscales with significant associations came from the BRIEF, RBS-R, CELF-4, CBCL, SRS-2, and Vineland-II tests. The BRIEF test, in particular, had significant subscales in many ROIs (Fig. 3). For example, the monitor subscale, which measures an individual’s ability to monitor plans, thoughts, and emotions, was significant for conduction velocity in 168 ROIs after multiple comparison corrections. Significant ROIs were located across a wide range of cortical ROIs but were especially prominent in the superior parietal, prefrontal cortex, and subcortical gray matter (Fig. 4).

When considering the same dataset, g-ratio was not as widely nor strongly associated across ROIs with behavioral metrics. Only seven different subscales were significantly associated with at least one ROI. Significant subscales for g-ratio were from the DAS-School Age, SRS-2, BRIEF, ADOS, and Language Acquisition tests. The most significant associations were found in the deep WM in the BRIEF and DAS, associated with 19 different ROIs. Across their different subscales, the CELF-4 was positively associated with conduction velocity, while RBS-R was negatively associated with conduction velocity.

While conduction velocity had a much stronger pattern of significance when considering all the data, when associations were considered exclusively within the autistic participants, g-ratio was more strongly associated with behavioral metrics across several ROIs. In ASD-only data evaluations, conduction velocity was significant for only three subscales from the Vineland-II and BRIEF tests. The composite total metric of Vineland-II, which was significant in 33 ROIs when analyzing data from all participants, was only significant for one ROI when considering solely ASD participants. g-ratio, conversely, was significant for nine different subscales derived from the BRIEF, ADOS, CBCL, Language Acquisitions, SRS-2, and Vineland-II tests. In particular, g-ratio was more strongly associated with the CBCL test than conduction velocity, and the total score of the CBCL subscales was significant for g-ratio in 37 ROIs. g-ratio relationships were primarily located in the sensorimotor cortex, insular cortex, and WM. Subscales of the SRS-2 demonstrated powerful associations across analyses with multiple significant areas in g-ratio tests when considering all participants and only those with ASD. A particular series of ROIs overlaps with regions associated with the Default Mode Network, such as the anterior medial prefrontal, posterior cingulate, and the temporal–parietal junction and is associated with the CBCL in ASD participants but with multiple behavioral scales in the full cohort.

### 3.4 CHOIR cluster analysis

**Fig. 5.**
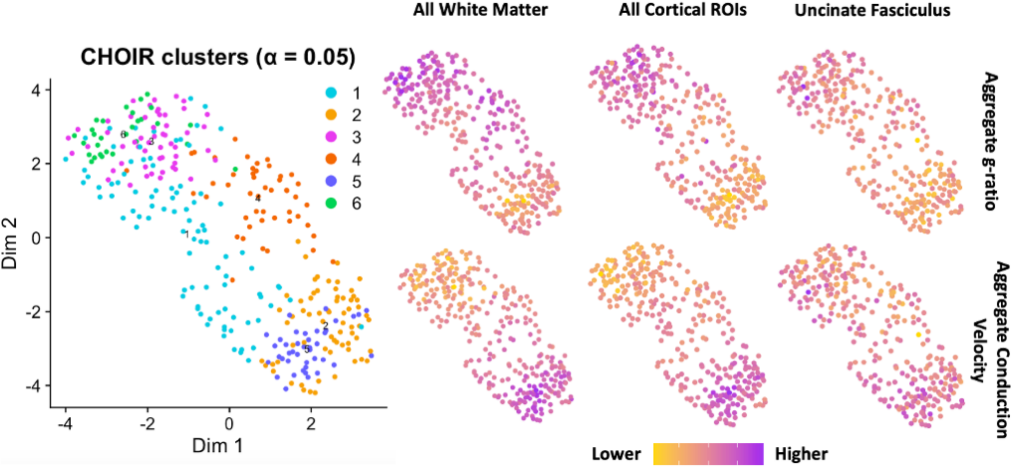
Results of the CHOIR cluster analysis. CHOIR identified six total clusters from the neuroimaging data alone. These tended to be aligned along a gradient with a high aggregate conduction velocity and low aggregate g-ratio pole at clusters 2 and 5 and, in the inverse, a high aggregate g-ratio and low aggregate conduction velocity pole at clusters 4 and 6.

**Fig. 6.**
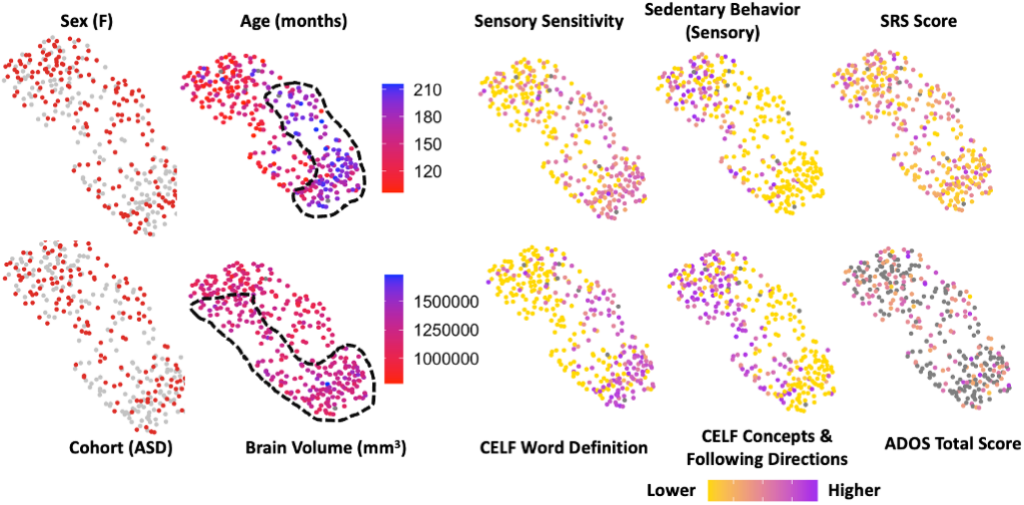
Demographic, behavioral, and cognitive metrics displayed over CHOIR clusters presented previously in Figure 5. None of these metrics were used in the computation of the clusters. Sex and ASD diagnosis were not strongly related to any cluster, but age and brain volume were starkly divided. Several behavioral measures also showed stark divides, mainly following the age-related clustering but differing directions between different subscales. Interestingly this occurs among some subscales, such as the CELF Word Definition and CELF Concepts & Following Directions that are standardized for age, suggesting that age-related maturation may be mediated by neuronal microstructural change. Some metrics, such as the SRS and ADOS (ASD only), did not display mapping onto the defined clusters.

CHOIR analysis of the 414 total ROIs (one separate contribution from the mean conduction velocity and mean g-ratio within each of the 212 ROIs used in the study) resulted in a total of 6 distinct clusters that were generally associated with a gradient between higher aggregate g-ratio and lower conduction velocity at 1 pole (lower microstructural maturity) and lower aggregate g-ratio and higher conduction velocity at the other pole (higher microstructural maturity; Fig. 5). This pattern was broadly similar across all the ROIs used to generate the clusters (Supplementary Fig. S1). Demographically, the clusters did not appear to be predictive of subject sex or ASD diagnosis (Fig. 6). However, there was a gradient for subject age, with clusters 2, 4, and 5 having a higher mean subject age than the overall mean and clusters 1, 3, and 6 having a lower mean subject age than the overall mean. Interestingly, this pattern was slightly different for brain volume, with clusters 1, 2, and 5 having higher mean subject brain volume than the overall mean and clusters 3, 4, and 6 having a lower mean subject brain volume than the overall mean. This allows the division of the clusters into four general patterns based on demographics: a lower maturity, younger cluster with low brain volume (clusters 3 and 6), a higher maturity, older cluster with high brain volume (clusters 2 and 5), a cluster with mixed microstructural maturity, younger, with high brain volume (cluster 1), and a cluster with mixed microstructural maturity, older, with low brain volume (cluster 4).

When the behavioral and cognitive test results were projected onto the clusters, the pattern generally appeared to follow the age-related poles defined earlier, with subscales having a high/low gradient of scoring matching the younger/older divide for clusters 3 and 6 and 2 and 5. However, within the same test, such as the CELF and AASP Sensory Profile, this polarity was switched between different items (Fig. 6). Age, rather than brain volume, appeared to be more indicative of which pole the intermediate clusters (1 and 4) were aligned to. The complete list of all behavioral results projected onto the CHOIR cluster is available in Supplementary Figure S2.

**Fig. 7.**
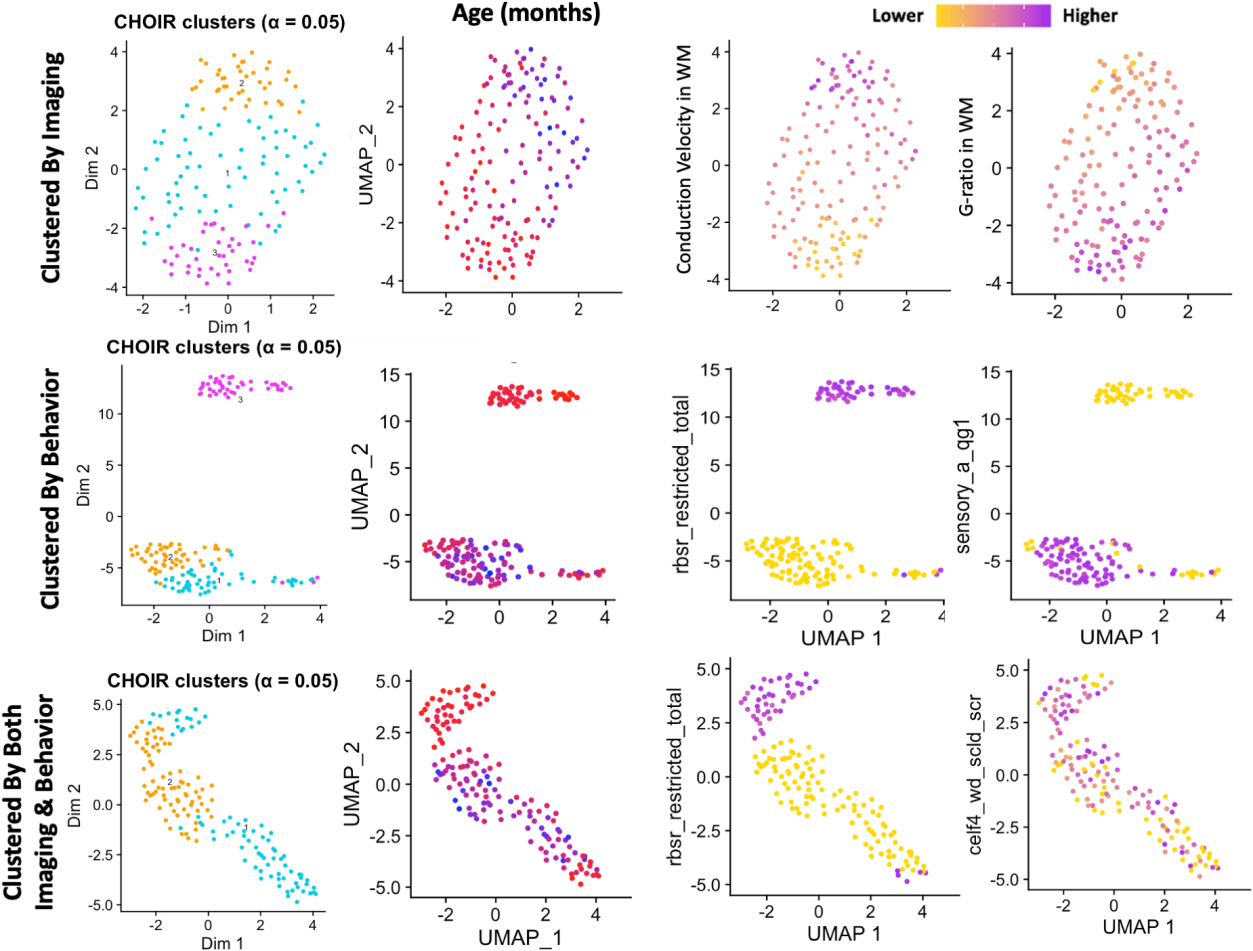
CHOIR clusters of ASD participants only using just neuroimaging data (top row), behavioral metrics (middle row), or all metrics from both behavior and neuroimaging (bottom row). All cluster results were polarized by age (second column), with a particular cluster of the youngest participants in the study clearly observable (red). Age was not used to cluster any subjects, indicating that an age gradient was able to be reconstructed using exclusively the neuroimaging and behavioral metrics. Behaviors showed clear differences between the age-delineated clusters. With repetitive behaviors and language and sensory sensitivity metrics showing an inverse relationship indicating two distinct behavioral profiles.

A final CHOIR analysis was performed on only ASD participants, both clustered using behavioral results, neuroimaging results, and using all the metrics available both behavioral and neuroimaging (Fig. 7). While the first two analyses delineated three clusters of ASD subjects, the combined analysis delineated two larger clusters. In each case, age was a vital factor that polarized the groups, with separate clusters for younger subjects in particular. In each case, a younger group with high scores on restricted and repetitive behavior was separated from other participants who had lower performance scores on language skills and higher scores on sensory sensitivity (The CHOIR results from all behavioral and neuroimaging metrics are available in Supplementary Fig. S3). This suggests that age polarizes two distinct clusters of ASD participants with common behavioral features. The same pattern is observed when neurotypical participants are included in Figure 6 where higher CELF language scale scores are paired with higher sensory sensitivity along an age polarized axis.

## 4 Discussion

This study identifies multiple regions associated with particular behavioral and cognitive autism evaluations. Several tests, particularly the CELF-4 and BRIEF, showed excellent associations between brain metrics and individual performance. Cluster analysis identified age as a defining axis for neuronal and behavioral metrics, and identified two distinct and inversely correlated groups of individuals, with restrictive and repetitive behavior in some participants and a second group that was defined by high sensory sensitivity and language performance. Despite differences in assessments, there was much overlap in brain regions associated with the various metrics, mainly when non-autistic participants were included. Associations in white matter regions demonstrated robust associations for both analysis of all participants and ASD-specific tests. However, when only autistic individuals were evaluated, aggregate g-ratio was significantly associated with more ROIs than conduction velocity. This switch from more significant conduction velocity measurements when all participants were assessed to more g-ratio associations when evaluating exclusively autistic participants suggests that the observed neurological differences between autistic and non-autistic individuals may differ from the neurological correlates of autism severity. This finding confirms the consensus among the ASD clinical community that behavioral tests need to be evaluated for effectiveness at assessing along separate dimensions of diagnosis and severity and that any single test may not assess both components well (Hus & Lord, 2014; Lord et al., 2012). By finding widespread findings between behavioral and cognitive tests and brain structure associated with ASD, this study suggests that clinical evaluations by experts and parental assessments are both able to predict differences in neurological structure to at least some degree. While the linear models showed that many different assessments can be reflective of neuronal structure, the cluster analyses suggested that the breadth of assessments within many major tests is important to fully capture individuals who may present different behavioral profiles depending on age. Many of these relationships are found in ROIs associated with the Default Mode Network (DMN), such as the anterior medial prefrontal, posterior cingulate, and the temporal–parietal junction. As our previous work has shown that ASD involves slower axonal transmission speed (Newman et al., 2024) and that the DMN may become less synchronized along the anterior-posterior axis with increasing age (Jones et al., 2011), it is possible that age-related change in network connectivity may drive or mediate age-related alterations in behavioral presentation.

While several of the tests examined in this study showed relationships between brain structure and total or composite scores across the internal metrics of the study, only the CBCL total score was significantly associated with multiple brain regions within the ASD-only cohort, suggesting that the notion of severity within ASD may manifest in highly different ways along different behavioral vectors. Though it should be noted that this was not a formal diagnostic group-based interaction and merely excluded non-ASD participants. Interestingly the CBCL has been found to have higher validity within subscales, particularly anxiety and depression, than the total score (Pandolfi et al., 2014). While we do not delineate specific groups or subgroups with this study, these findings do suggest that differential brain regions may be involved in different symptom profiles, and lends support for clinical examination of multiple subscale scores, rather than composite scores alone. Conversely, it is possible that some of the associations between various subscales and brain microstructure are not unique or wholly specific to ASD, and it is possible that these tests are capturing behavior not unique to ASD (Havdahl et al., 2016).

The unequal distribution of significant results across behavioral and cognitive tests indicates that diagnostic assessments vary in their correlation to brain structure. Prior to the DSM-5 recategorization, the BRIEF assessment was reported to be elevated across subgroups of ASD, including autistic disorder, Asperger’s syndrome, and pervasive developmental disorder (Blijd-Hoogewys et al., 2014). While these diagnostic labels have been discontinued, the high number of associations between microstructural metrics and BRIEF subscales may demonstrate that BRIEF captures a breadth of behaviors, some of which may not be specific to autism alone, in a way that aligns with what is occurring structurally in the brain. The SRS-2 assessment displayed multiple associations with g-ratio when analyzed in the context of all participants and the ASD subgroup specifically. The SRS is a valid predictive measure of ASD across time points in childhood, indicating that it captures consistent autistic traits within a child over time (Chan et al., 2017). This continuity across development may indicate an underlying neurological difference present from a young age, and thus, it has the potential to be detected early on. Conversely, the lack of associations between microstructural metrics and widely used diagnostic assessments, such as the ADOS, suggests that there may be a disjunction between behaviors emphasized in diagnostic measures and those that stem from neurological differences shared across ASD individuals.

Previous work in this same cohort examining aggregate conduction velocity and aggregate g-ratio found that there was limited associations between age and microstructural metrics, with aggregate g-ratio significantly associated with age largely in the cortex but not in the white matter and aggregate conduction velocity significantly associated with age largely in the white matter but not in the cortex (Newman et al., 2024). Moreover, changes observed across large brain regions were relatively small between 8 to 18 years old (approximately 5% change in aggregate conduction velocity and approximately 1% change in aggregate g-ratio in white matter), however, the hierarchical cluster analysis repeatedly generated age-related gradients using both exclusively behavioral metrics (including those standardized for age) and exclusively microstructural metrics. At no point was age or ASD diagnosis used to cluster participants, indicating that these features are distinct enough to be recoverable from the imaging and behavioral metrics even when age is either standardized or not significantly associated with a number of brain ROIs. Previous behavioral work in ASD has suggested that social metrics improve and repetitive behaviors worsen across adolescent development (Bal et al., 2019; Guthrie et al., 2013; Seltzer et al., 2003); however, this study, particularly the lack of a connection between clustered microstructural metrics and ASD diagnosis, suggests that age-related neuronal changes during development are similar between ASD and non-ASD individuals. This suggests that age-related changes are not occurring differentially in ASD and non-ASD adolescents, and that both groups are undergoing equivalent age-related neuronal processes. This, however, raises the question of why ASD symptomology has been observed to change with age and whether this is connected to underlying neuronal development. The CHOIR analysis further shows that different subscales within tests are associated with clusters of different aged individuals, as shown in Figure 6 with high scores on the CELF-Concepts and Following Directions being associated with the youngest clusters and the CELF-Word Definition being associated with the oldest clusters, despite both metrics being similarly scored higher in non-ASD participants and the scores being age standardized. It should further be noted that neither age nor behavioral metrics were used to generate the clusters in Figure 6, the brain metrics alone were sufficient to identify different behavioral groups at different ages. This suggests that age is mediating the brain–behavior relationship in both ASD and non-ASD individuals, and strengthening the association between altered conduction velocity and g-ratio and ASD presentation.

A striking finding is the lack of significant correlations between the summary scores of the Autism Diagnostic Observation Schedule (ADOS), the gold standard of ASD diagnosis, and any other cognitive or behavioral metrics. This lack of correlation suggests that while the ADOS summary scores effectively capture diagnostic criteria for ASD, they may not reflect the underlying individual traits or symptoms measured by other assessments. This finding could indicate that, when broken down, the components of the ADOS summary scores may not align well with specific metrics across a heterogeneous population. This observation highlights the profound heterogeneity inherent to ASD. The diagnosis of autism encompasses a broad spectrum of presentations, with individuals exhibiting diverse profiles of strengths and challenges. As such, a diagnosis of “autism” alone may not provide detailed information about a person’s specific symptomatology or functional abilities. The variability in correlations observed in this study underscores the importance of examining individual dimensions of behavior and cognition rather than relying solely on diagnostic labels to guide intervention or research. While the behavioral and cognitive assessments analyzed in this study utilize a variety of age-standardized and non-standardized scores, the values were compared as most typically reported to support ease of interpretation. Though more rigorous standardization would likely refine the correlation analysis, the different standardization process, for example, providing entirely different questions and test versions for differently defined age groupings, this was not conducive to the extremely large number of tests employed in the present study. Generally, however, the results in this study are consistent between the directionality displayed in Figures 1 and 2, with metrics that are higher in ASD vs. non-ASD being negatively correlated with metrics that are lower in ASD vs. non-ASD and positively correlated with other metrics that are also higher in ASD than in non-ASD.

The observed differences in correlations across behavioral tests may also reflect underlying neurobiological diversity within the ASD population. Tests may tap into distinct neuronal networks or functional systems, particularly the insular and sensorimotor networks as observed in this study, leading to variable patterns of association depending on the metric being assessed. For example, language skills measured by the CELF may rely on different brain regions than those associated with social metrics captured by the AAS or sensory metrics. Within ASD participants, the CBCL subscales were associated with g-ratio in the cingulum, sensorimotor cortex, and superior longitudinal fasciculus, potentially suggesting that the quality of long-range neuronal bundles is most affected by changes in g-ratio and that this results in behavioral changes, rather than disruptions to local processing. When non-ASD participants are also included, a similar spatial pattern appears, which supports the conclusion that global deficits in conduction velocity and g-ratio affect primarily long distance connections rather than a pattern of focal, localized neuronal differences. This is supported by a number of functional MRI studies reporting DMN dysfunction in ASD, particularly dysregulation between DMN nodes, and more localized activity (Harikumar et al., 2021; Padmanabhan et al., 2017; Washington et al., 2014). Alterations in the DMN have been suggested to underlie differences in theory of mind and social communication in ASD (Padmanabhan et al., 2017). The results from this study suggest that DMN network alterations in aggregate conduction velocity may underlie a range of behavioral metrics including BRIEF and CBCL, though the strong association between DMN network nodes and the SRS also suggests a strong social component. Imbalances between excitation and inhibition throughout the network and within processing hubs have been theorized to cause DMN alterations (Padmanabhan et al., 2017; Washington et al., 2014), which would match the findings of altered conduction velocity described in this study. Differential action potential transmission speed is potentially highly disregulatory when a network requires multiple coordinated hubs with high structural integrity, especially via long-distance connections. This may be even more impactful if particular populations of excitatory or inhibitory neurons are more likely to be affected and become less efficient. Effective social communication requires rapid integration and processing of sensory information, including detection of subtle cues. Globally slowed processing in the DMN may thus be strongly associated with social differences due to the specific demands of the task and the vulnerability of the widely distributed DMN network in particular.

Interestingly the frontal cortex did not have a large number of significant ROIs, despite frontal cortex development being a hallmark of adolescence. These distinct neural profiles likely contribute to the variability in test outcomes and further reinforce the need for multidimensional approaches to understanding ASD. Pinpointing which behavioral metrics align with brain structure across ranges of symptom severity may better enable diagnostic tools to accurately distinguish ASD children from those who are neurotypical or have other disorders.

The cluster analysis performed here was unique in adapting both methods, previously designed for single-cell analysis (CHOIR) and psychometric latent factor analysis (EGA) to neuroimaging data. CHOIR can cluster subjects incorporating multiple features per subject, while EGA can associate features across subjects, providing different but complementary approaches. While conclusions drawn from this novel approach should be limited and done cautiously, CHOIR, in particular, appeared to replicate many demographic and behavioral differences from exclusively microstructural neuroimaging data that suggests that accounting for multiple ROI measurements may have value in neuroimaging analysis. The CHOIR results instead elegantly showcase associations between brain metrics and age as being essential markers of change relative to sex or even ASD diagnosis.

Overlap between age-related clusters and different behavioral metrics suggests that the behavioral profile of autism may be modulated by age. Other studies have suggested that some ASD behaviors decrease with age during the end of the developmental period, such as restricted and repetitive behaviors (Esbensen et al., 2009), while other studies have found more complex age-related trajectories of behavioral score increase and decrease (Waizbard‐Bartov et al., 2022). The EGA technique correctly replicated correlations between metrics and replicated clusters of imaging metrics despite being provided with these metrics as independent features, for example, the purple cluster at the bottom of the conduction velocity figure represents the left and right olfactory cortex, left and right nucleus accumbens, and left and right subcallosal gyrus. This symmetry between left and right ROIs appearing in the same cluster was common throughout the EGA results. Conduction velocity appeared to have more distinct clustering of brain regions compared with the three clusters of g-ratio ROIs. This may be due to increased sensitivity to the development of axonal tracts that cross or connect, and are thus shared between, multiple ROIs.

A previous study found that ASD was associated with decreased aggregate g-ratio and conduction velocity but not a significant difference in T1/T2 ratio, demonstrating that neurological differences in ASD may be based on changes in axonal structure and not simply a deficit of myelination (Newman et al., 2024). These changes may cause the observed switch when evaluating ASD participants solely as differences in axonal architecture manifest uniquely in conduction velocity compared with g-ratio. Subtle differences in inner axonal diameter can significantly affect conduction velocity, while g-ratio relies on the balance between axonal diameter and myelin thickness. Based on our observations, ASD participants with more severe behavioral symptoms may have similarly altered ratios between inner axonal diameter and myelin diameter. In contrast, high-performing ASD participants may have a more neurotypical relationship between diameter and myelin. This range of g-ratios within the ASD cohort may underlie the range of behavioral severity seen across the disorder. As g-ratio had more and stronger significant relationships with behavioral metrics than conduction velocity, combined with the lack of significant associations between T1w/T2w ratio, the critical difference affecting g-ratio in ASD participants may be inner axon diameter as opposed to myelin diameter.

These findings are supported by prior post-mortem electron microscopy studies of the corpus callosum, in which ASD subjects were found to have significantly decreased axon diameters and cross-sectional areas (Wegiel et al., 2018). Additionally, a decrease in the percentage of large-diameter axons in all five segments of the corpus callosum was observed in autistic individuals. Prior work with the data used in this study found the same result from microstructural analysis (Newman et al., 2024). Furthermore, the histological study found that autism had a more significant correlation with axon diameter and area than with myelin thickness, agreeing with the explanation that the axonal diameter element of g-ratio has a predominating effect on autism development compared with myelin diameter. The structural abnormalities in axonal development observed in this study demonstrated deficits in interhemispheric connection specificity, which may underlie dysregulation of velocity and volume of information and issues with information processing. This study reinforces our findings that axonal diameter may inform behavior patterns associated with ASD and provides a basis for how this property may alter long-range connections.

A recent structural MRI study in toddlers and preschoolers found that age-related increases in cortical myelination in TD participants were absent in children with ASD, indicating that myelin trajectory may follow a different timeline in those with ASD (Chen et al., 2022). A significant association was not present between cortical myelin and autism symptoms when analyzing results from ASD participants, aligning with our observations that myelination may not heavily contribute to ASD behaviors as development continues. The disruption of myelination in young children found by this study may reflect that the microstructural differences underlying ASD are not constant until the brain is further developed, explaining the changes in symptom severity that can be present as a child with ASD gets older.

Interestingly, these microstructural and behavioral patterns uncovered by this study do not respect the traditional boundaries of an ASD diagnosis. Instead, they highlight the need to study autism as a spectrum where overlapping features and dimensions provide insights into individual variability. By focusing on the relationships between brain features and behavioral subtypes, research can move toward identifying meaningful subgroups within the spectrum. This approach may provide valuable insights into how unique neurobiological profiles underlie specific behavioral patterns, enabling the development of more targeted interventions. Ultimately, this framework shifts the focus from strict diagnostic categories to understanding ASD as a multidimensional condition defined by shared and distinct traits across individuals.

While other MRI studies have found associations between decreased axonal growth and autism, this study correlates these microstructural differences to behavioral severity. It evaluates a wide range of commonly administered ASD evaluations. As the symptomatic behaviors of ASD can span a wide range, determining differences within ASD subgroups is crucial to understanding the neurobiological factors driving these diverse behaviors. Further research is needed to track the relationships between microstructural metrics and behavioral subscales and investigate whether the associations between g-ratio and behavior remain as the individuals in this study continue to develop. In particular, observing the changes in these associations as participants reach and pass pubescent markers will provide evidence that microstructural metrics remain abnormal in the long term. A longitudinal framework will also be crucial to elucidate sex differences in ASD behaviors observed in score distributions. Insight into how microstructural metrics are related to behavior across the lifespan has the potential to serve as the basis of a biomarker of ASD and provide a neurobiological understanding of the disorder. Moreover, having a neurological explanation of behavioral severity in ASD can help inform the effect of psychological treatment and identify individuals at high risk of ASD before behavioral tests in infancy and early childhood can evaluate them.

## Supplementary Material

Supplementary Material

## Data Availability

The data underlying the results presented in this study are available currently through the NIMH Data Archive (NDA): https://nda.nih.gov/edit_collection.html?id=2021. Code is available at https://github.com/btn6sb/Conduction_Velocity
