# Supplementary material for: Widespread associations between behavioral metrics and brain microstructure in ASD suggest age mediates subtypes of ASD
Source: Imaging Neurosci (Camb). 2025 Sep 10;3:IMAG.a.144. doi: 10.1162/IMAG.a.144 (PMC12423640; doi:10.1162/IMAG.a.144)
Supplement: Supplementary Material [file IMAG.a.144_supp.zip › Supplementary Legends_v3.docx]

**Supplementary Figure Legends:**

**Supplementary Table 1:** Table of Pearson Correlation Coefficients, p-values, and adjusted p-values corrected for multiple comparisons using the Benjamini & Hochberg (1995) method, for each pairwise behavioral and cognitive metric collected in this study. For description of the behavioral metrics refer to Table 1.

**Supplementary Table 2:** Table of linear model results from both including all participants and only on the ASD participants with number of ROIs significantly associated with each behavioral metric following Benjamini & Hochberg correction as well as the average slope of that association.

**Supplementary Table 3:** Table of all linear model slopes and Benjamini & Hochberg adjusted p-values for each behavioral assessment for aggregate g-ratio in each ROI in all participants in the cohort including non-ASD.

**Supplementary Table 4:** Table of all linear model slopes and Benjamini & Hochberg adjusted p-values for each behavioral assessment for aggregate conduction velocity in each ROI in all participants in the cohort including non-ASD.

**Supplementary Table 5:** Table of all linear model slopes and Benjamini & Hochberg adjusted p-values for each behavioral assessment for aggregate g-ratio in each ROI in only ASD participants.

**Supplementary Table 6:** Table of all linear model slopes and Benjamini & Hochberg adjusted p-values for each behavioral assessment for aggregate conduction velocity in only ASD participants.

**Supplementary Figure 1:** Values from all neuroimaging data by ROI projected onto the CHOIR clusters. Each dot represents a single participant and the color scale represents greater or lower aggregate g-ratio or aggregate conduction velocity. This neuroimaging data was used by the hierarchical clustering algorithm to generate the clusters, with each dot representing a participant. Each value from each participant’s ROI is presented individually on the final CHOIR cluster as in Figure 5 and is presented here for reference. ROIs with stronger correspondence to the existing clusters are assumed to more heavily influence the algorithms clustering decision, wereas ROIs without good correspondence likely did not differentiate between subjects well.

**Supplementary Figure 2:** Values from all behavioral and cognitive metrics projected onto the CHOIR clusters from Figures 5 & 6. With each dot representing a single subject, the behavioral or cognitive metrics can be displayed on a per-subject basis. These behavioral and cognitive metrics were not available to the clustering algorithm and patterns thus originated from microstructural brain markers. This list is presented as a reference for all the behavioral and cognitive metrics used in the study. Metrics that correspond to the clusters can be assumed to arise from microstructural processes. Conversely, metrics that do not correspond to clusters or microstructural gradients would be assumed to not have strong connections to microstructural processes used in this study.

**Supplementary Figure 3:** Values from all behavioral and cognitive and neuroimaging metrics projected onto the ASD only CHOIR clustering analysis. All of these metrics were used to generate the clusters in the bottom row of Figure 7. All of the individual metrics are displayed on the participant they are associated with identically to supplementary figures 1 & 2. Color scales and all metrics have been rescaled prior to clustering. Association between metrics and clusters in this analysis are indicative of a metric that was highly discriminative between individuals and was utilized by the cluster algorithm for sorting. All metrics presented were used in the clustering process.
