# Supplementary material for: Widespread associations between behavioral metrics and brain microstructure in ASD suggest age mediates subtypes of ASD
Source: Imaging Neurosci (Camb). 2025 Sep 10;3:IMAG.a.144. doi: 10.1162/IMAG.a.144 (PMC12423640; doi:10.1162/IMAG.a.144)
Supplement: Supplementary Material [file IMAG.a.144_supp.zip › Supplementary_Figure_S1.pdf]

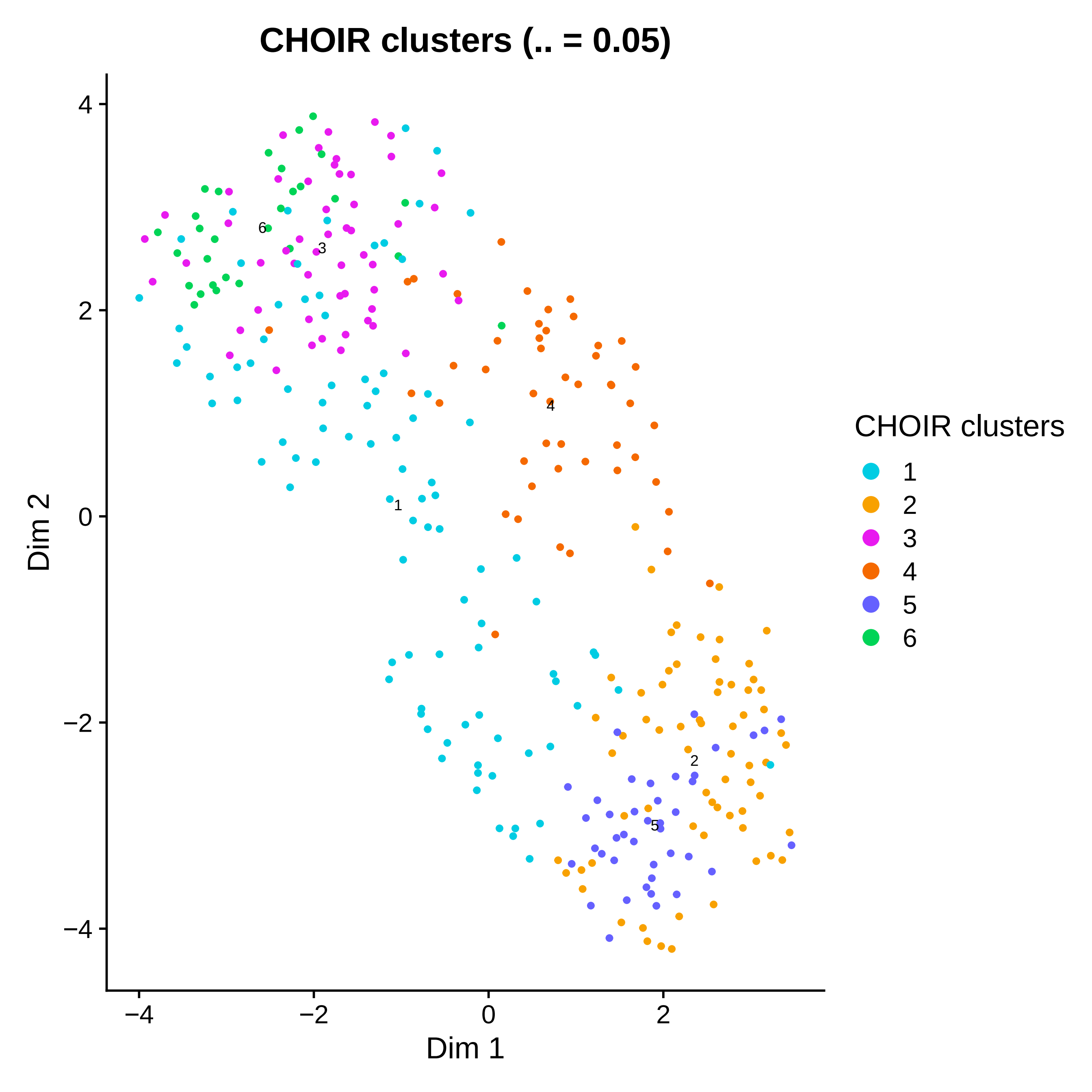
CHOIR clusters (.. = 0.05)
Dim 2
Dim 1
4
2
0
−2
−4
−4
−2
0
2
6
3
1
4
2
5
CHOIR clusters
1
2
3
4
5
6

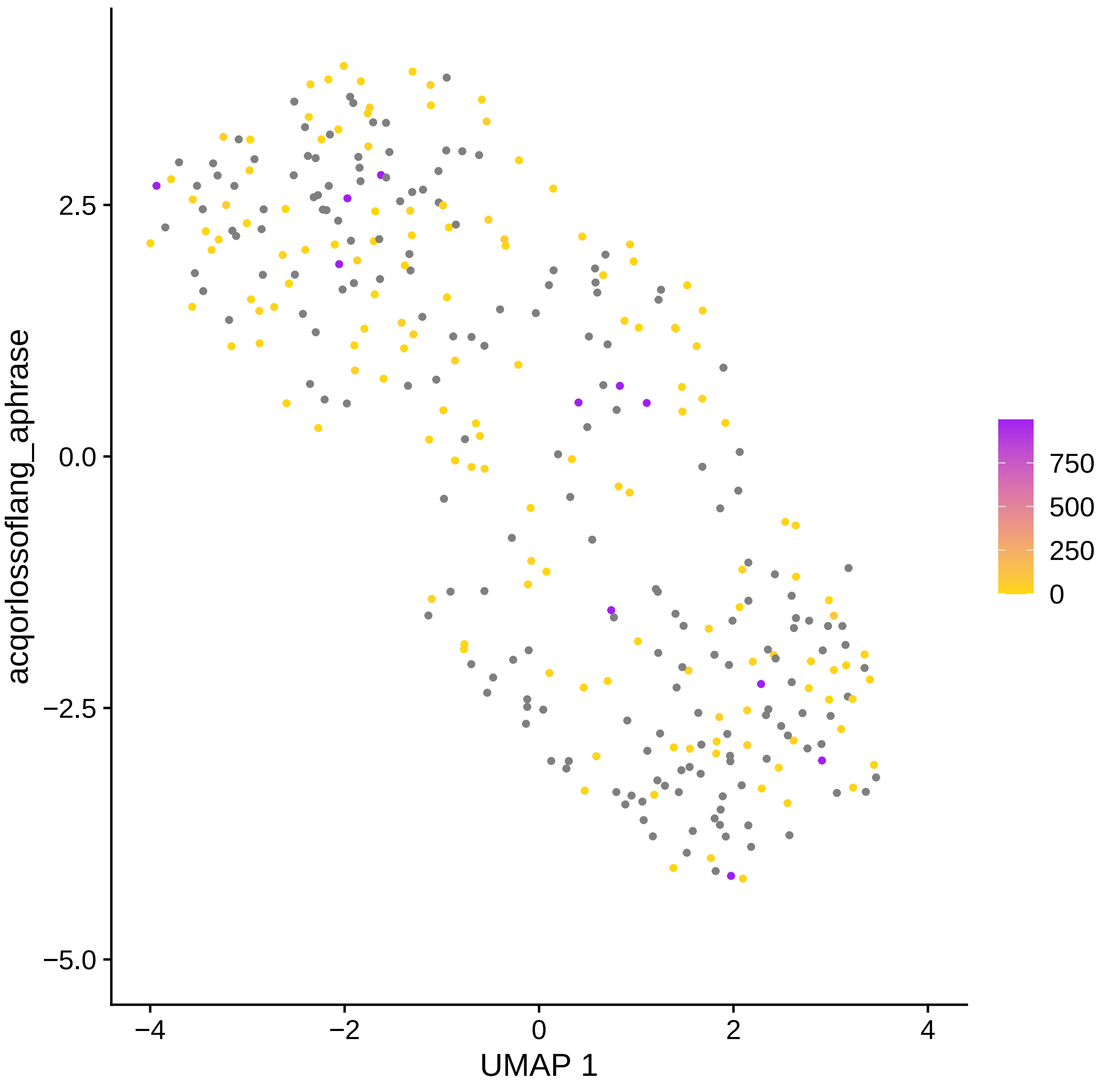
acqorlossoflang_aphrase
UMAP 1
2.5
0.0
−2.5
−5.0
−4
−2
0
2
4
750
500
250
0

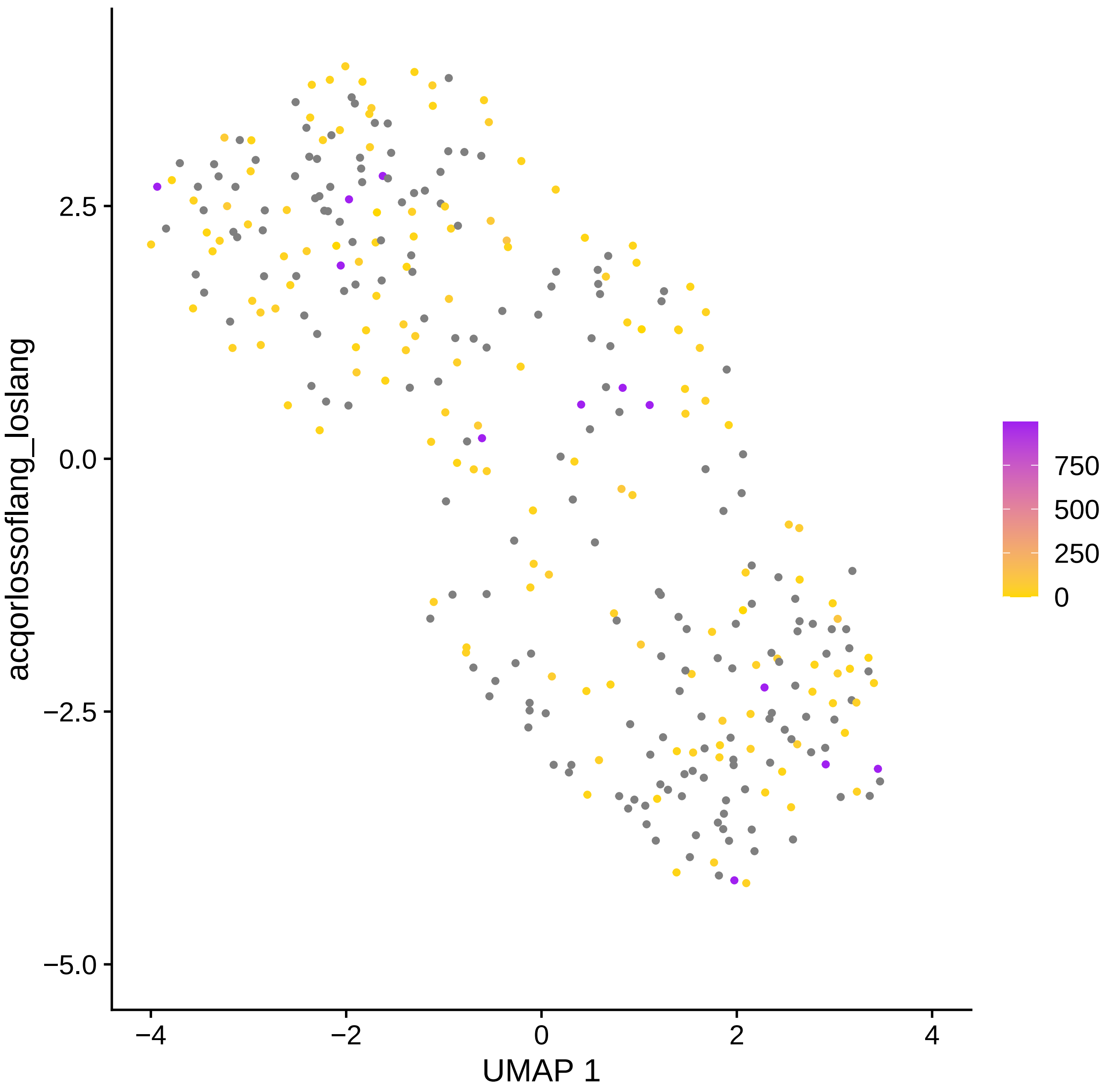
acqorlossoflang_loslang
UMAP 1
2.5
0.0
−2.5
−5.0
−4
−2
0
2
4
750
500
250
0

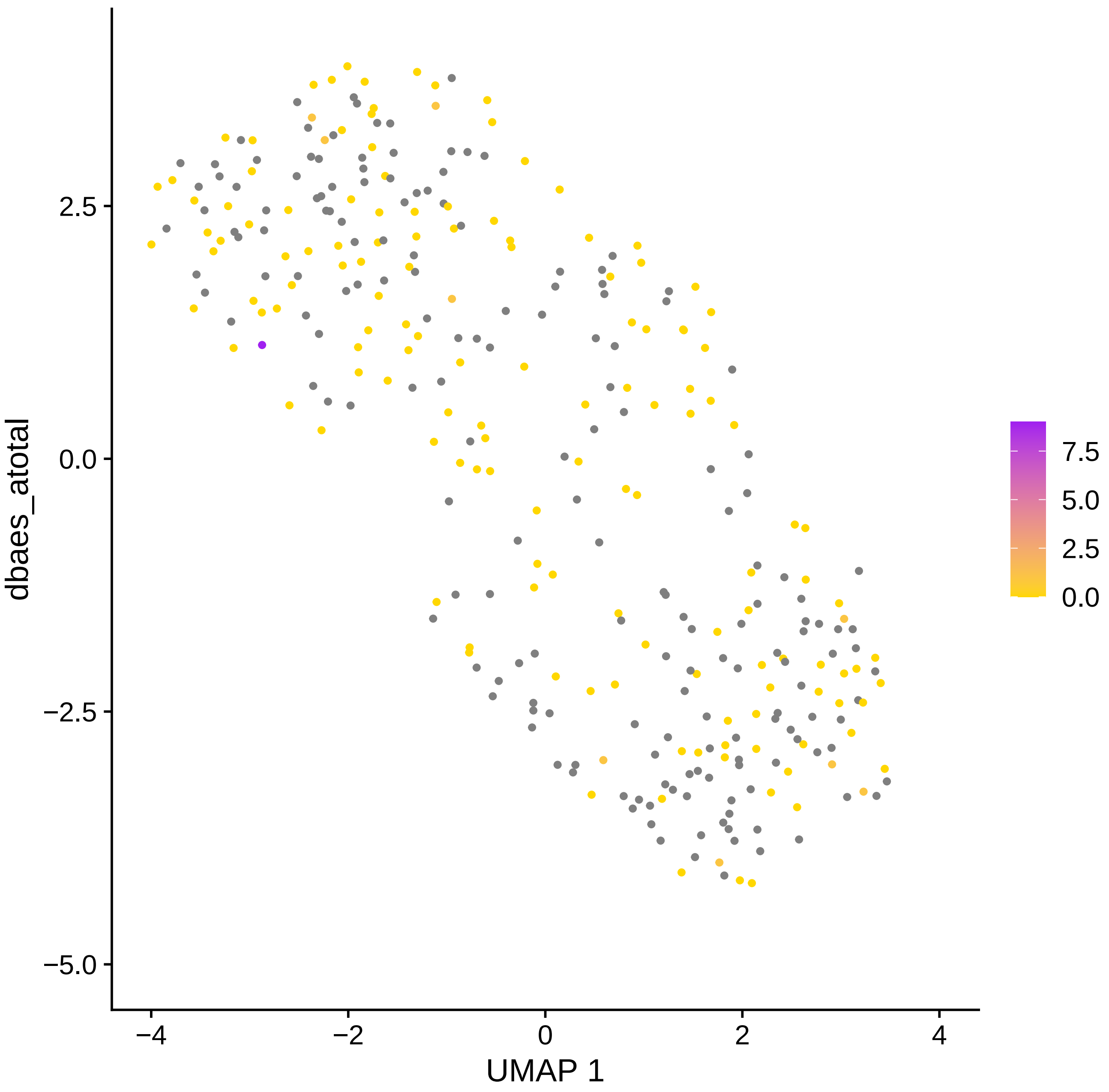
dbaes_atotal
2.5
0.0
−2.5
−5.0
−4
−2
0
2
4
UMAP 1
7.5
5.0
2.5
0.0

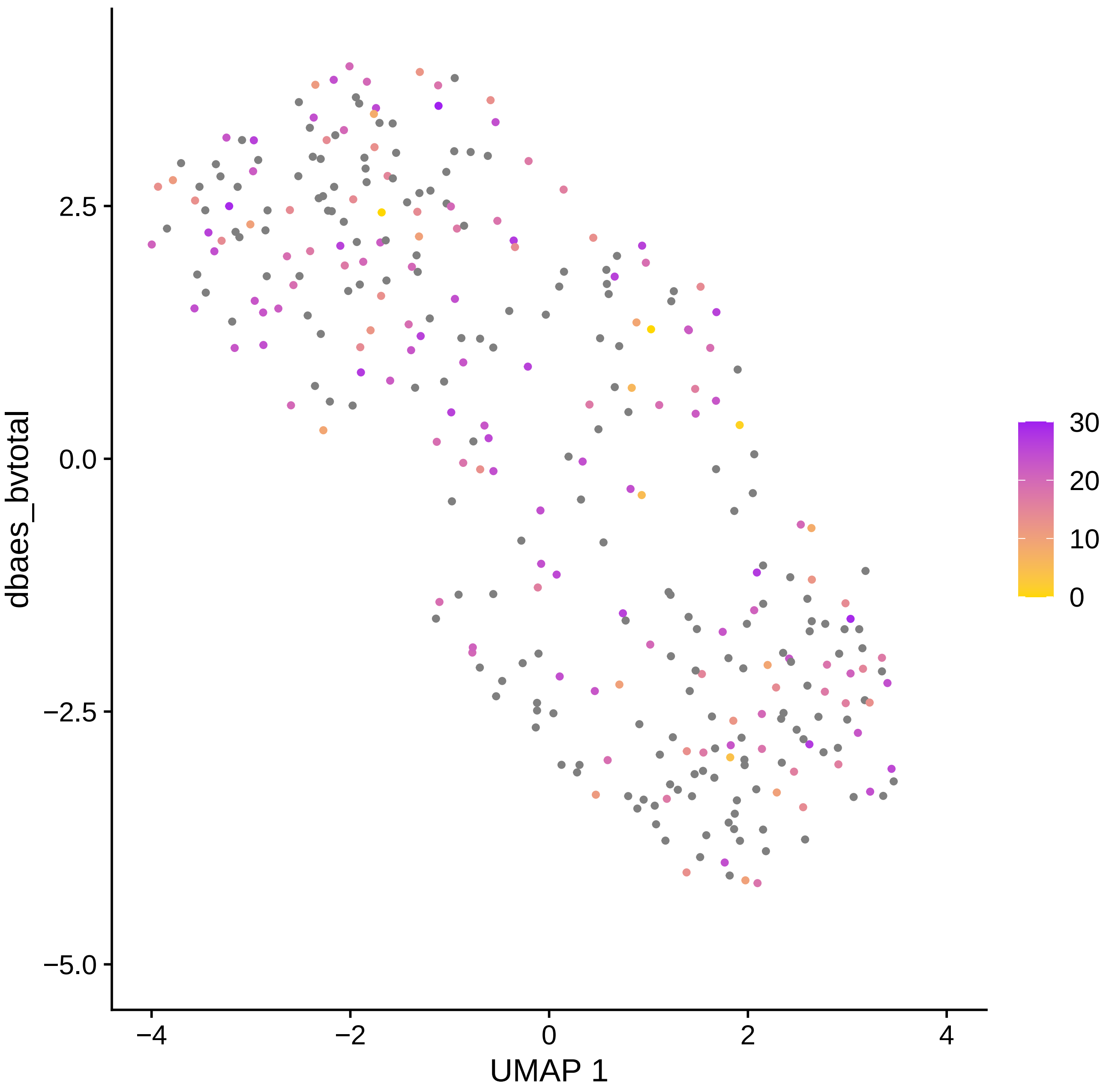
dbaes_bvtotal
UMAP 1
2.5
0.0
−2.5
−5.0
−4
−2
0
2
4
30
20
10
0

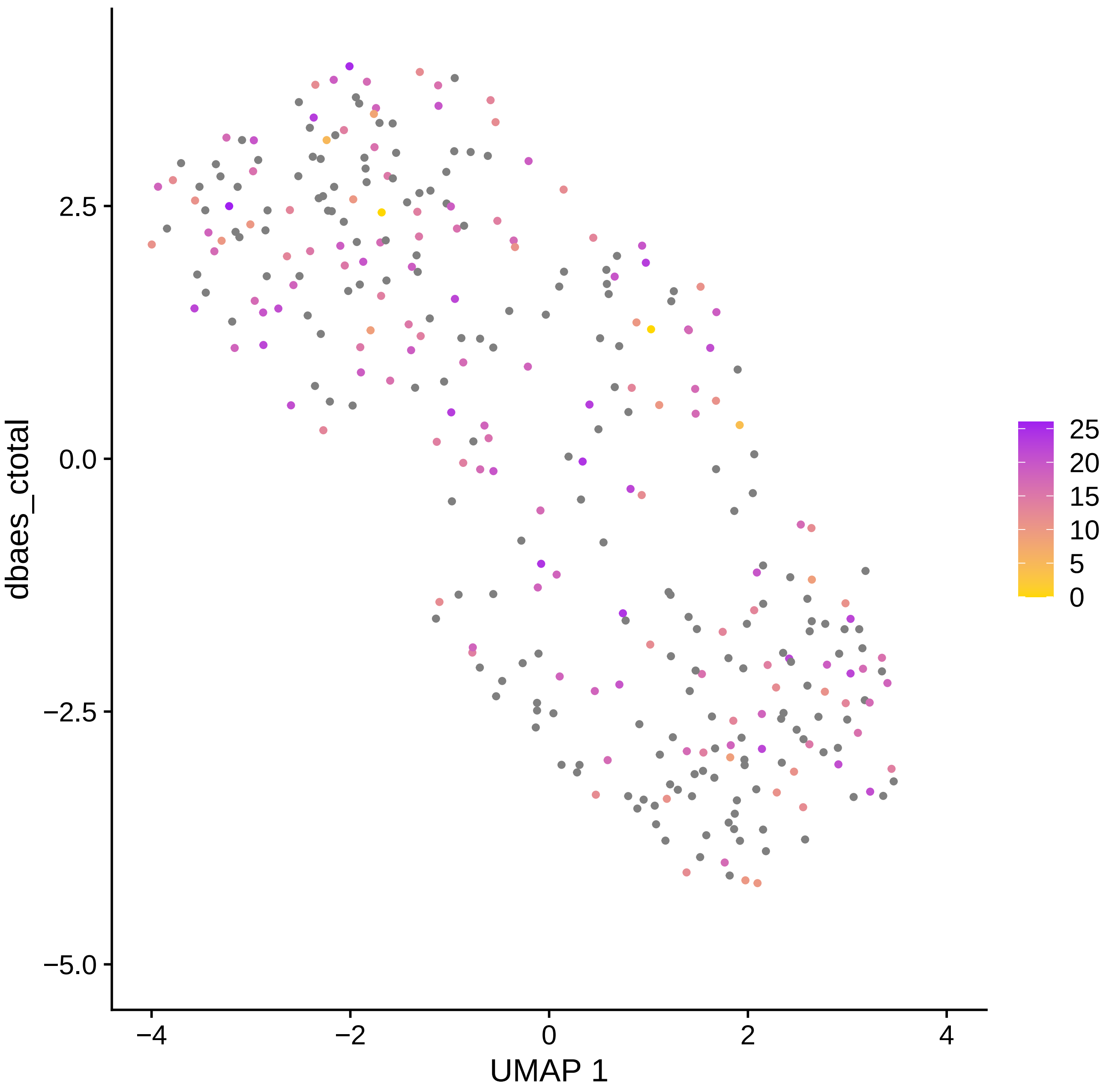

dbaes_ctotal
UMAP 1
2.5
0.0
−2.5
−5.0
−4
−2
0
2
4
25
20
15
10
5
0

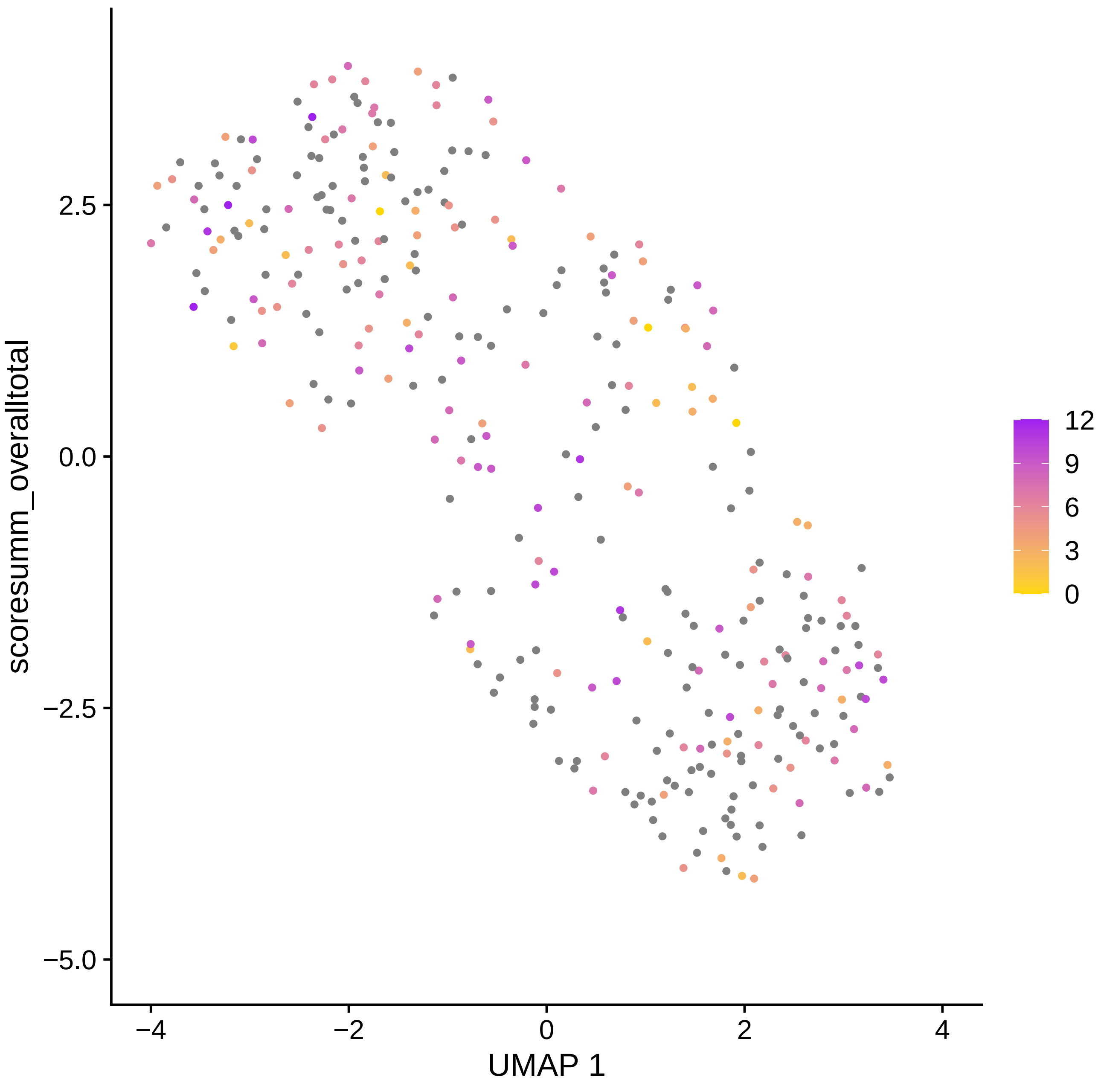

scoresumm_overalltotal
UMAP 1
2.5
0.0
−2.5
−5.0
−4
−2
0
2
4
12
9
6
3
0

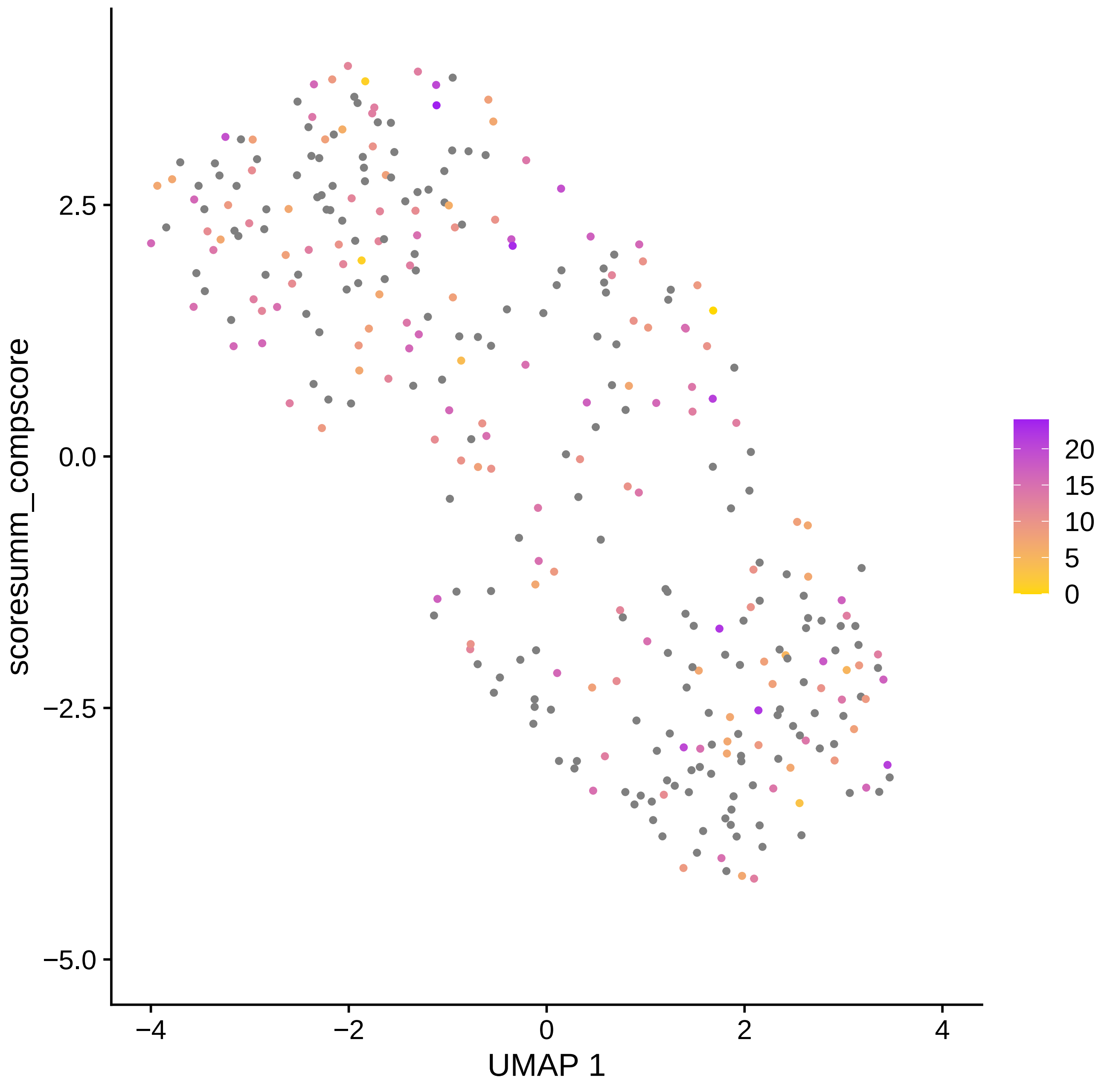

scoresumm_compscore
UMAP 1
2.5
0.0
−2.5
−5.0
−4
−2
0
2
4
20
15
10
5
0

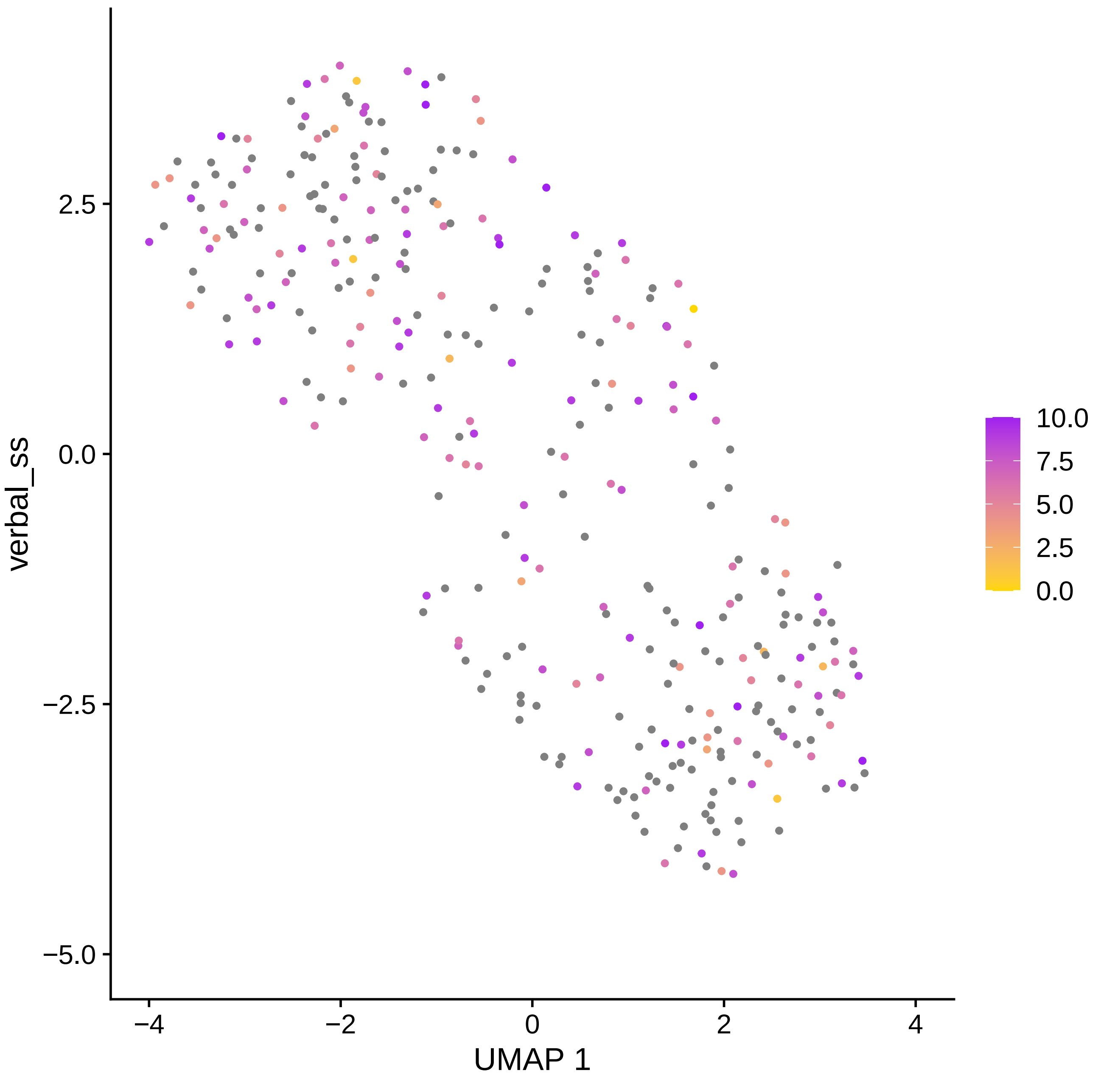
verbal_ss
2.5
0.0
−2.5
−5.0
−4
−2
0
2
4
UMAP 1
10.0
7.5
5.0
2.5
0.0

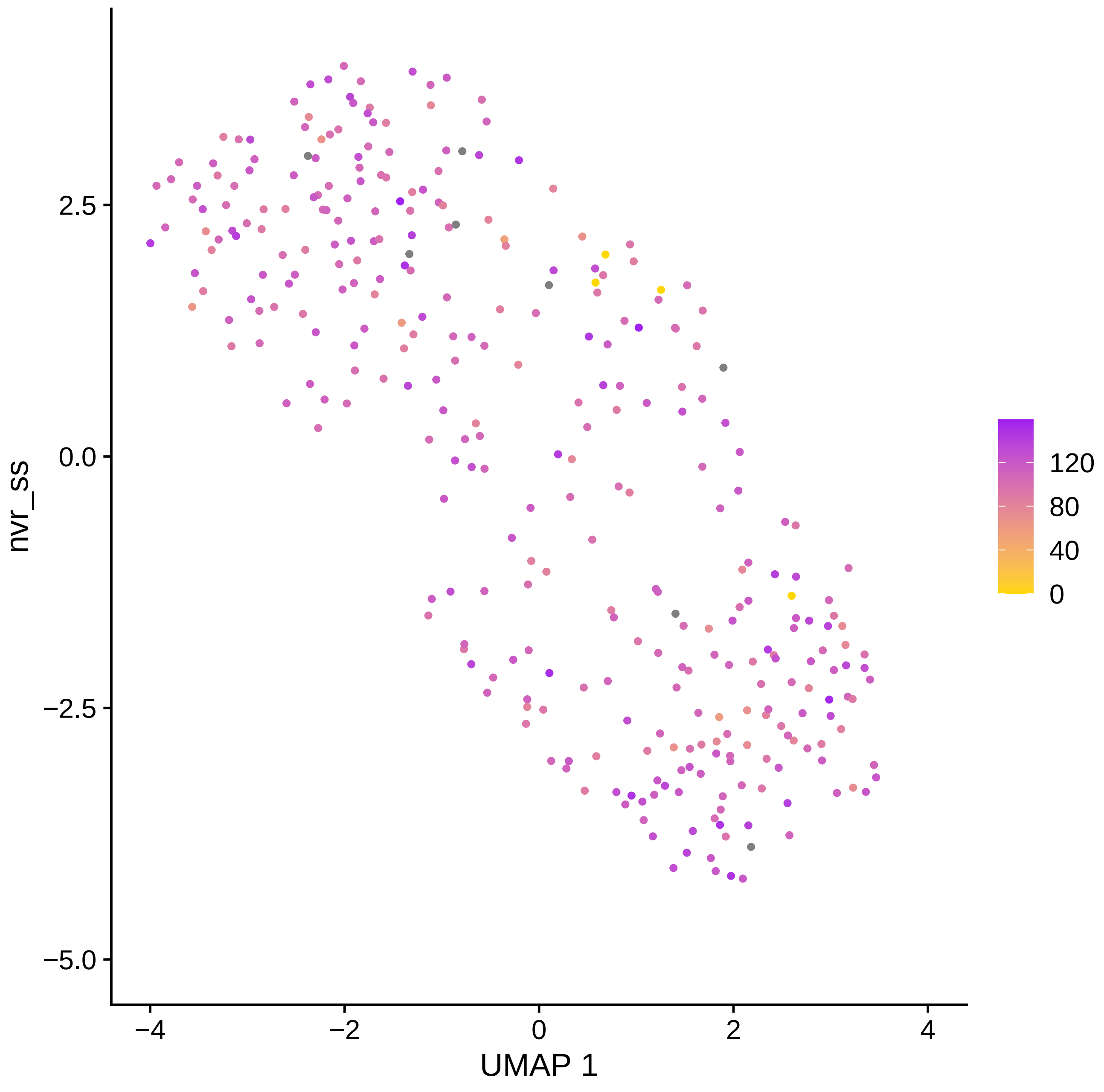

nvr_ss
2.5
0.0
−2.5
−5.0
−4
−2
0
2
4
UMAP 1
120
80
40
0

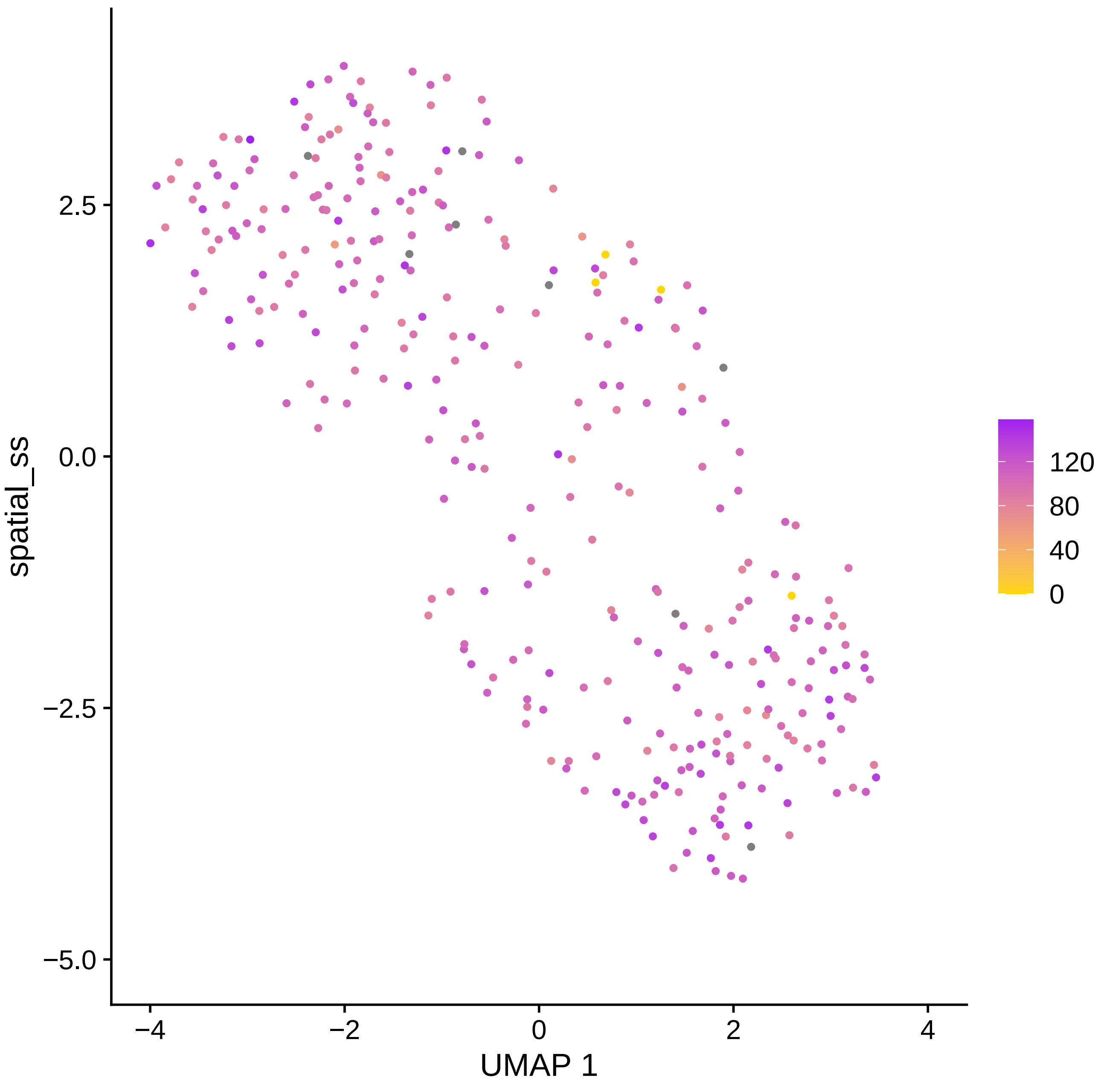
spatial_ss
UMAP 1
2.5
0.0
−2.5
−5.0
−4
−2
0
2
4
120
80
40
0

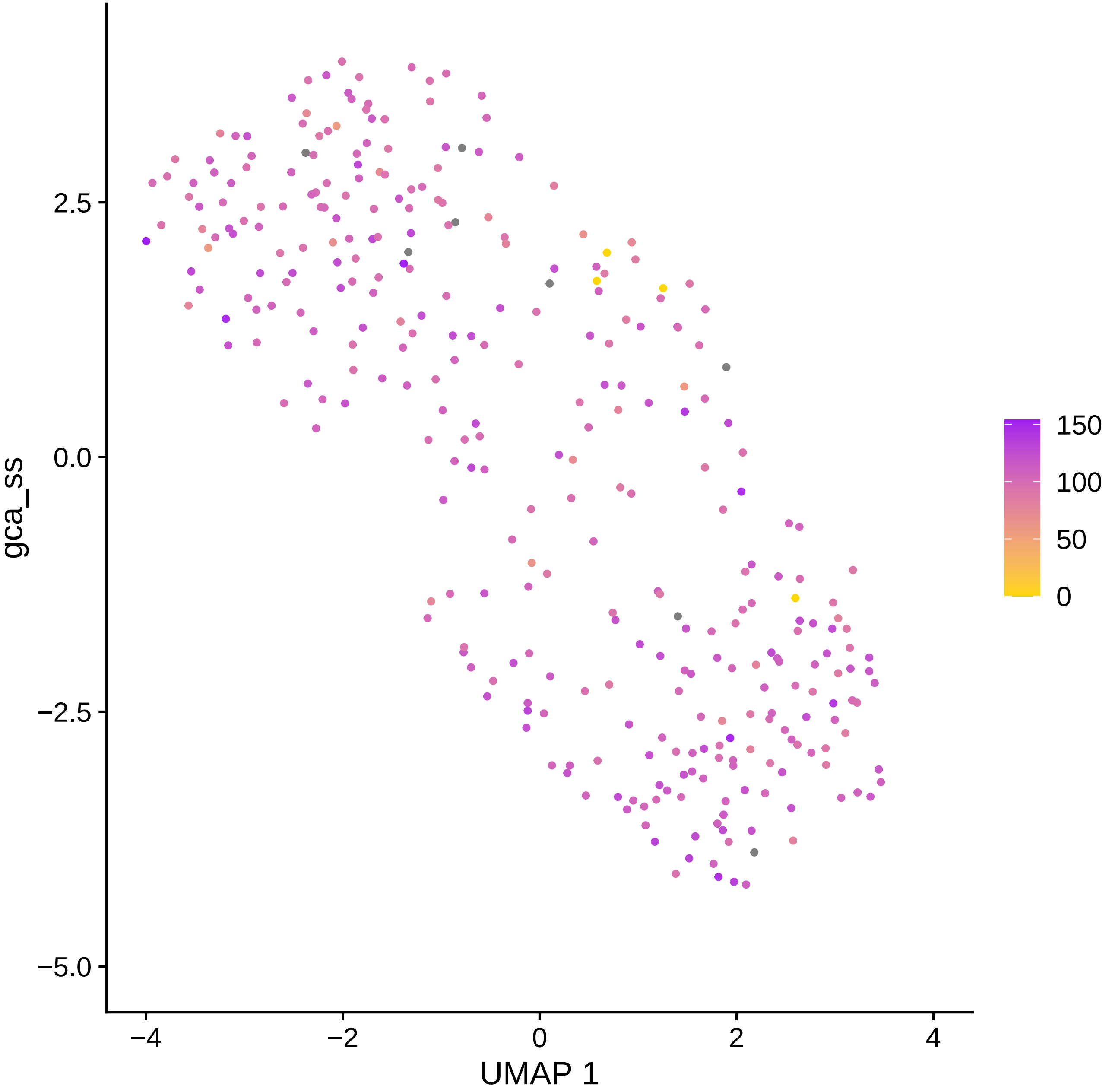
gca_ss
UMAP 1
2.5
0.0
−2.5
−5.0
−4
−2
0
2
4
150
100
50
0

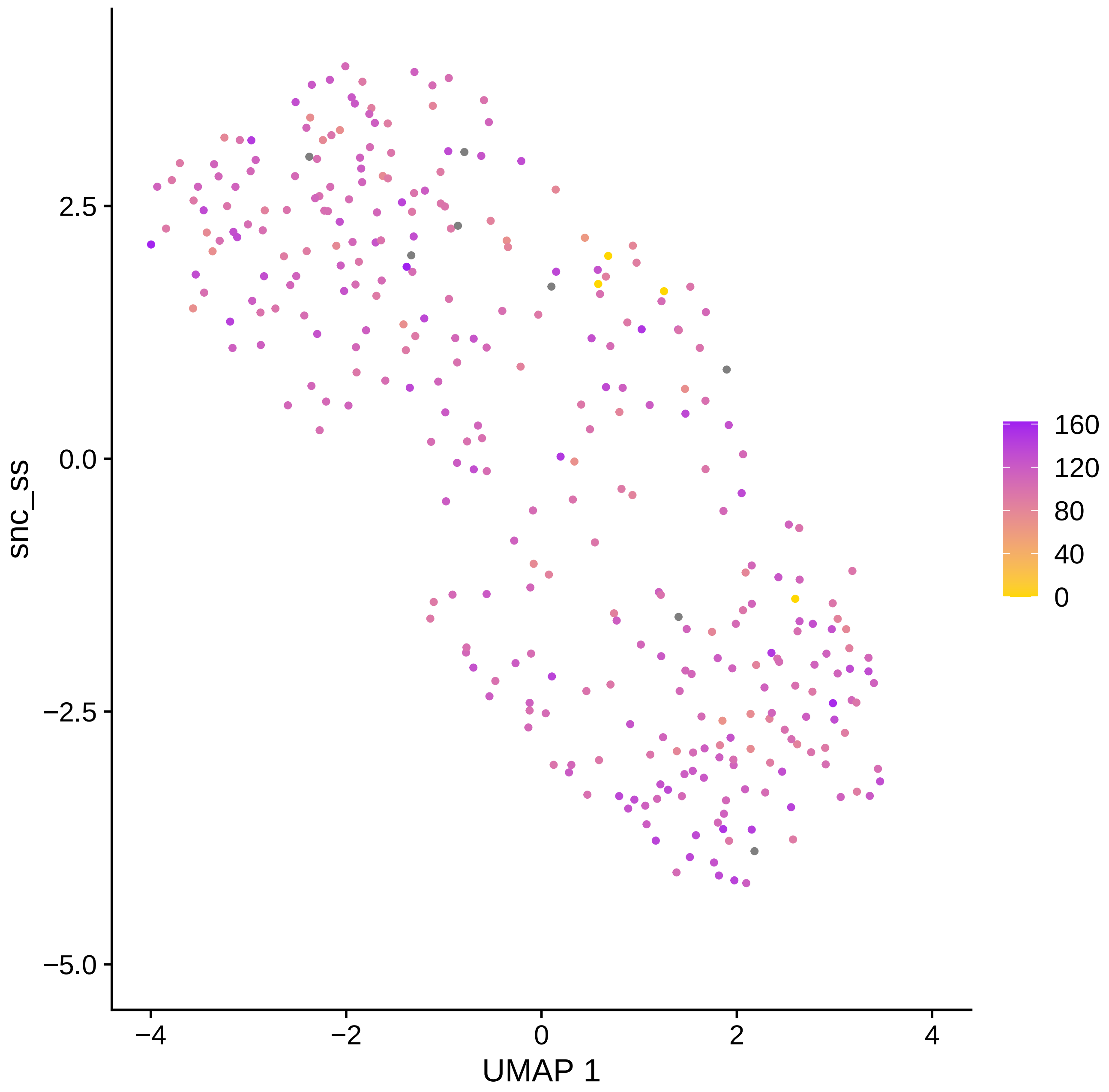

snc_ss
UMAP 1
2.5
0.0
−2.5
−5.0
−4
−2
0
2
4
160
120
80
40
0

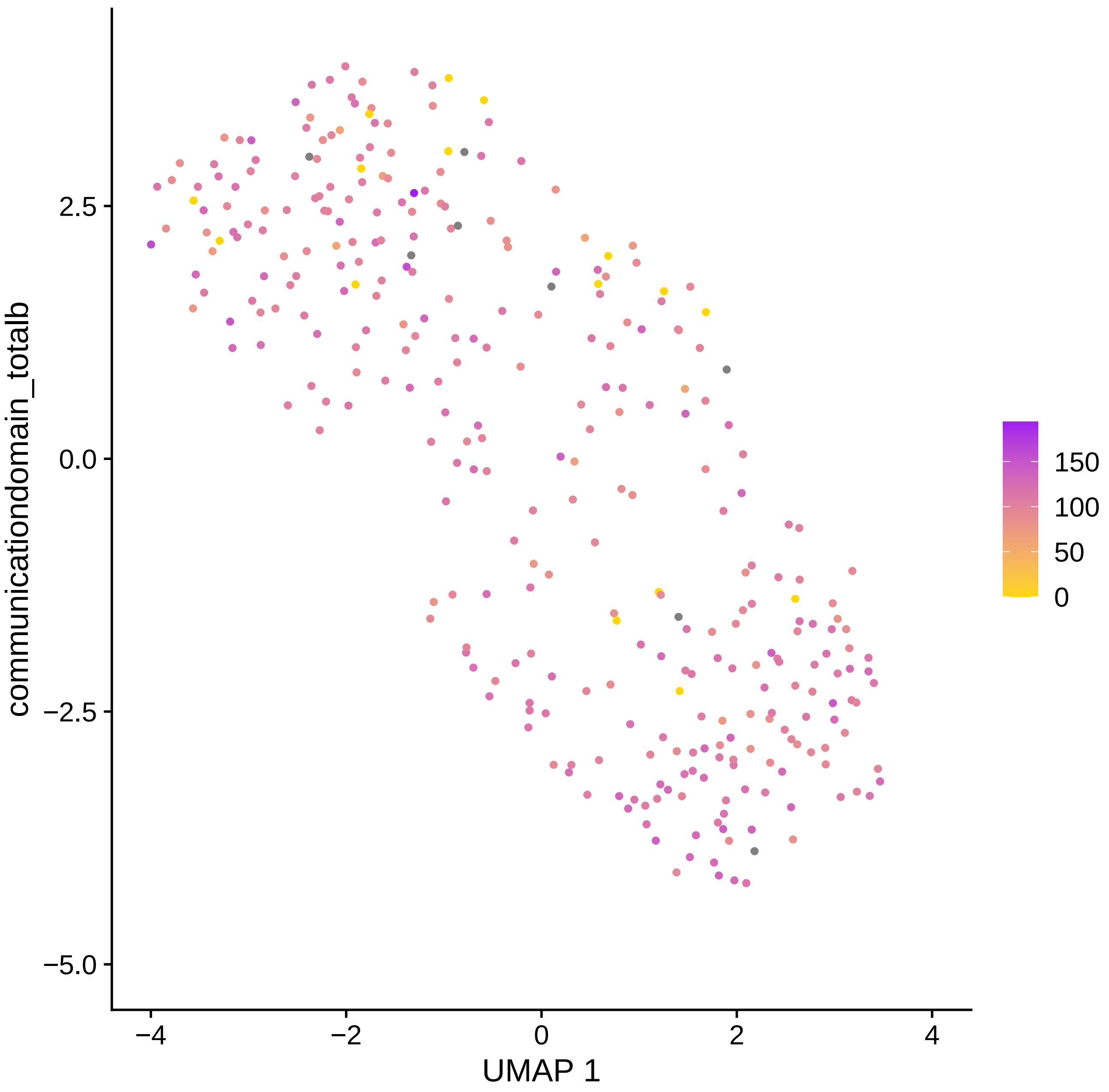

communicationdomain_totalb
UMAP 1
2.5
0.0
−2.5
−5.0
−4
−2
0
2
4
150
100
50
0

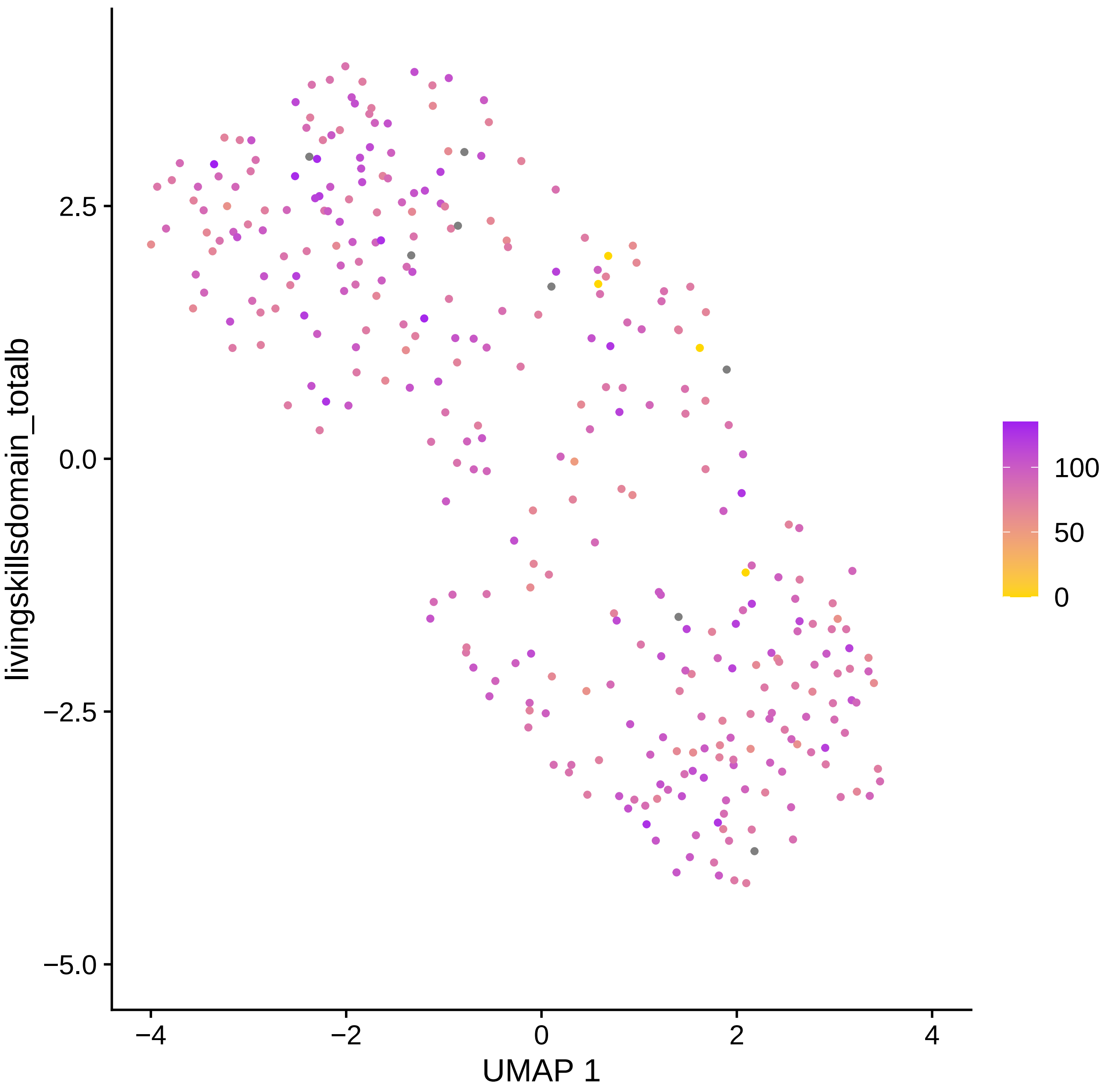

livingskillsdomain_totalb
UMAP 1
2.5
0.0
−2.5
−5.0
−4
−2
0
2
4
100
50
0

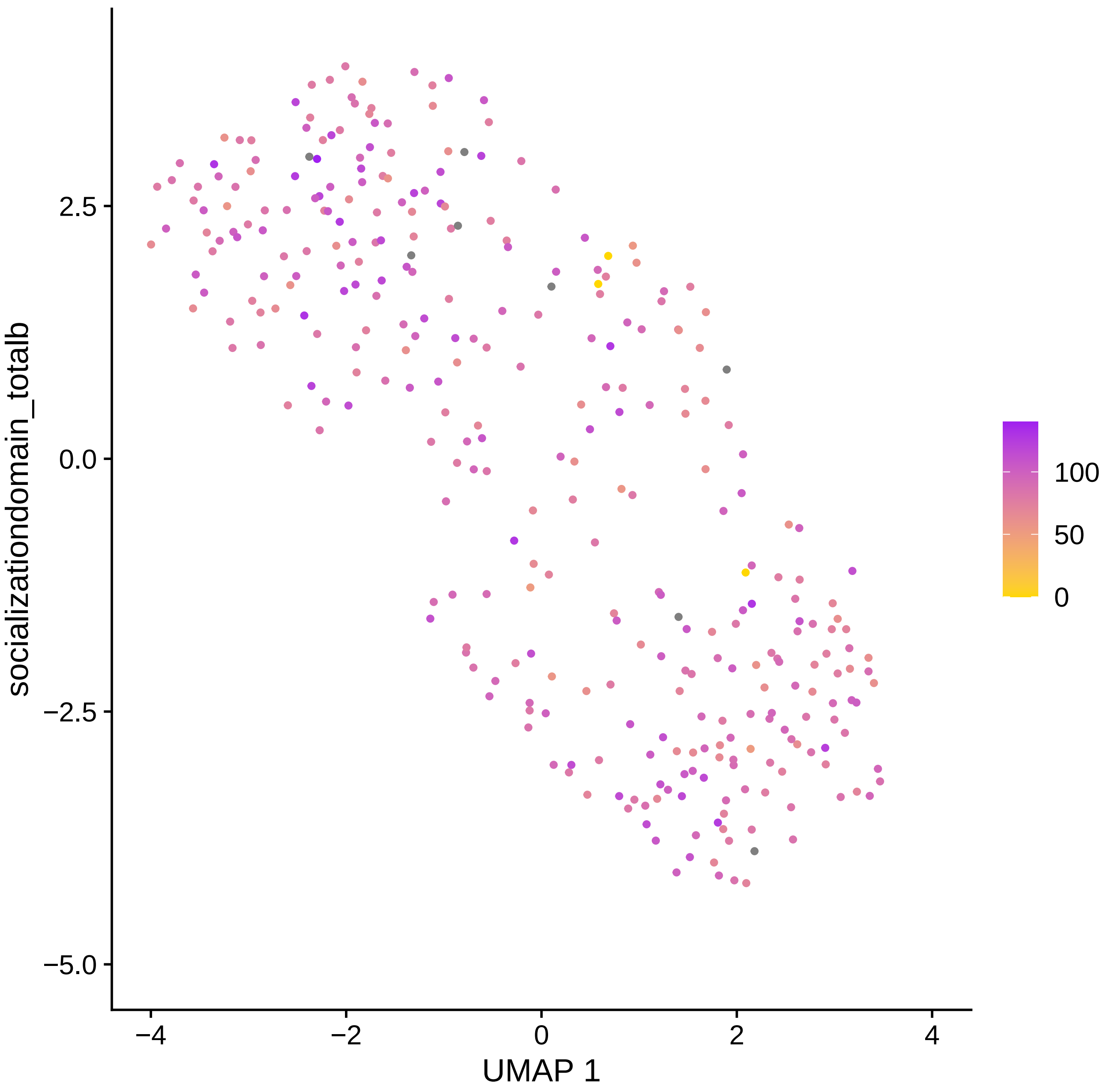
socializationdomain_totalb
UMAP 1
2.5
0.0
−2.5
−5.0
−4
−2
0
2
4
100
50
0

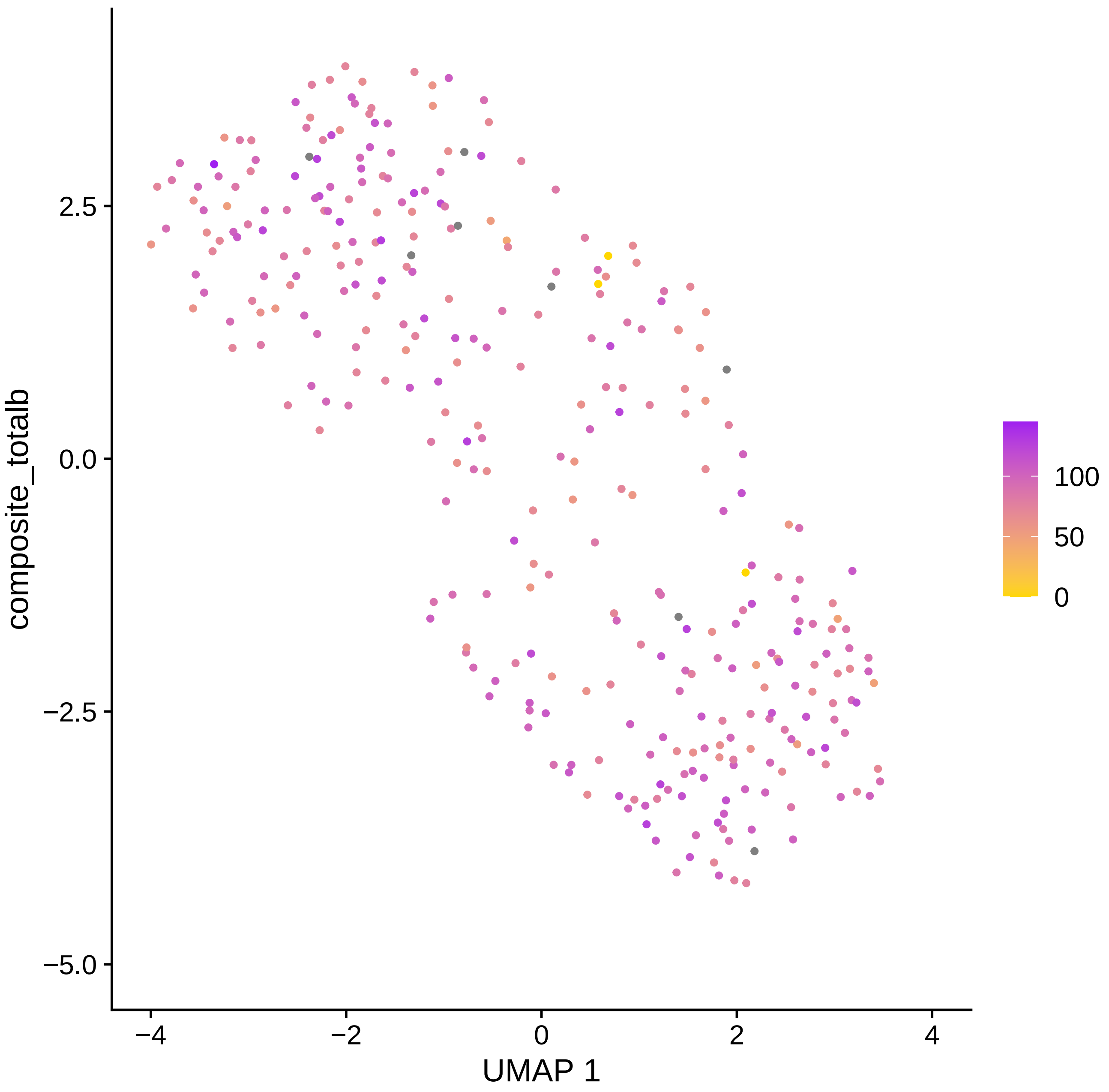
composite_totalb
2.5
0.0
−2.5
−5.0
−4
−2
0
2
4
UMAP 1
100
50
0

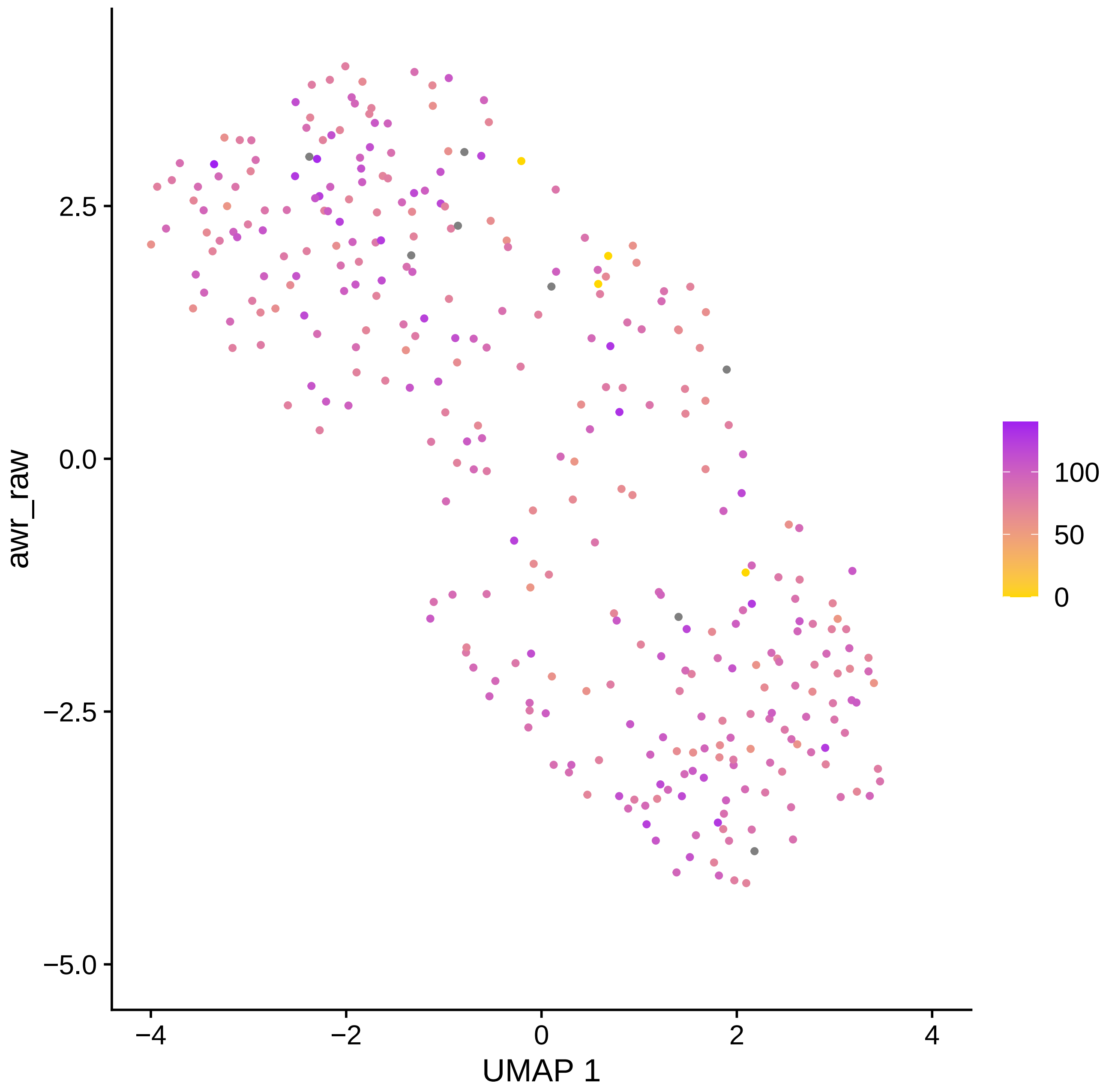

awr_raw
UMAP 1
2.5
0.0
−2.5
−5.0
−4
−2
0
2
4
100
50
0

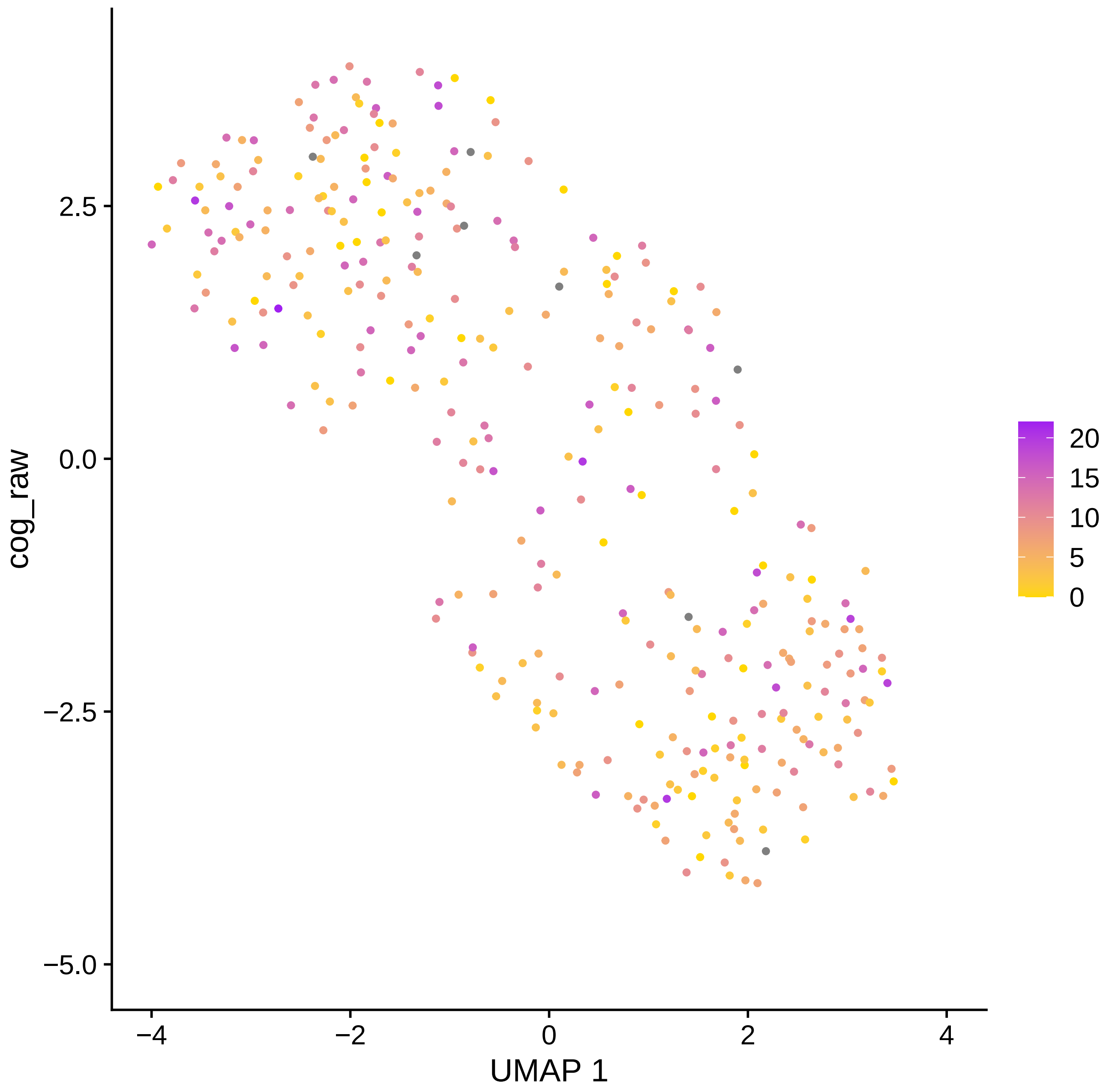
cog_raw
UMAP 1
2.5
0.0
−2.5
−5.0
−4
−2
0
2
4
20
15
10
5
0

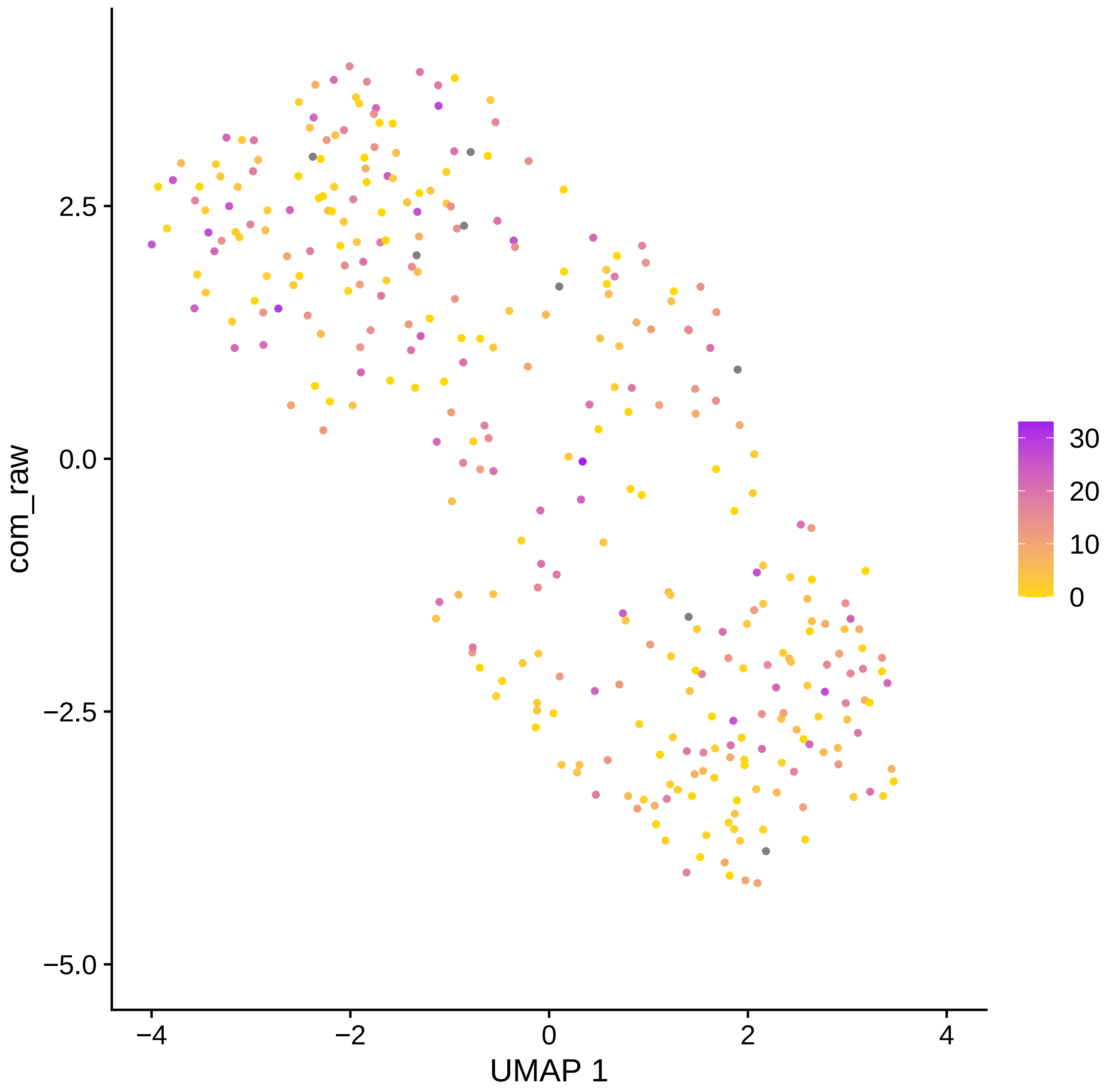

com_raw
UMAP 1
2.5
0.0
−2.5
−5.0
−4
−2
0
2
4
30
20
10
0

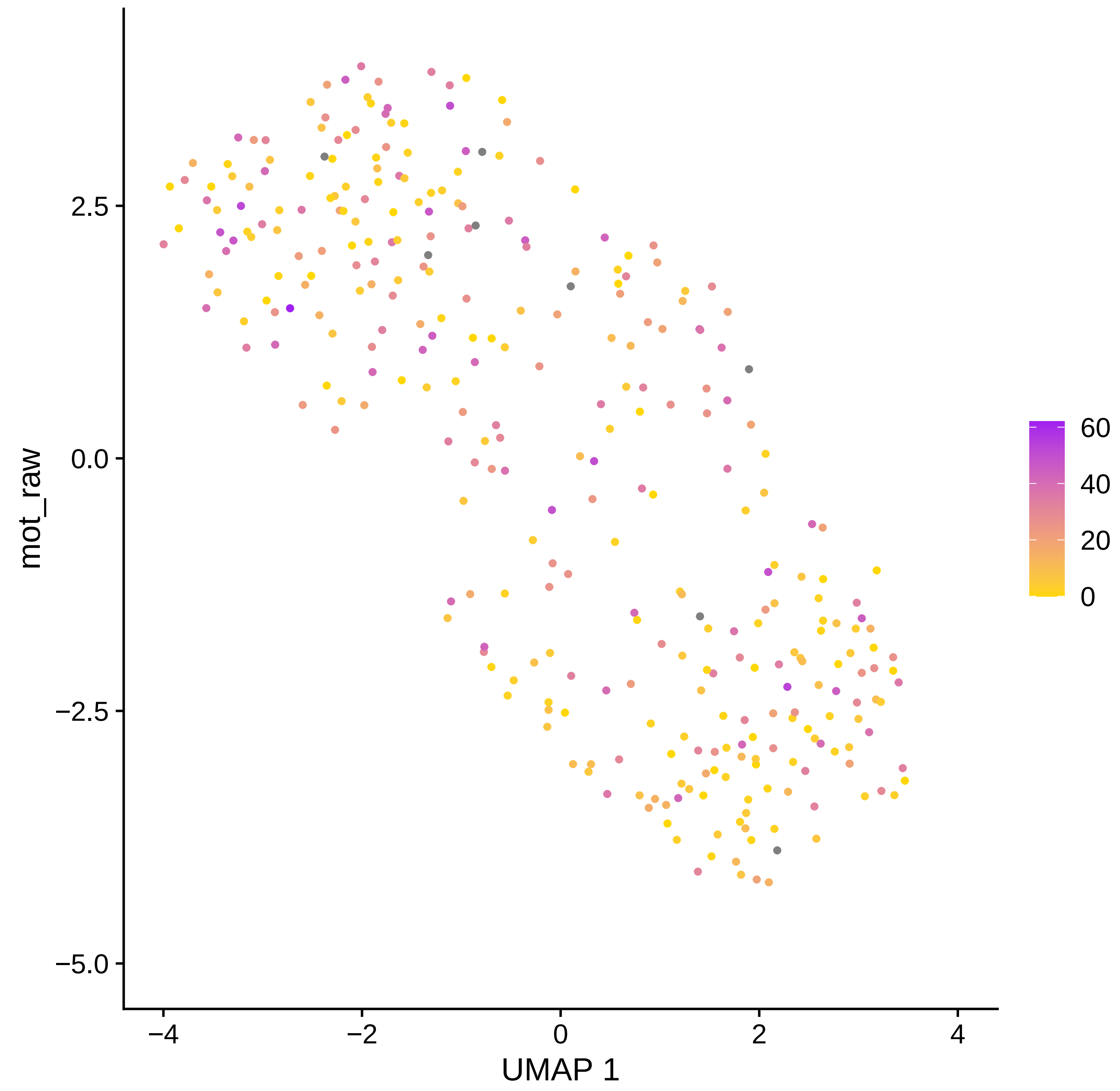

mot_raw
2.5
0.0
−2.5
−5.0
−4
−2
0
2
4
UMAP 1
60
40
20
0

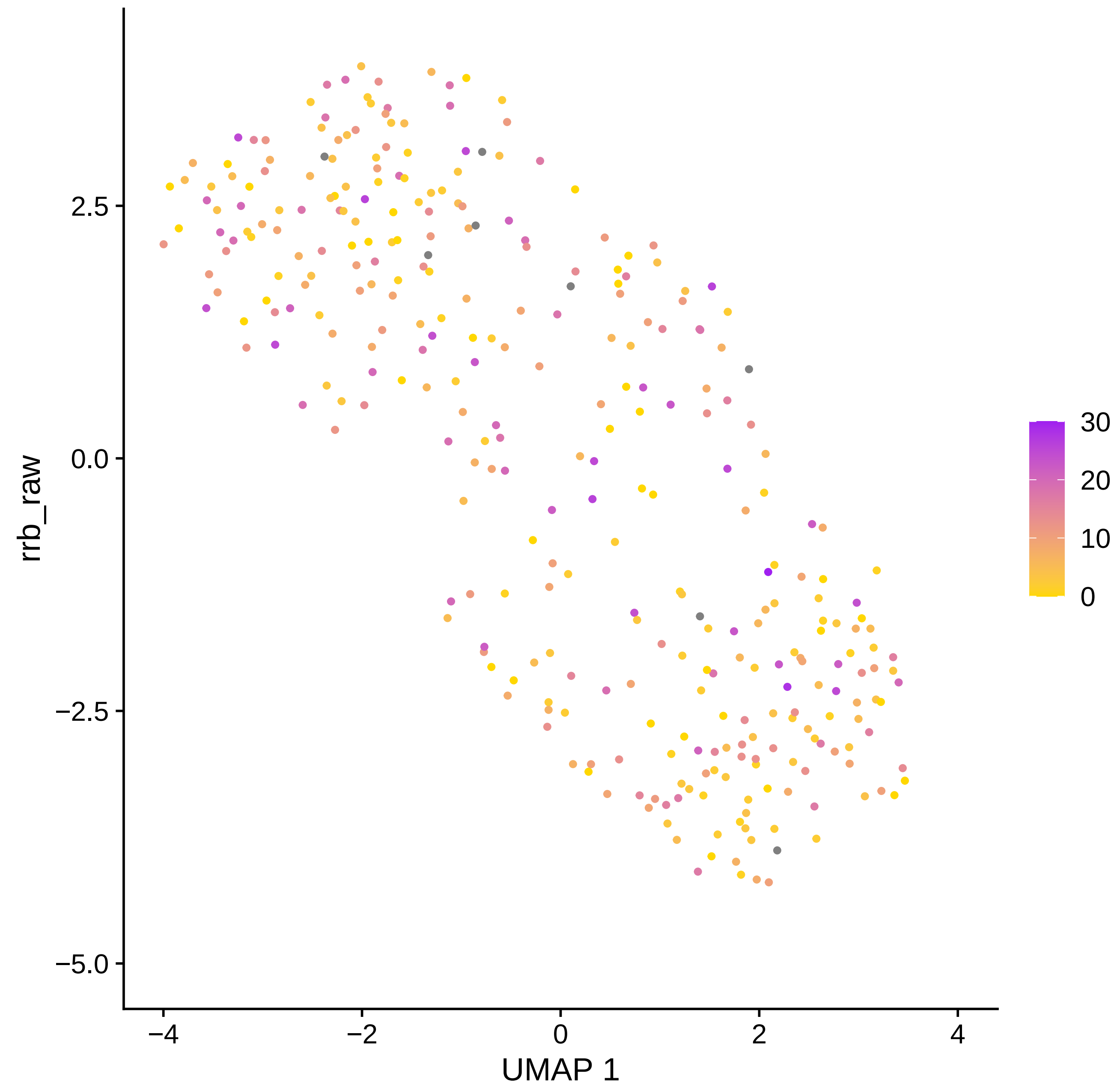

rrb_raw
UMAP 1
2.5
0.0
−2.5
−5.0
−4
−2
0
2
4
30
20
10
0

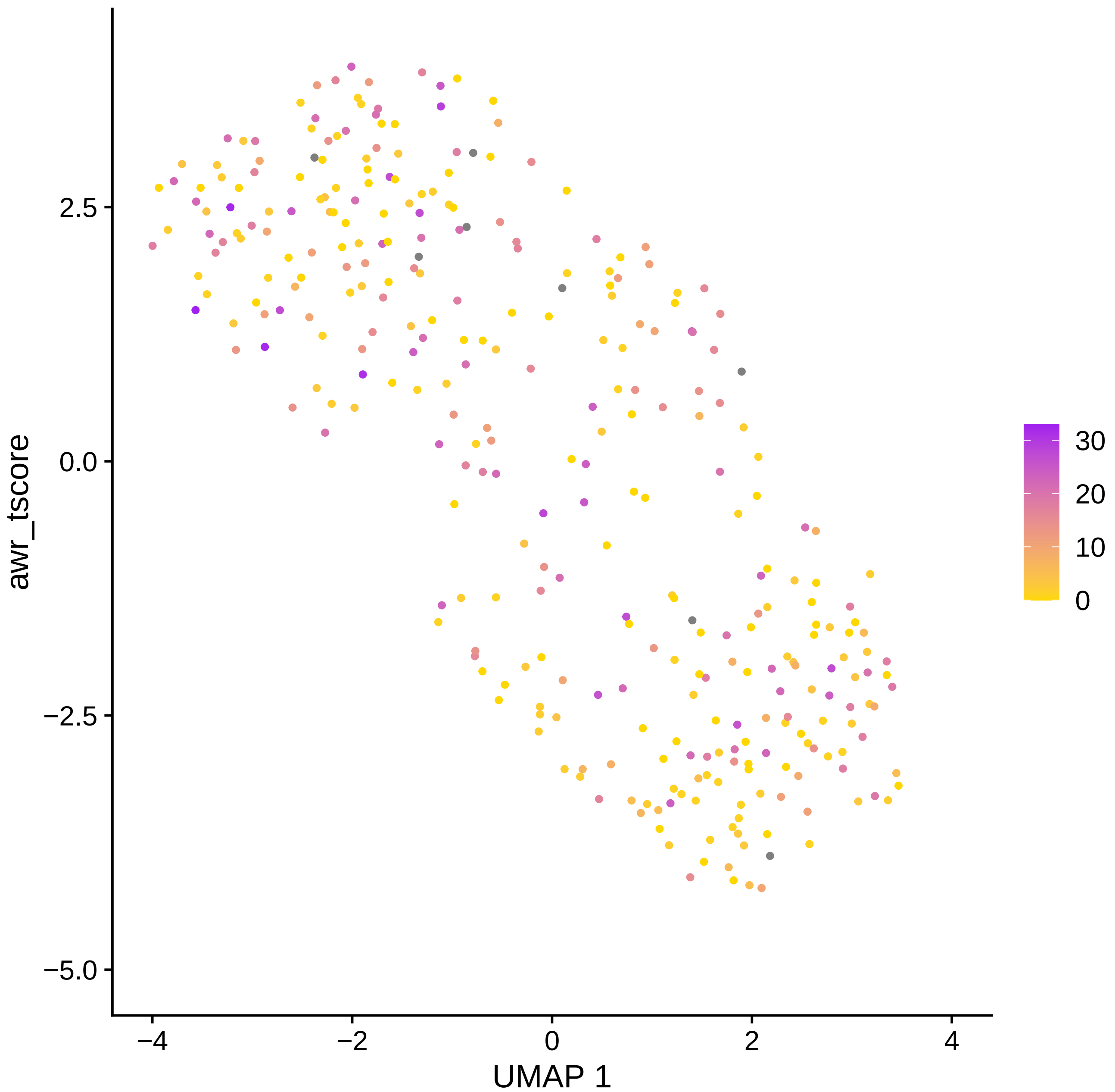

awr_tscore
UMAP 1
2.5
0.0
−2.5
−5.0
−4
−2
0
2
4
30
20
10
0

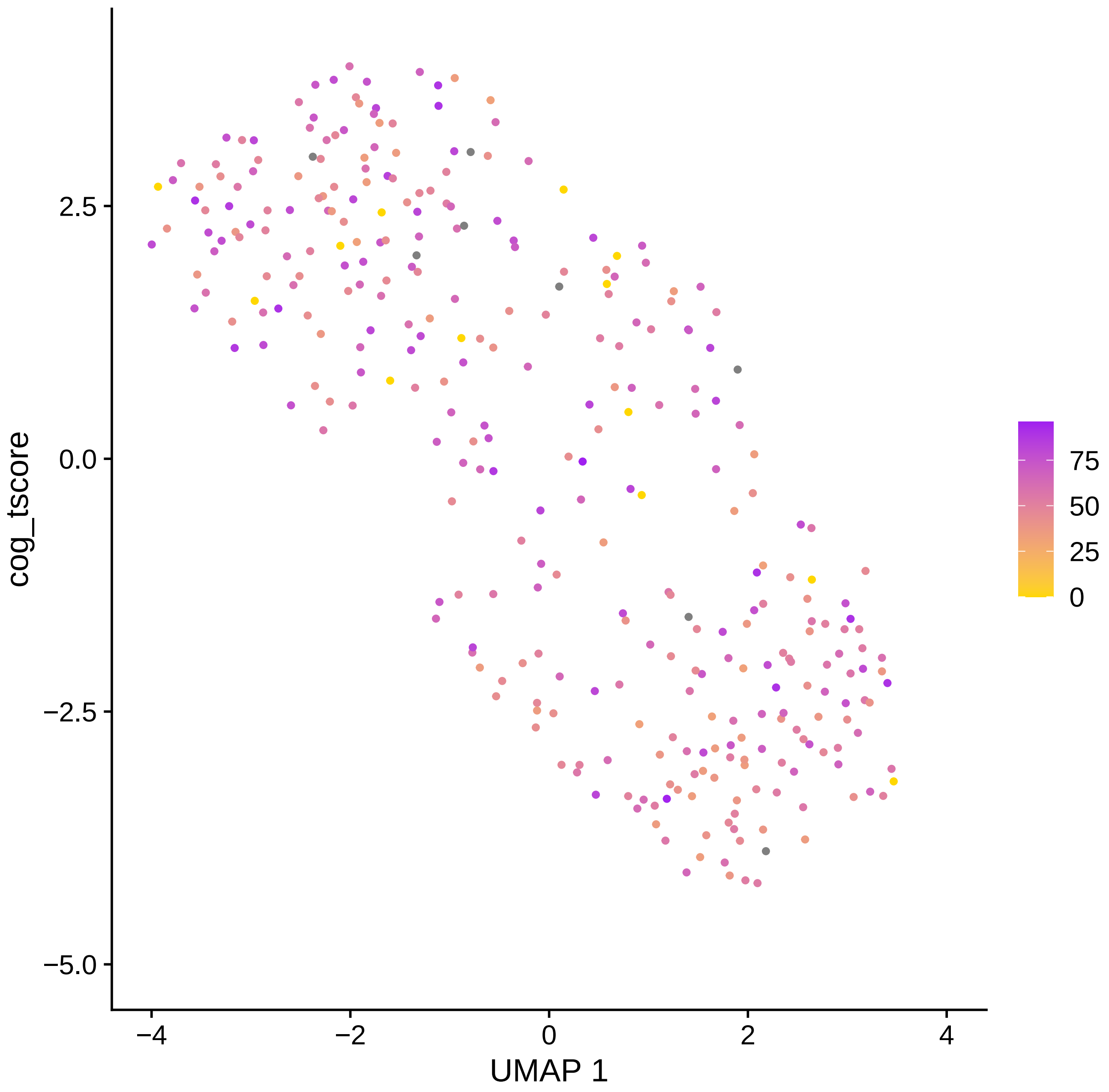
cog_tscore
2.5
0.0
−2.5
−5.0
−4
−2
0
2
4
UMAP 1
75
50
25
0

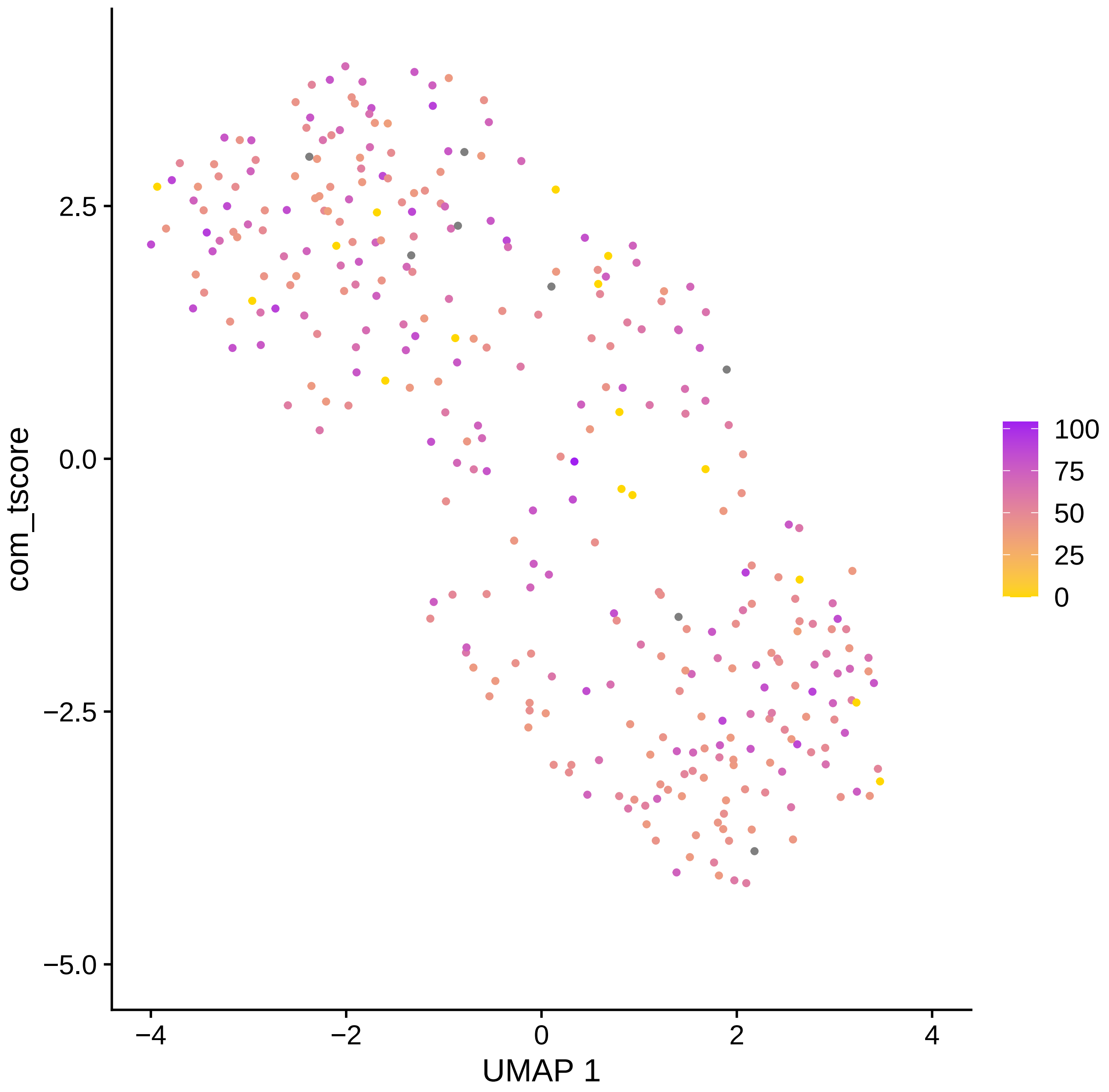
com_tscore
UMAP 1
2.5
0.0
−2.5
−5.0
−4
−2
0
2
4
100
75
50
25
0

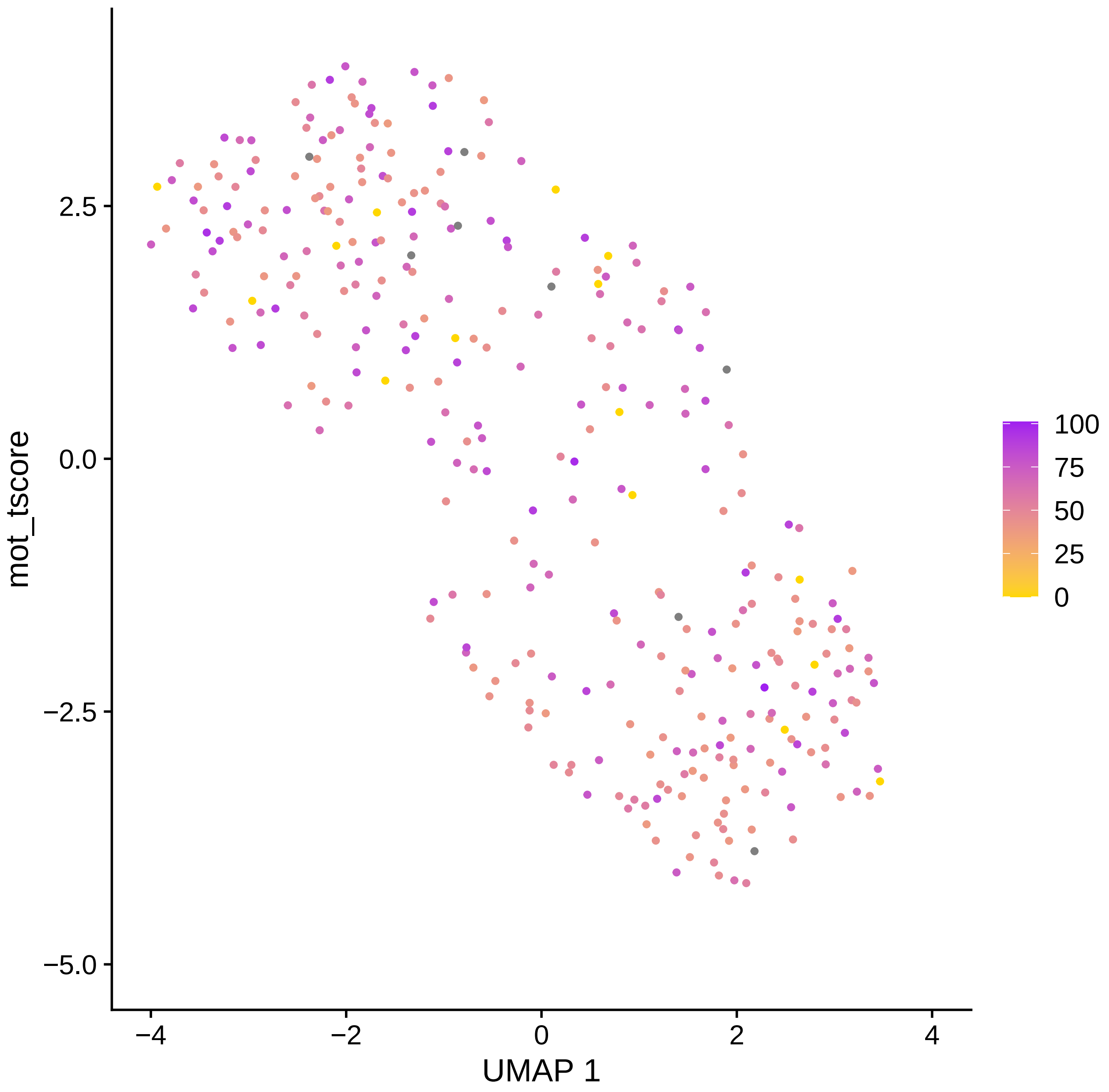

mot_tscore
2.5
0.0
−2.5
−5.0
−4
−2
0
2
4
UMAP 1
100
75
50
25
0

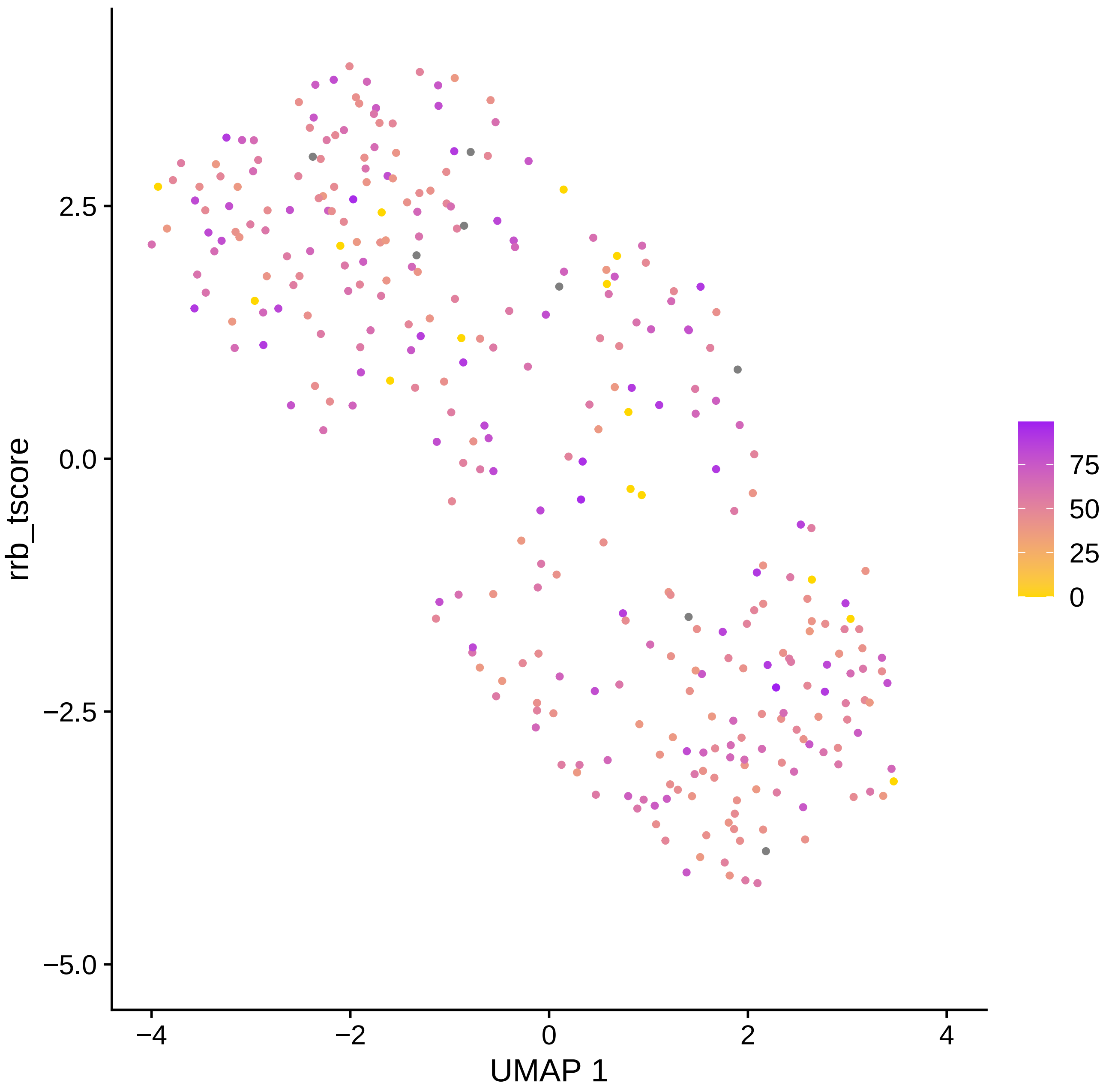

rrb_tscore
2.5
0.0
−2.5
−5.0
−4
−2
0
2
4
UMAP 1
75
50
25
0

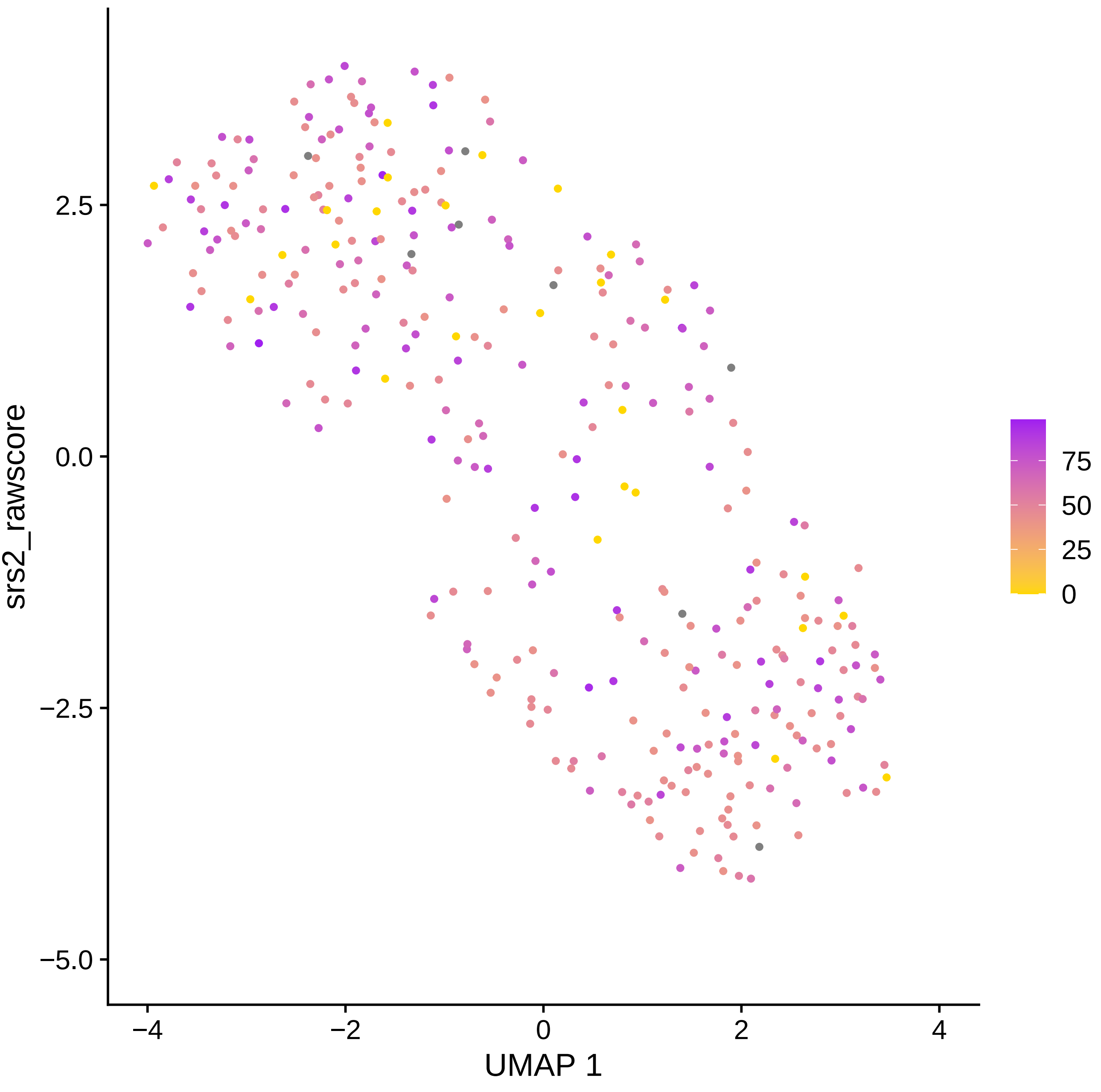
srs2_rawscore
2.5
0.0
−2.5
−5.0
−4
−2
0
2
4
UMAP 1
75
50
25
0

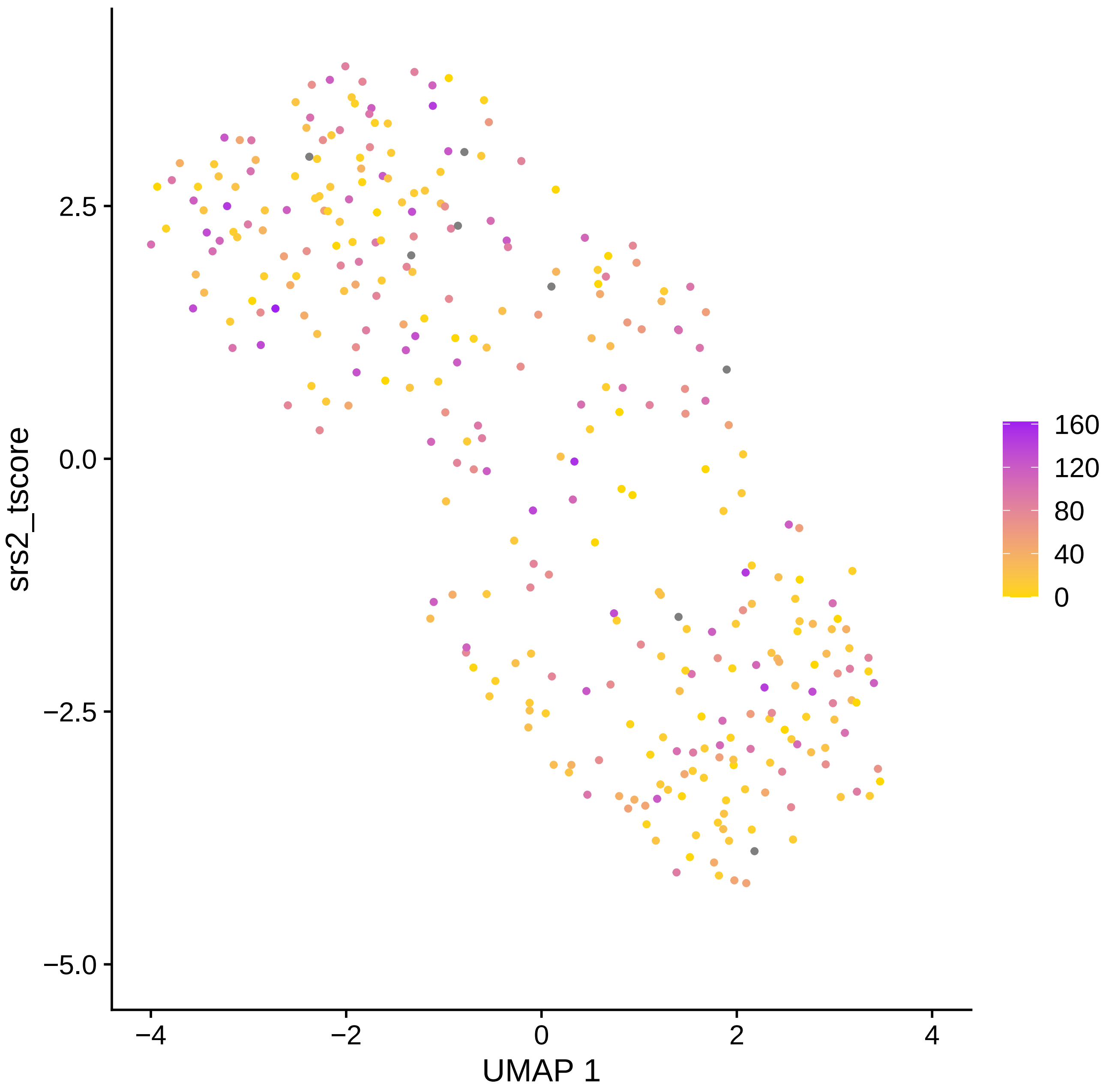
srs2_tscore
UMAP 1
2.5
0.0
−2.5
−5.0
−4
−2
0
2
4
160
120
80
40
0

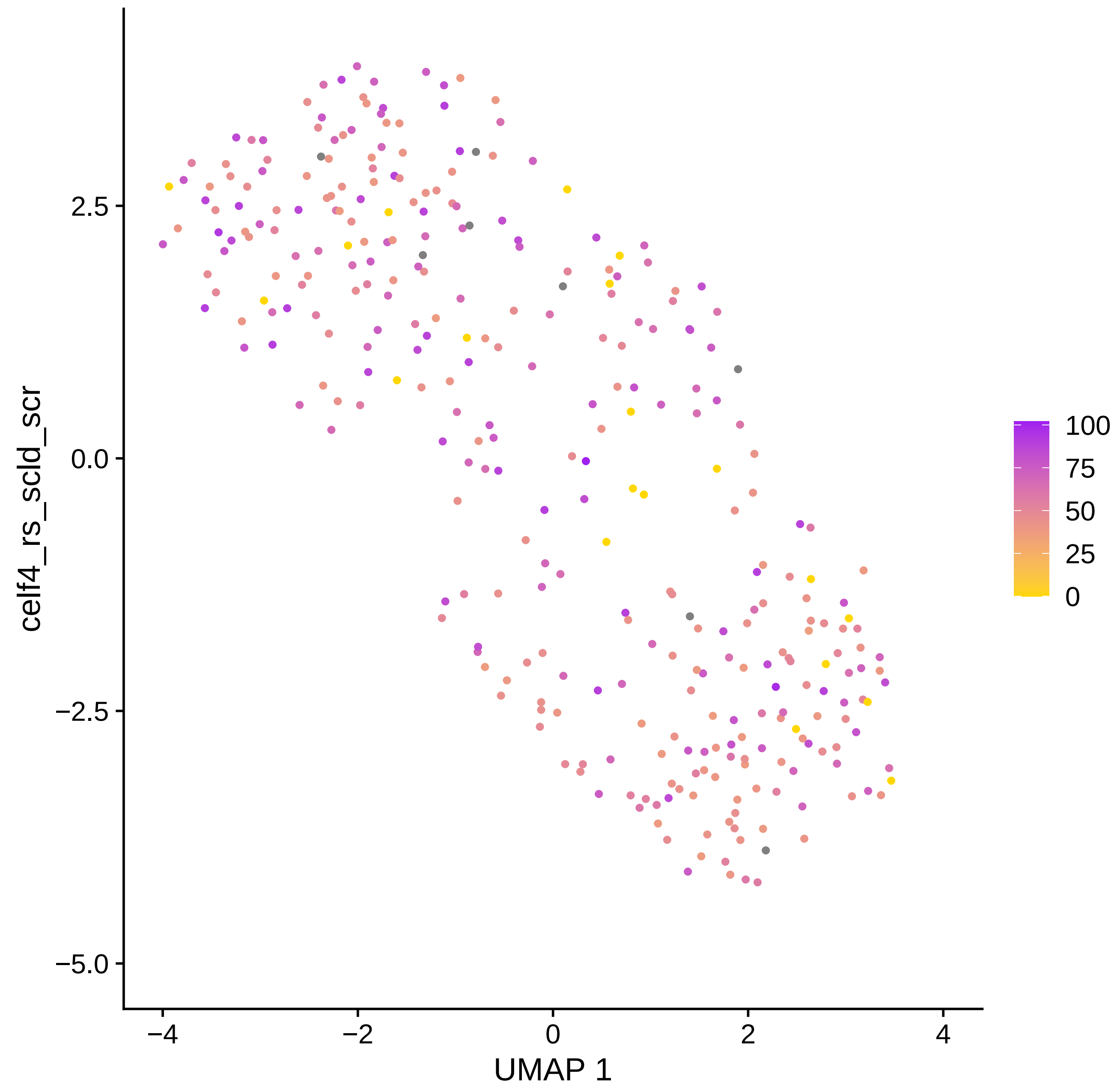

celf4_rs_scld_scr
UMAP 1
2.5
0.0
−2.5
−5.0
−4
−2
0
2
4
100
75
50
25
0

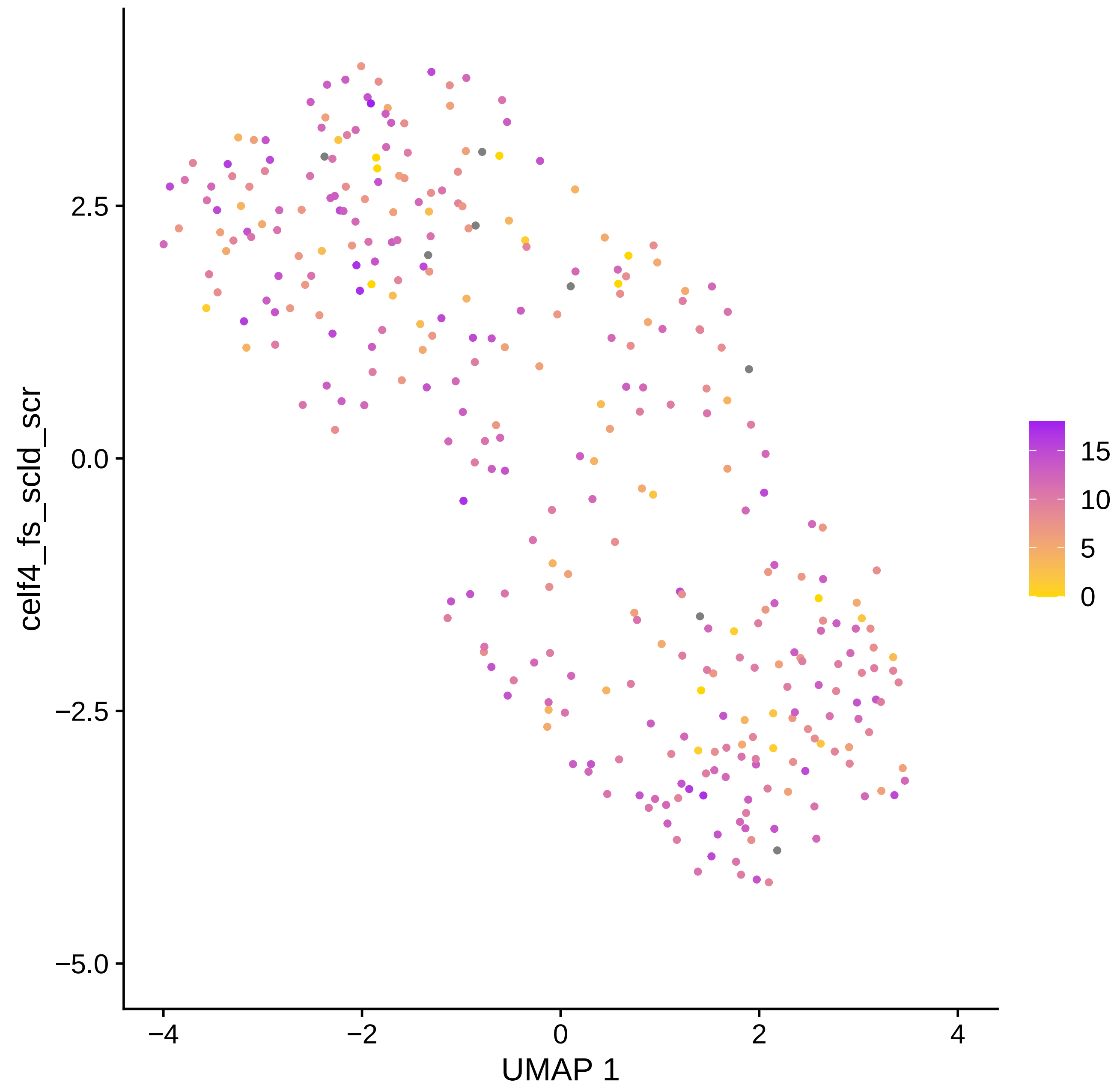
celf4_fs_scld_scr
UMAP 1
2.5
0.0
−2.5
−5.0
−4
−2
0
2
4
15
10
5
0

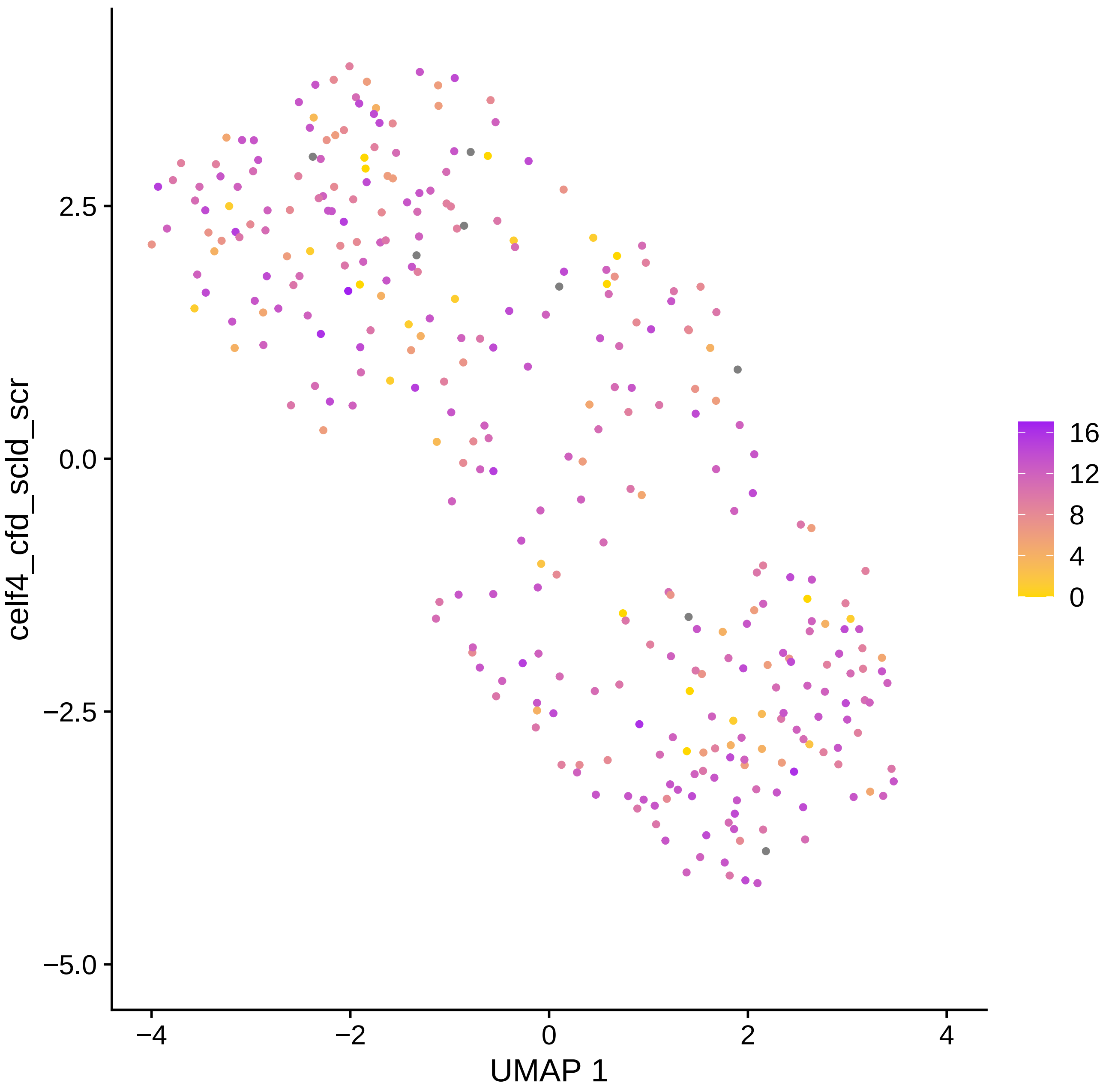
celf4_cfd_scld_scr
2.5
0.0
−2.5
−5.0
−4
−2
0
2
4
UMAP 1
16
12
8
4
0

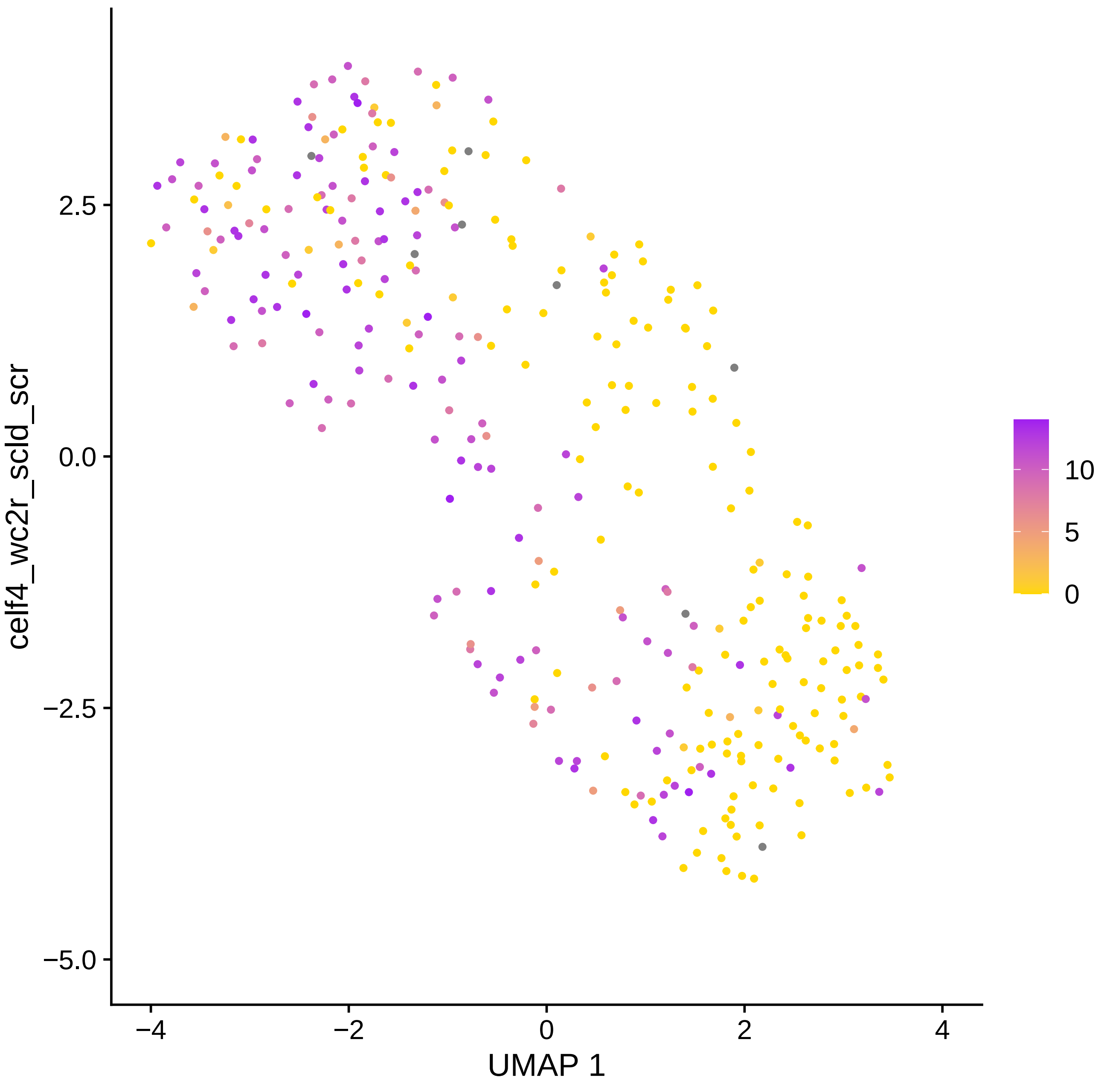

celf4_wc2r_scld_scr
2.5
0.0
−2.5
−5.0
−4
−2
0
2
4
UMAP 1
10
5
0

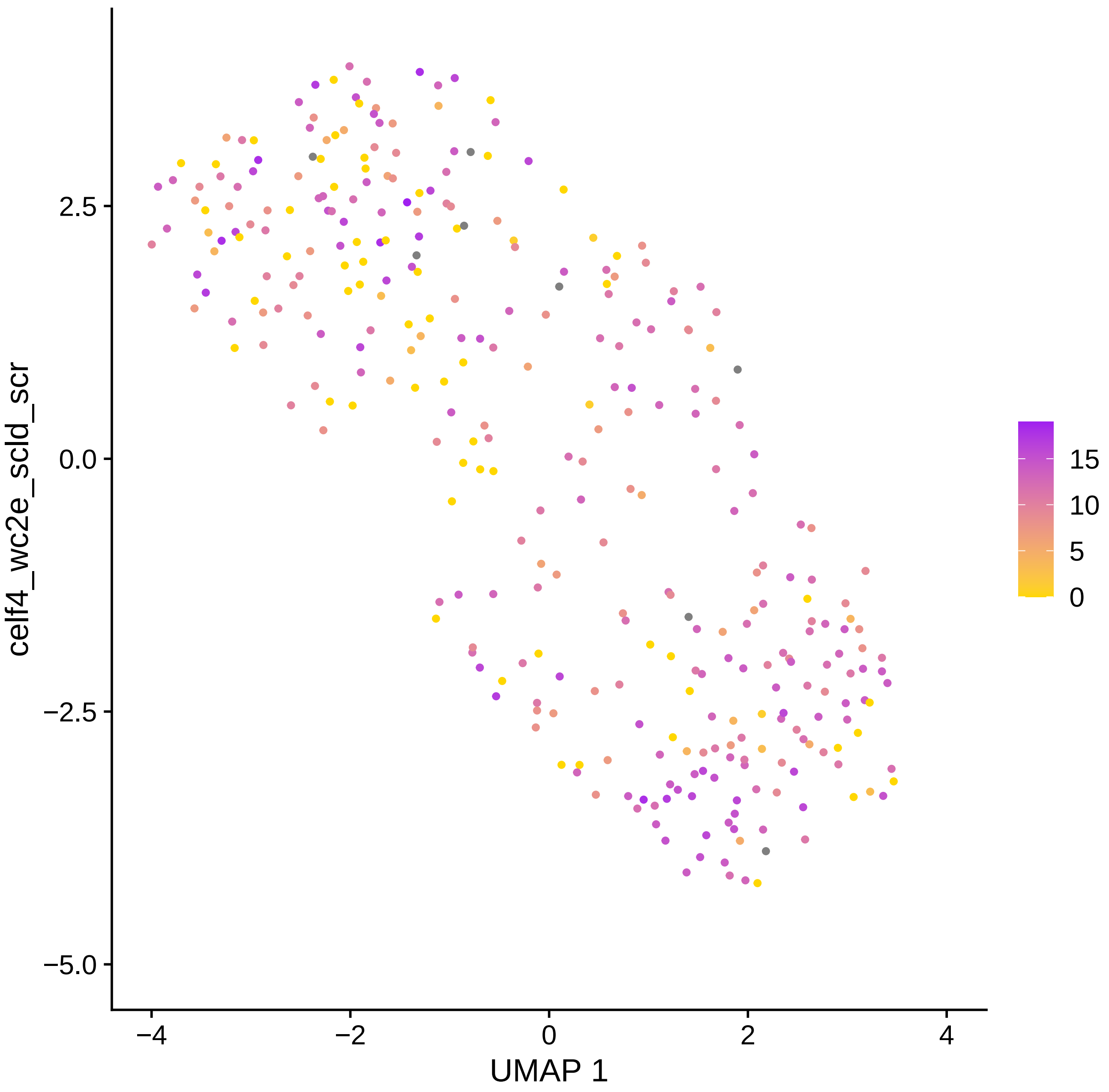

celf4_wc2e_scld_scr
2.5
0.0
−2.5
−5.0
−4
−2
0
2
4
UMAP 1
15
10
5
0

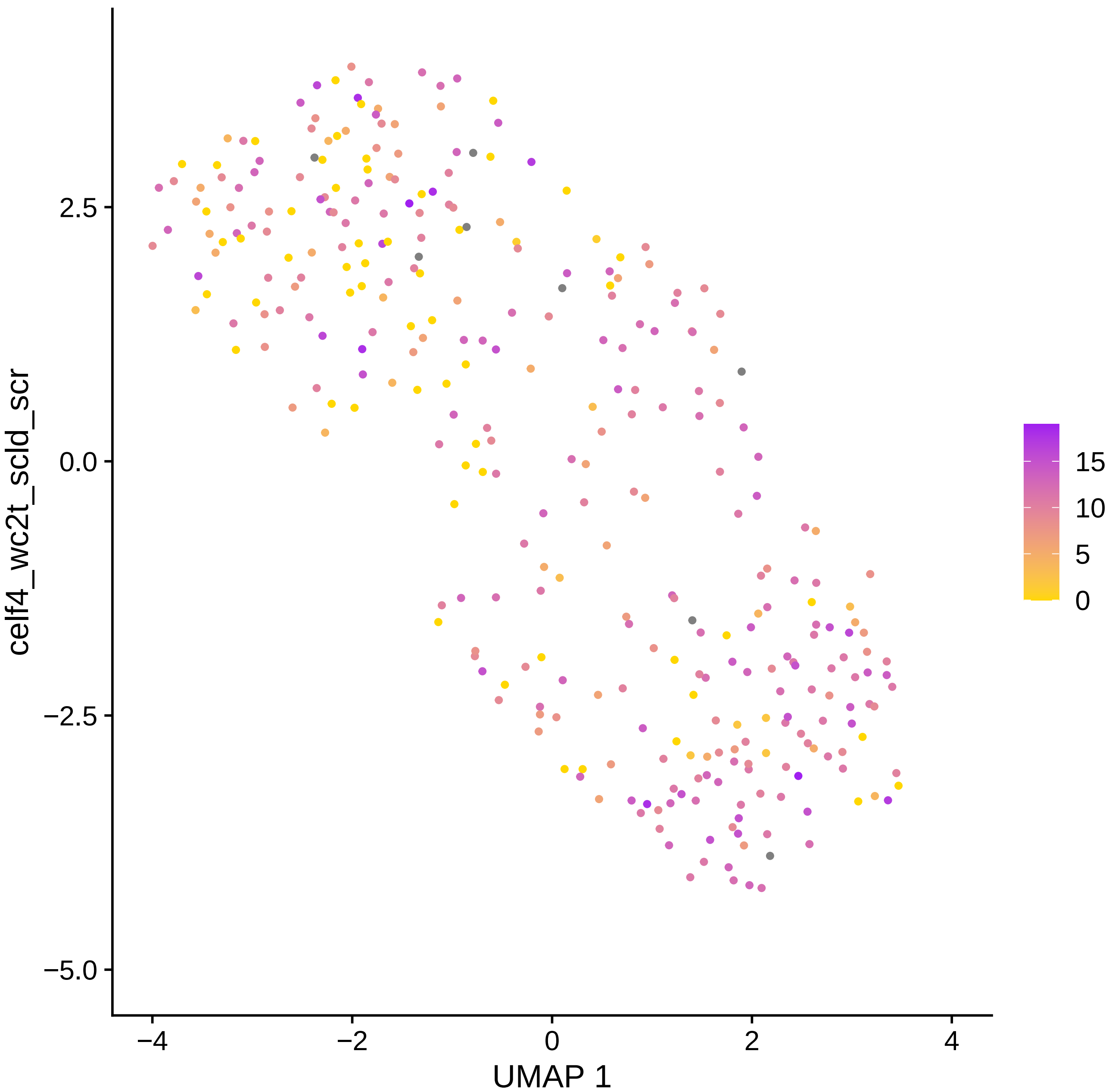

celf4_wc2t_scld_scr
UMAP 1
2.5
0.0
−2.5
−5.0
−4
−2
0
2
4
15
10
5
0

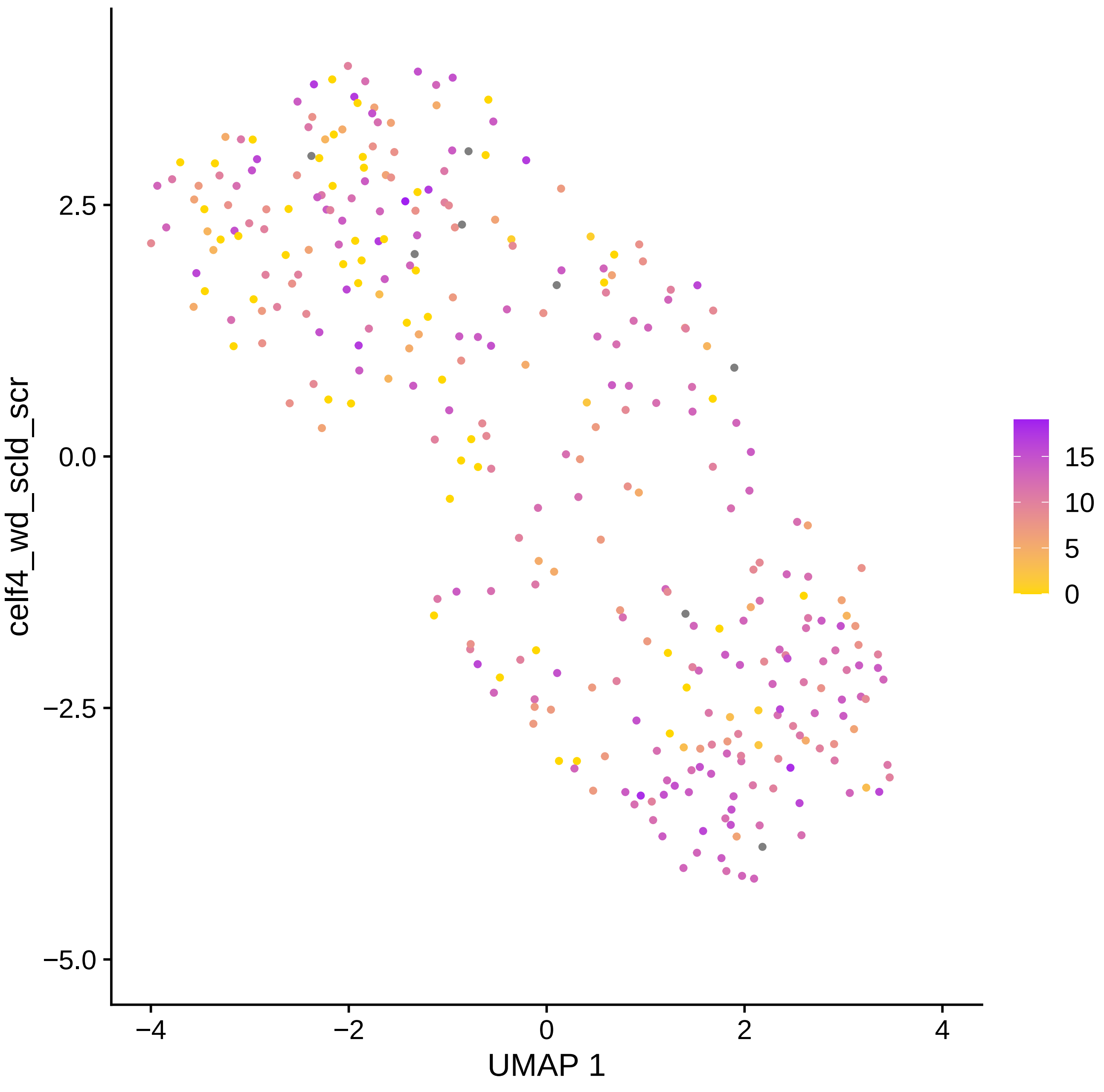
celf4_wd_scld_scr
UMAP 1
2.5
0.0
−2.5
−5.0
−4
−2
0
2
4
15
10
5
0

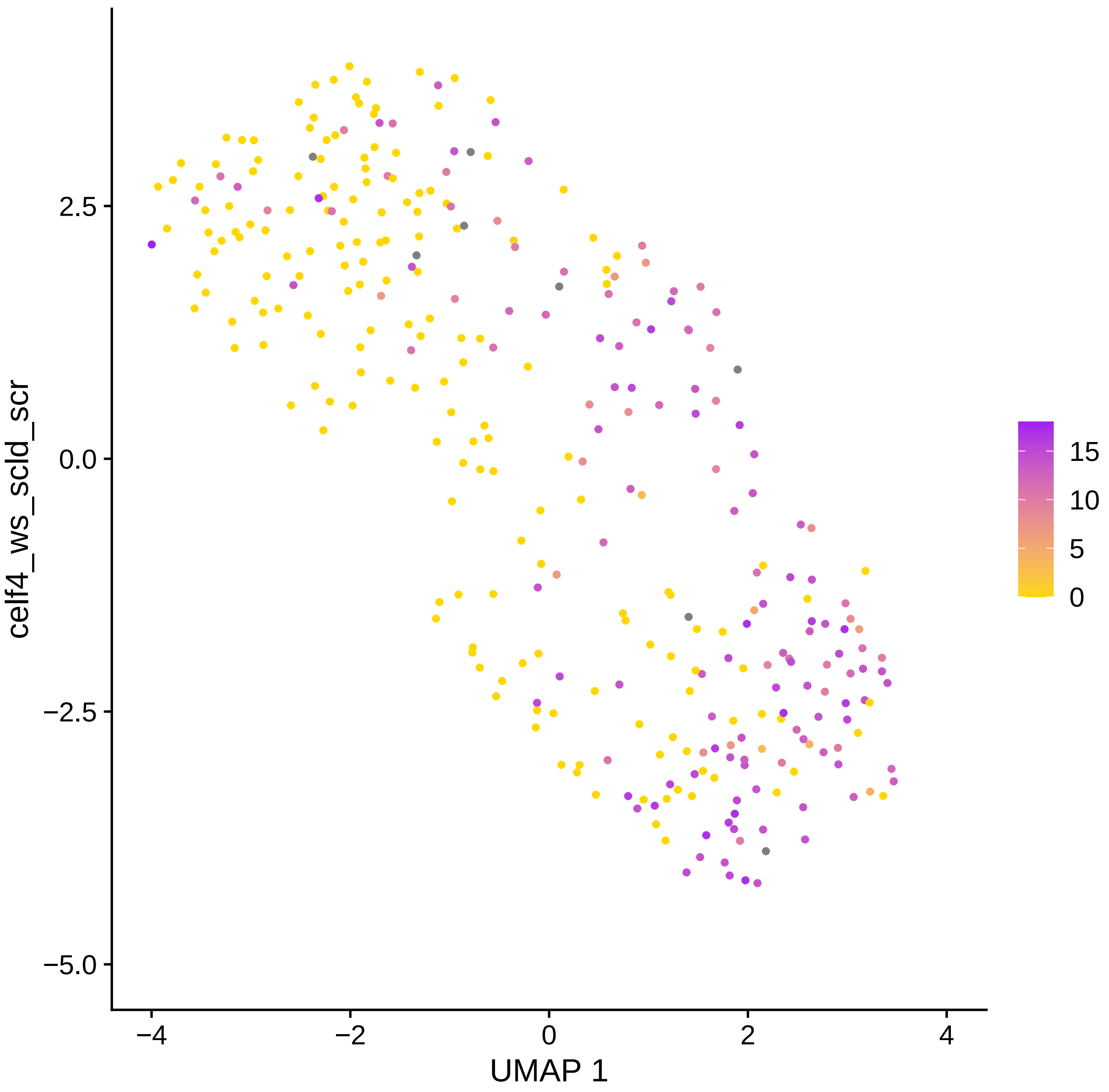

celf4_ws_scld_scr
UMAP 1
2.5
0.0
−2.5
−5.0
−4
−2
0
2
4
15
10
5
0

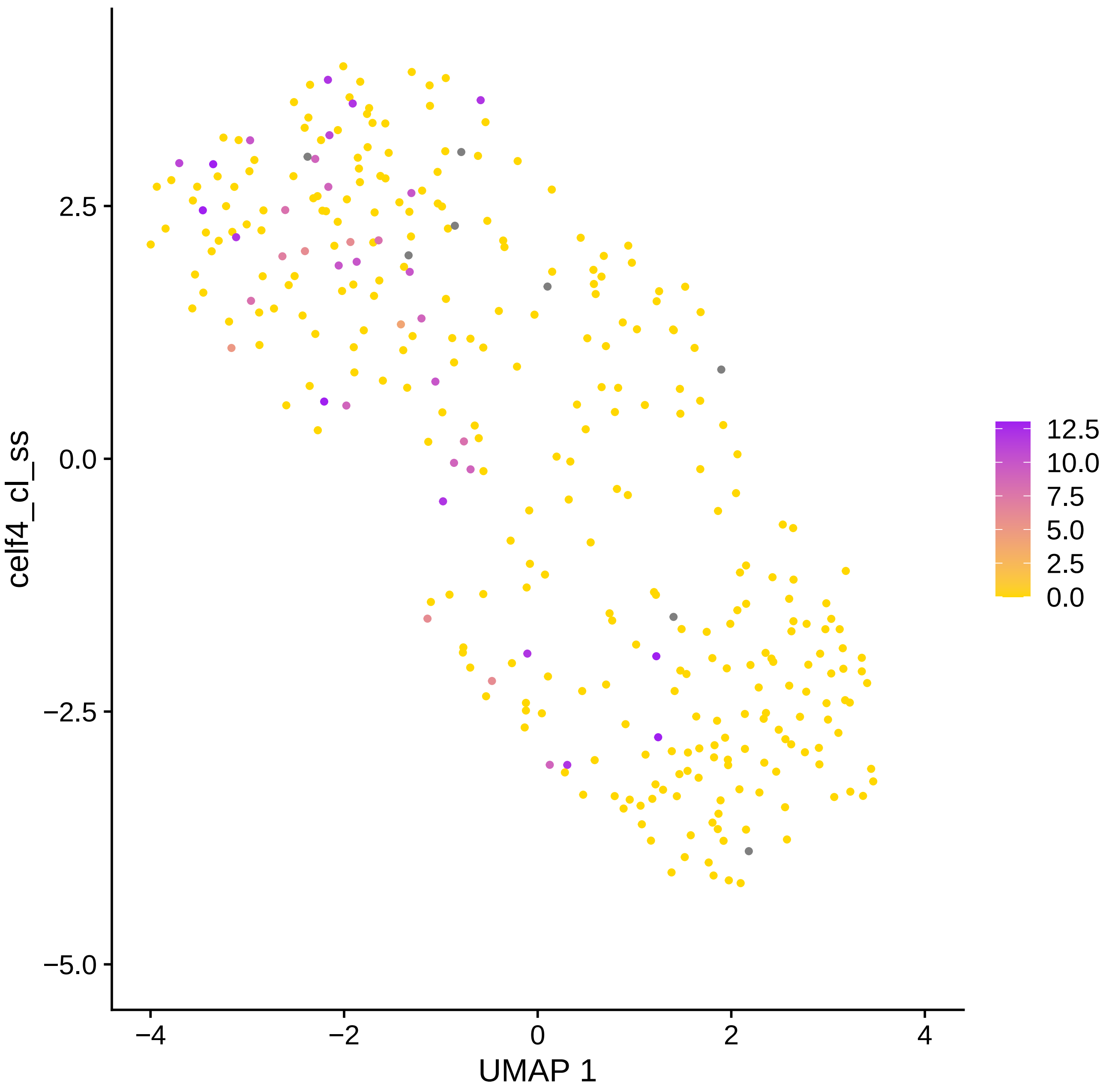

celf4_cl_ss
2.5
0.0
−2.5
−5.0
−4
−2
0
2
4
UMAP 1
12.5
10.0
7.5
5.0
2.5
0.0

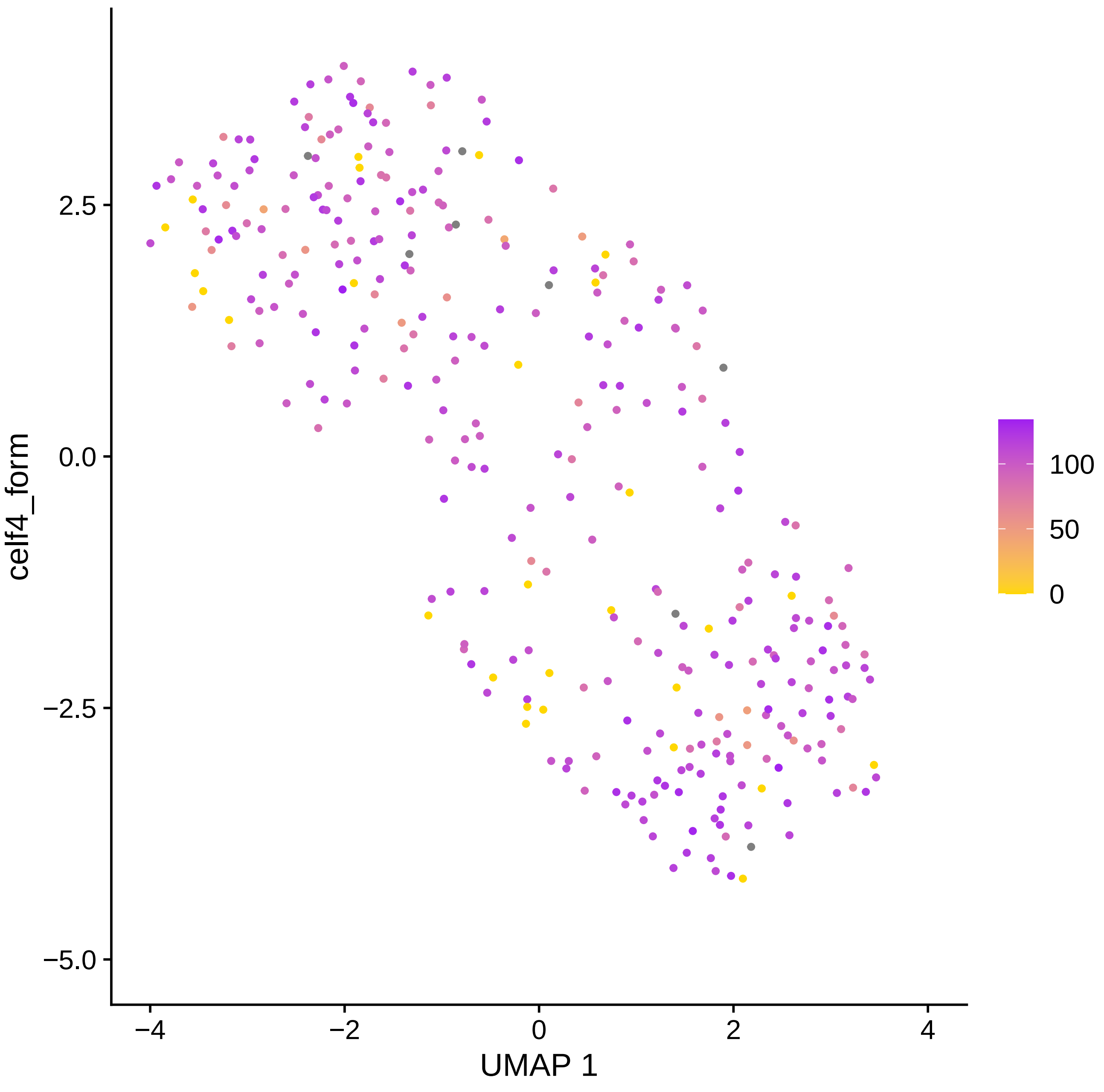

celf4_form
2.5
0.0
−2.5
−5.0
−4
−2
0
2
4
UMAP 1
100
50
0

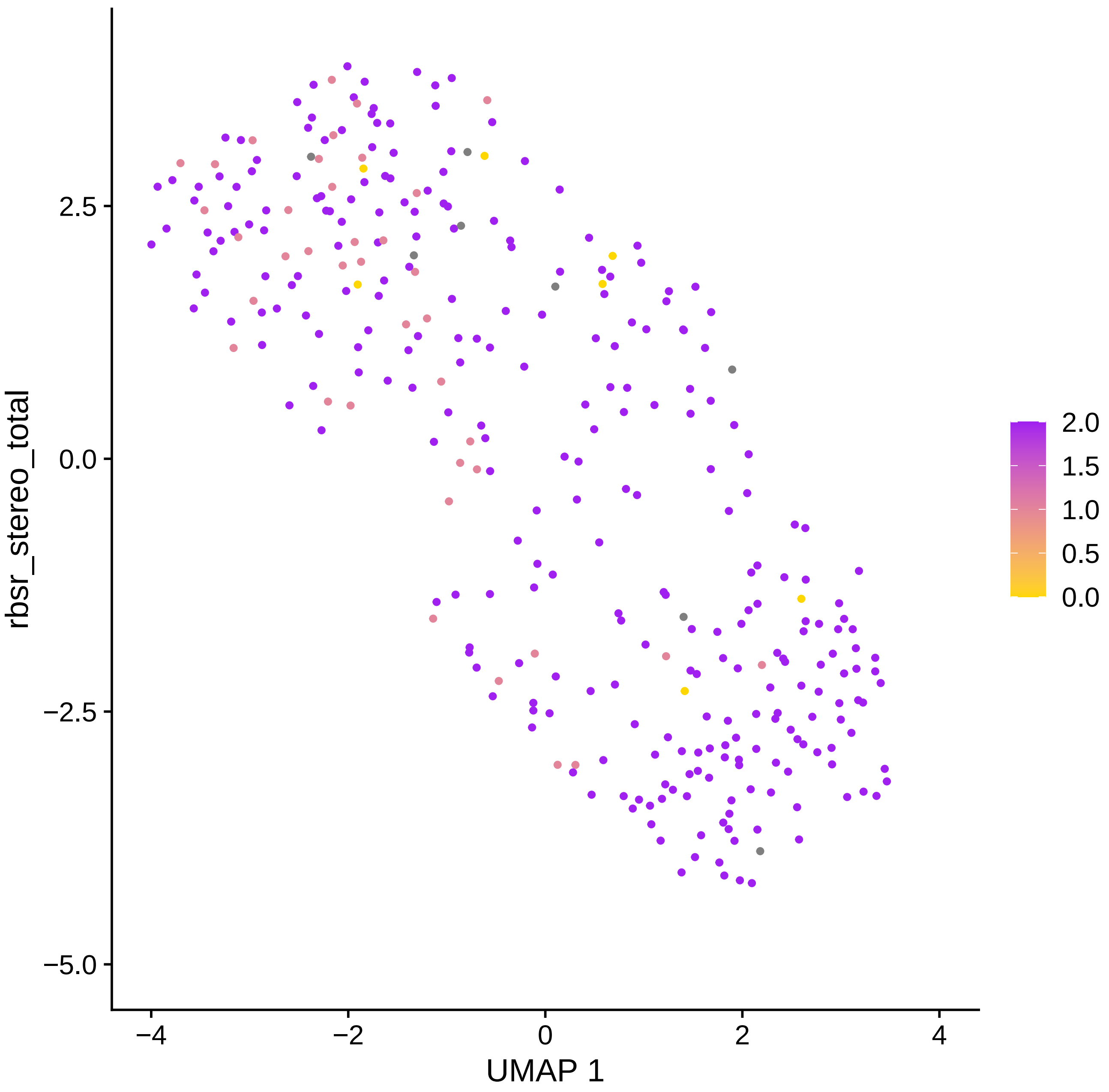

rbsr_stereo_total
2.5
0.0
−2.5
−5.0
−4
−2
0
2
4
UMAP 1
2.0
1.5
1.0
0.5
0.0

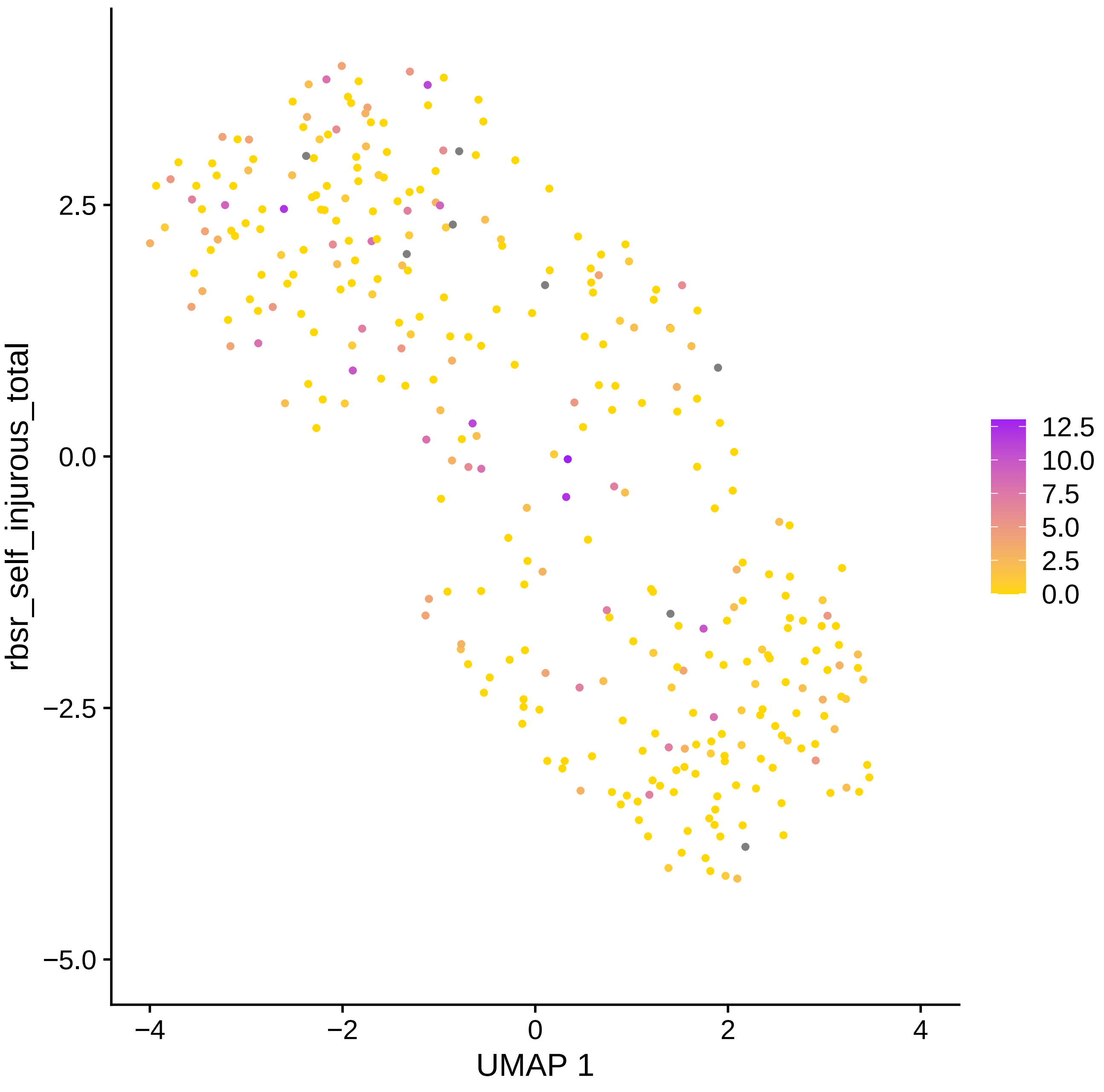

rbsr_self_injurous_total
UMAP 1
2.5
0.0
−2.5
−5.0
−4
−2
0
2
4
12.5
10.0
7.5
5.0
2.5
0.0

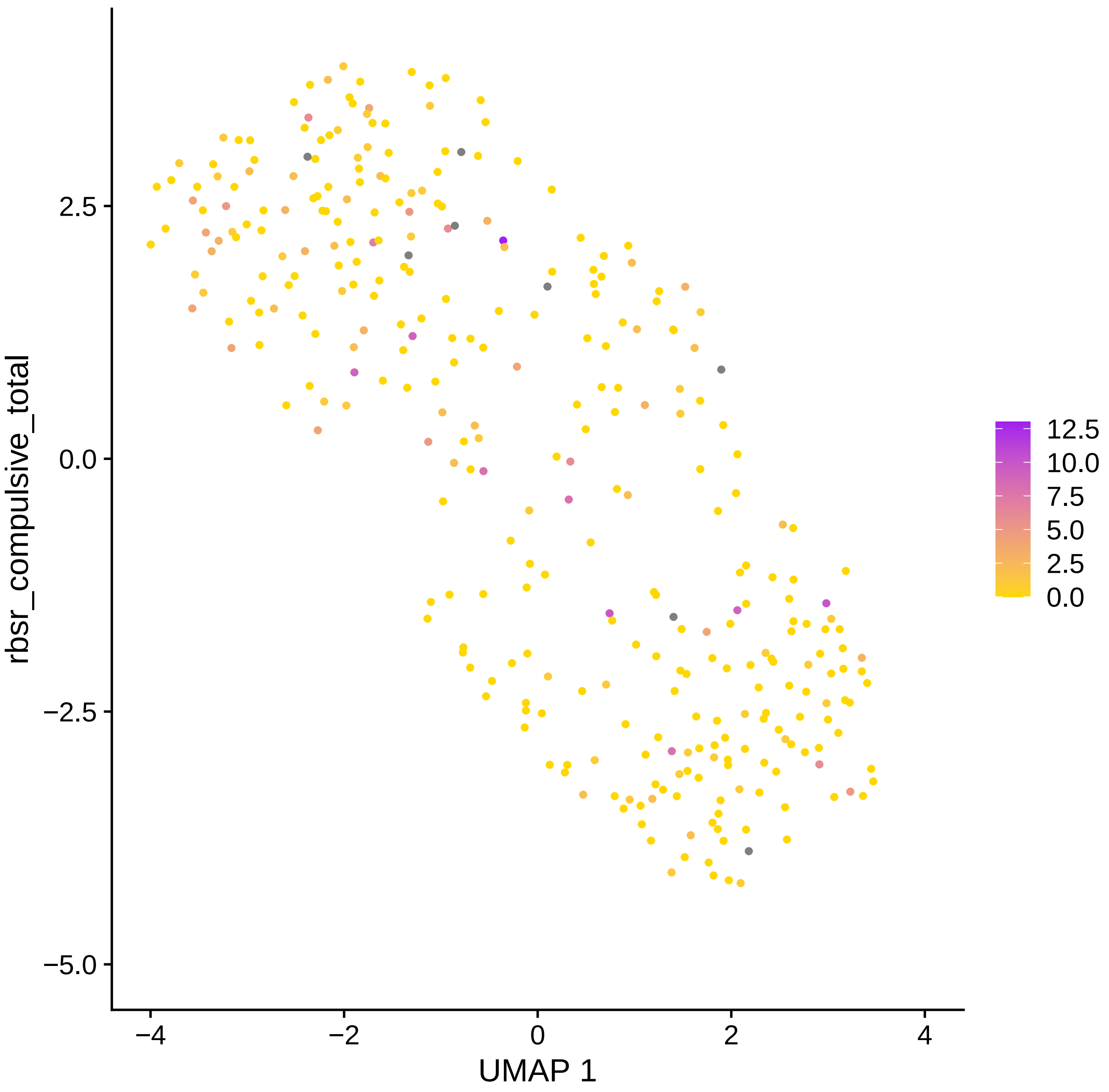

rbsr_compulsive_total
2.5
0.0
−2.5
−5.0
−4
−2
0
2
4
UMAP 1
12.5
10.0
7.5
5.0
2.5
0.0

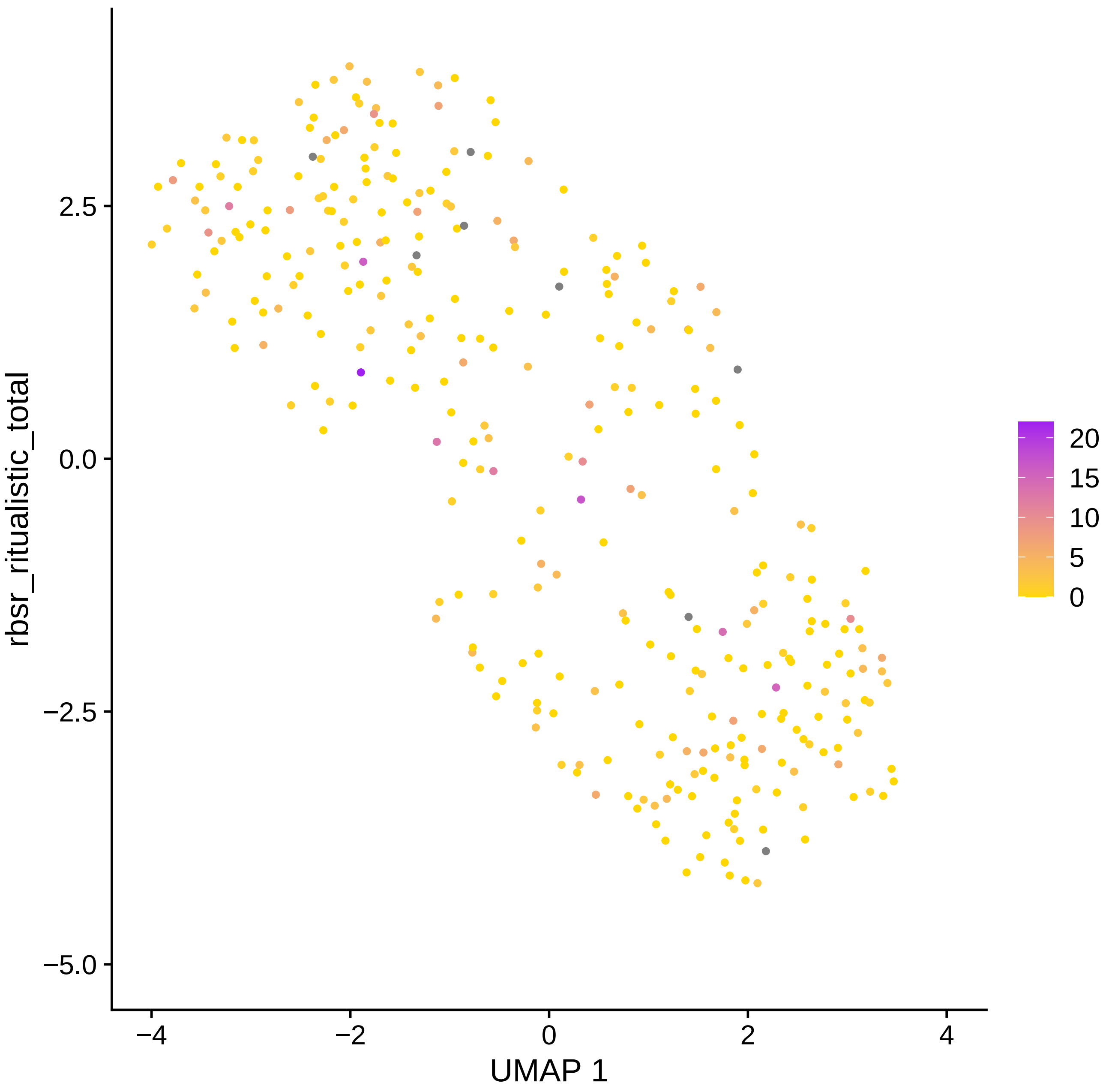
rbsr_ritualistic_total
2.5
0.0
−2.5
−5.0
−4
−2
0
2
4
UMAP 1
20
15
10
5
0

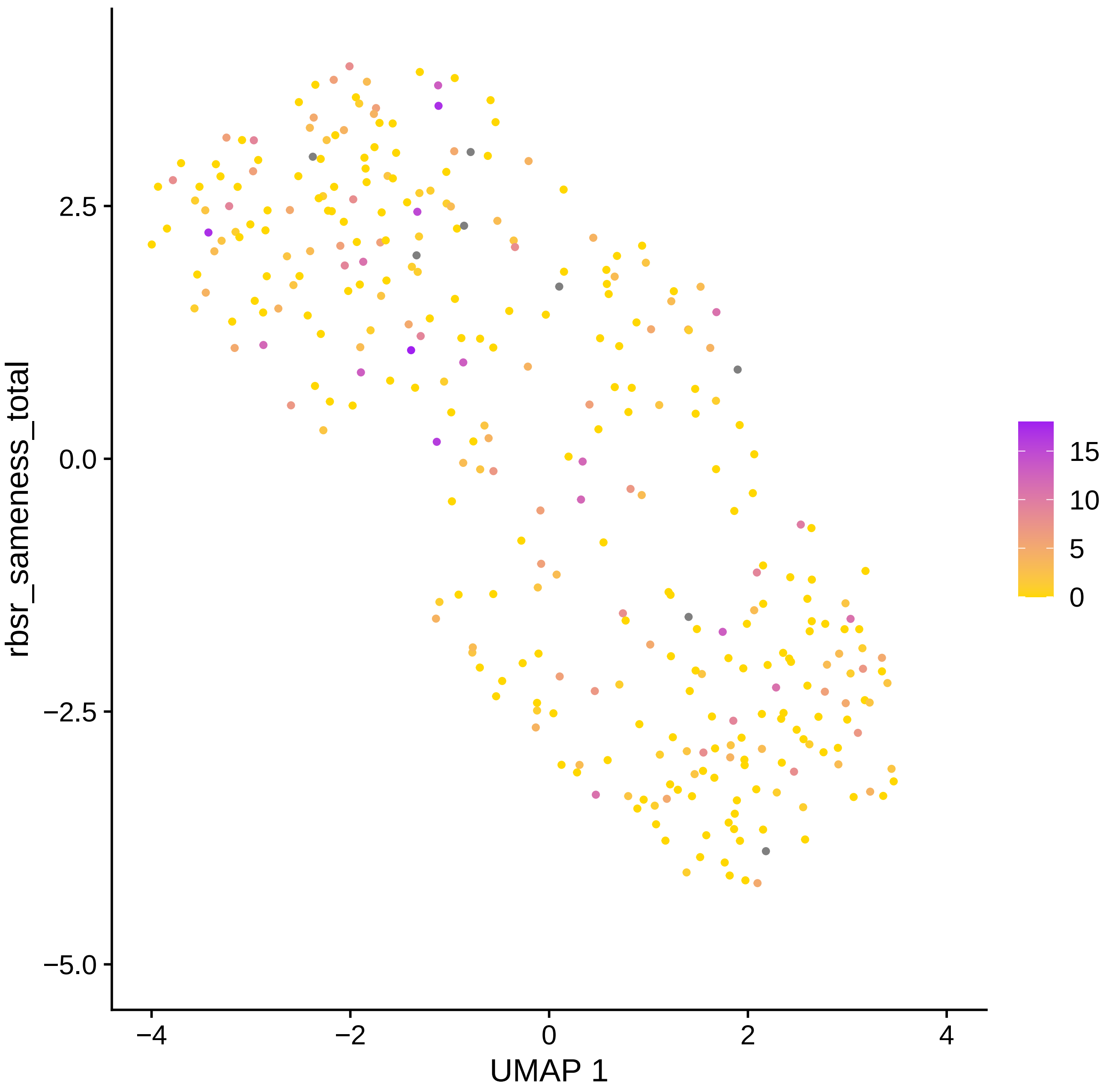
rbsr_sameness_total
UMAP 1
2.5
0.0
−2.5
−5.0
−4
−2
0
2
4
15
10
5
0

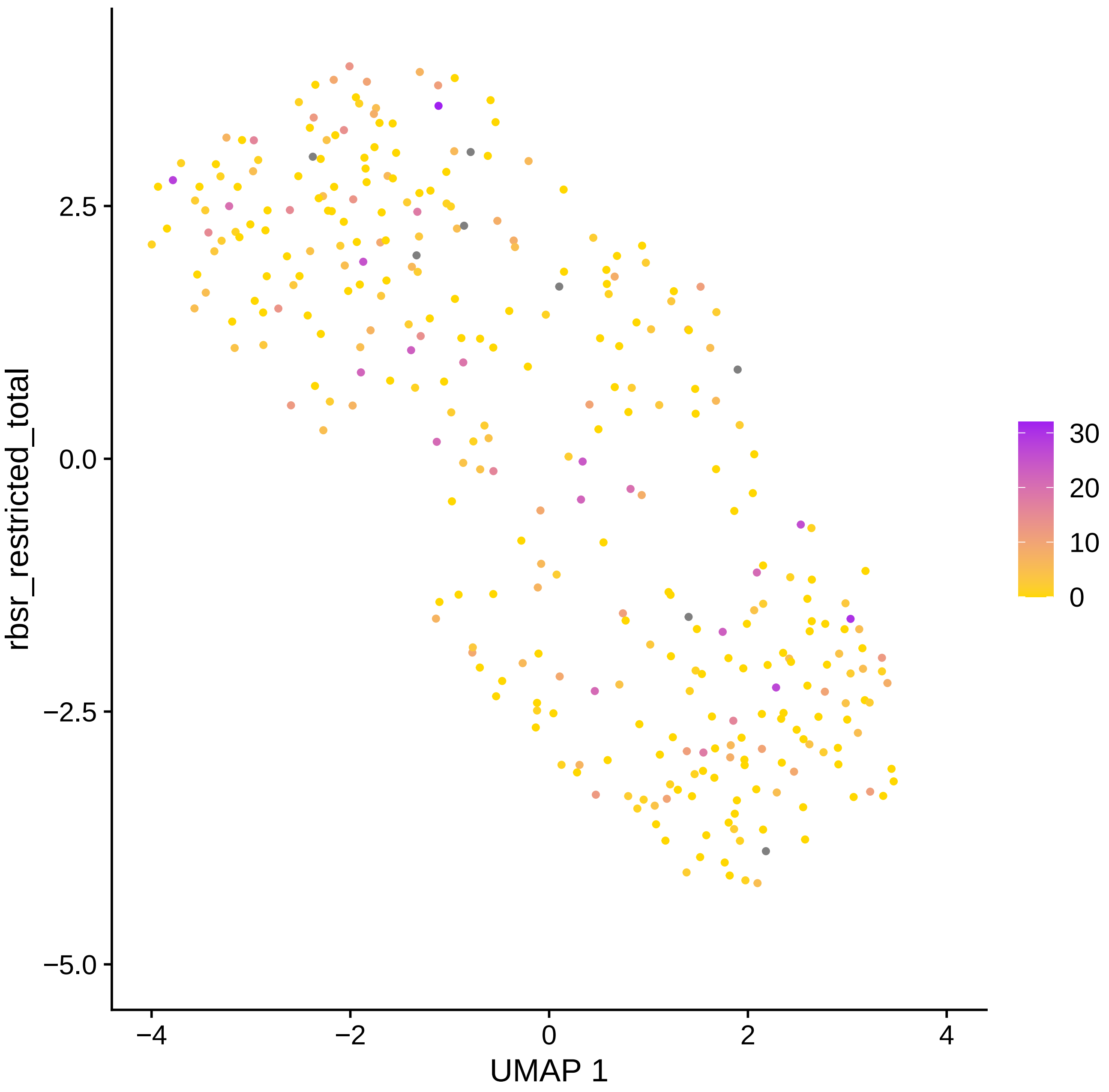

rbsr_restricted_total
UMAP 1
2.5
0.0
−2.5
−5.0
−4
−2
0
2
4
30
20
10
0

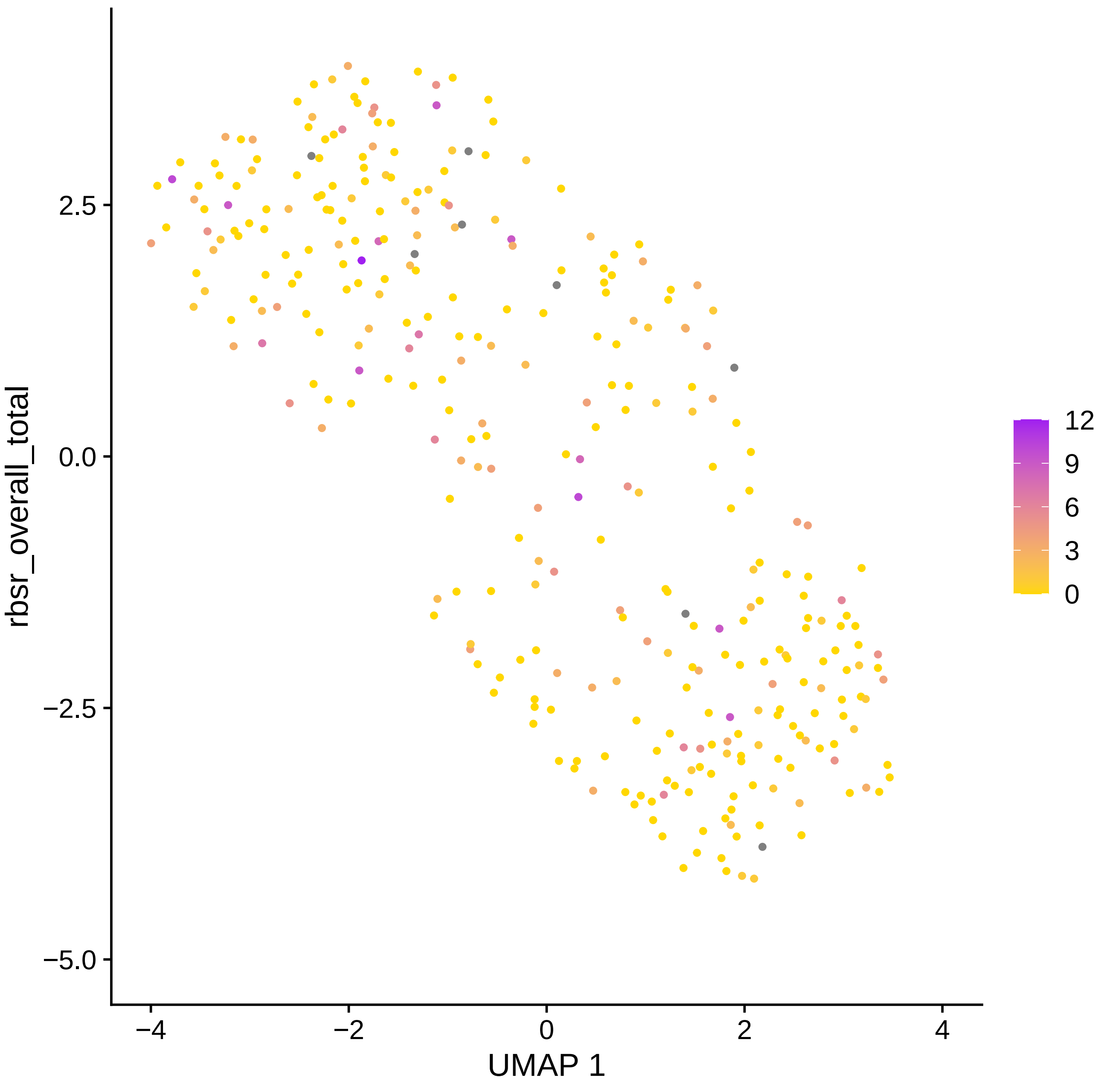
rbsr_overall_total
2.5
0.0
−2.5
−5.0
−4
−2
0
2
4
UMAP 1
12
9
6
3
0

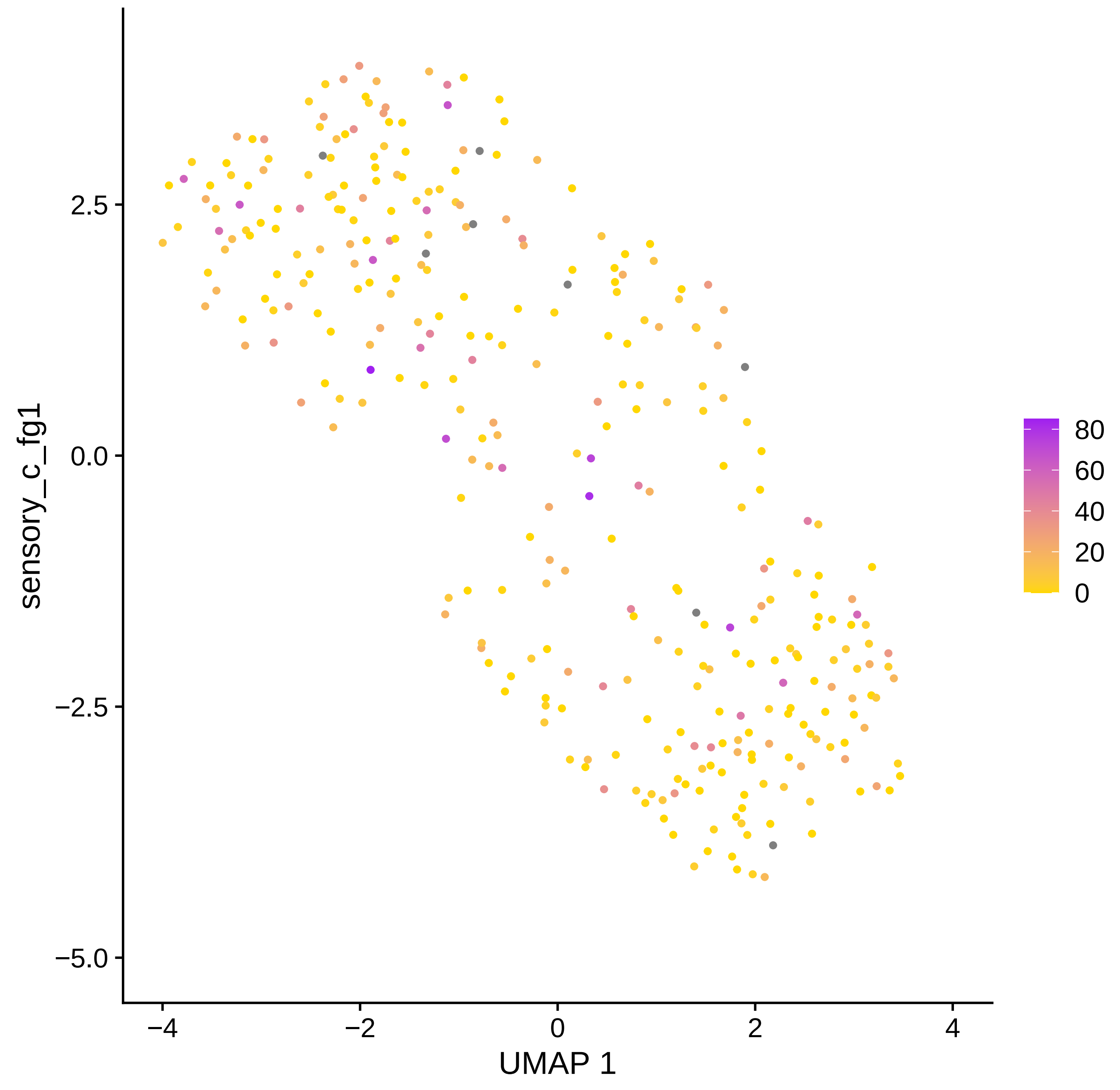

sensory_c_fg1
2.5
0.0
−2.5
−5.0
−4
−2
0
2
4
UMAP 1
80
60
40
20
0

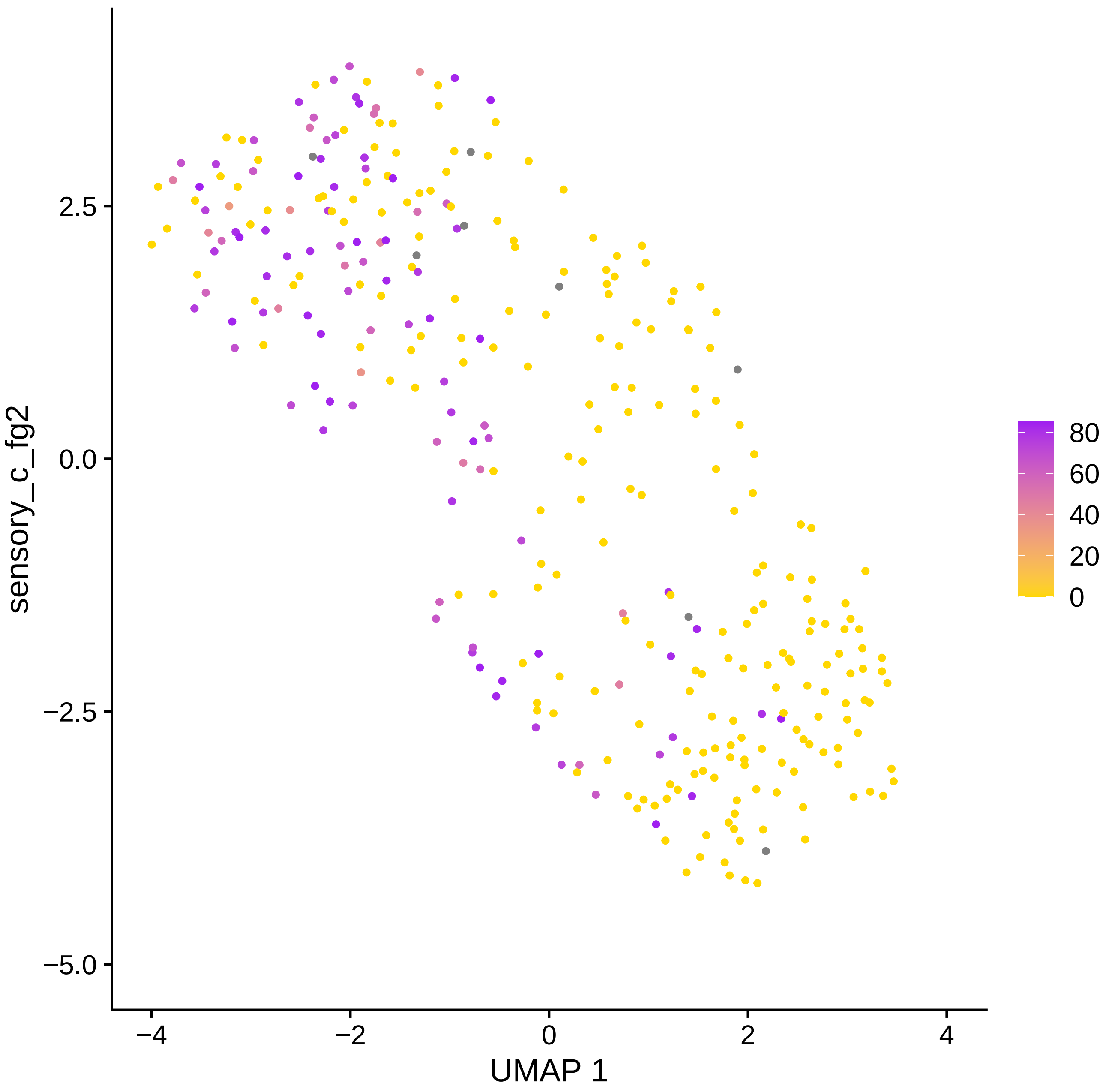

sensory_c_fg2
2.5
0.0
−2.5
−5.0
−4
−2
0
2
4
UMAP 1
80
60
40
20
0

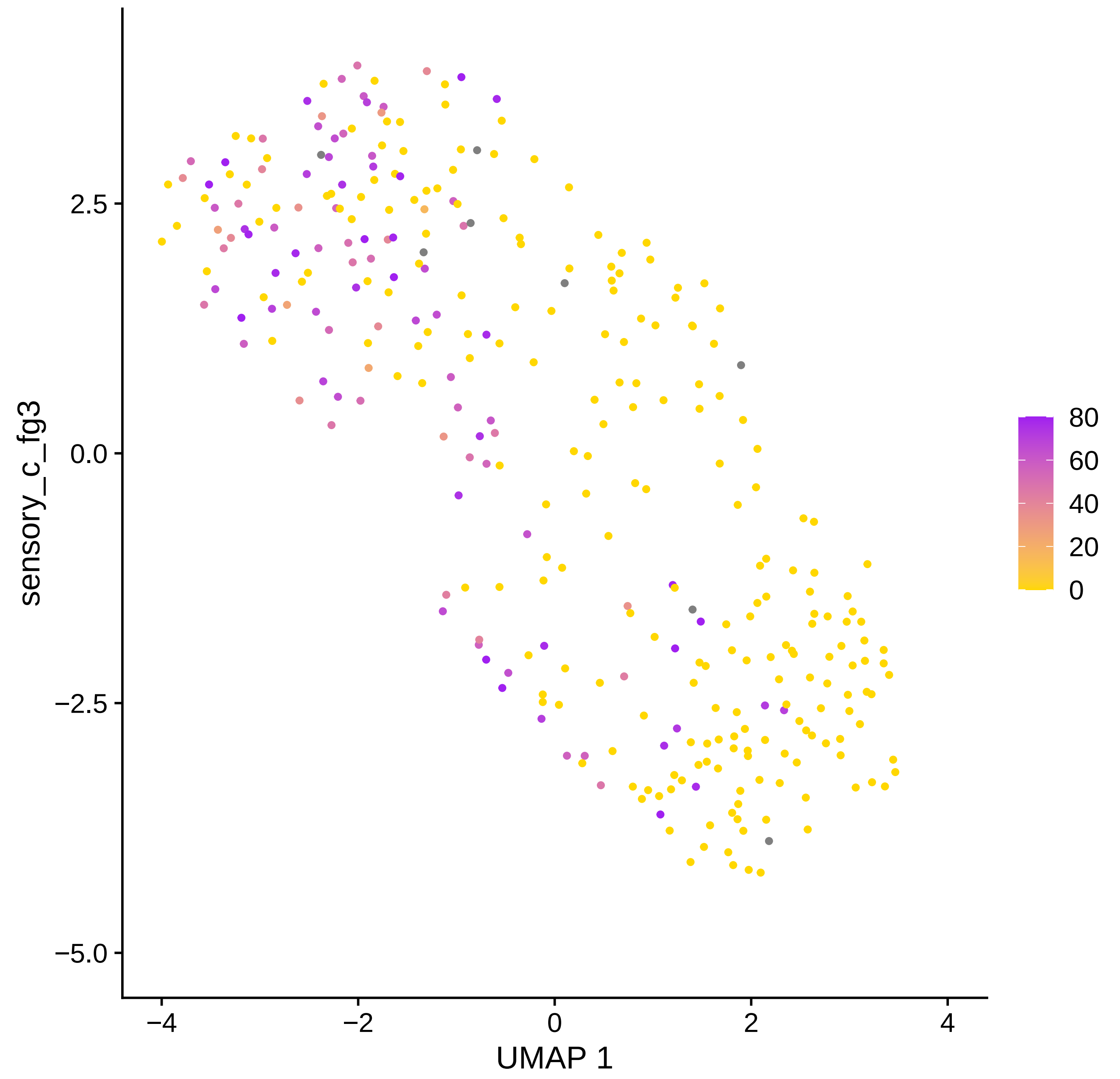

sensory_c_fg3
UMAP 1
2.5
0.0
−2.5
−5.0
−4
−2
0
2
4
80
60
40
20
0

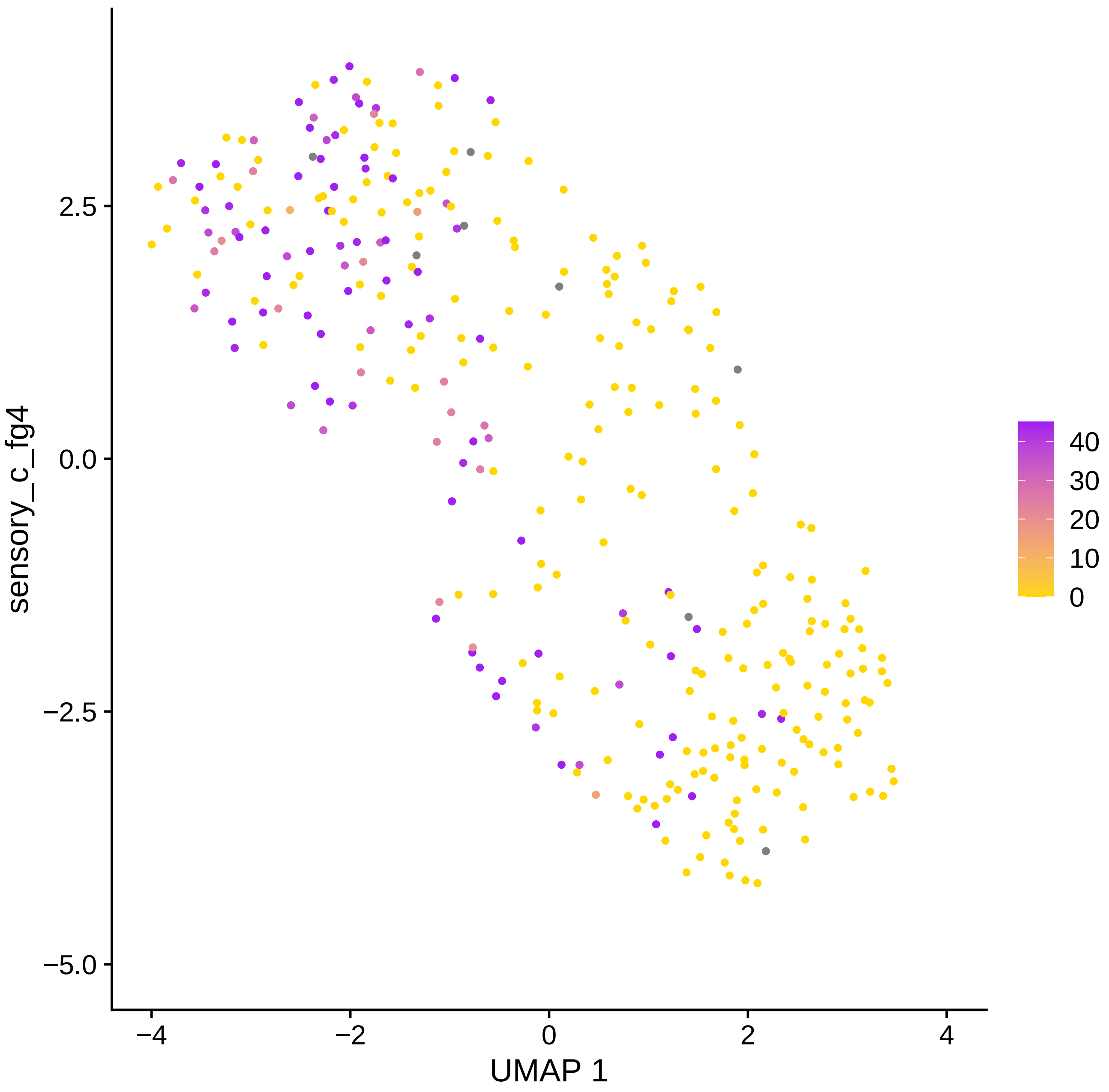

sensory_c_fg4
UMAP 1
2.5
0.0
−2.5
−5.0
−4
−2
0
2
4
40
30
20
10
0

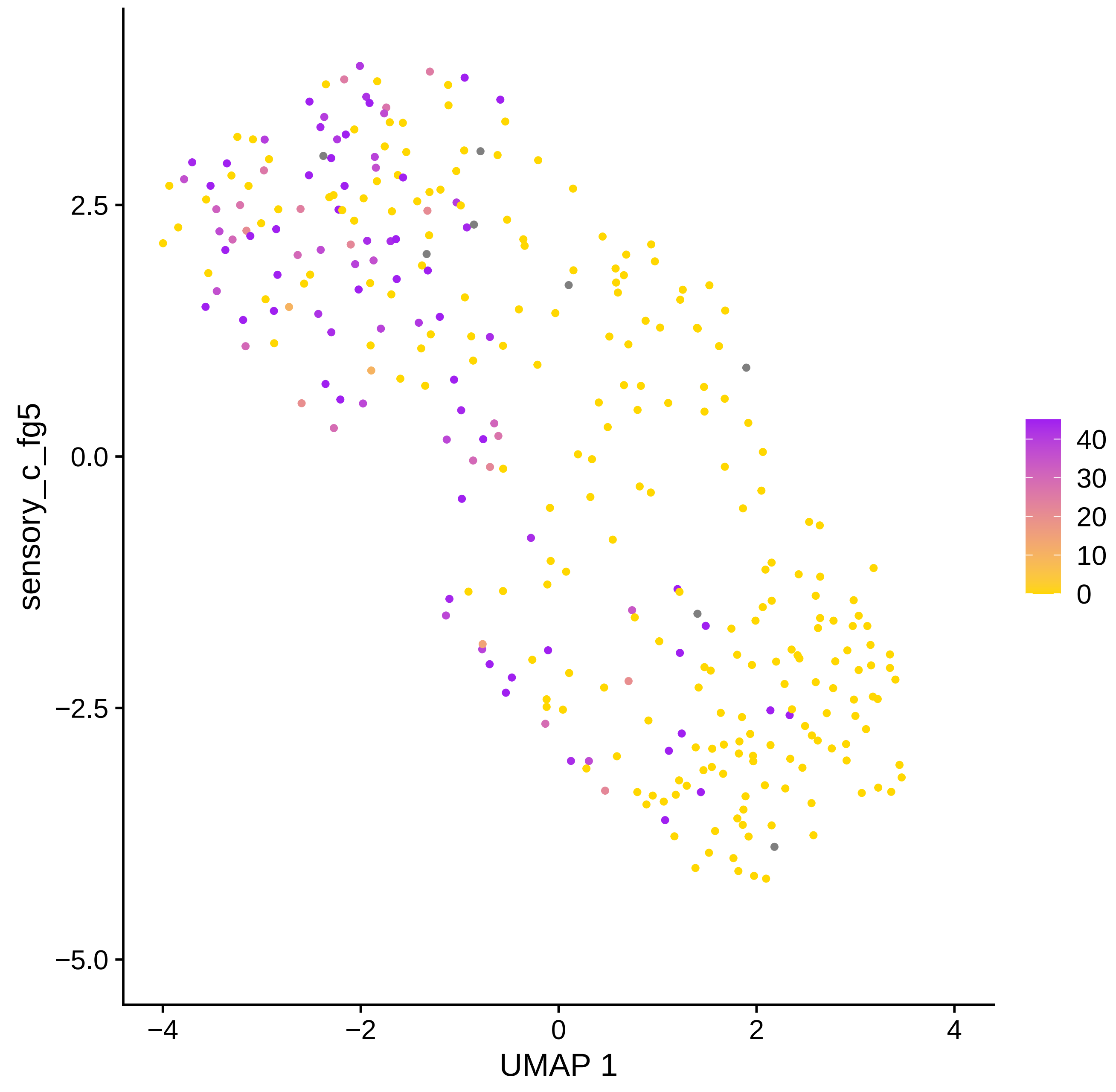
sensory_c_fg5
2.5
0.0
−2.5
−5.0
−4
−2
0
2
4
UMAP 1
40
30
20
10
0

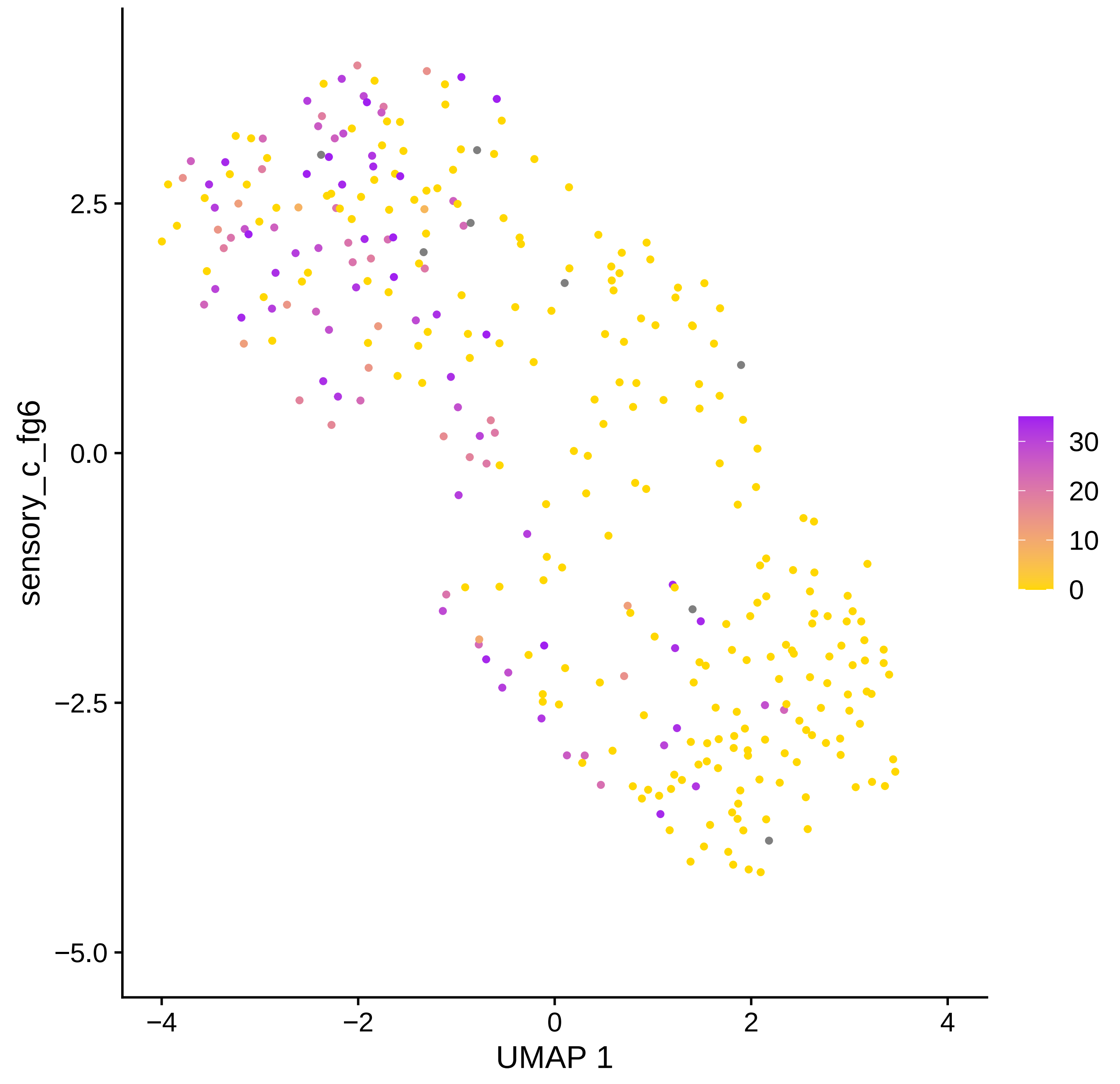

sensory_c_fg6
UMAP 1
2.5
0.0
−2.5
−5.0
−4
−2
0
2
4
30
20
10
0

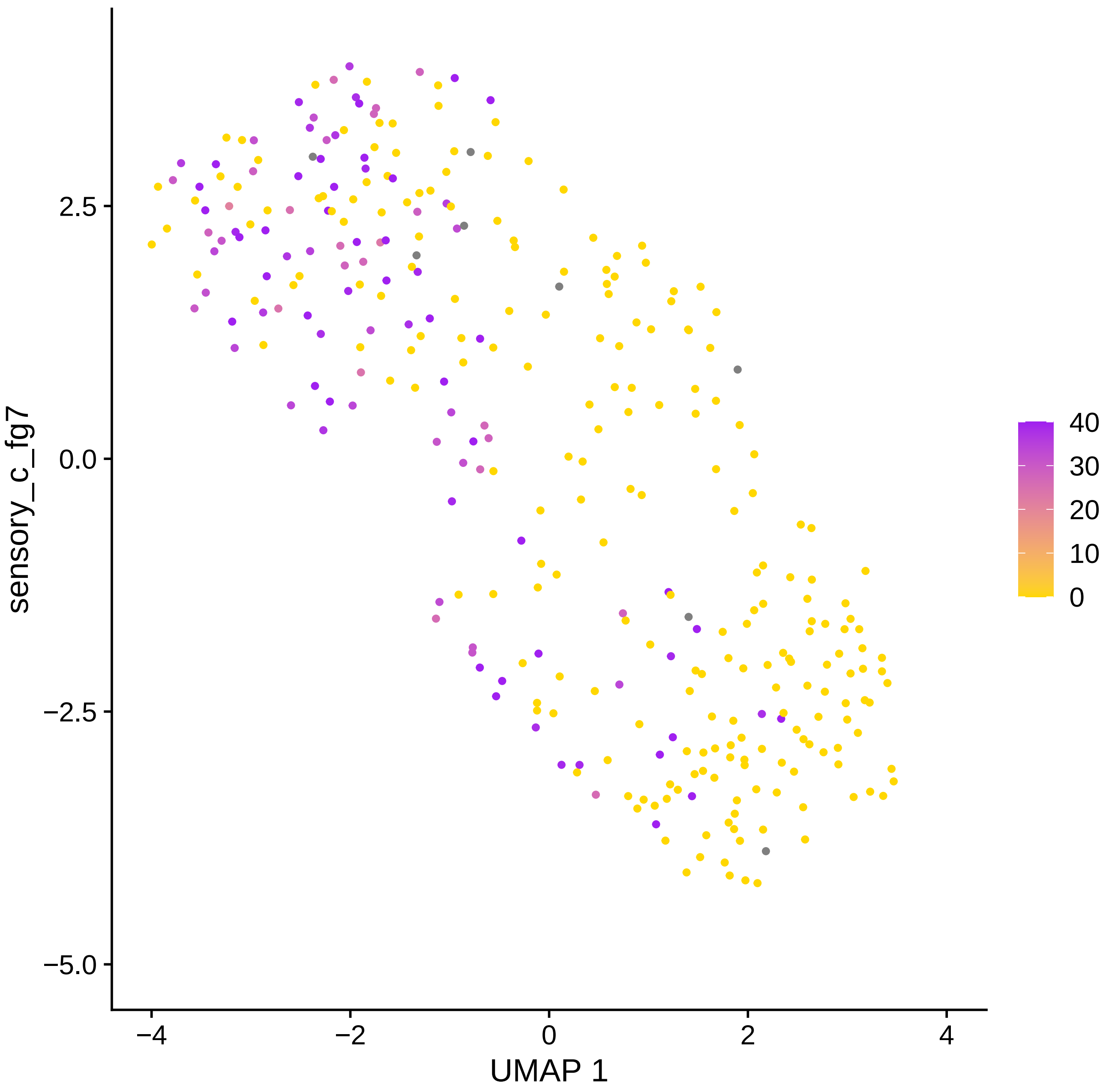
sensory_c_fg7
2.5
0.0
−2.5
−5.0
−4
−2
0
2
4
UMAP 1
40
30
20
10
0

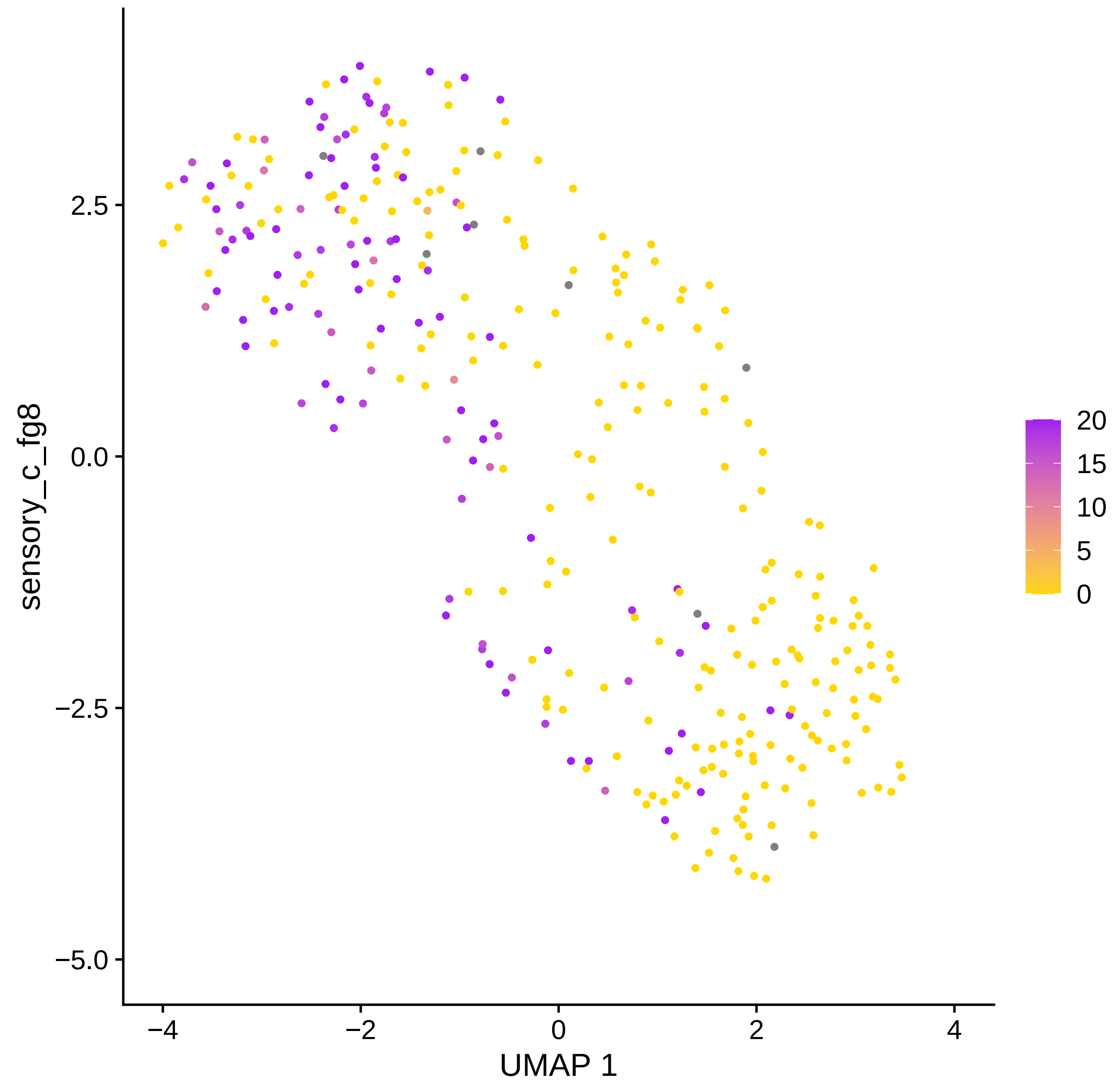
sensory_c_fg8
UMAP 1
2.5
0.0
−2.5
−5.0
−4
−2
0
2
4
20
15
10
5
0

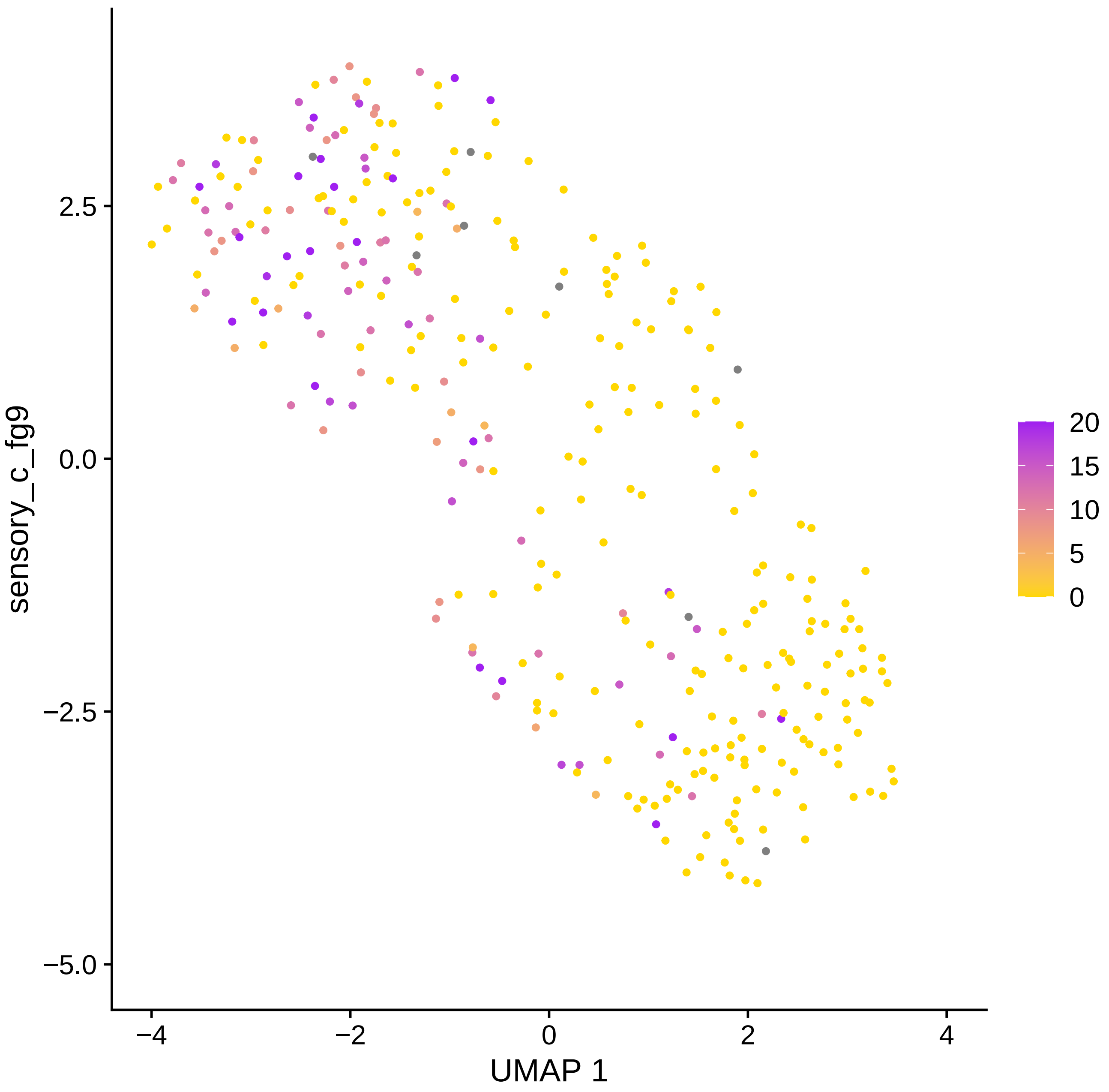

sensory_c_fg9
2.5
0.0
−2.5
−5.0
−4
−2
0
2
4
UMAP 1
20
15
10
5
0

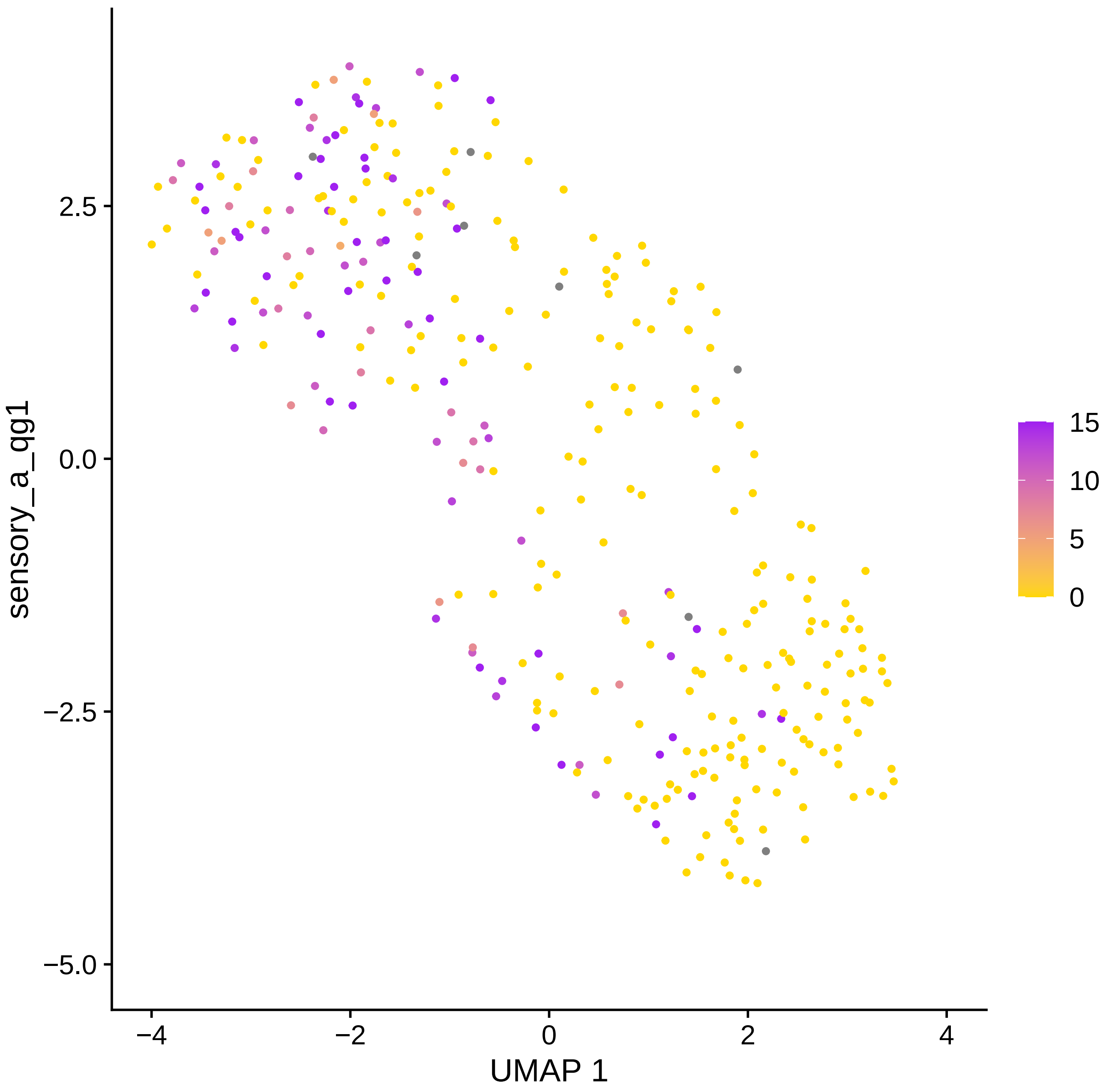

sensory_a_qg1
UMAP 1
2.5
0.0
−2.5
−5.0
−4
−2
0
2
4
15
10
5
0

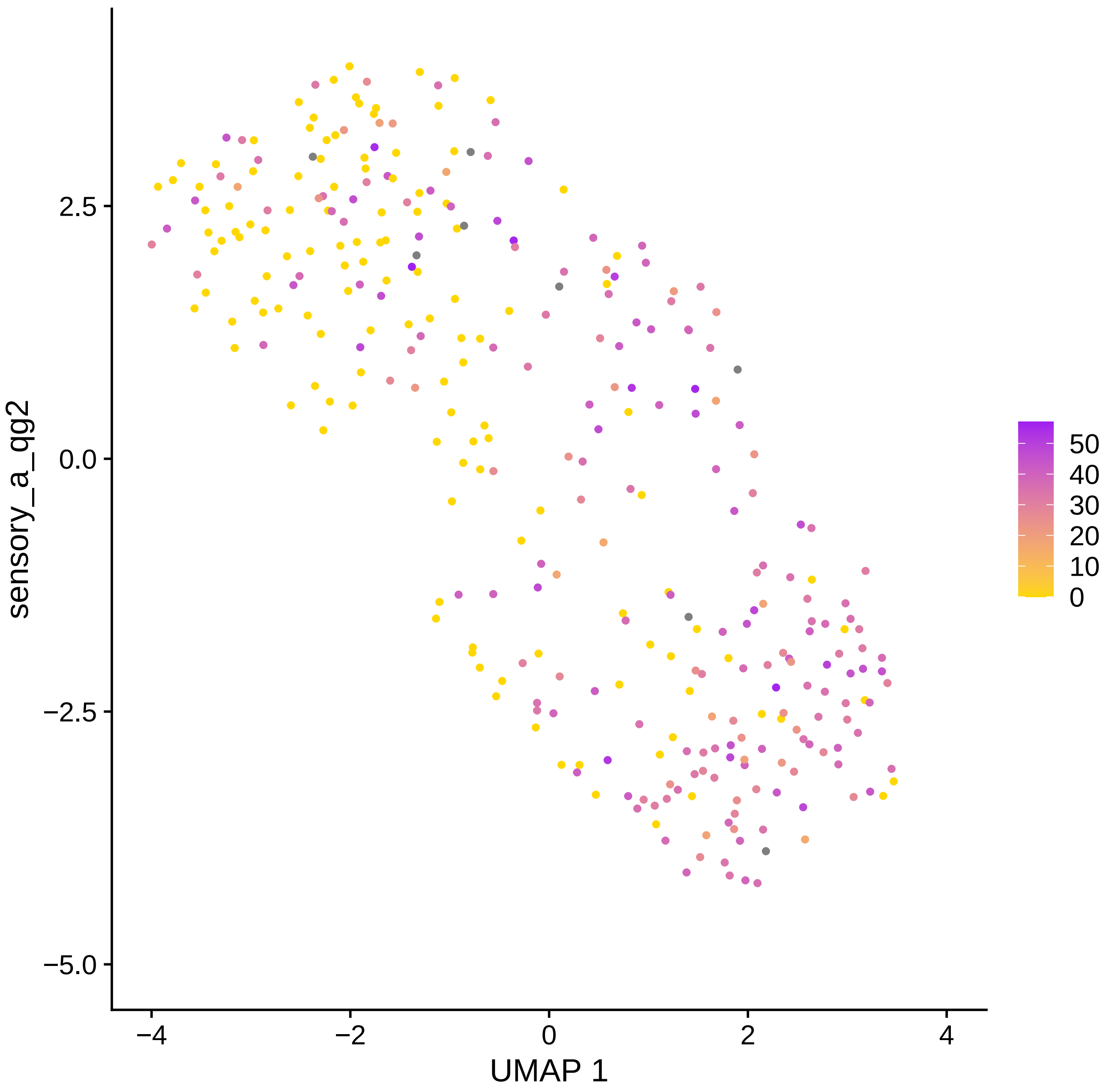

sensory_a_qg2
UMAP 1
2.5
0.0
−2.5
−5.0
−4
−2
0
2
4
50
40
30
20
10
0

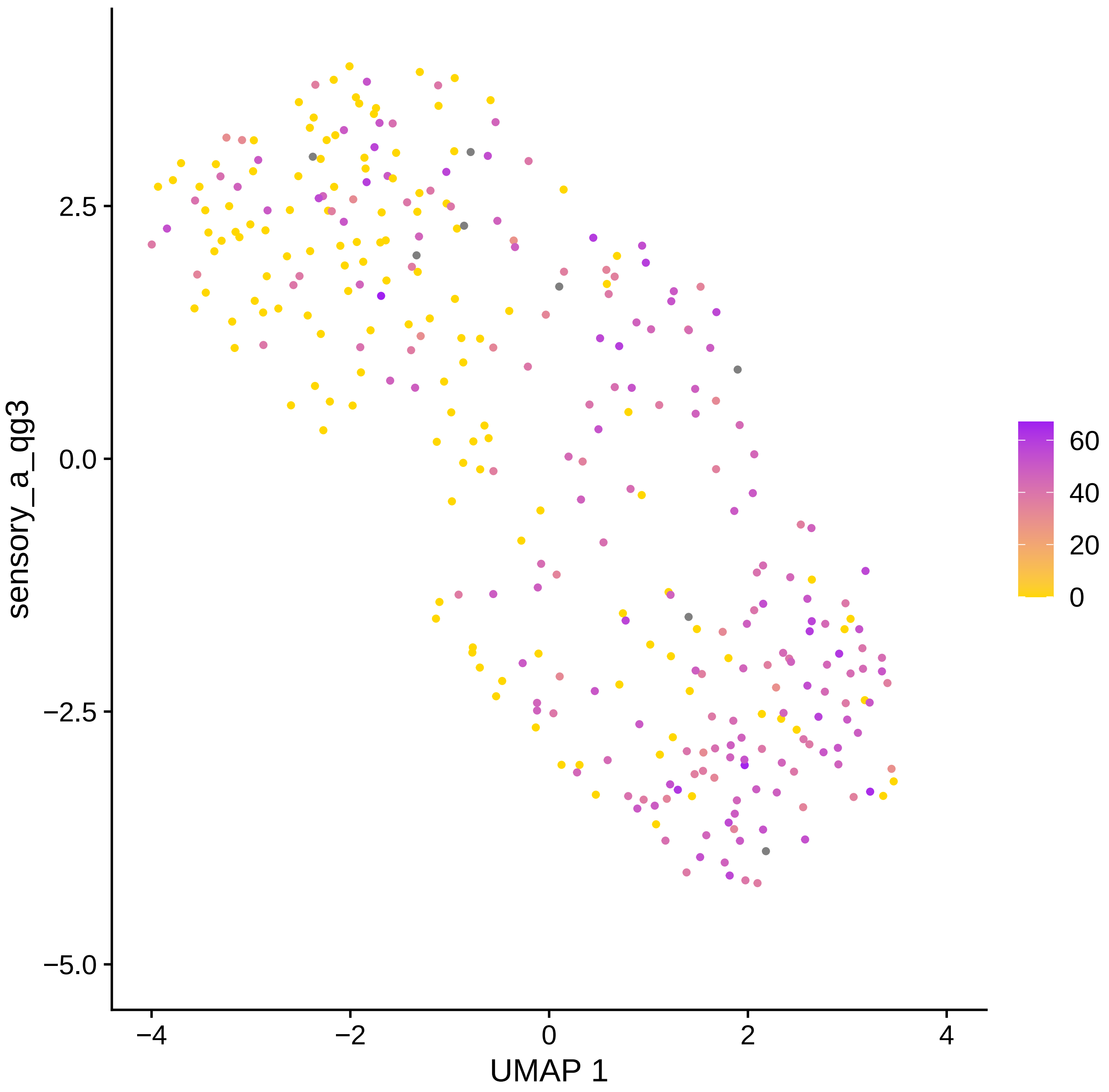
sensory_a_qg3
UMAP 1
2.5
0.0
−2.5
−5.0
−4
−2
0
2
4
60
40
20
0

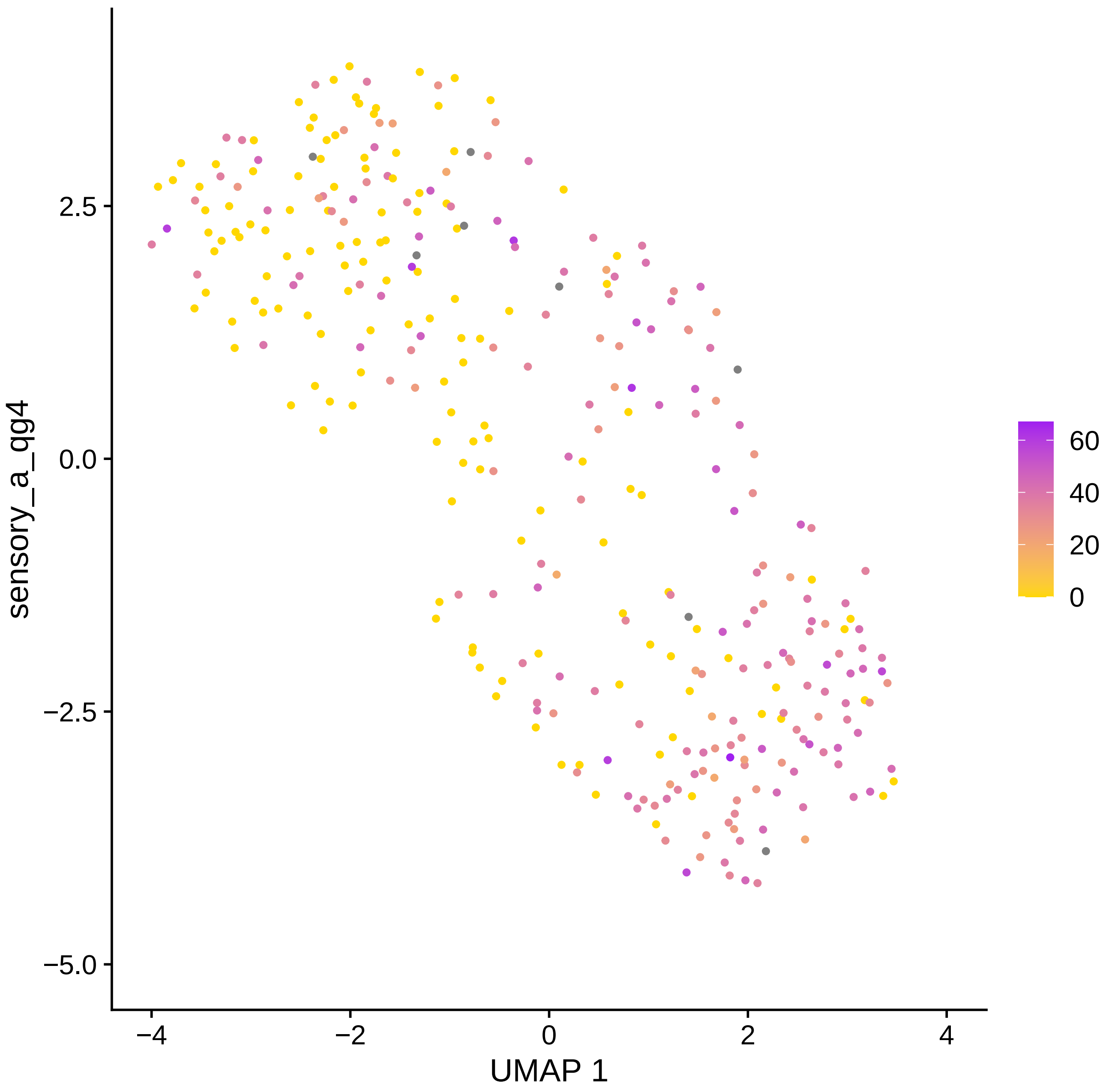

sensory_a_qg4
2.5
0.0
−2.5
−5.0
−4
−2
0
2
4
UMAP 1
60
40
20
0

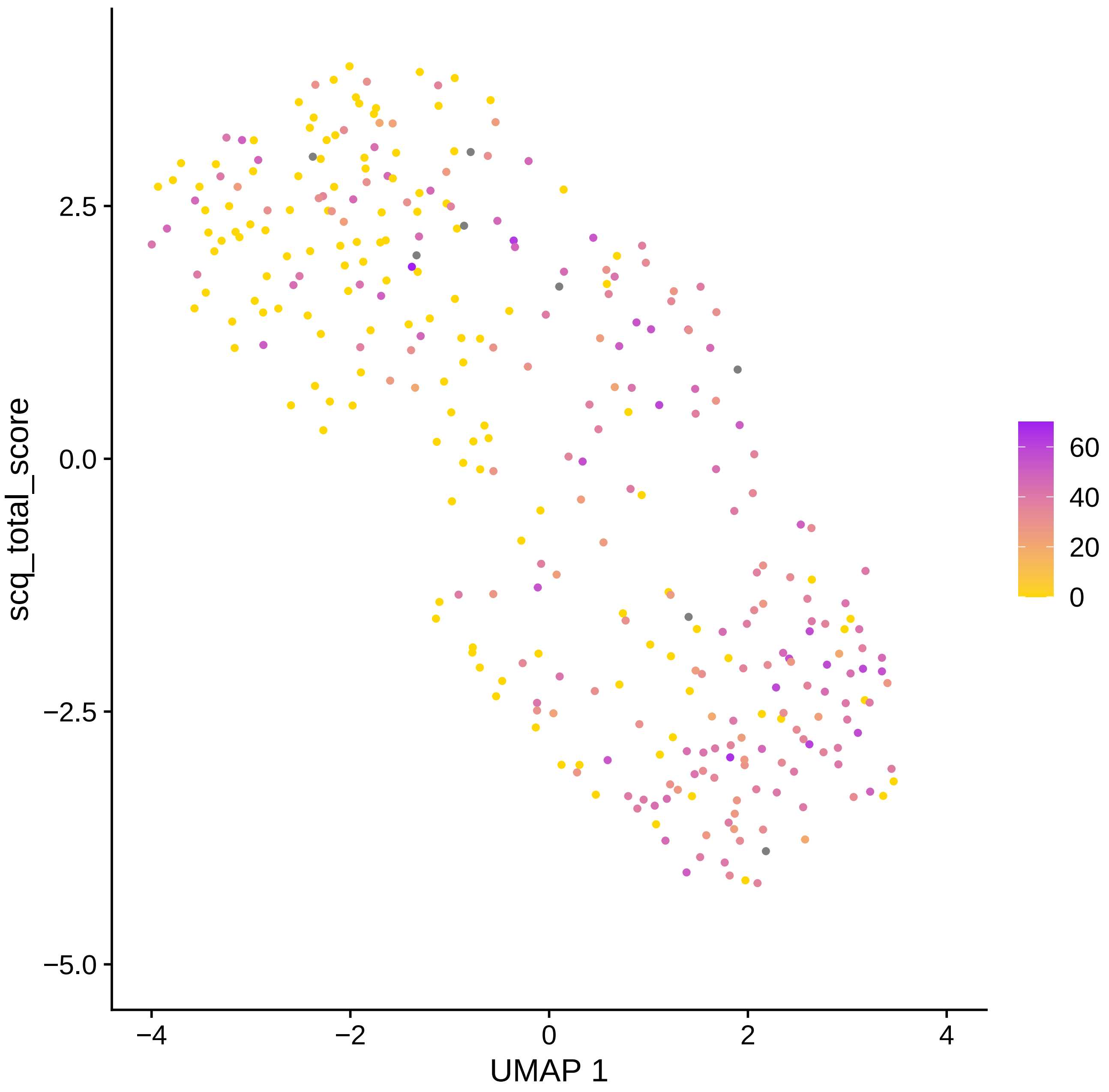
scq_total_score
2.5
0.0
−2.5
−5.0
−4
−2
0
2
4
UMAP 1
60
40
20
0

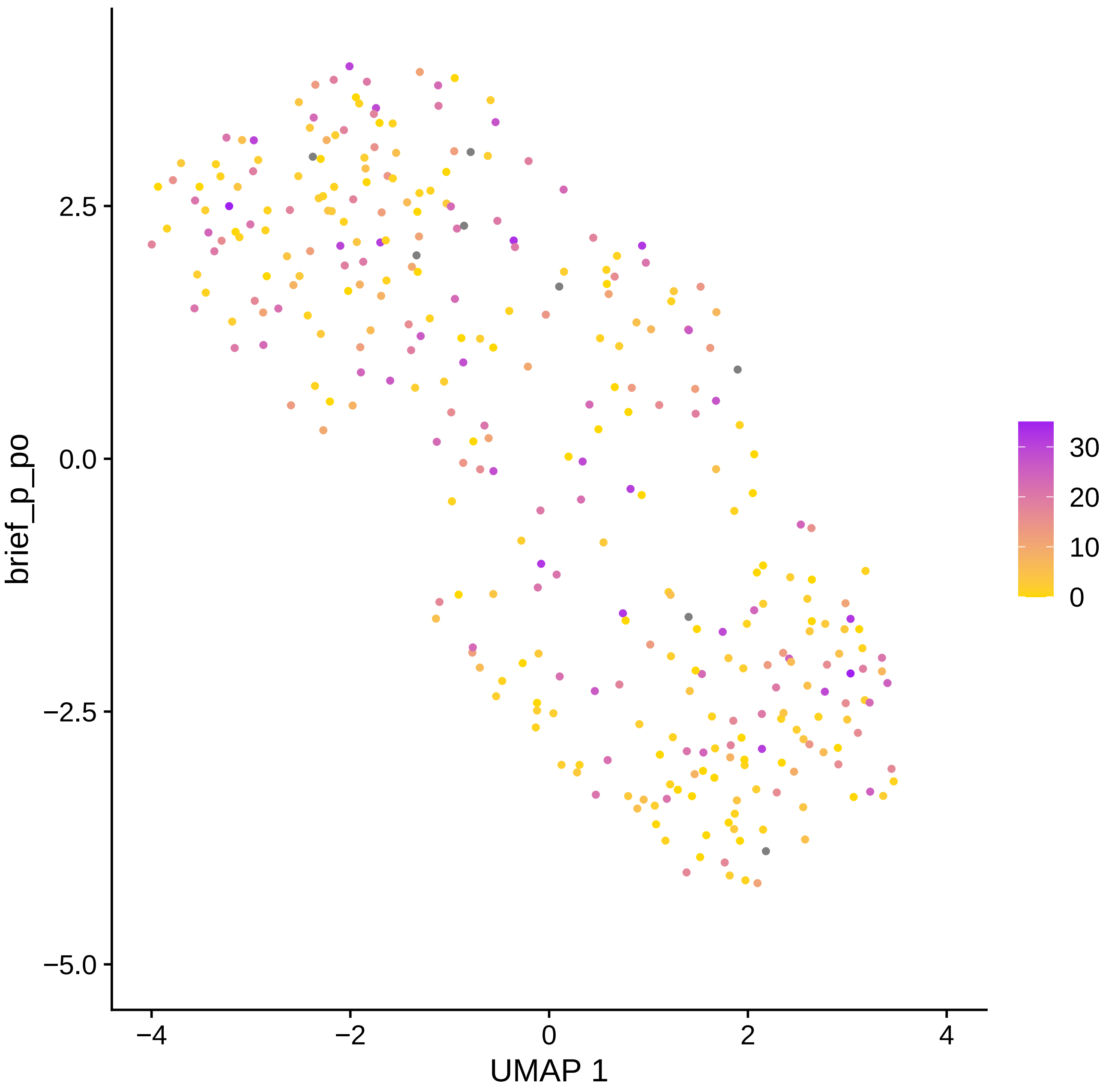
brief_p_po
2.5
0.0
−2.5
−5.0
−4
−2
0
2
4
UMAP 1
30
20
10
0

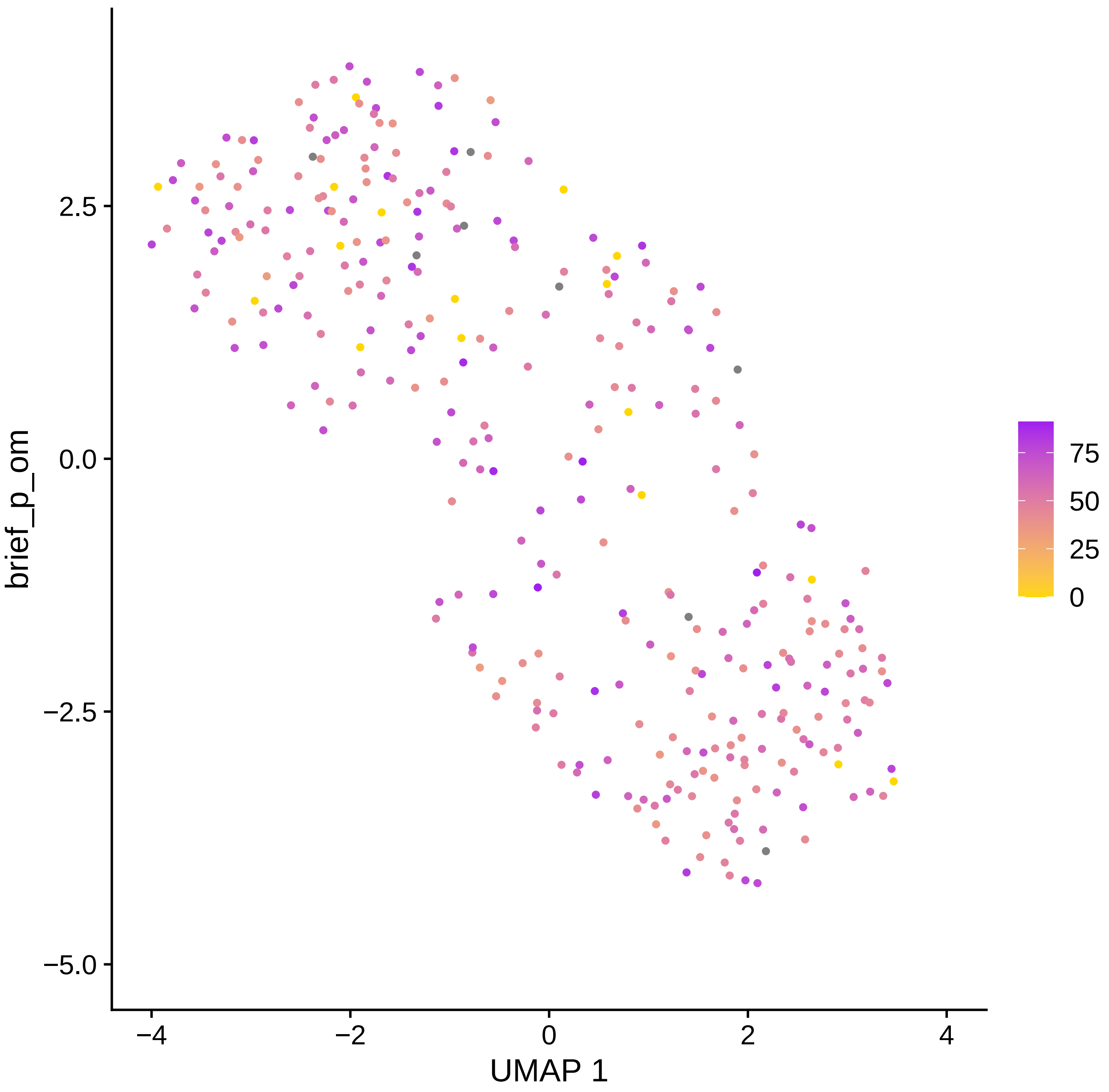
brief_p_om
UMAP 1
2.5
0.0
−2.5
−5.0
−4
−2
0
2
4
75
50
25
0

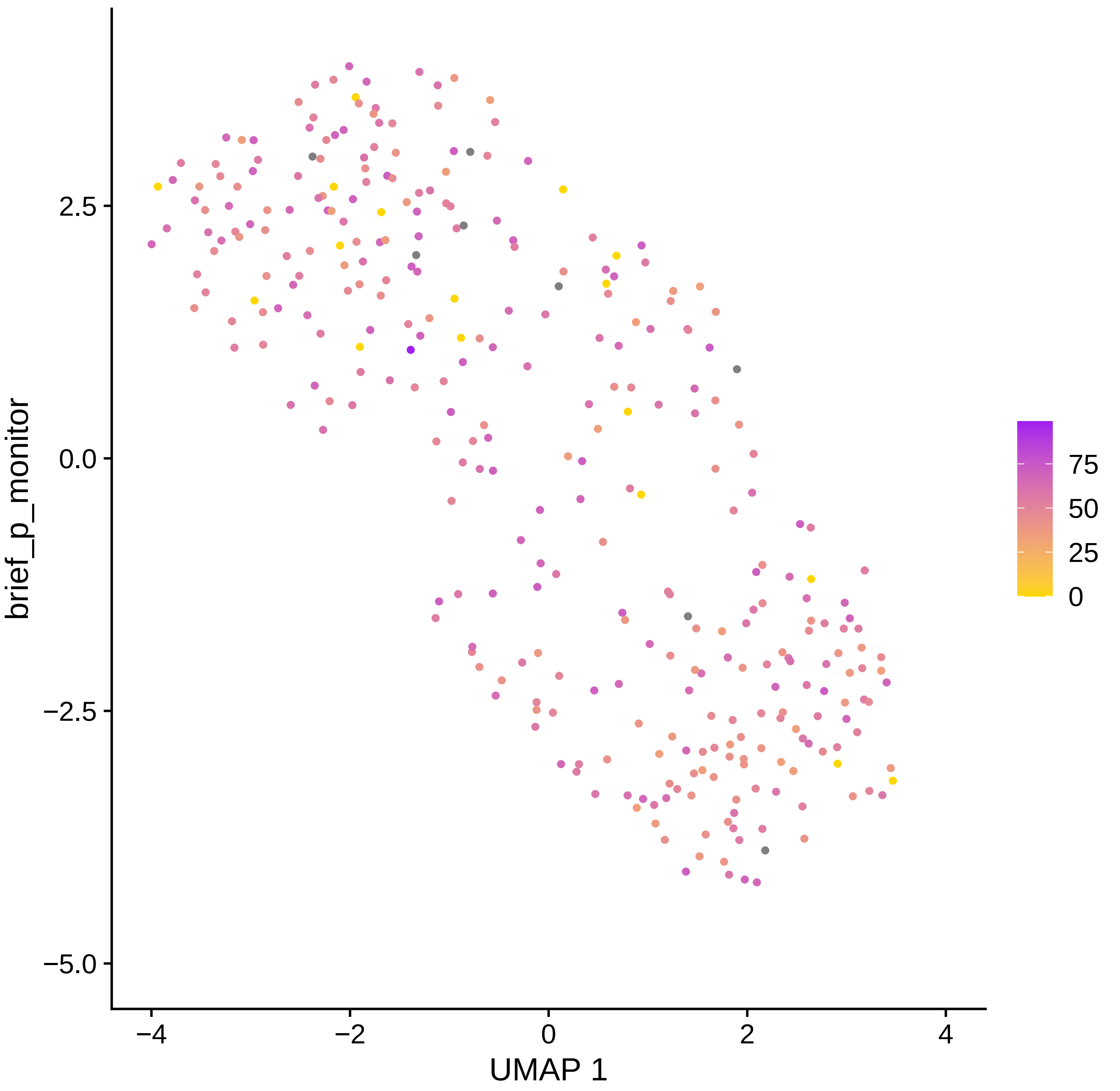
brief_p_monitor
UMAP 1
2.5
0.0
−2.5
−5.0
−4
−2
0
2
4
75
50
25
0

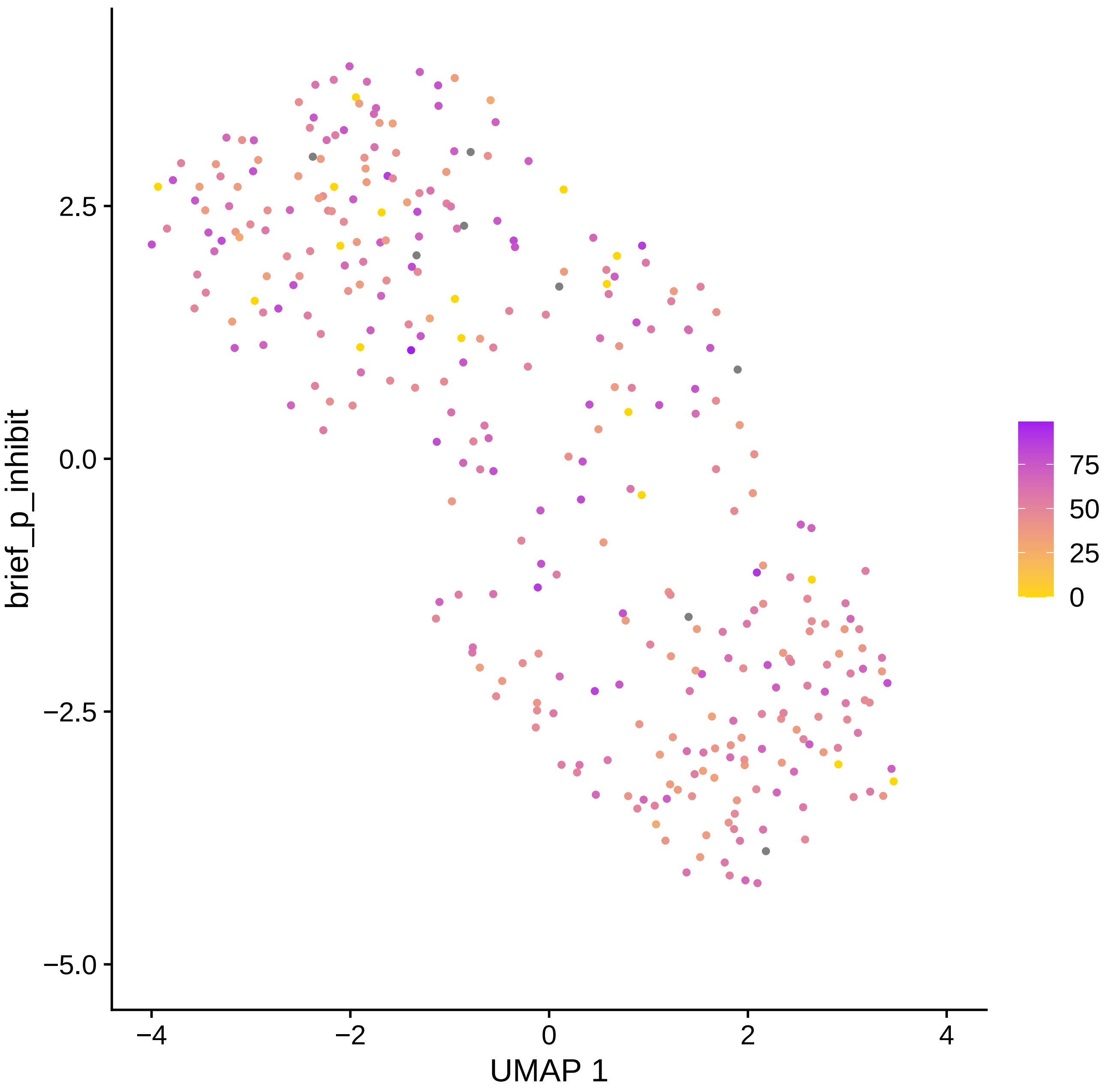

brief_p_inhibit
2.5
0.0
−2.5
−5.0
−4
−2
0
2
4
UMAP 1
75
50
25
0

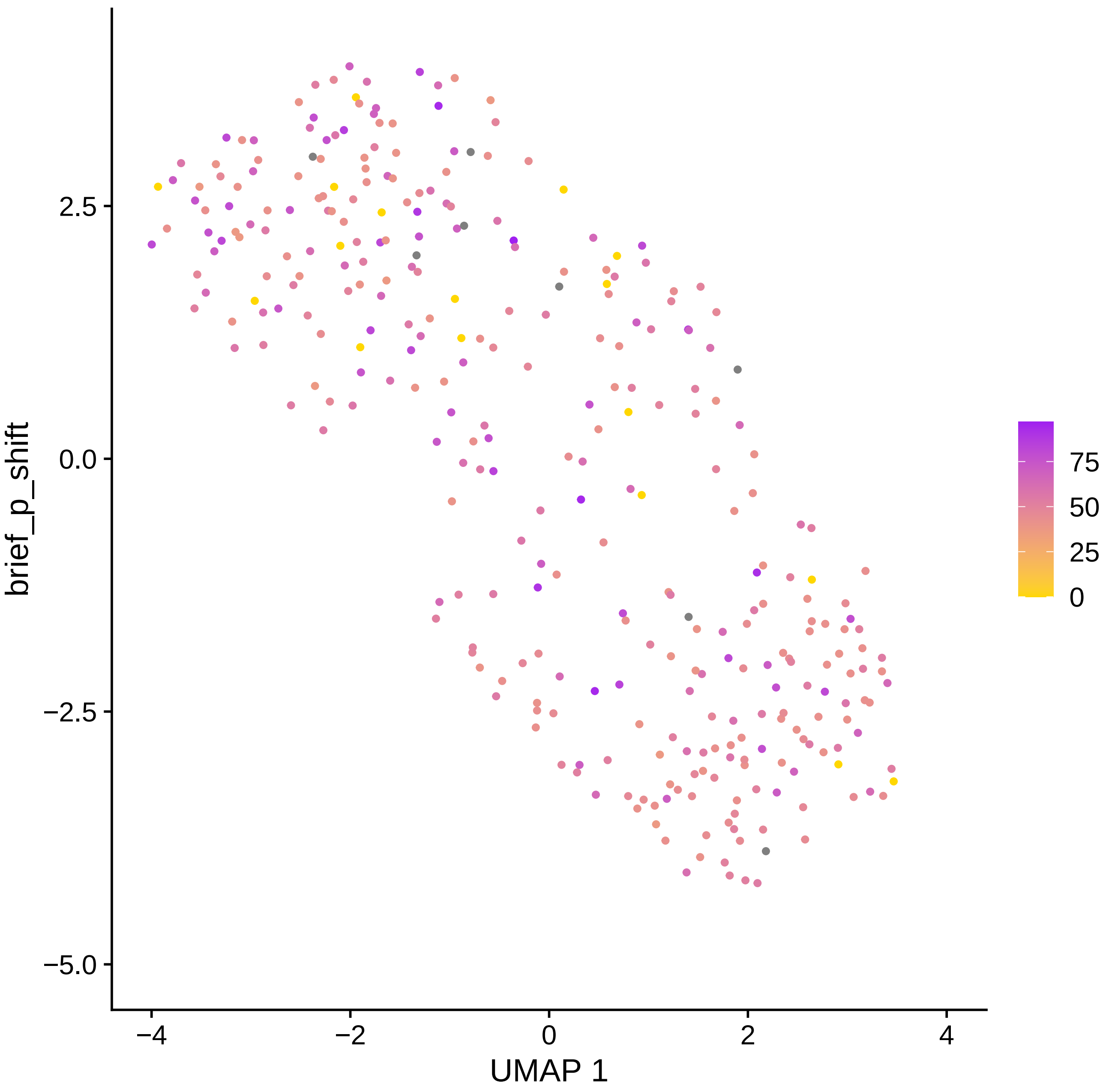

brief_p_shift
UMAP 1
2.5
0.0
−2.5
−5.0
−4
−2
0
2
4
75
50
25
0

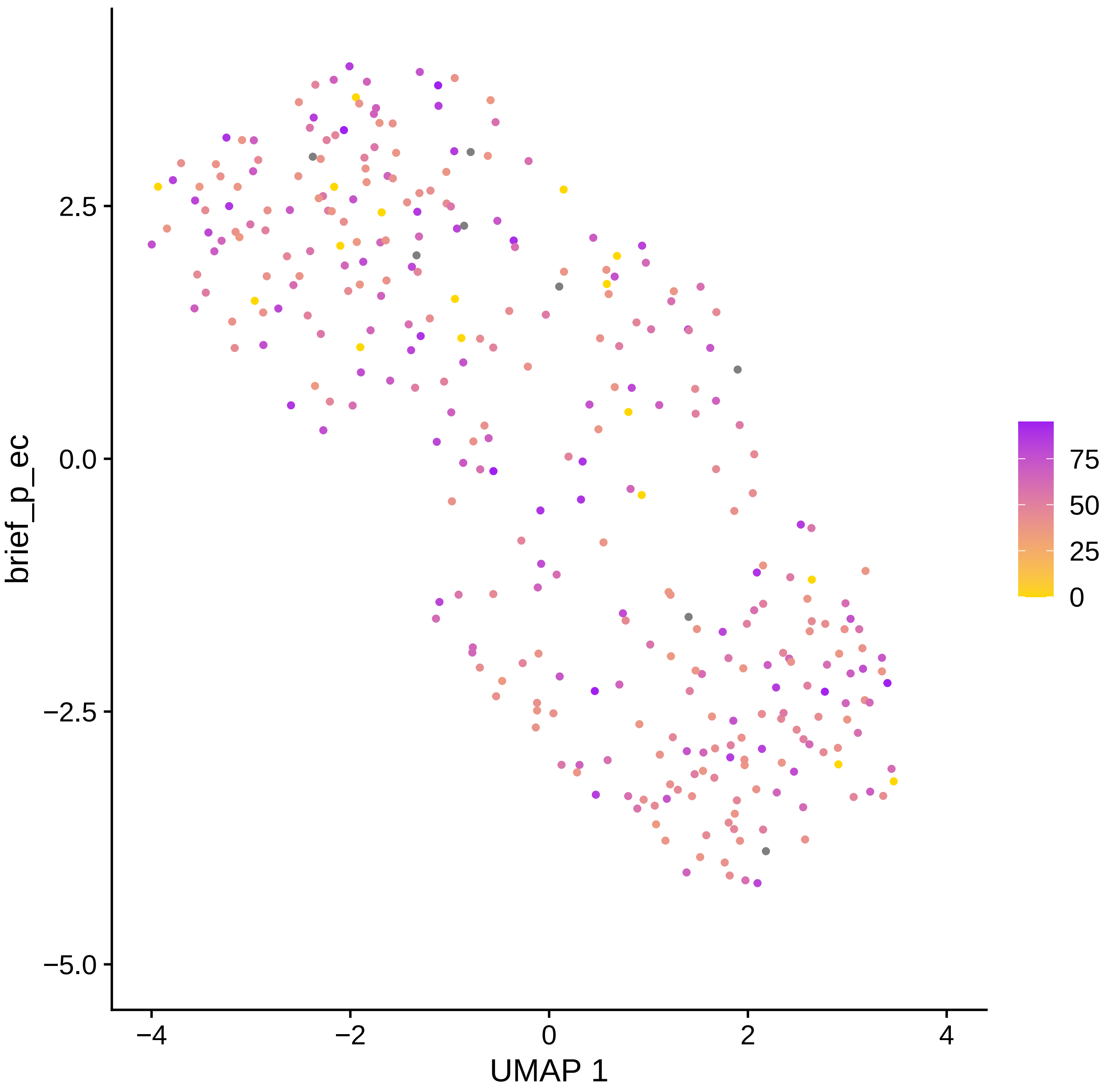

brief_p_ec
2.5
0.0
−2.5
−5.0
−4
−2
0
2
4
UMAP 1
75
50
25
0

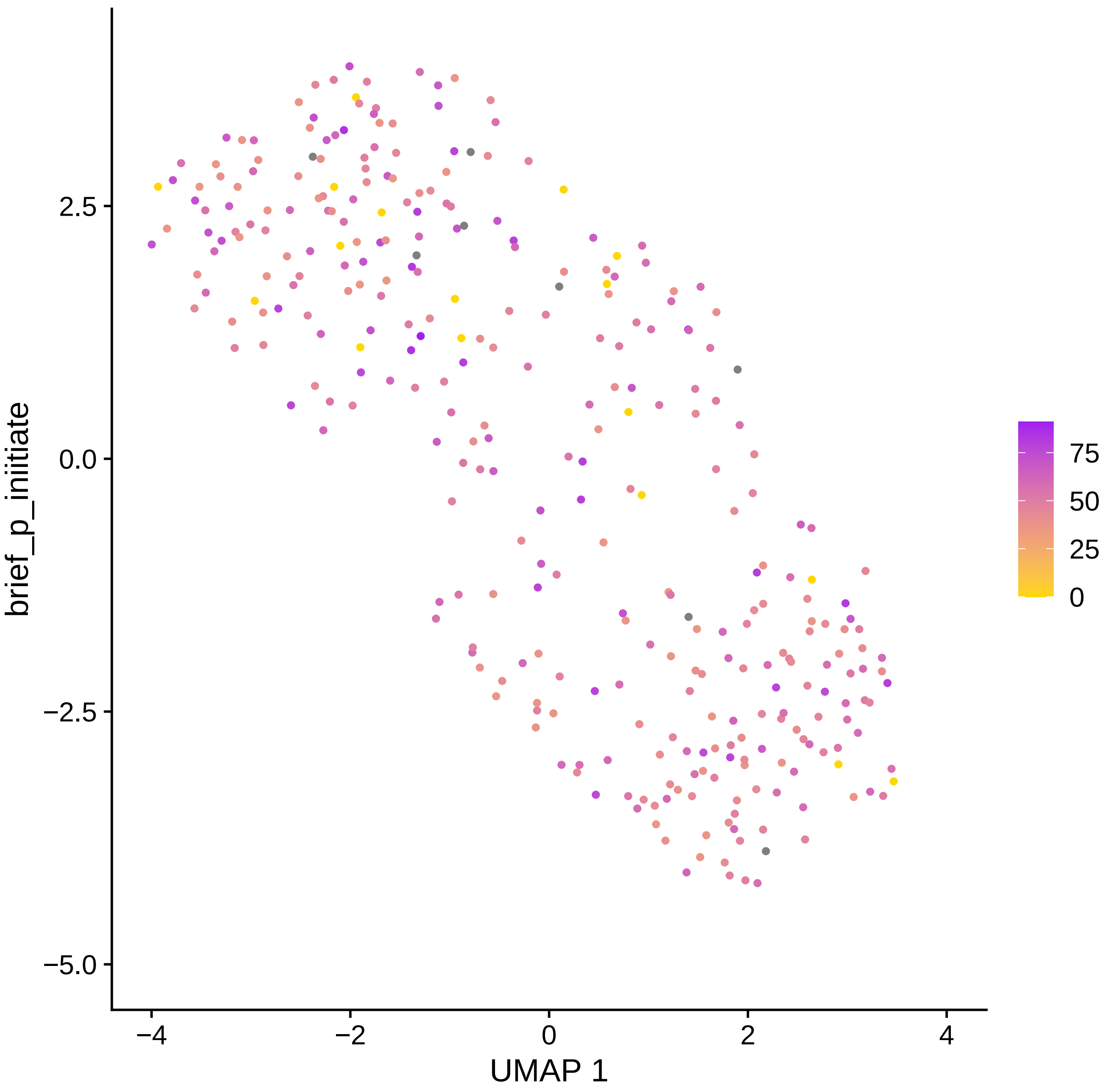
brief_p_initiate
2.5
0.0
−2.5
−5.0
−4
−2
0
2
4
UMAP 1
75
50
25
0

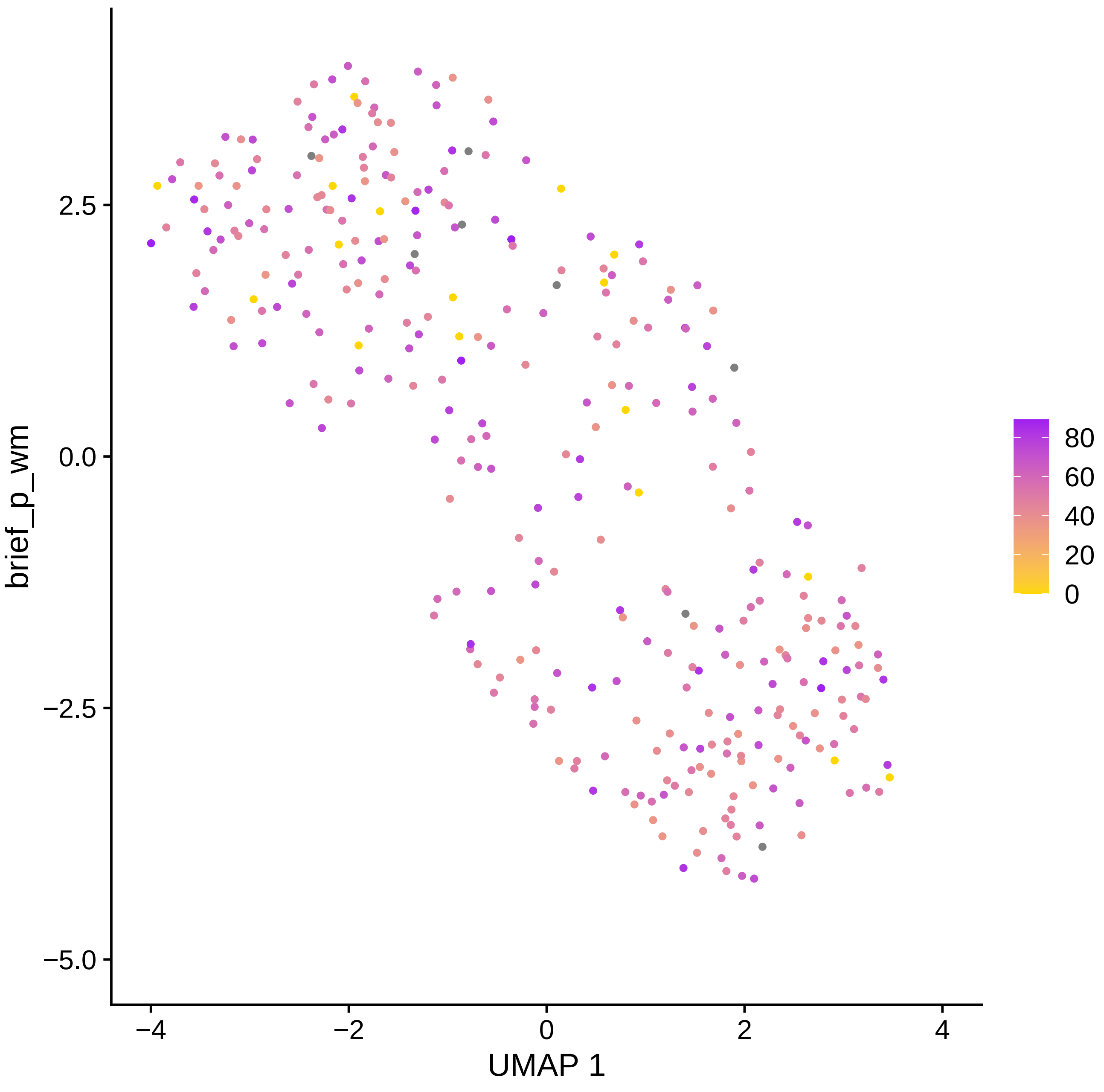
brief_p_wm
UMAP 1
2.5
0.0
−2.5
−5.0
−4
−2
0
2
4
80
60
40
20
0

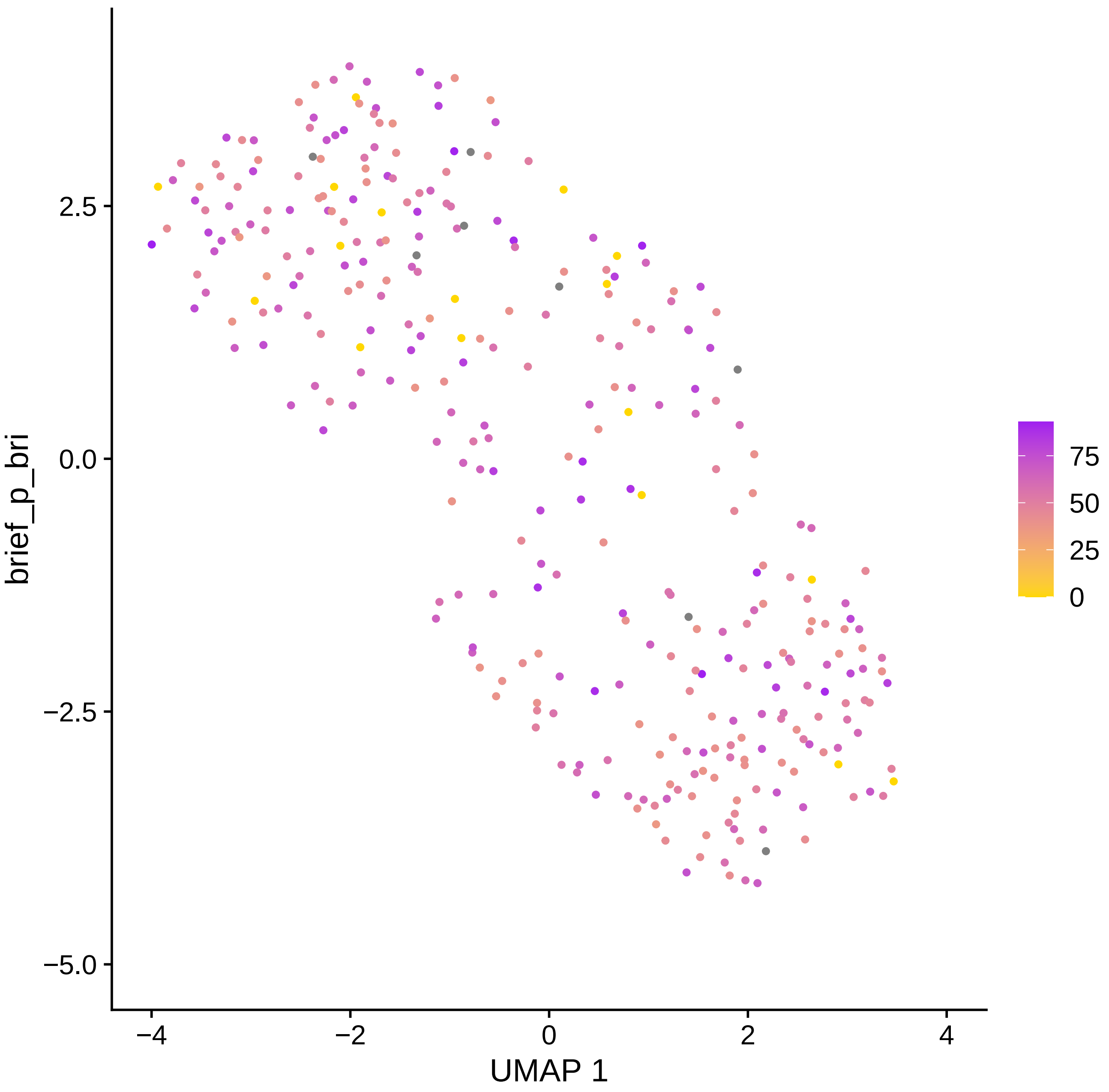
brief_p_bri
UMAP 1
2.5
0.0
−2.5
−5.0
−4
−2
0
2
4
75
50
25
0

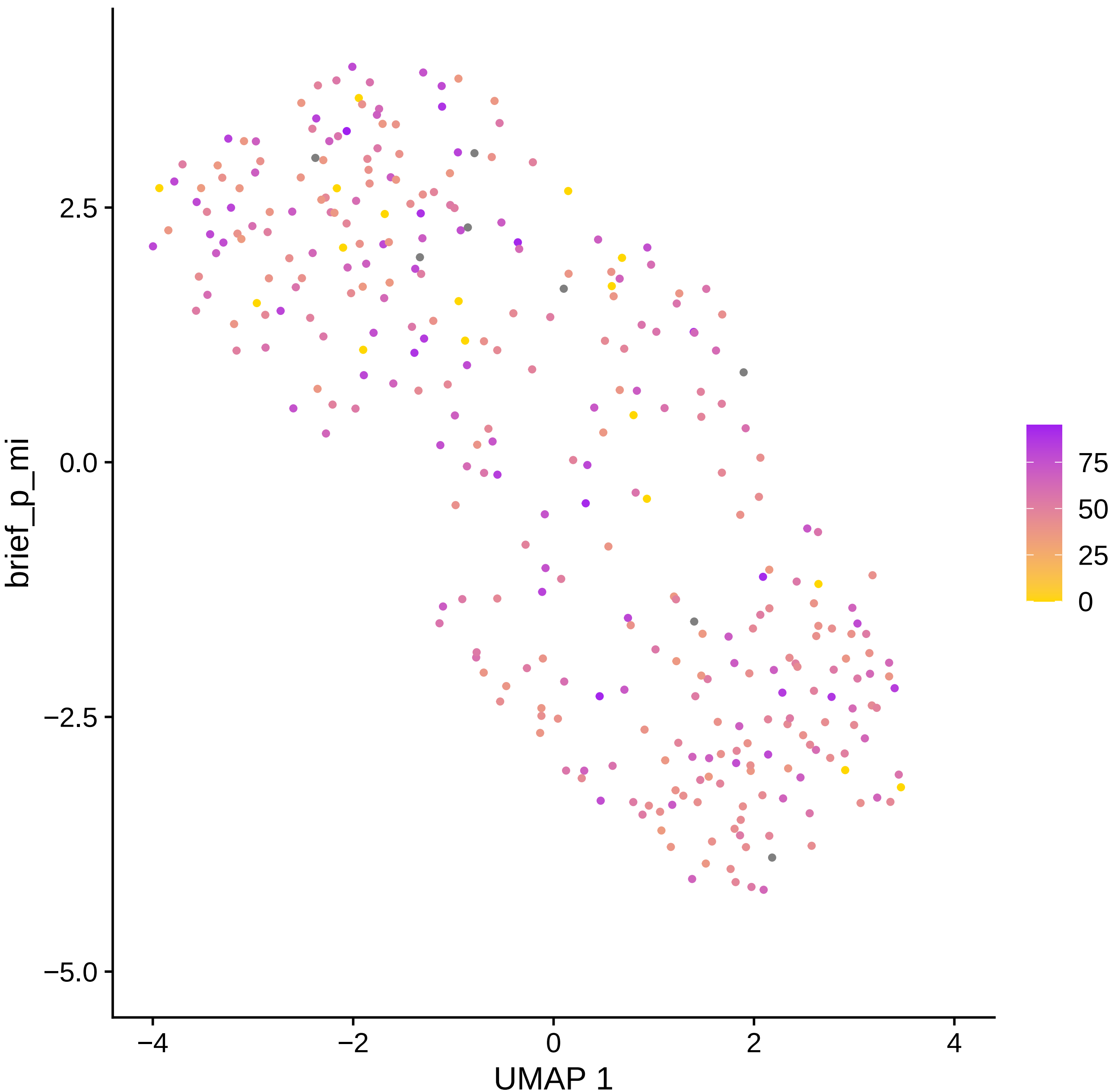
brief_p_mi
UMAP 1
2.5
0.0
−2.5
−5.0
−4
−2
0
2
4
75
50
25
0

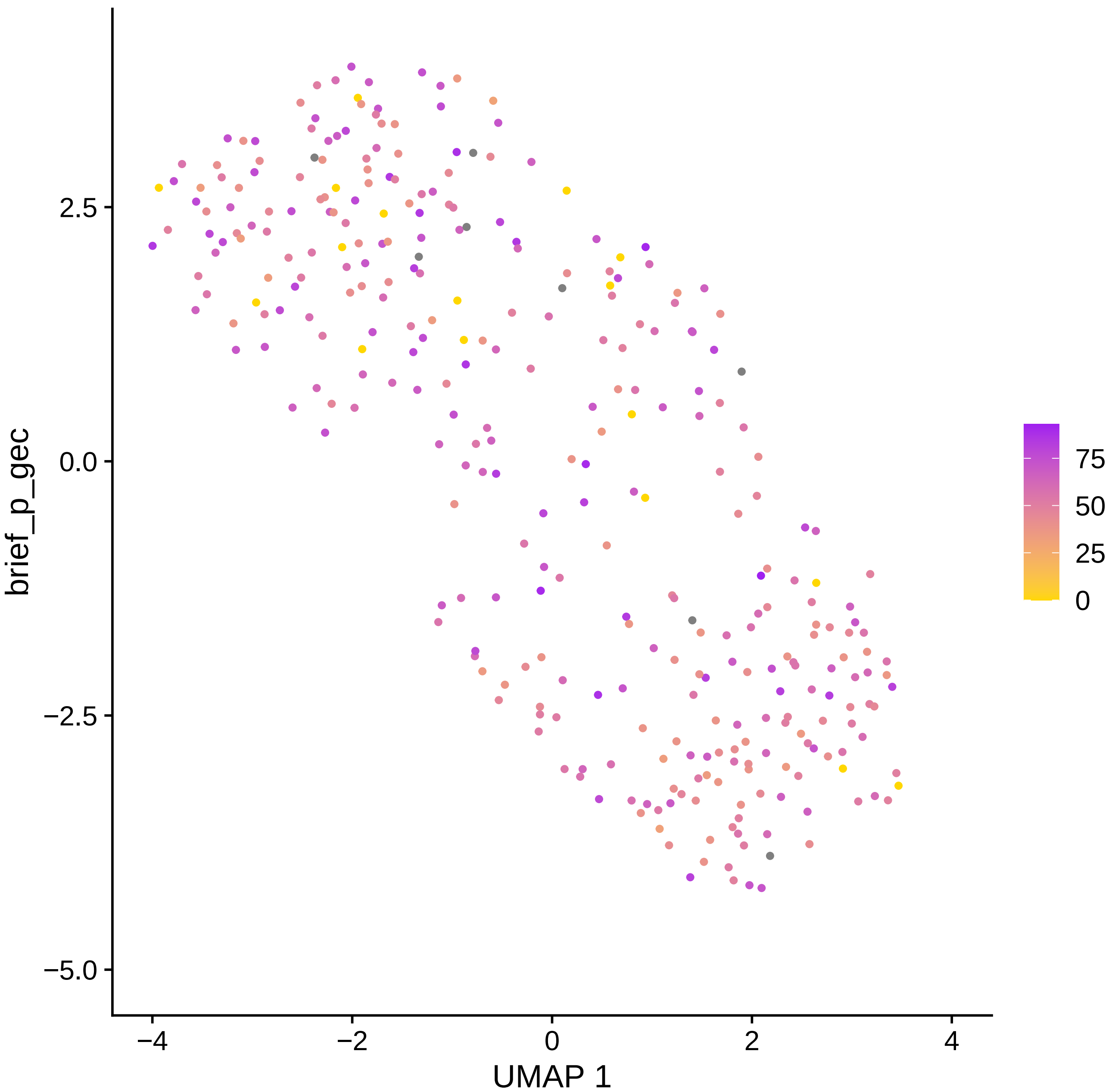

brief_p_gec
2.5
0.0
−2.5
−5.0
−4
−2
0
2
4
UMAP 1
75
50
25
0

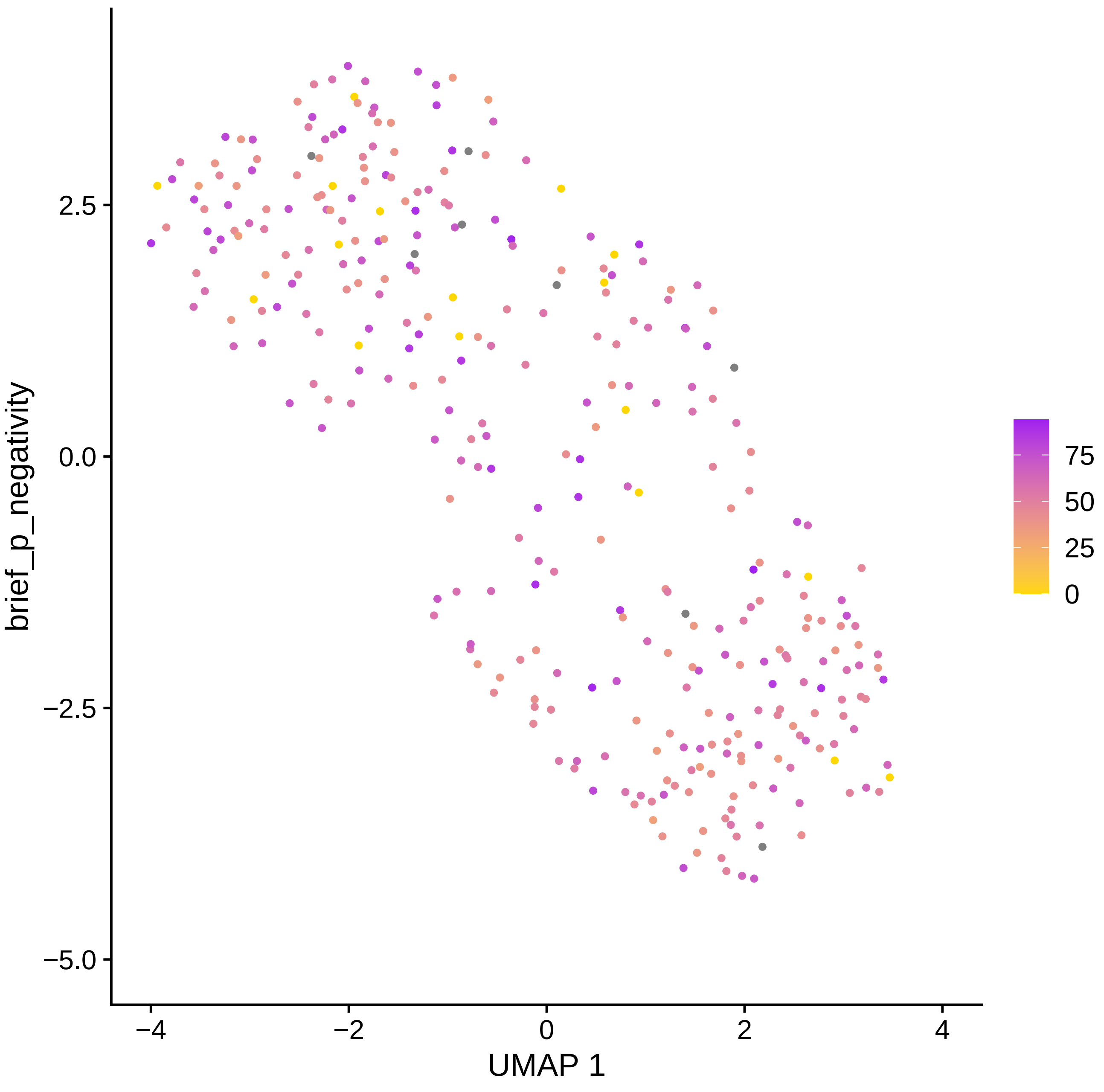

brief_p_negativity
UMAP 1
2.5
0.0
−2.5
−5.0
−4
−2
0
2
4
75
50
25
0

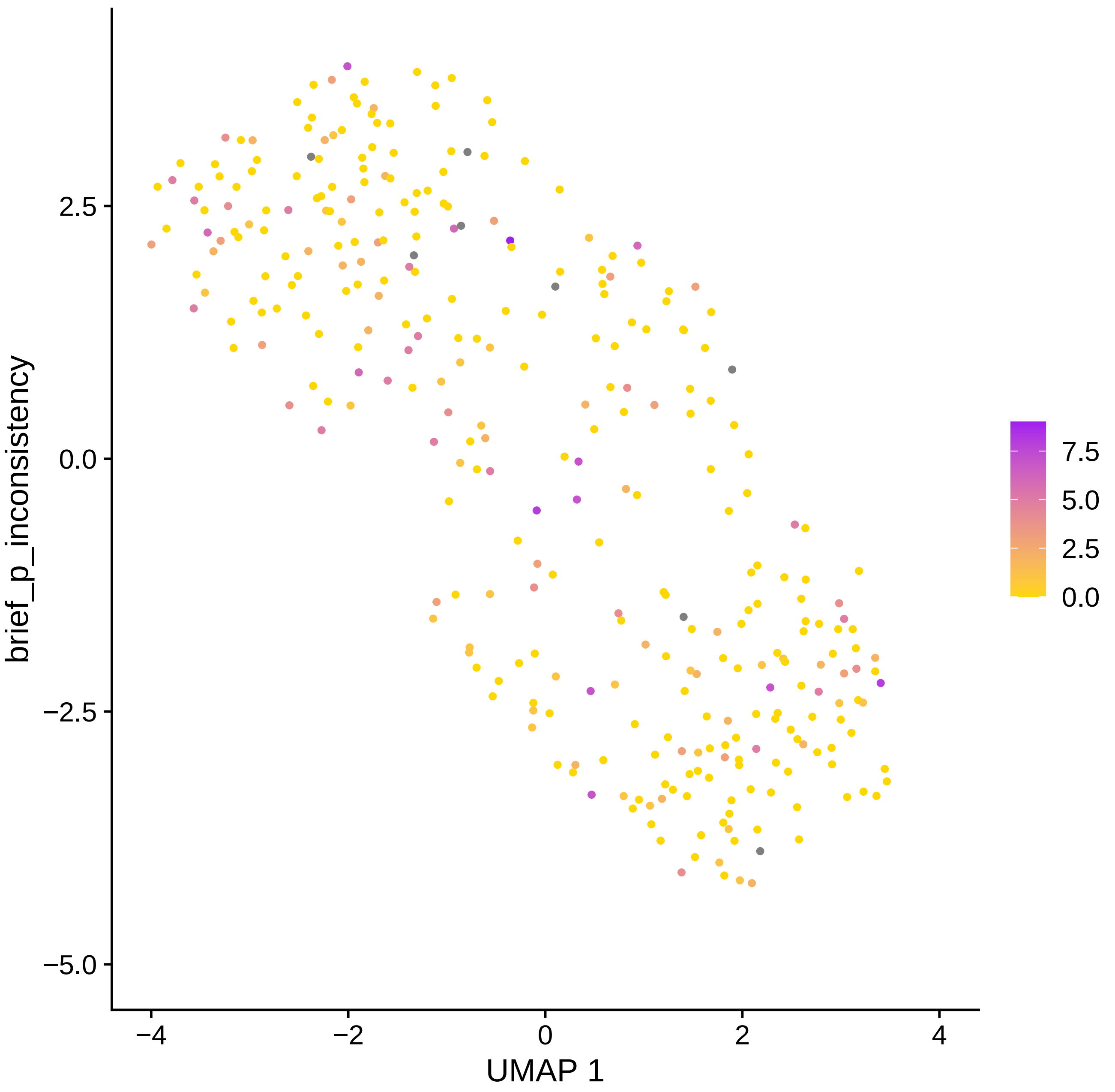
brief_p_inconsistency
UMAP 1
2.5
0.0
−2.5
−5.0
−4
−2
0
2
4
7.5
5.0
2.5
0.0

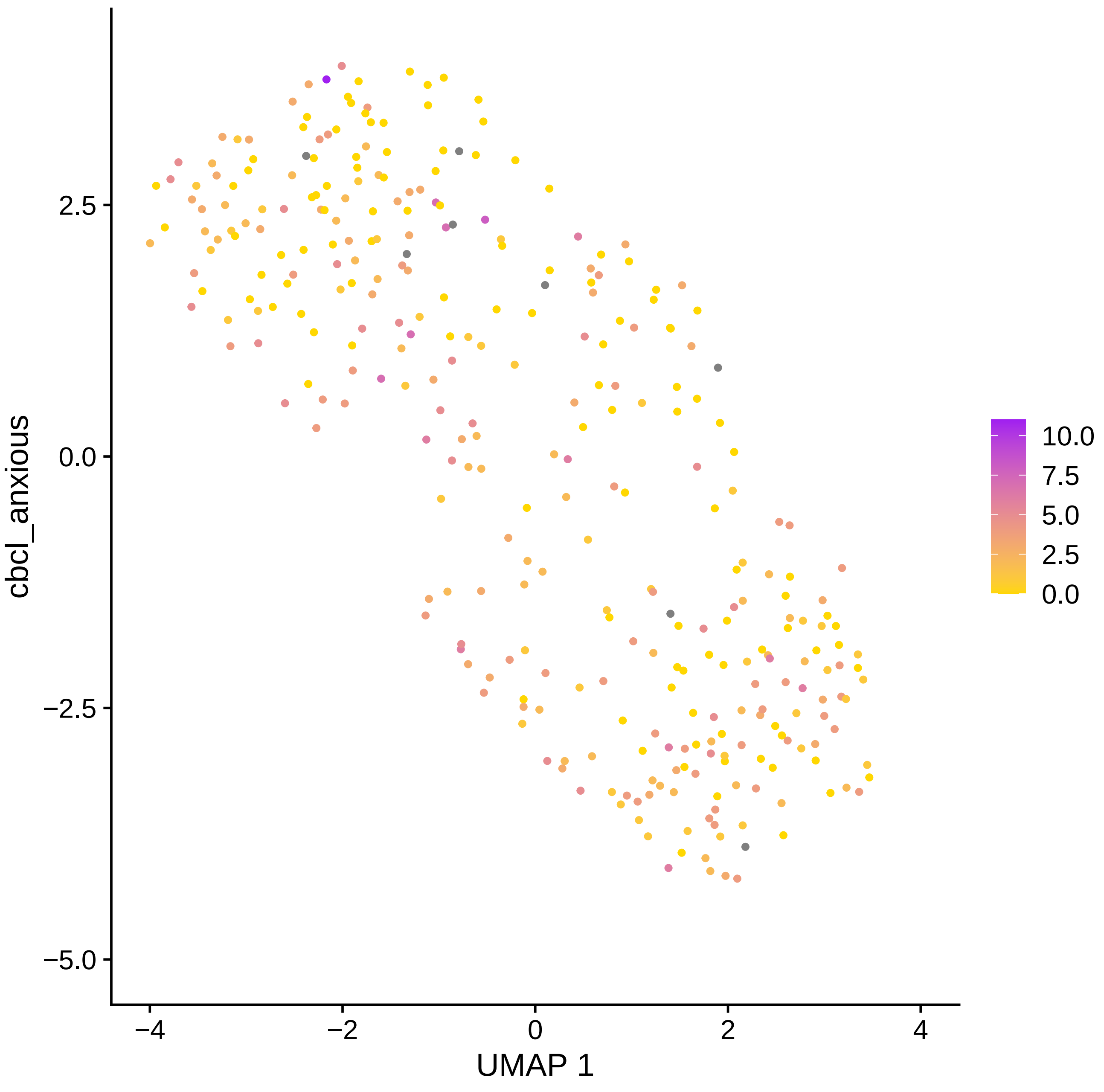
cbcl_anxious
2.5
0.0
−2.5
−5.0
−4
−2
0
2
4
UMAP 1
10.0
7.5
5.0
2.5
0.0

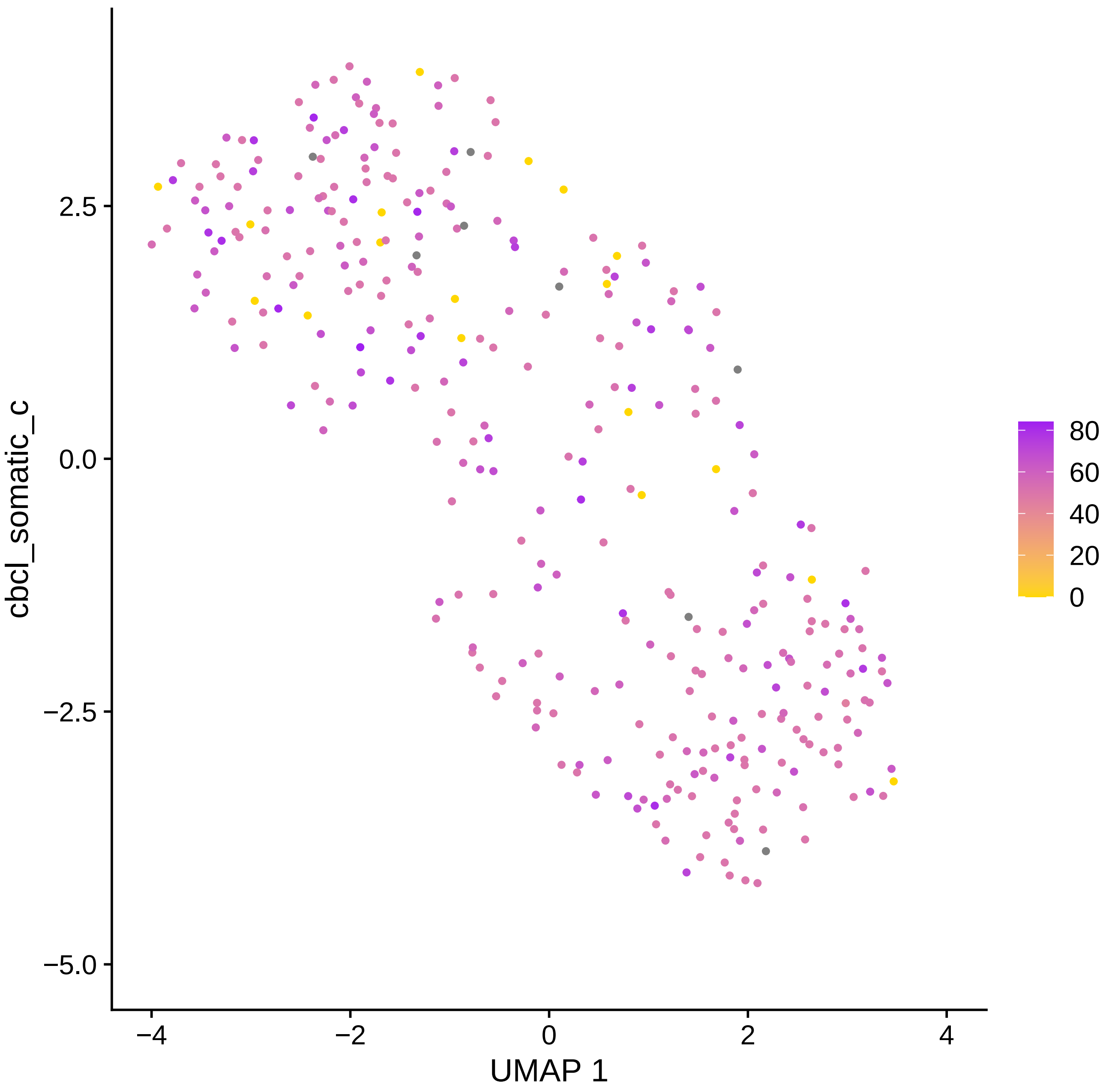

cbcl_somatic_c
UMAP 1
2.5
0.0
−2.5
−5.0
−4
−2
0
2
4
80
60
40
20
0

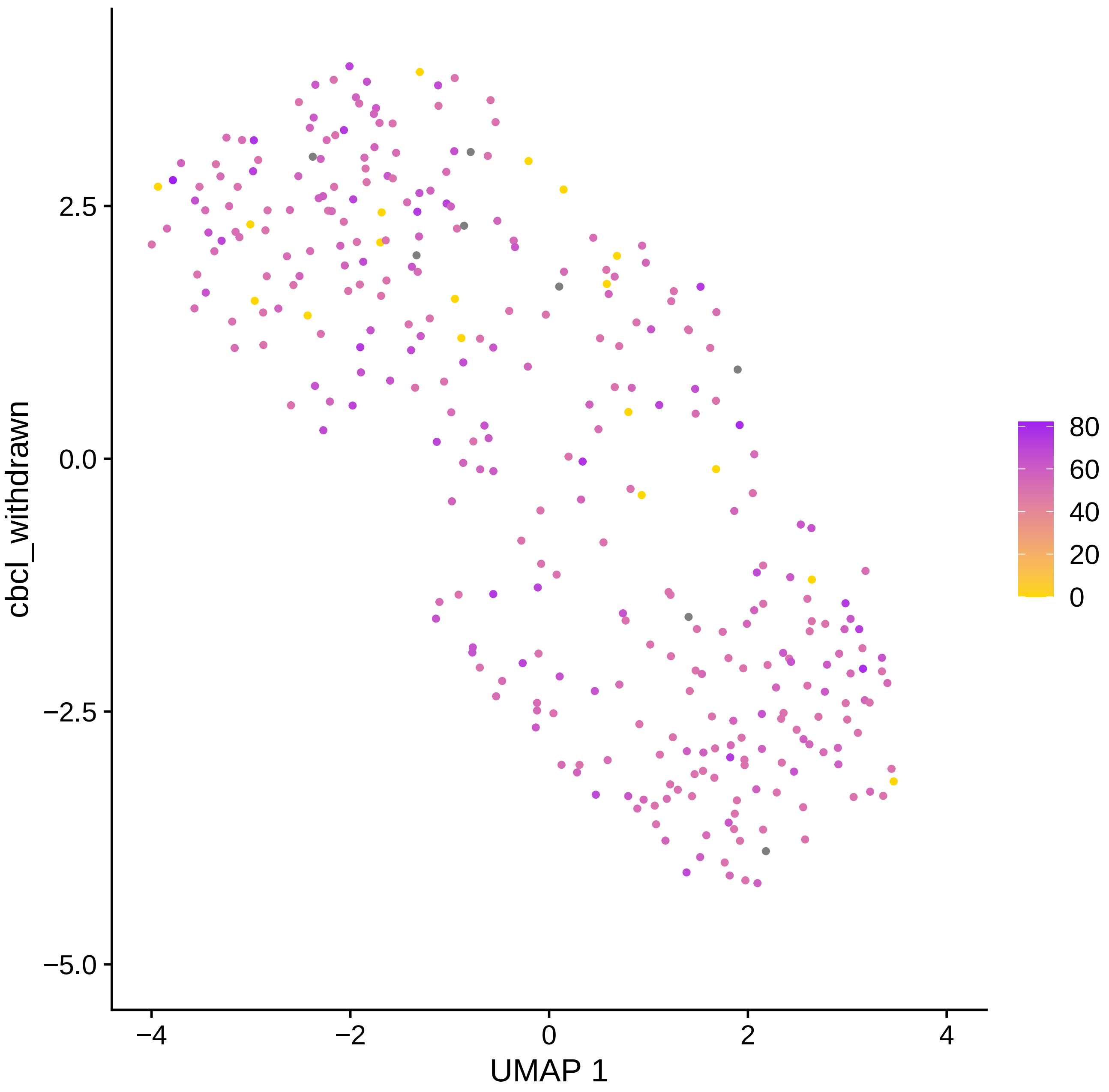

cbcl_withdrawn
UMAP 1
2.5
0.0
−2.5
−5.0
−4
−2
0
2
4
80
60
40
20
0

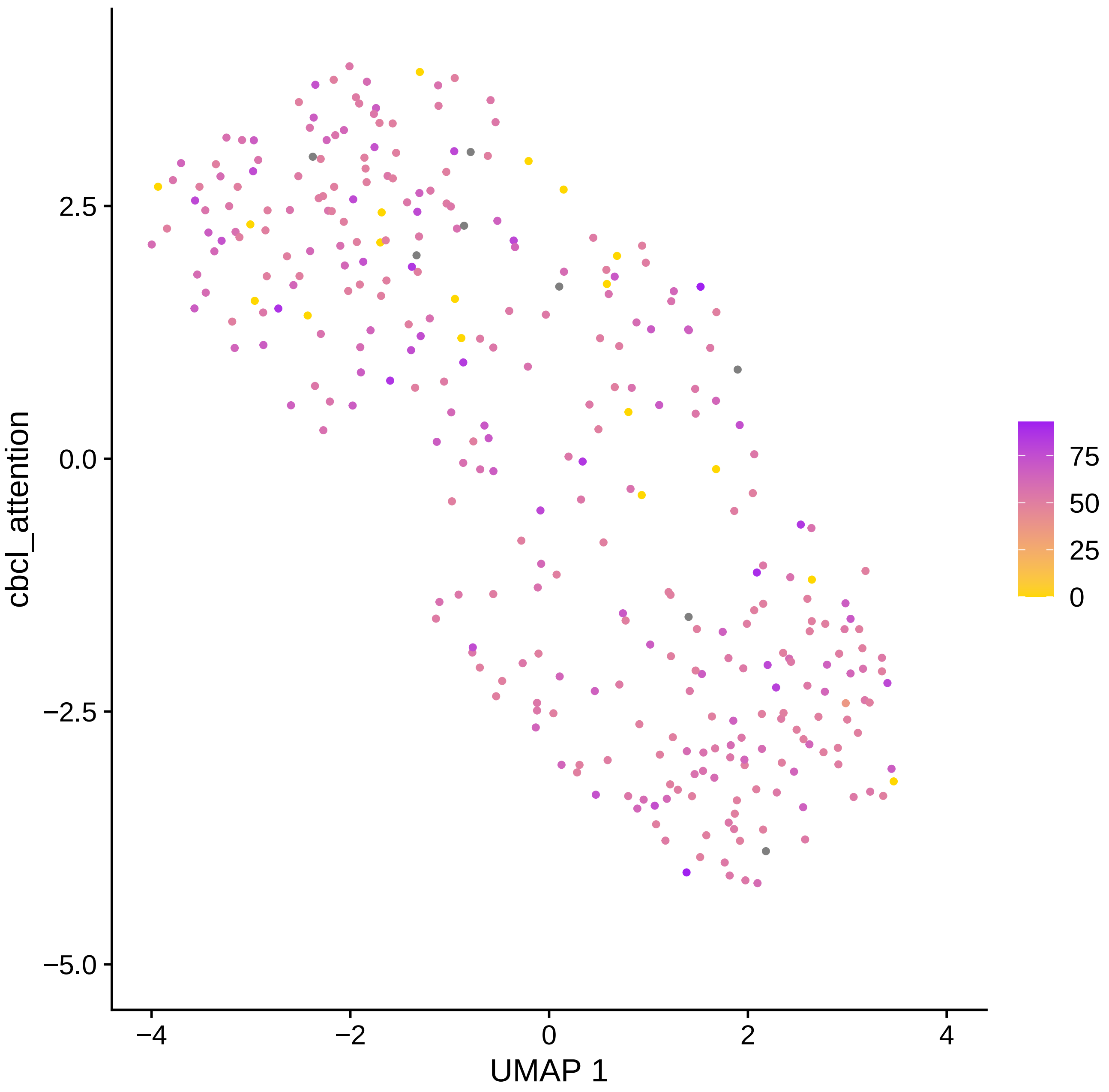

cbcl_attention
2.5
0.0
−2.5
−5.0
−4
−2
0
2
4
UMAP 1
75
50
25
0

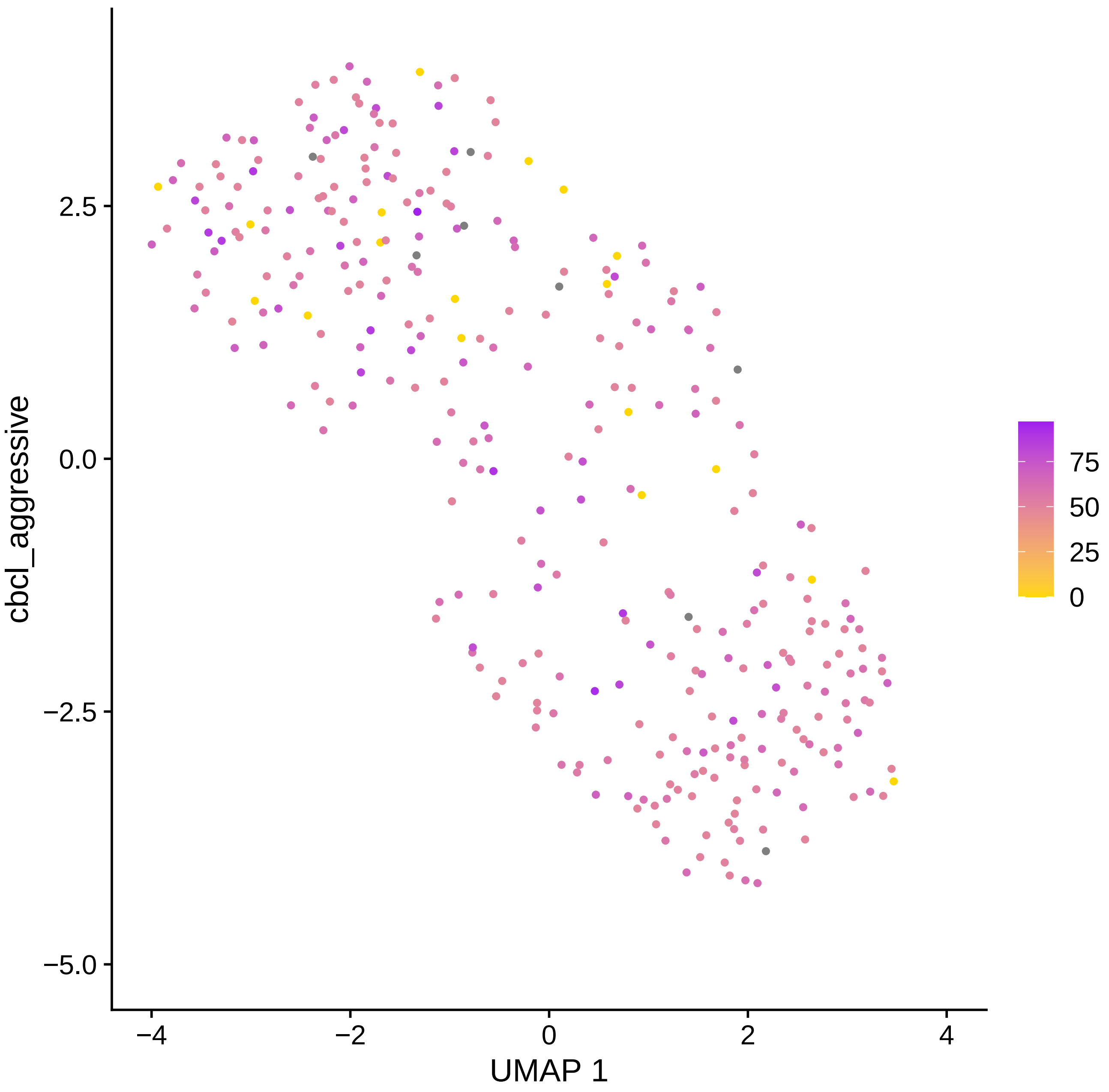

cbcl_aggressive
2.5
0.0
−2.5
−5.0
−4
−2
0
2
4
UMAP 1
75
50
25
0

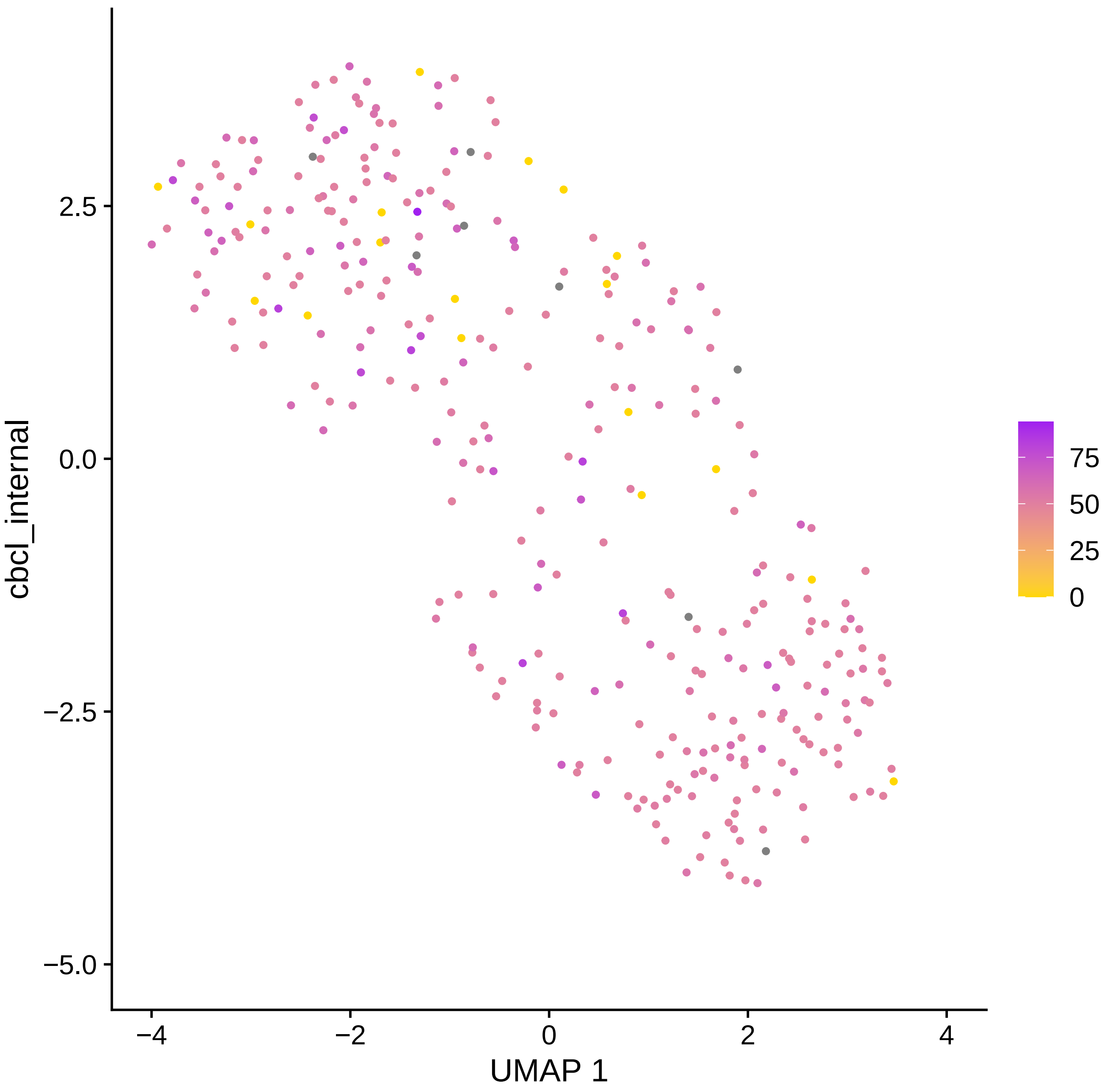

cbcl_internal
2.5
0.0
−2.5
−5.0
−4
−2
0
2
4
UMAP 1
75
50
25
0

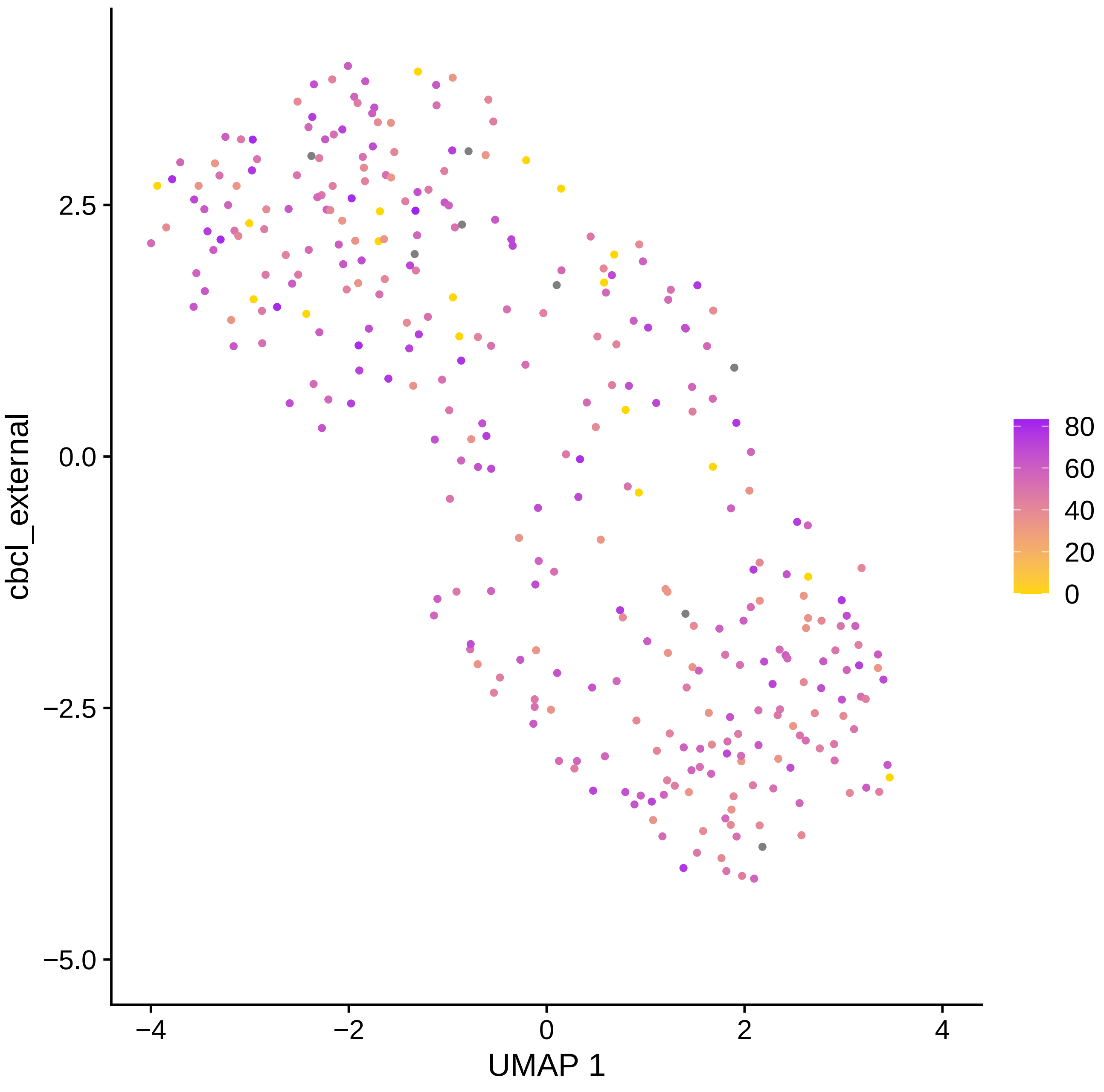

cbcl_external
UMAP 1
2.5
0.0
−2.5
−5.0
−4
−2
0
2
4
80
60
40
20
0

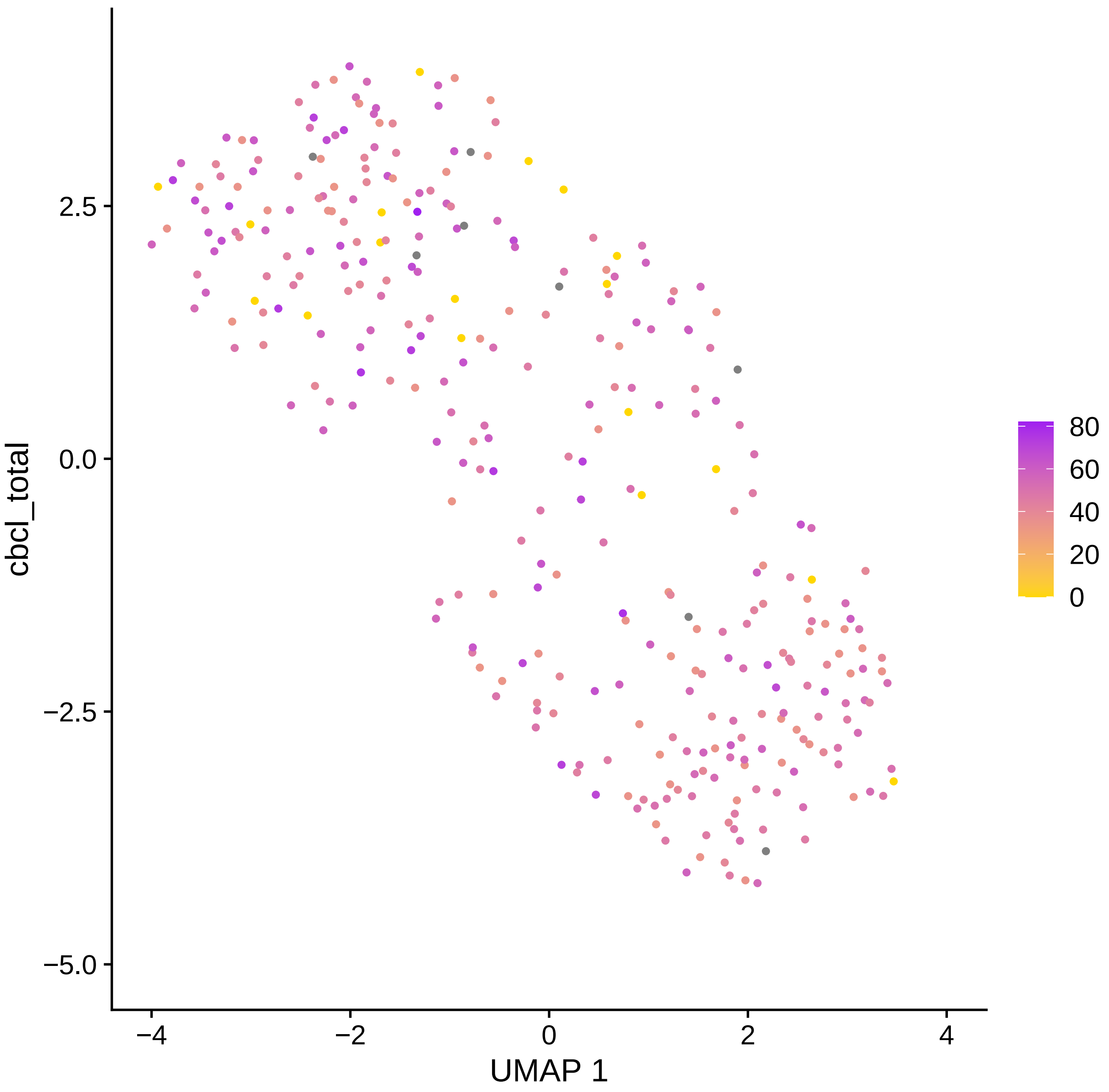
cbcl_total
UMAP 1
2.5
0.0
−2.5
−5.0
−4
−2
0
2
4
80
60
40
20
0

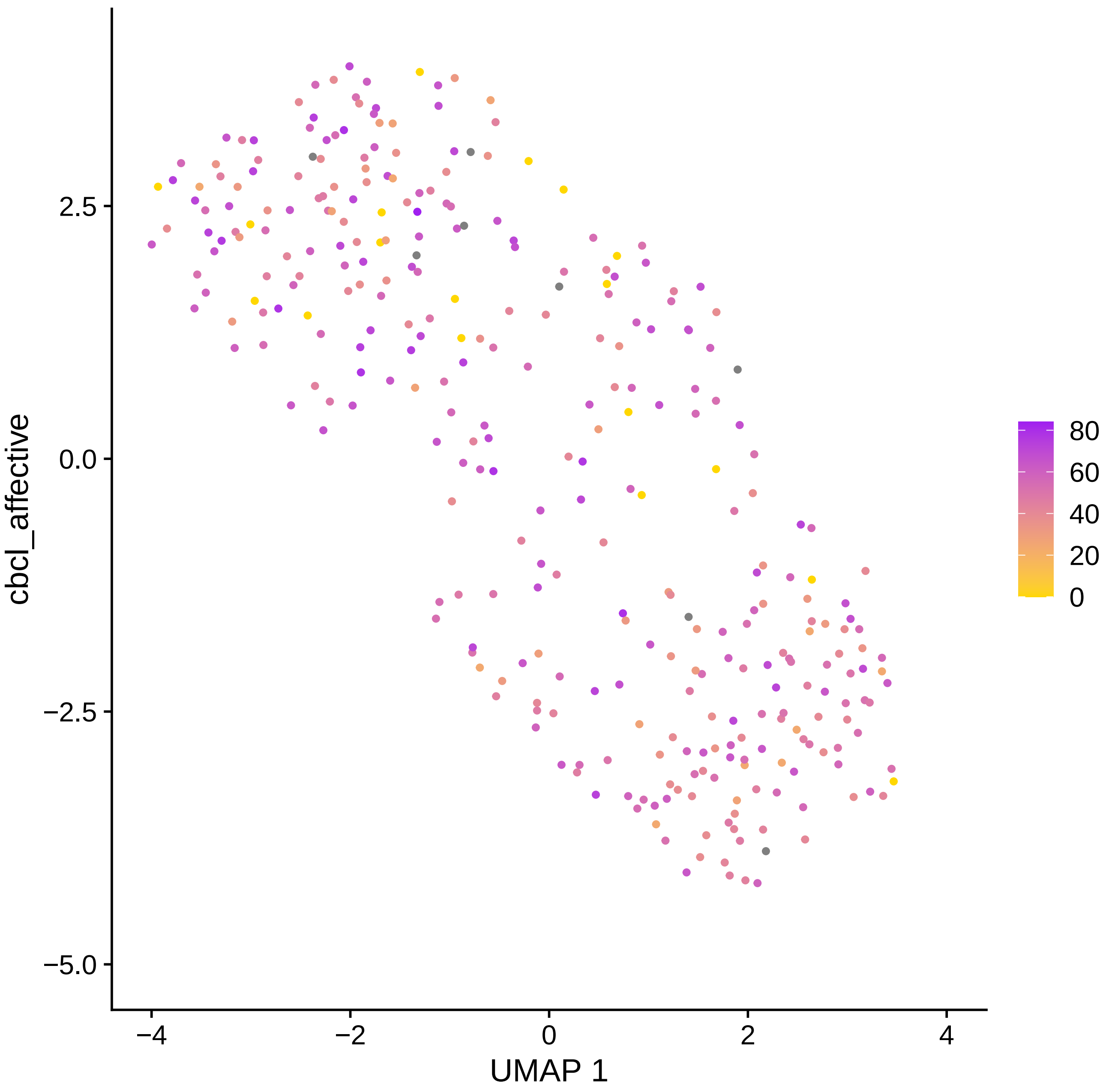

cbcl_affective
UMAP 1
2.5
0.0
−2.5
−5.0
−4
−2
0
2
4
80
60
40
20
0

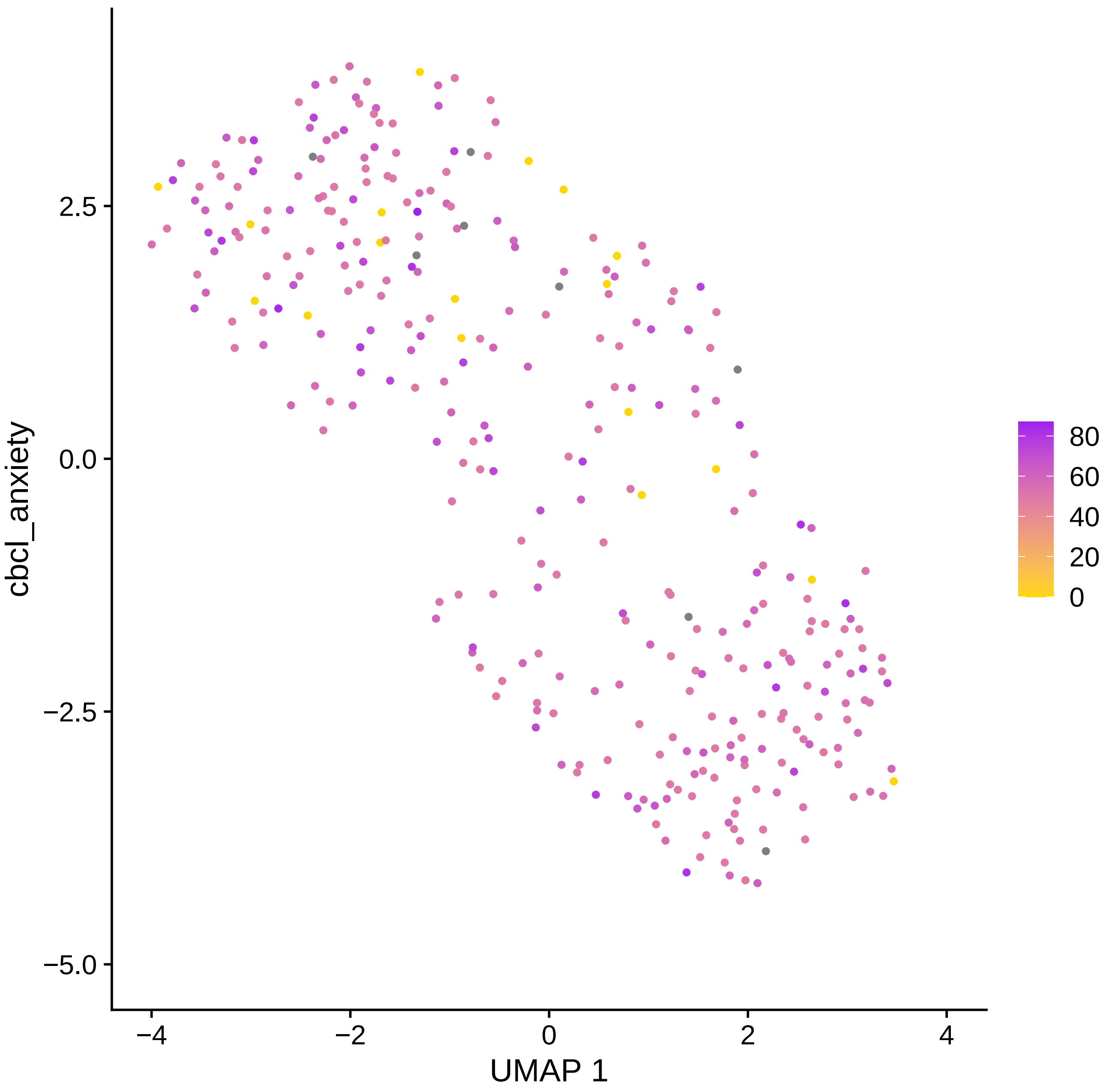

cbcl_anxiety
UMAP 1
2.5
0.0
−2.5
−5.0
−4
−2
0
2
4
80
60
40
20
0

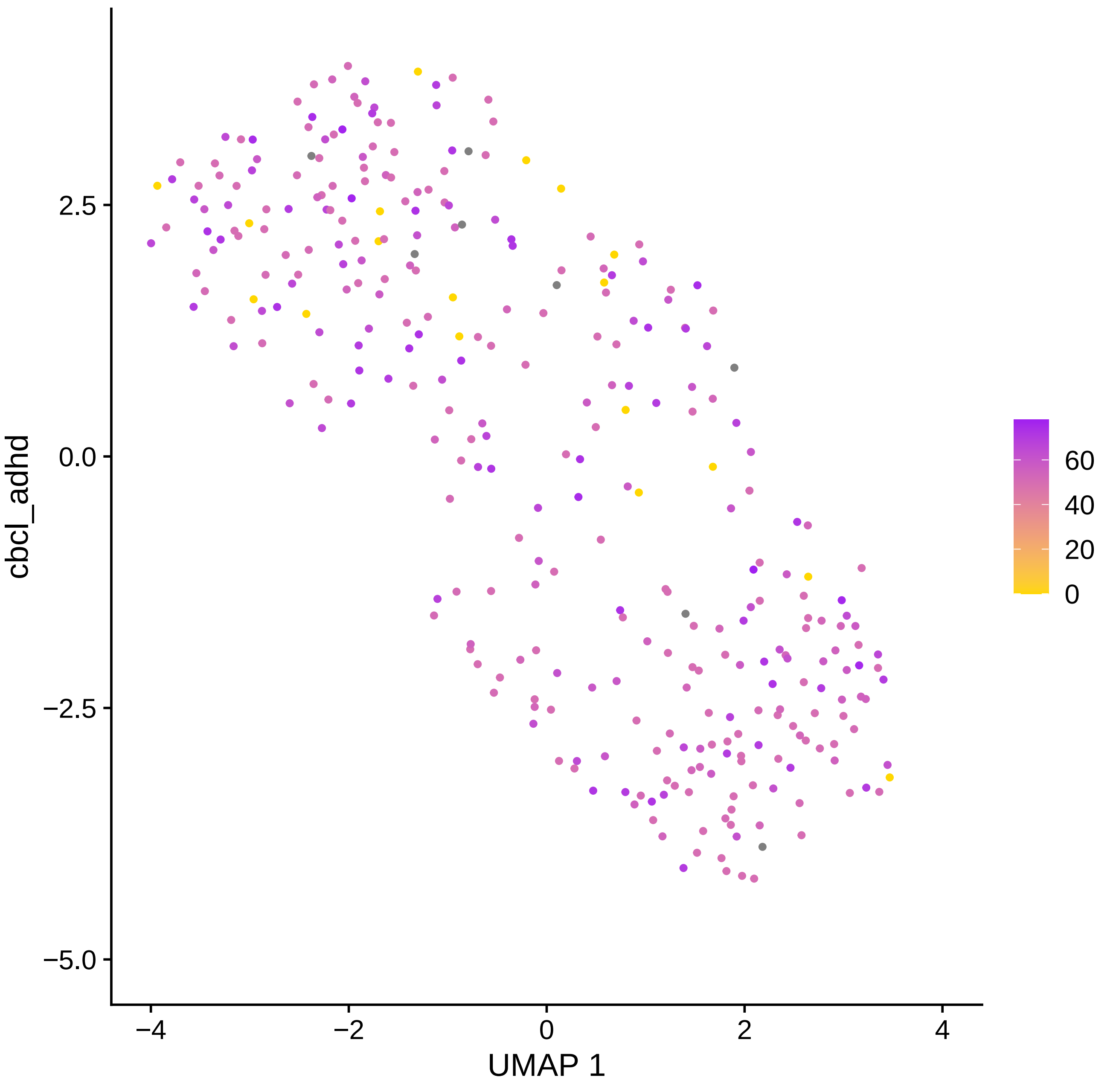

cbcl_adhd
2.5
0.0
−2.5
−5.0
−4
−2
0
2
4
UMAP 1
60
40
20
0

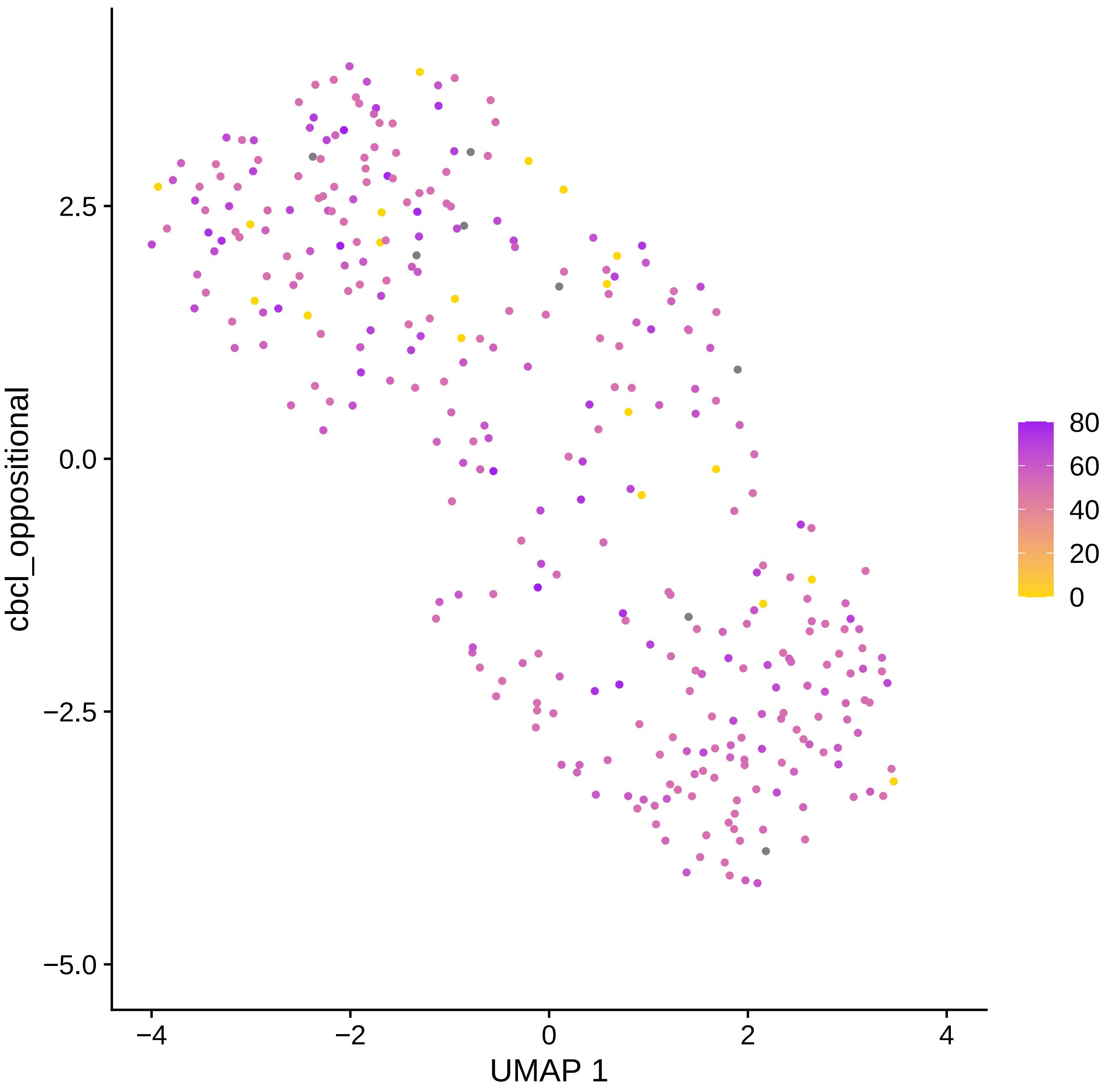
cbcl_oppositional
2.5
0.0
−2.5
−5.0
−4
−2
0
2
4
UMAP 1
80
60
40
20
0

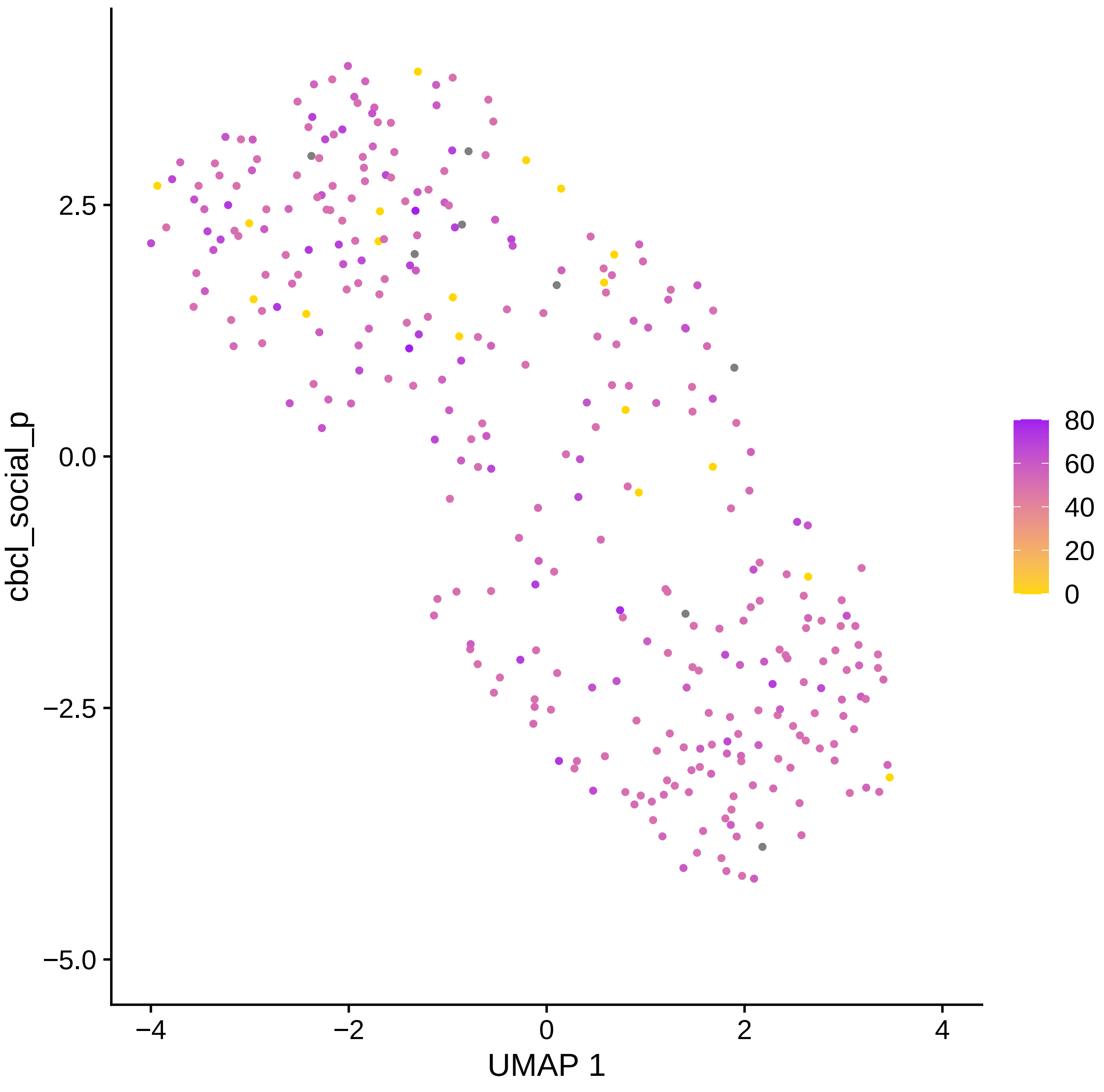

cbcl_social_p
UMAP 1
2.5
0.0
−2.5
−5.0
−4
−2
0
2
4
80
60
40
20
0

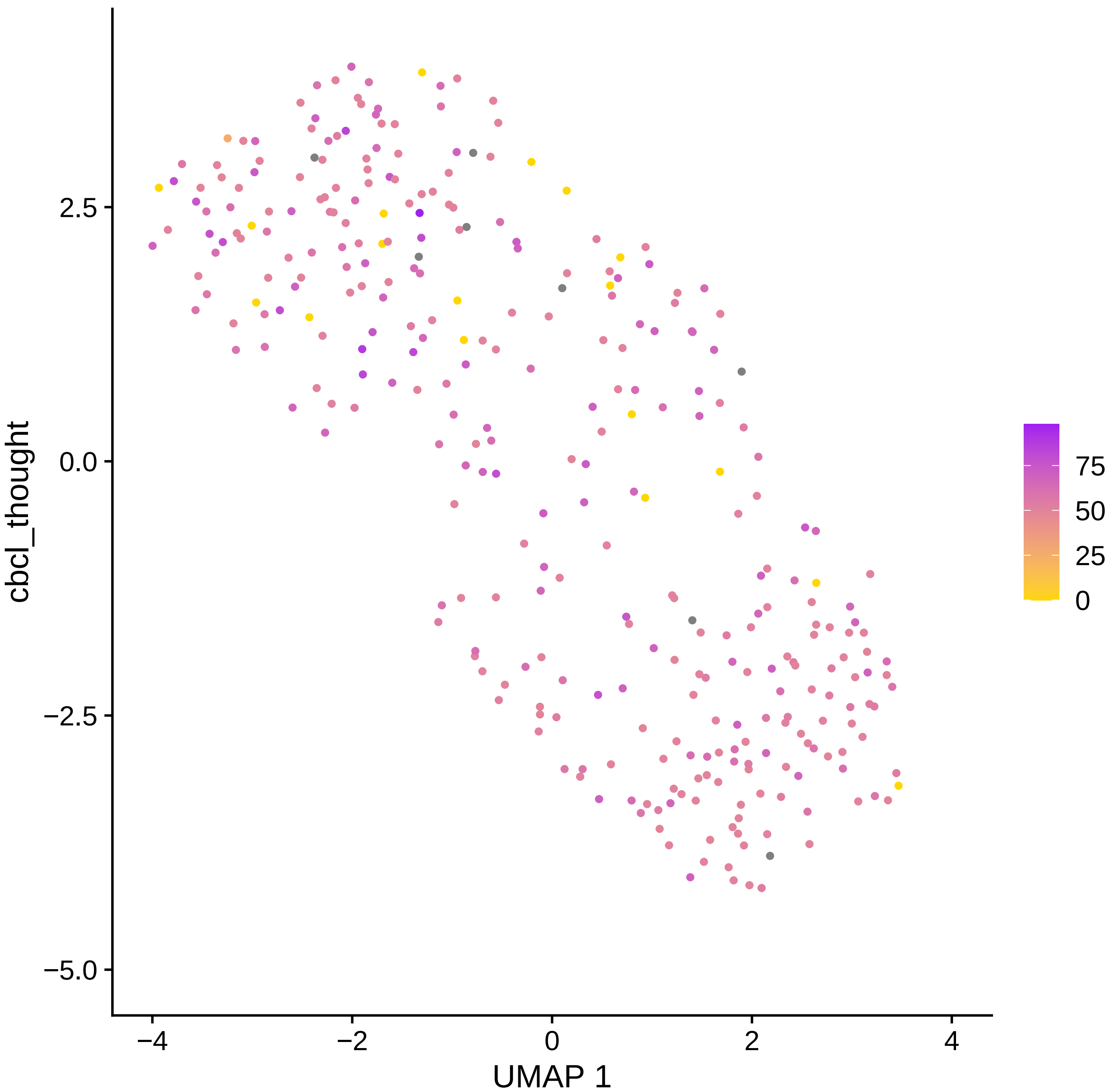

cbcl_thought
2.5
0.0
−2.5
−5.0
−4
−2
0
2
4
UMAP 1
75
50
25
0

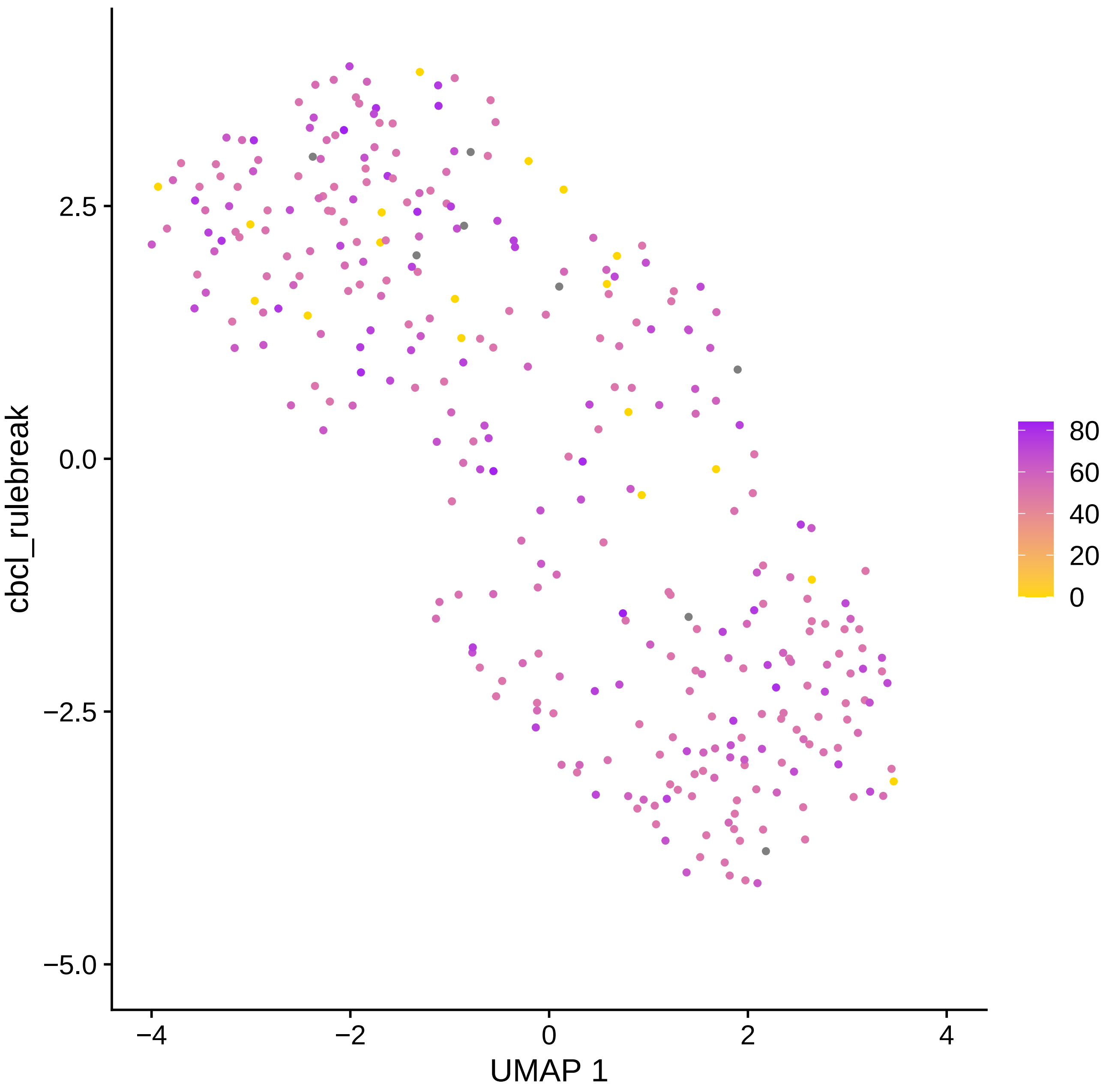

cbcl_rulebreak
UMAP 1
2.5
0.0
−2.5
−5.0
−4
−2
0
2
4
80
60
40
20
0

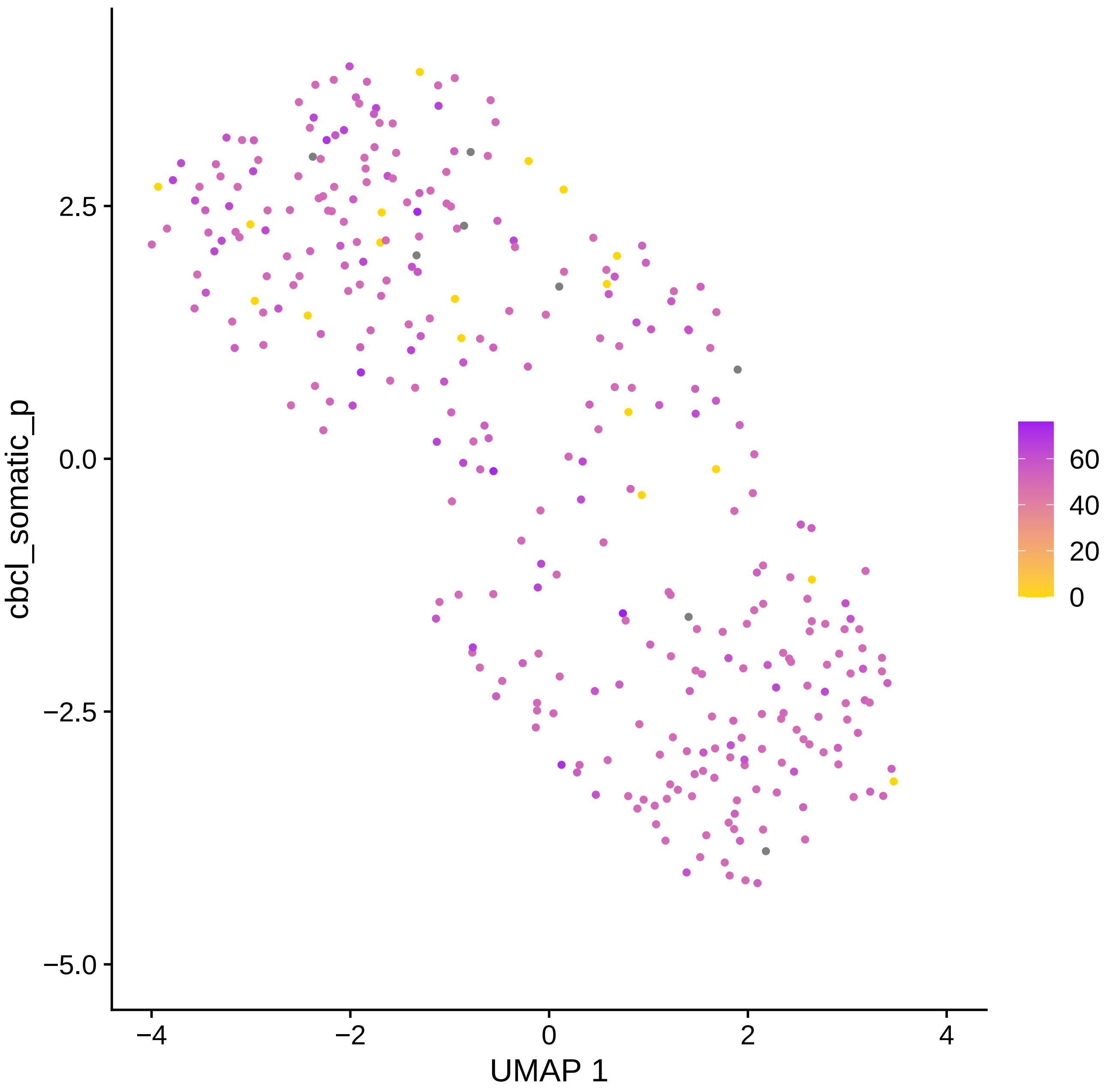
cbcl_somatic_p
UMAP 1
2.5
0.0
−2.5
−5.0
−4
−2
0
2
4
60
40
20
0

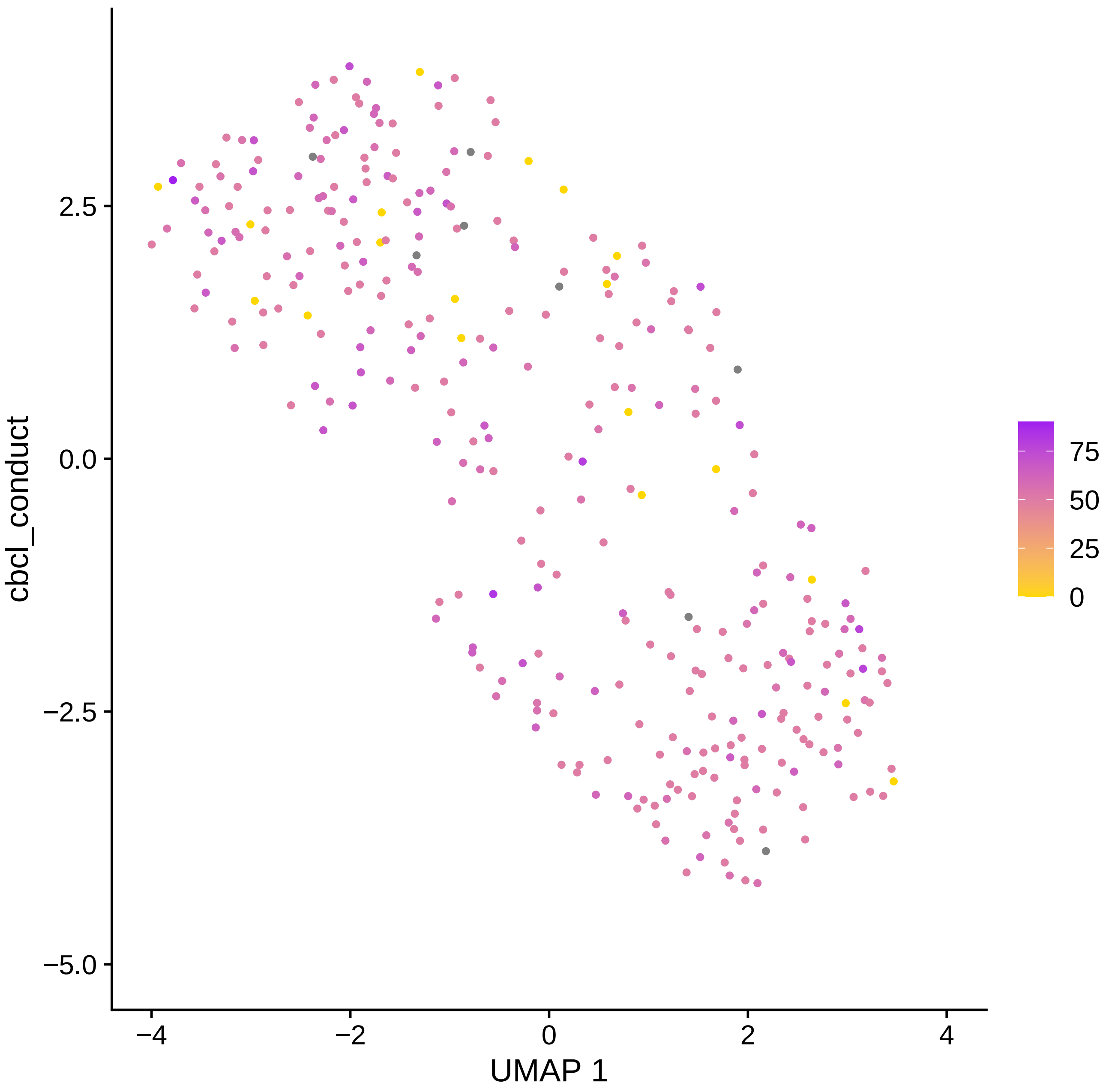
cbcl_conduct
UMAP 1
2.5
0.0
−2.5
−5.0
−4
−2
0
2
4
75
50
25
0

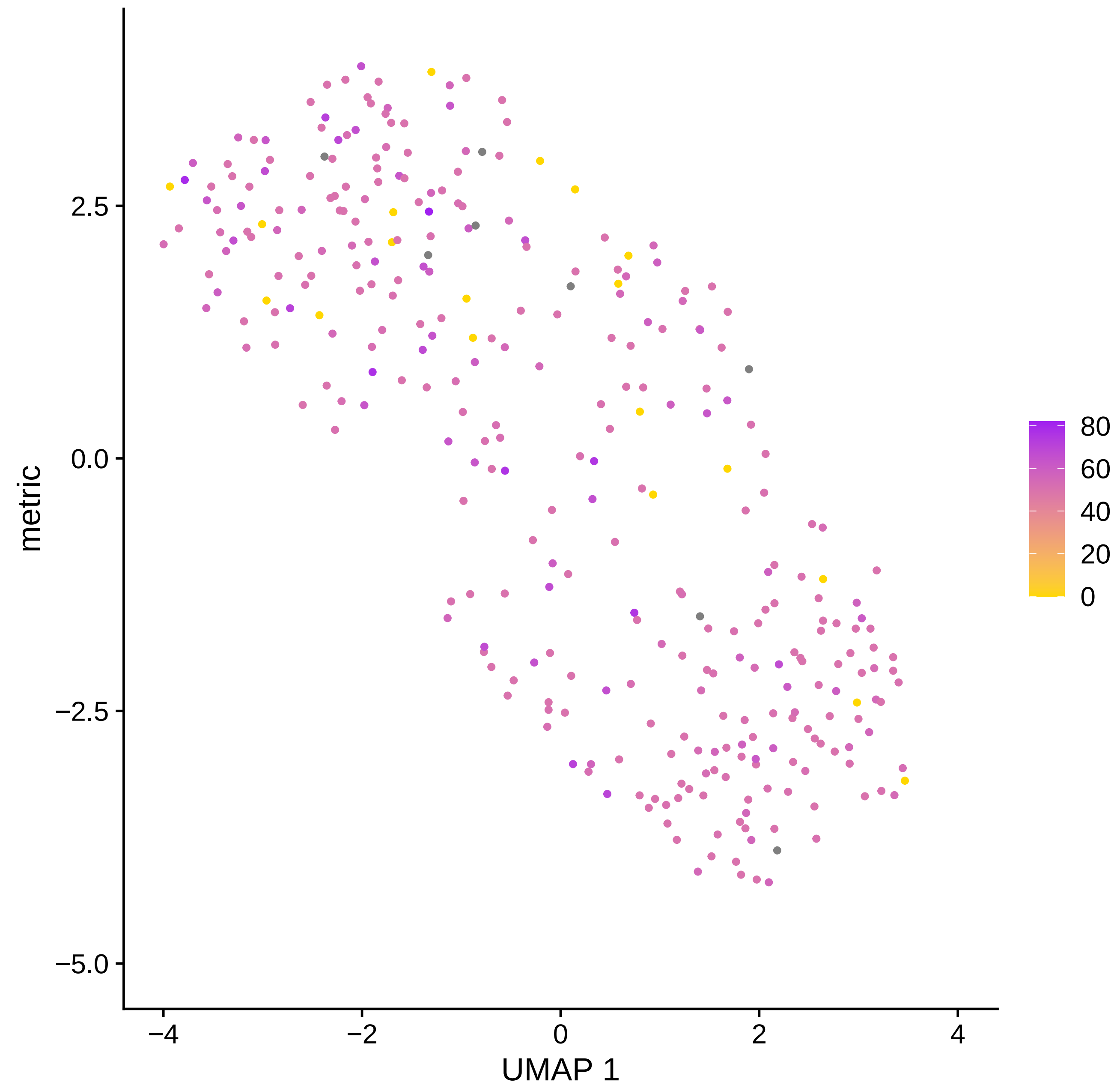
metric
2.5
0.0
−2.5
−5.0
−4
−2
0
2
4
UMAP 1
80
60
40
20
0
