# Supplementary figures and images for: Widespread associations between behavioral metrics and brain microstructure in ASD suggest age mediates subtypes of ASD
Source: Imaging Neurosci (Camb). 2025 Sep 10;3:IMAG.a.144. doi: 10.1162/IMAG.a.144 (PMC12423640; doi:10.1162/IMAG.a.144)

CHOIR clusters (.. = 0.05)

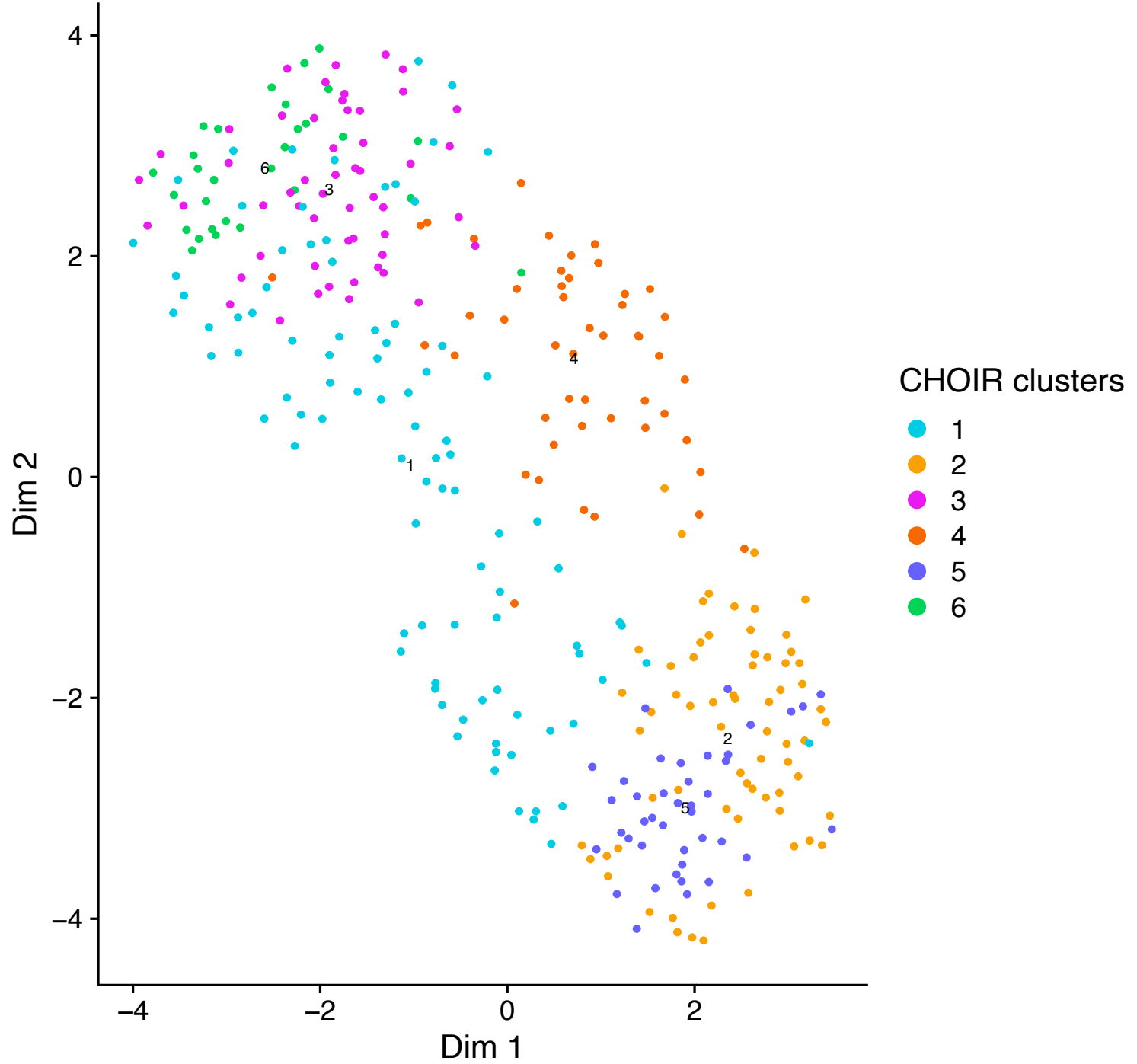

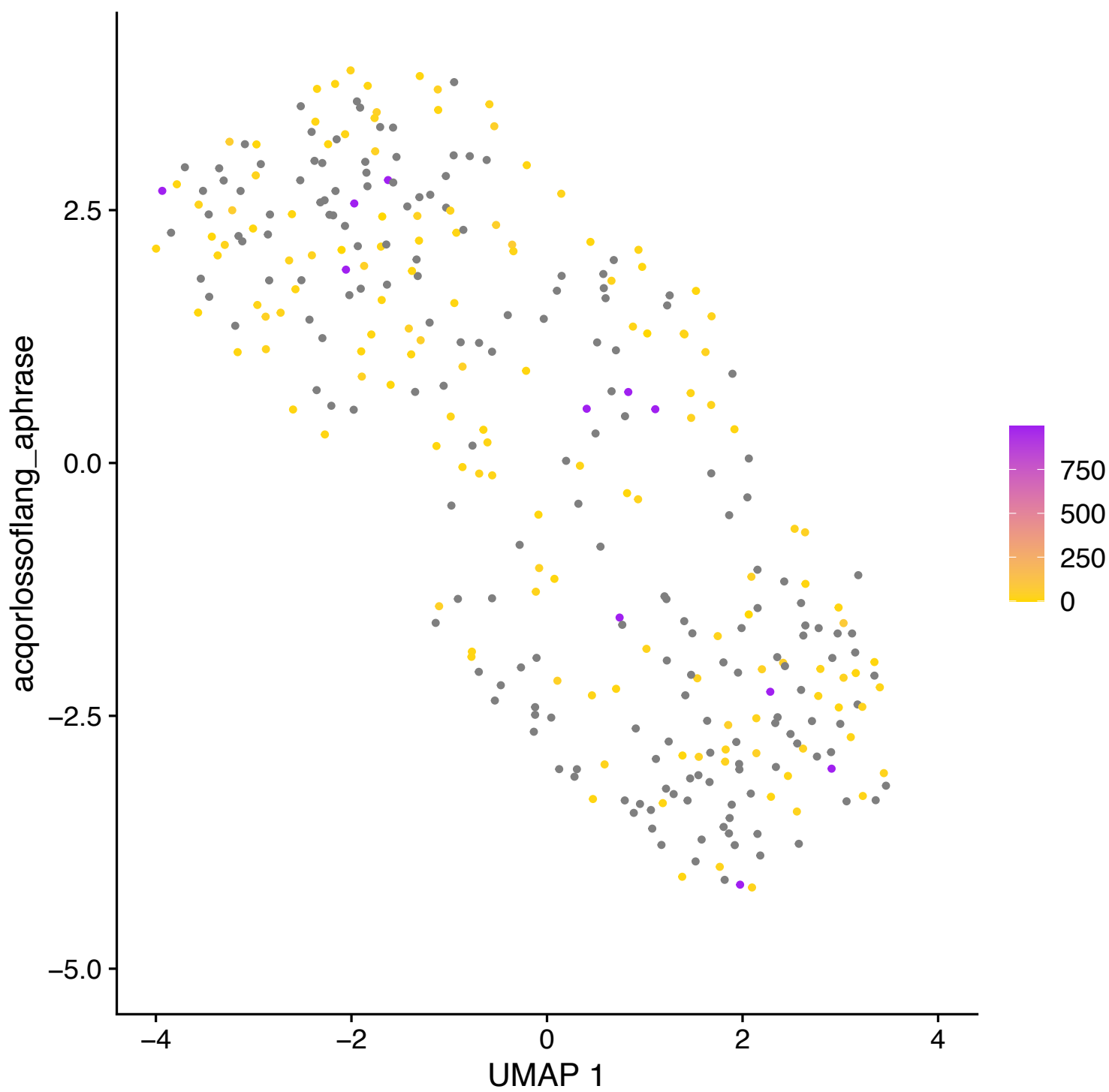

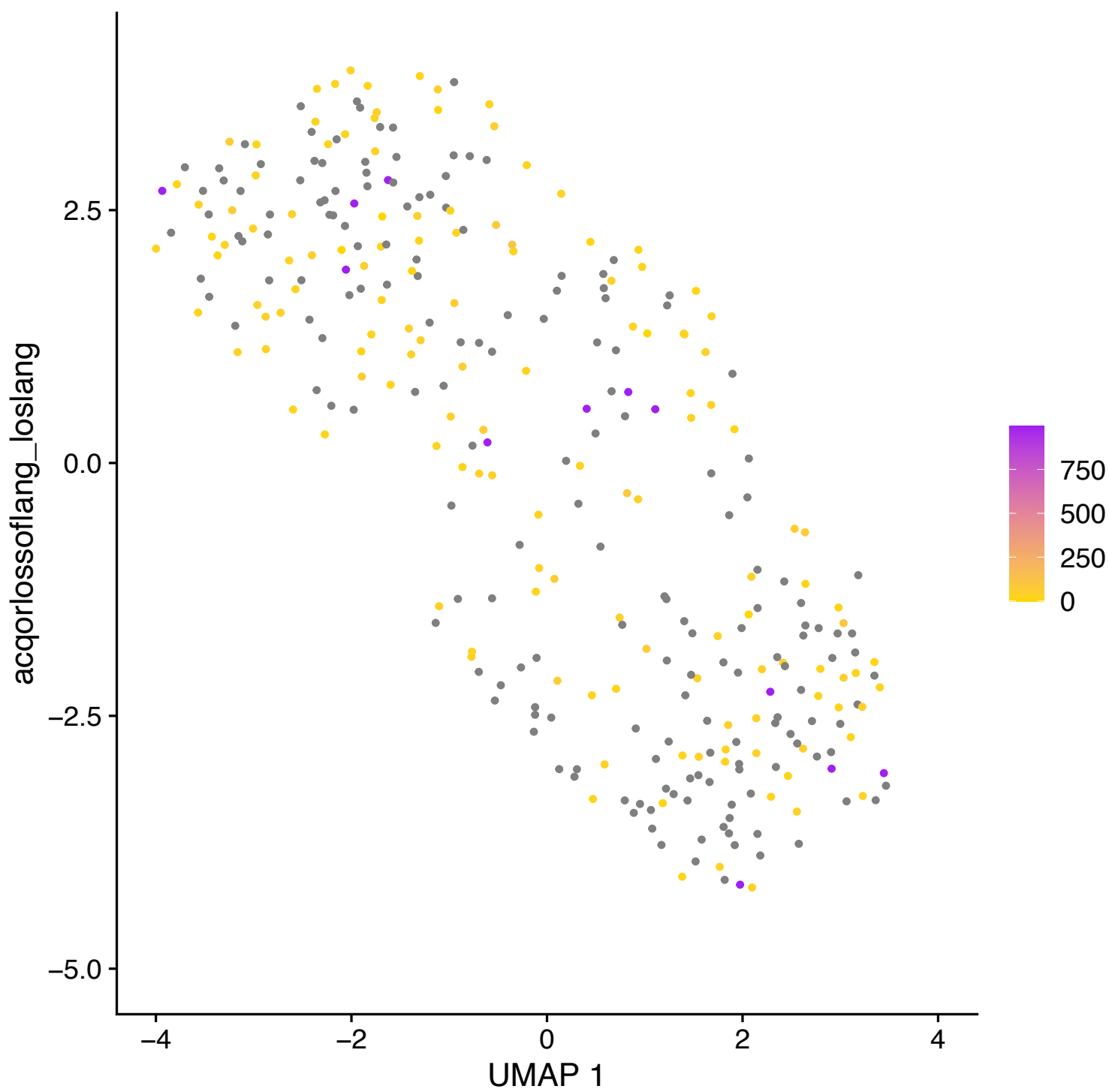

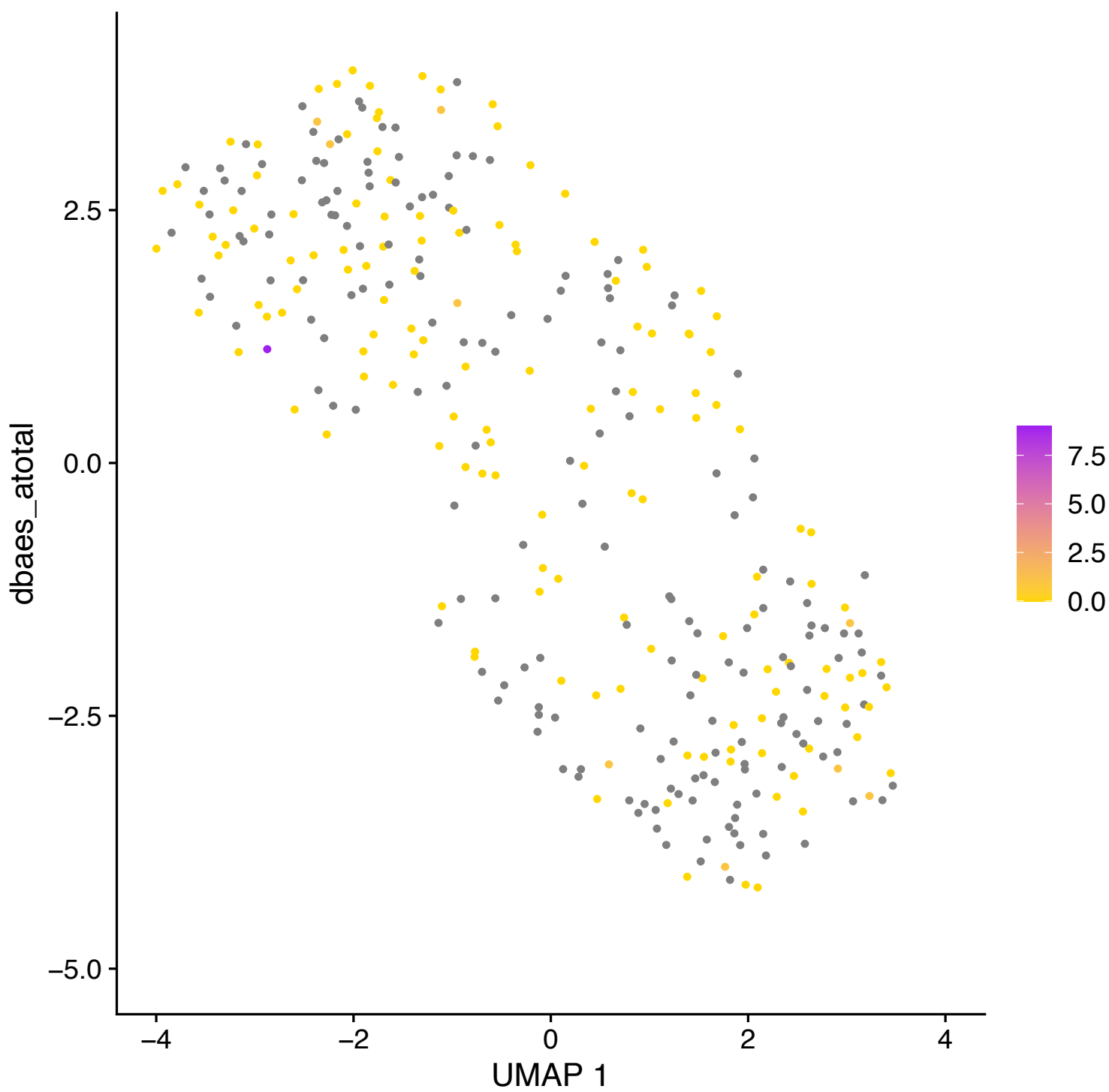

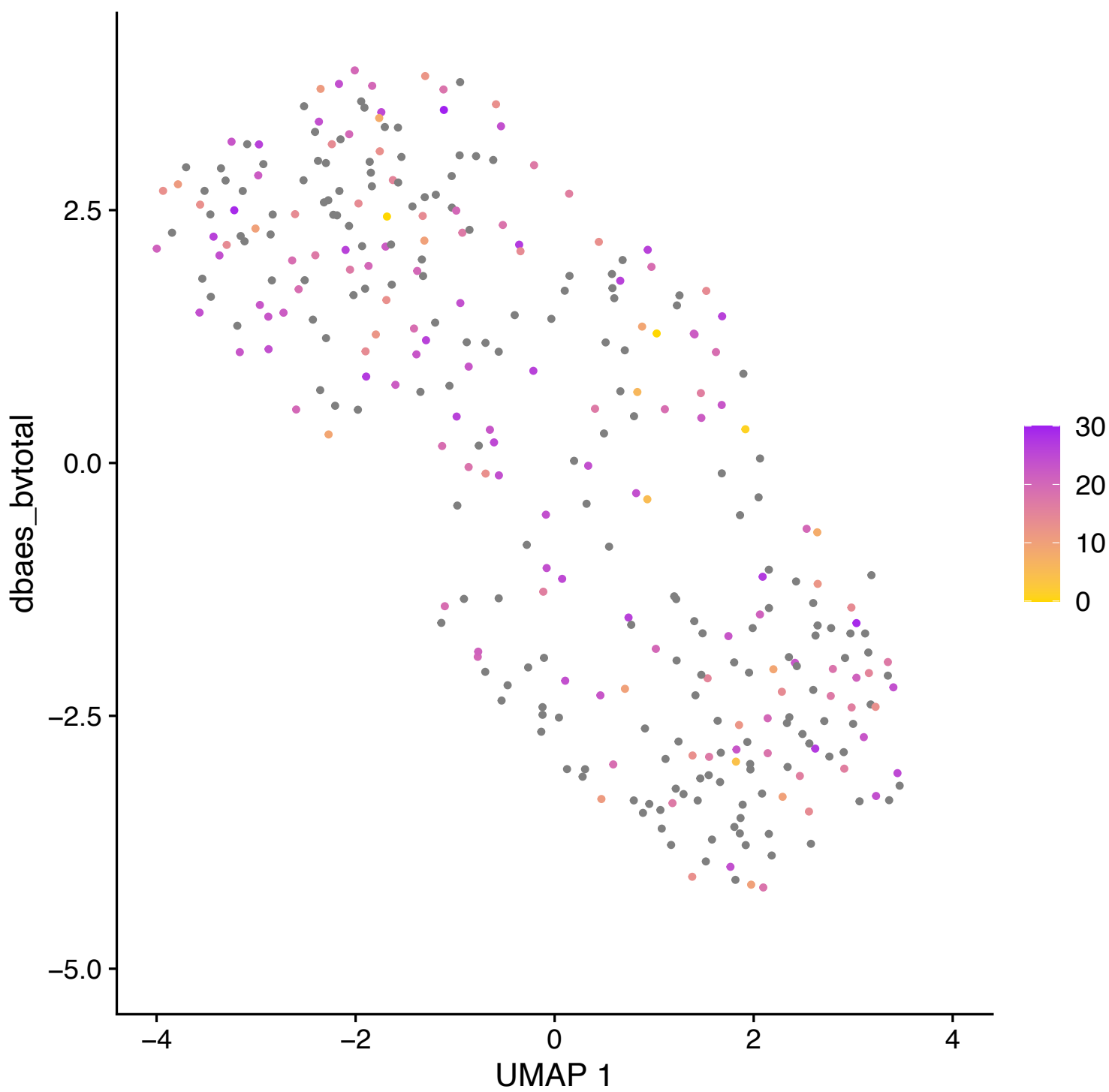

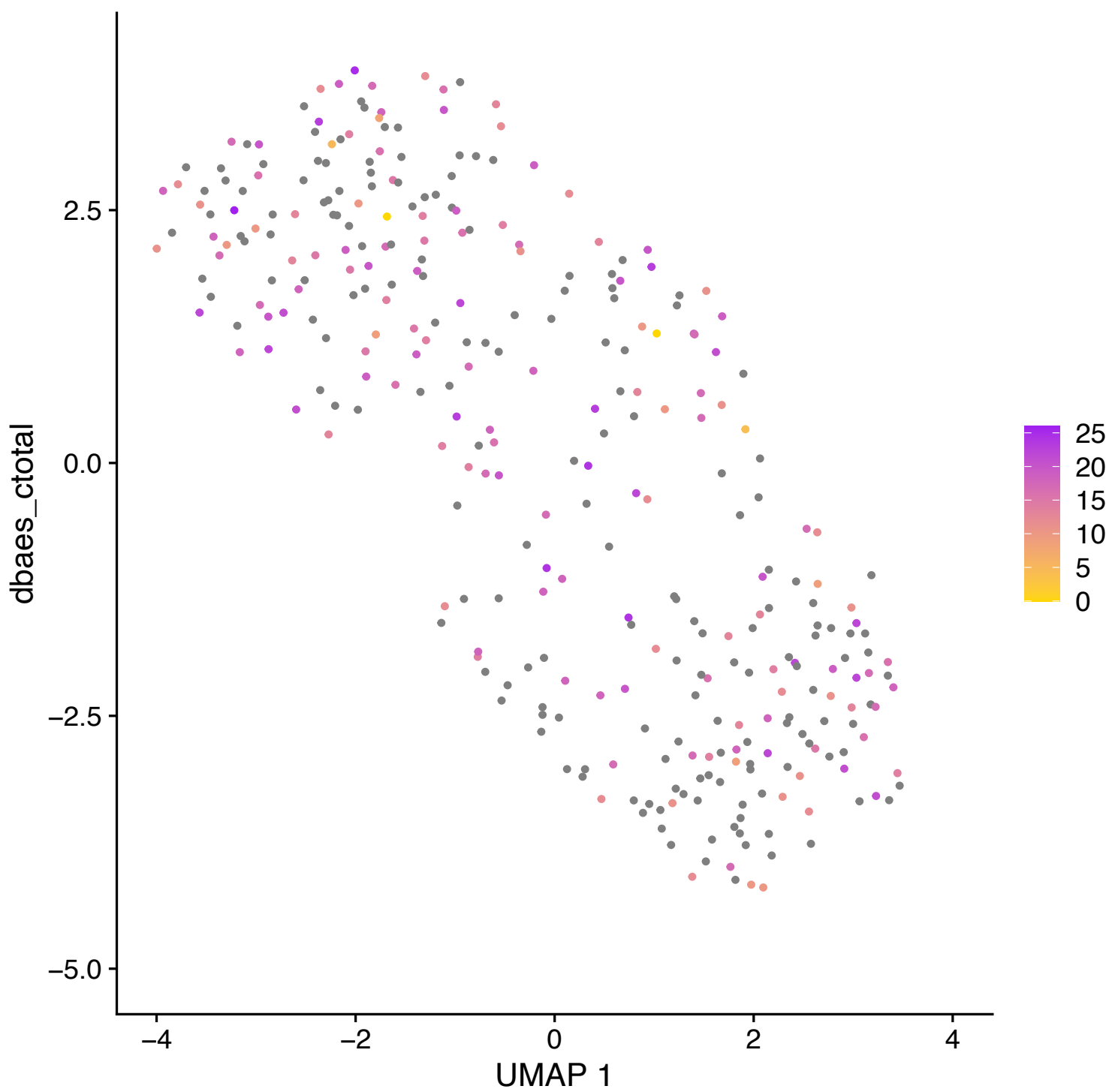

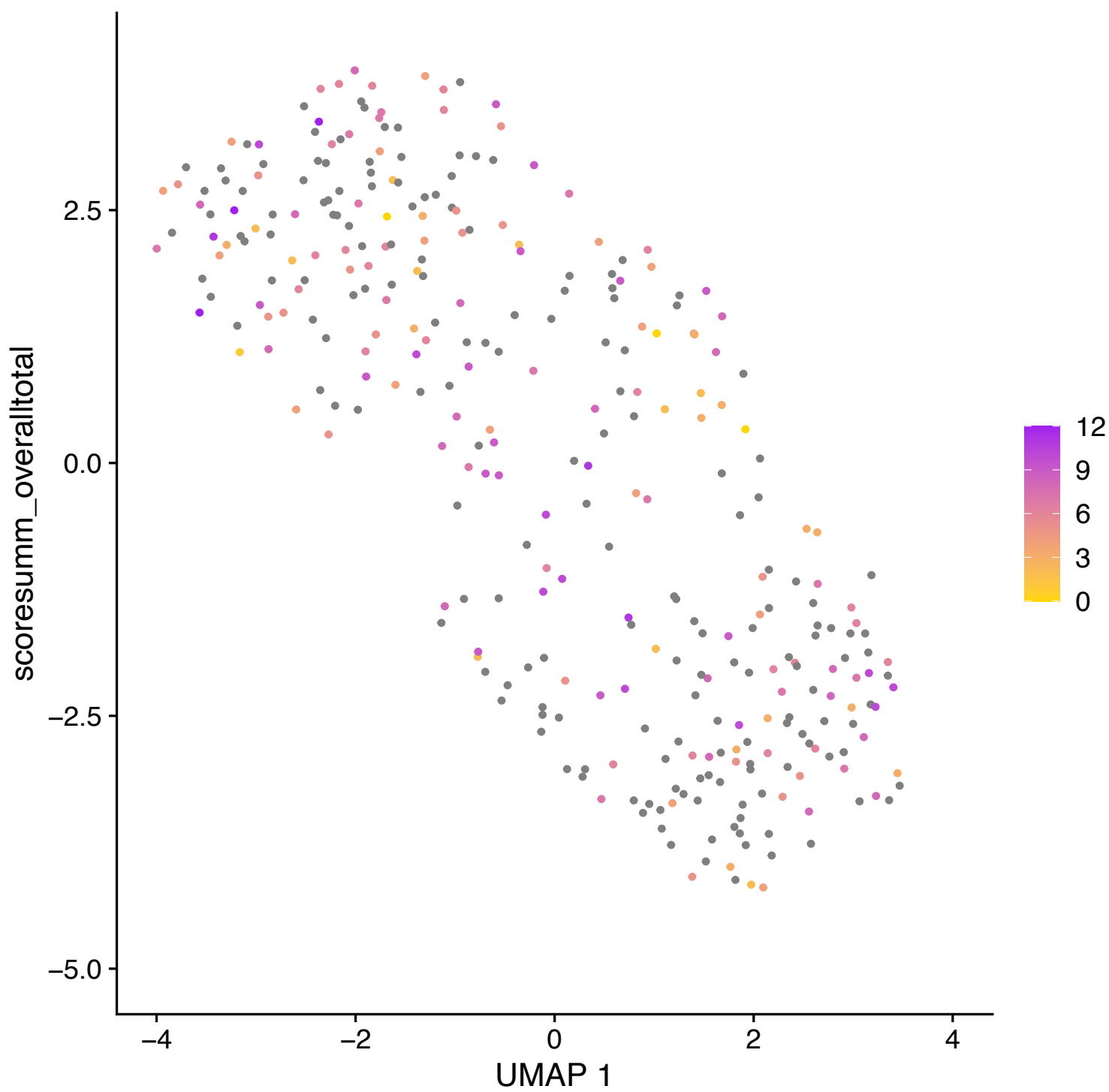

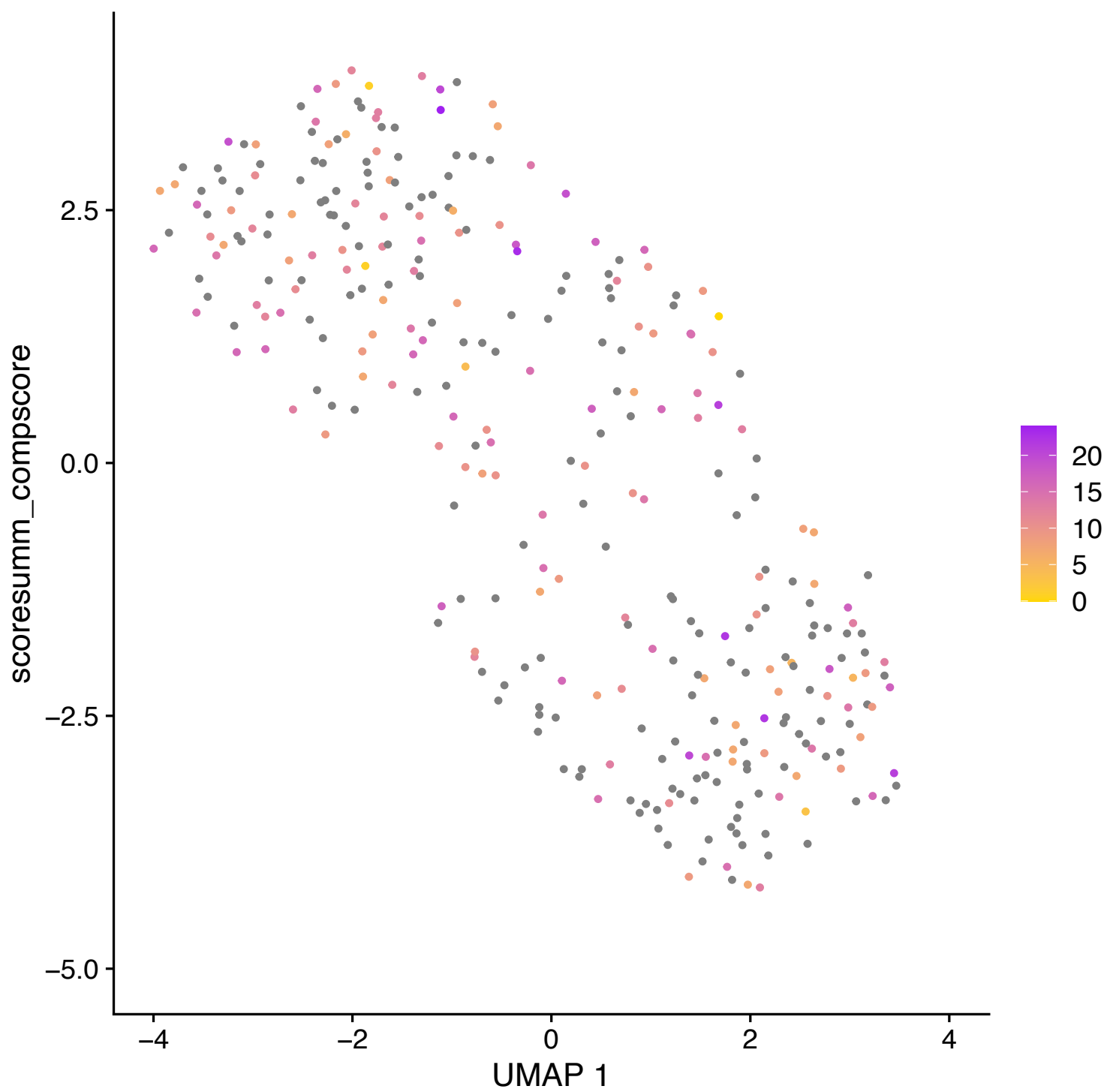

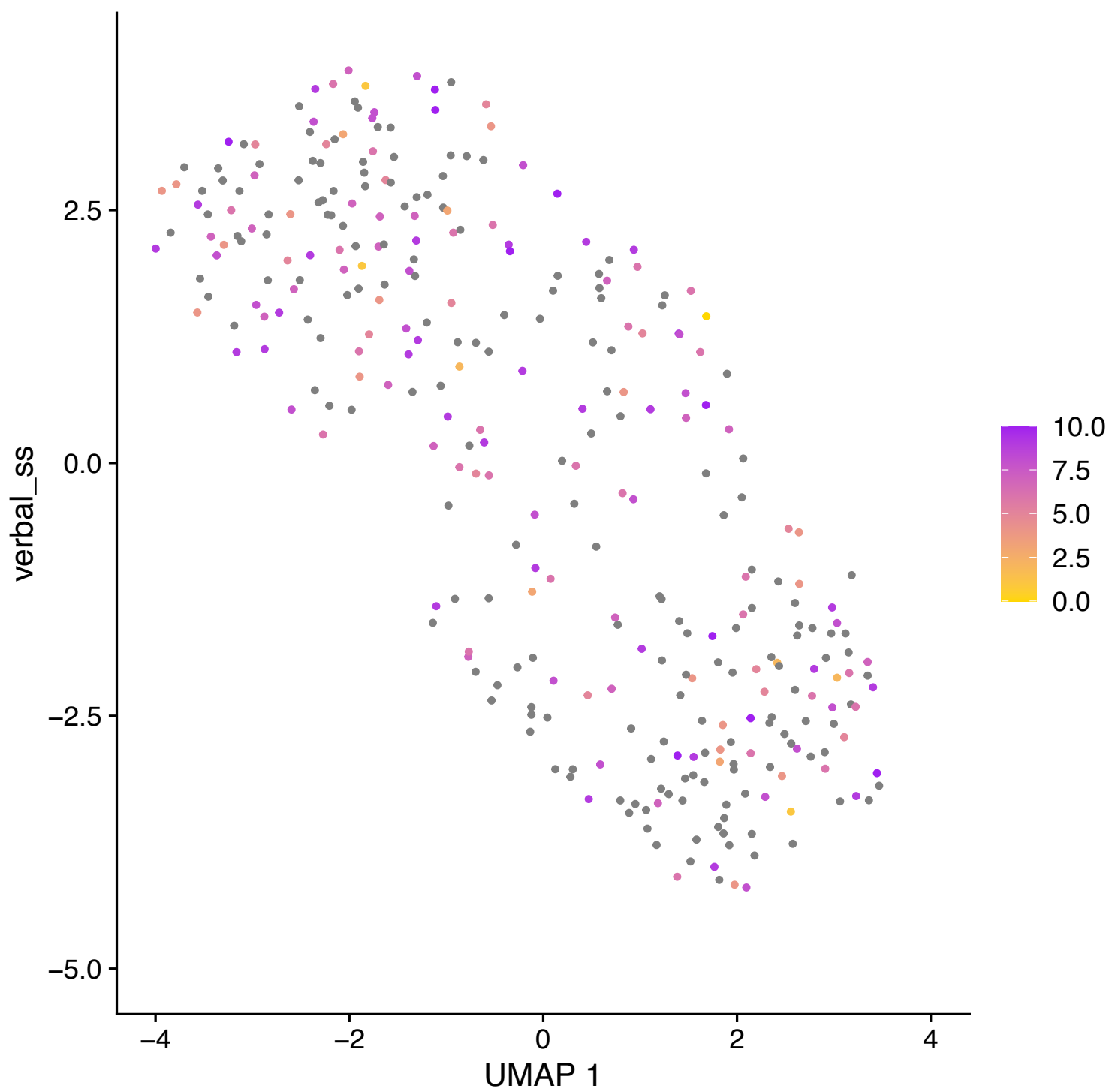

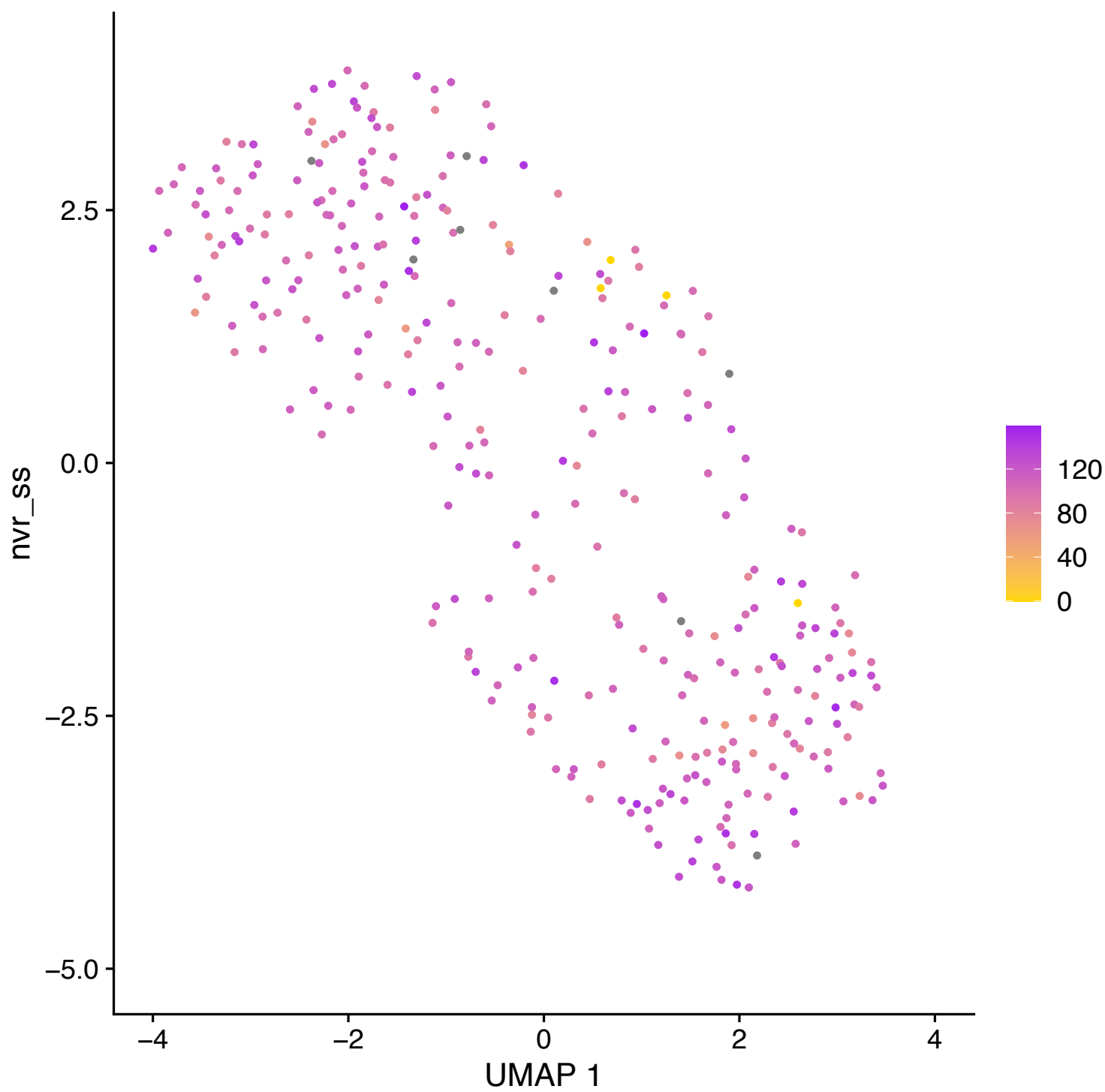

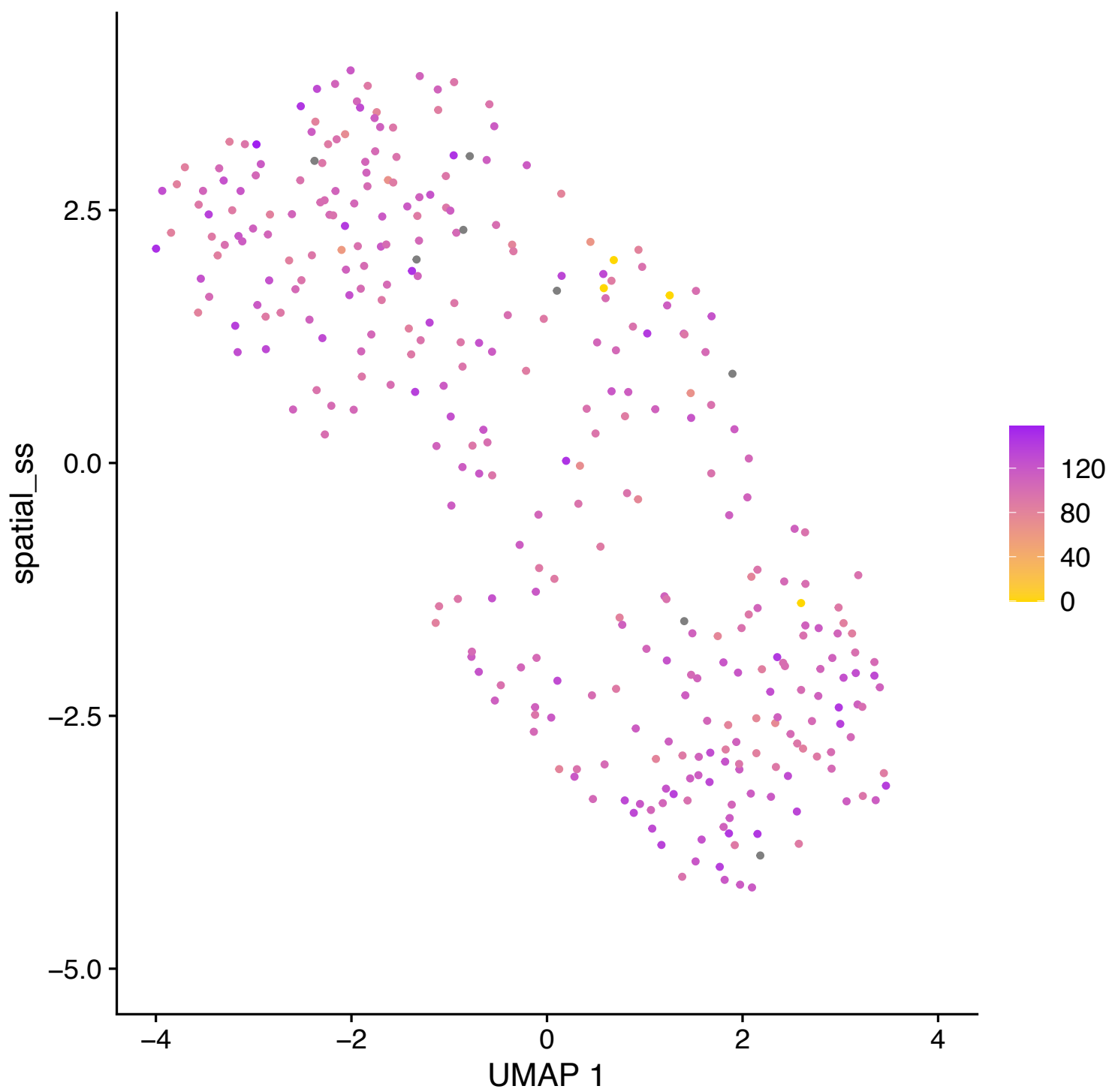

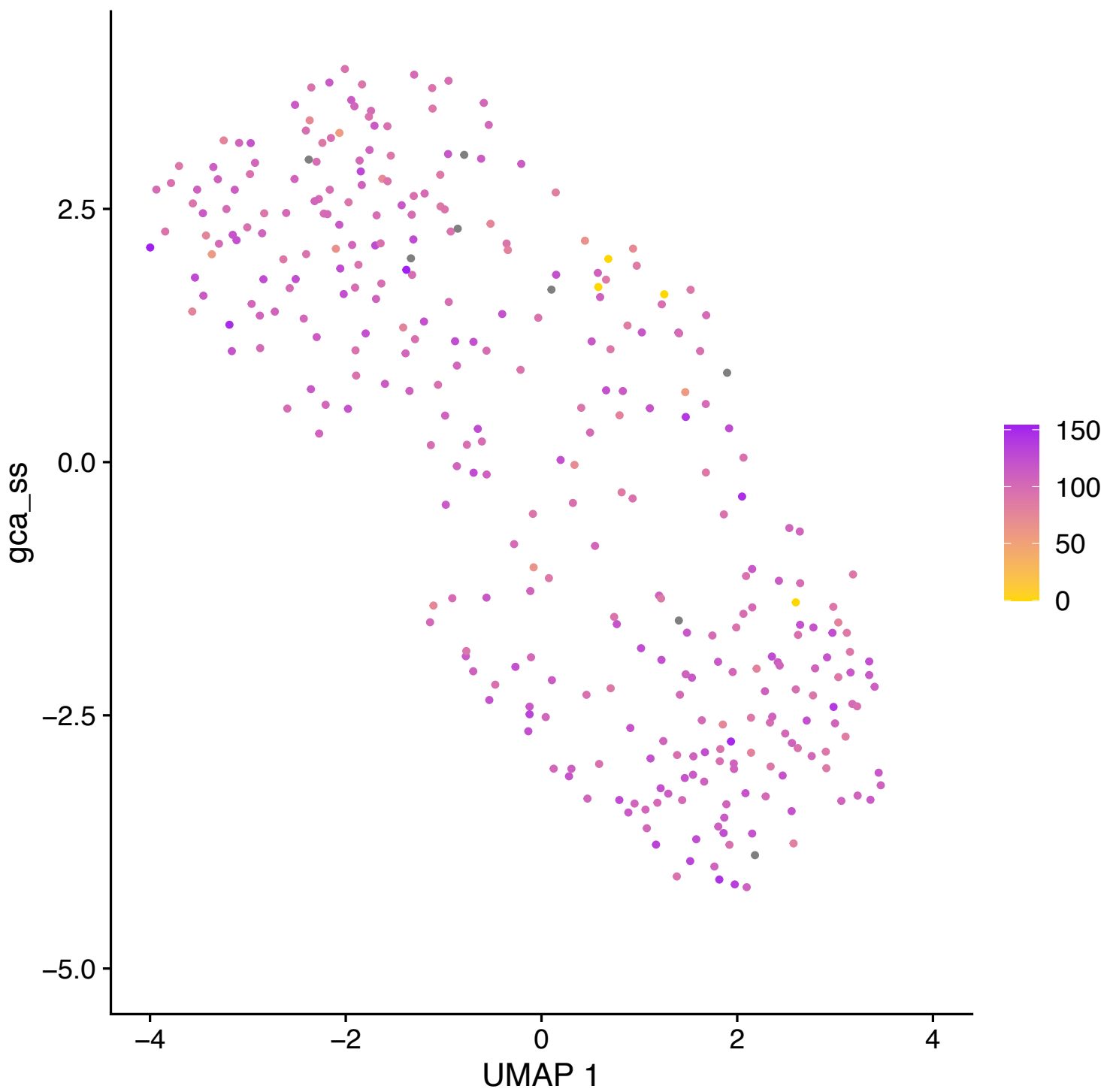

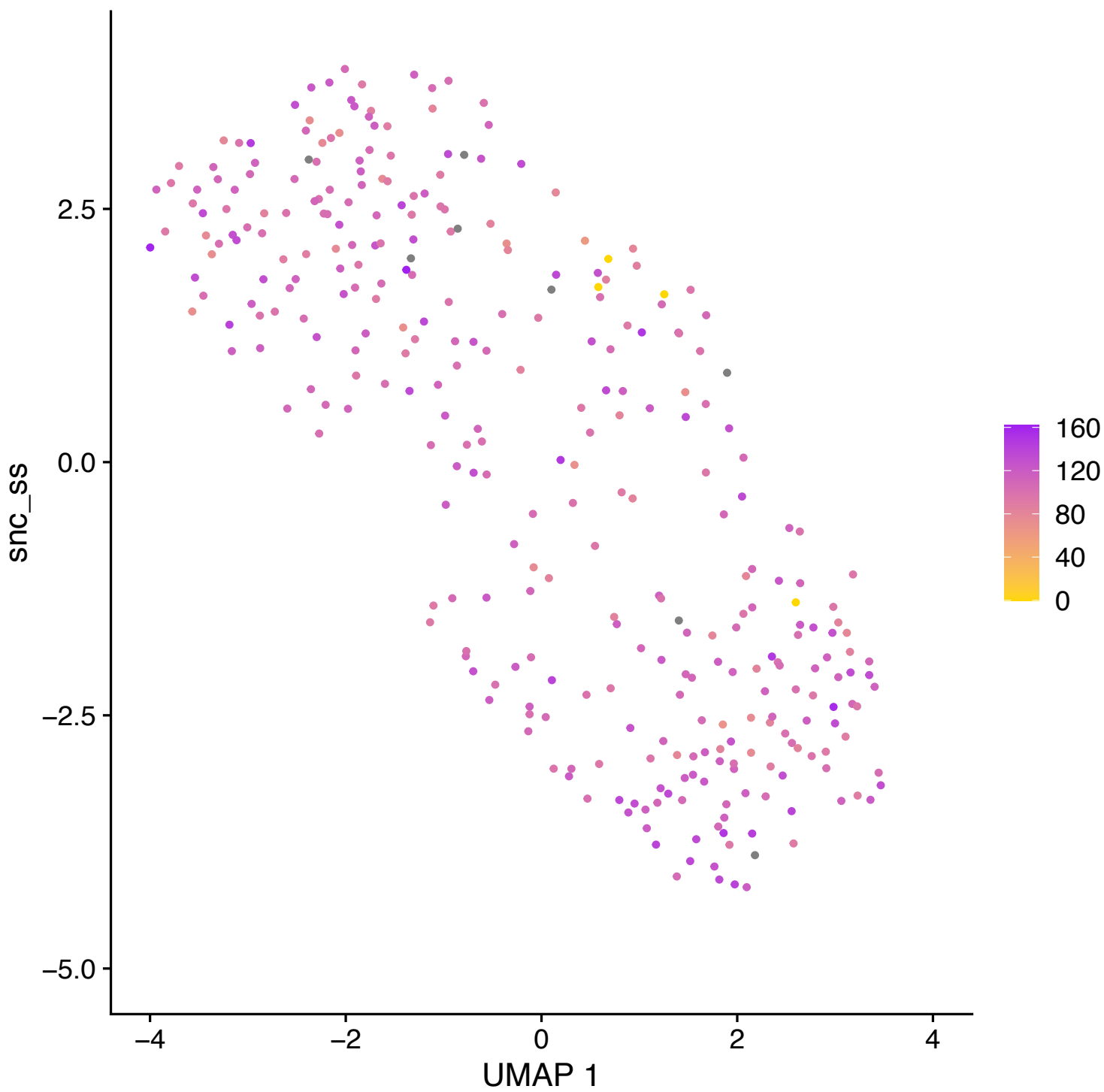

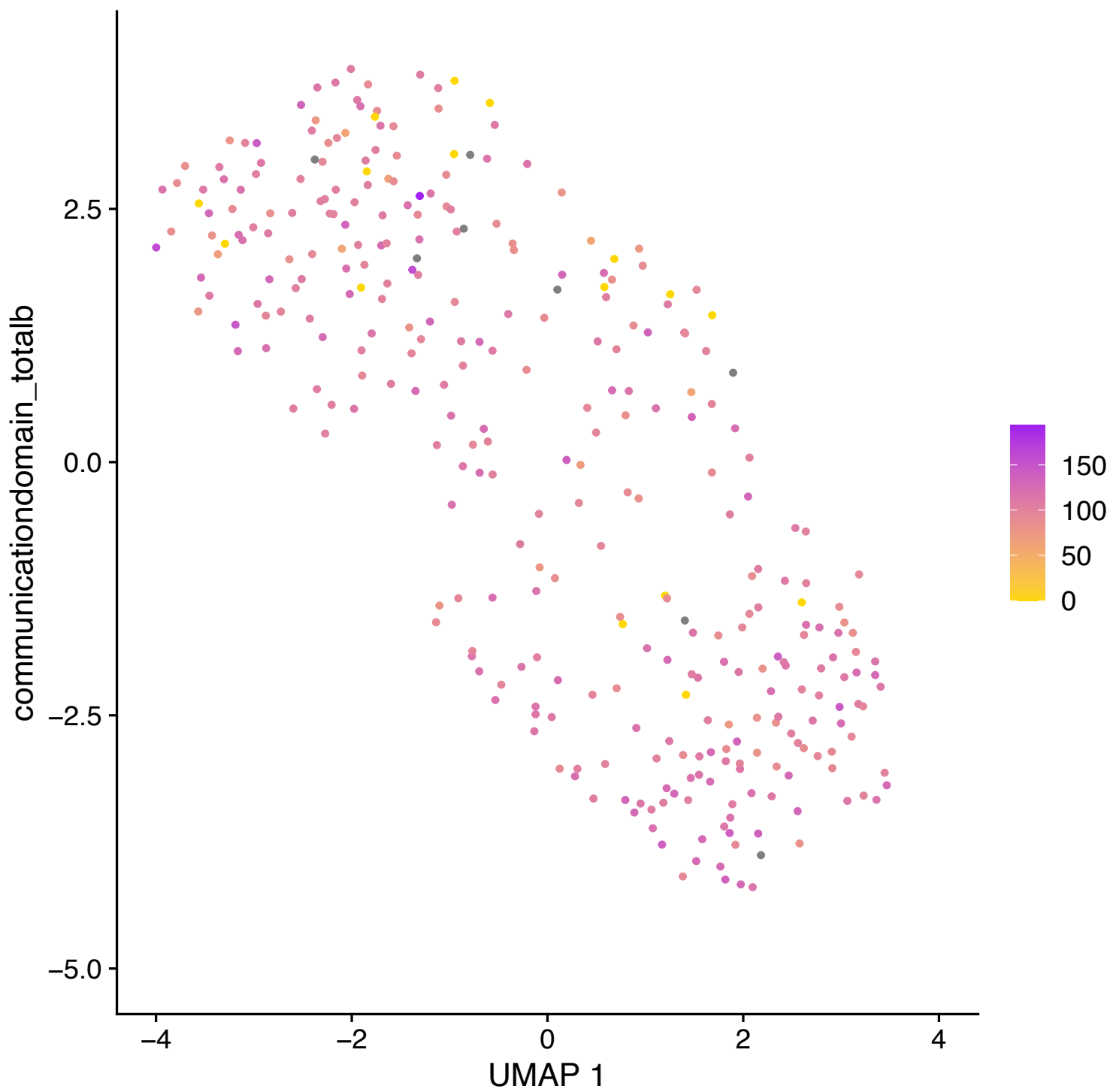

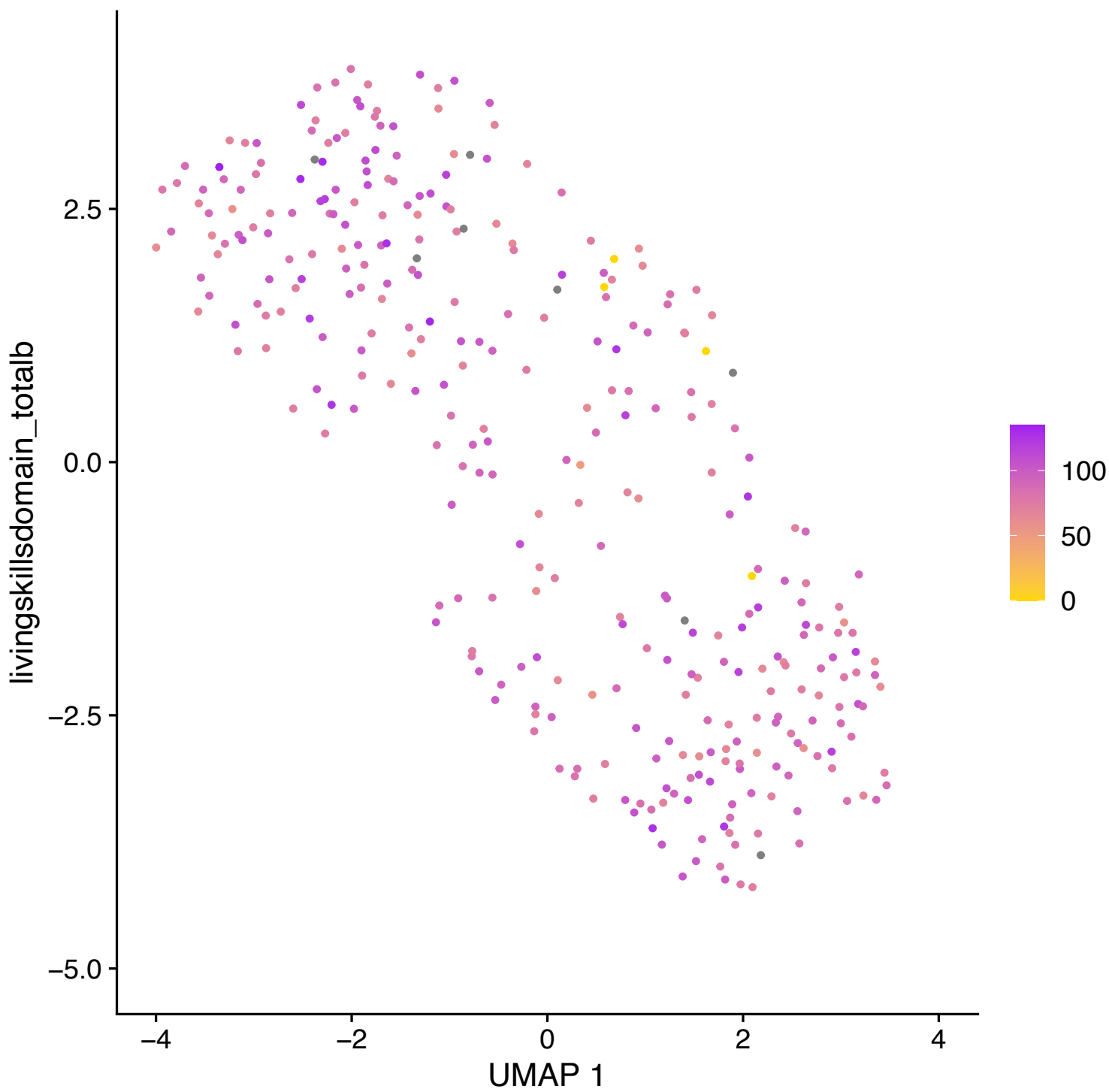

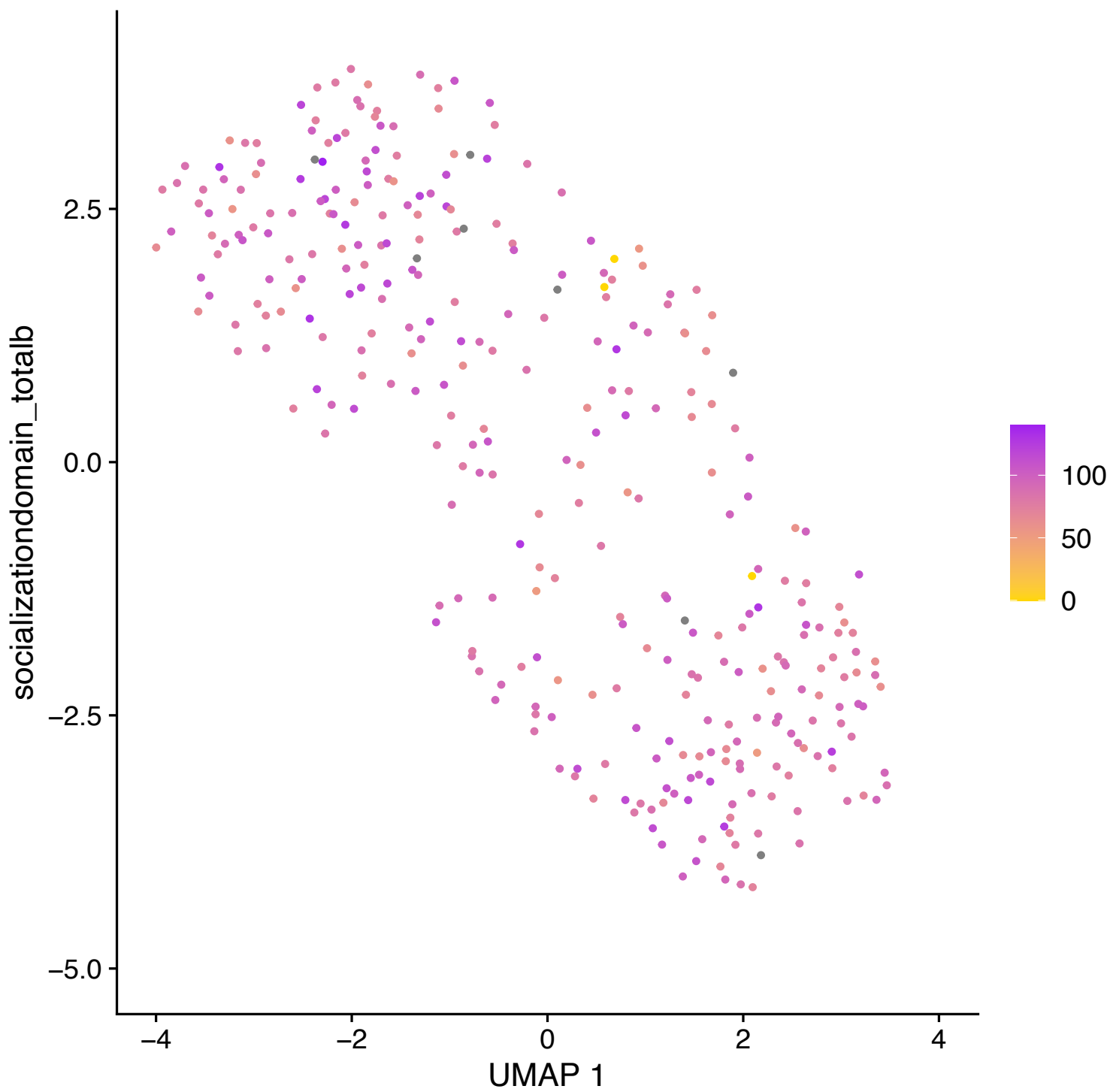

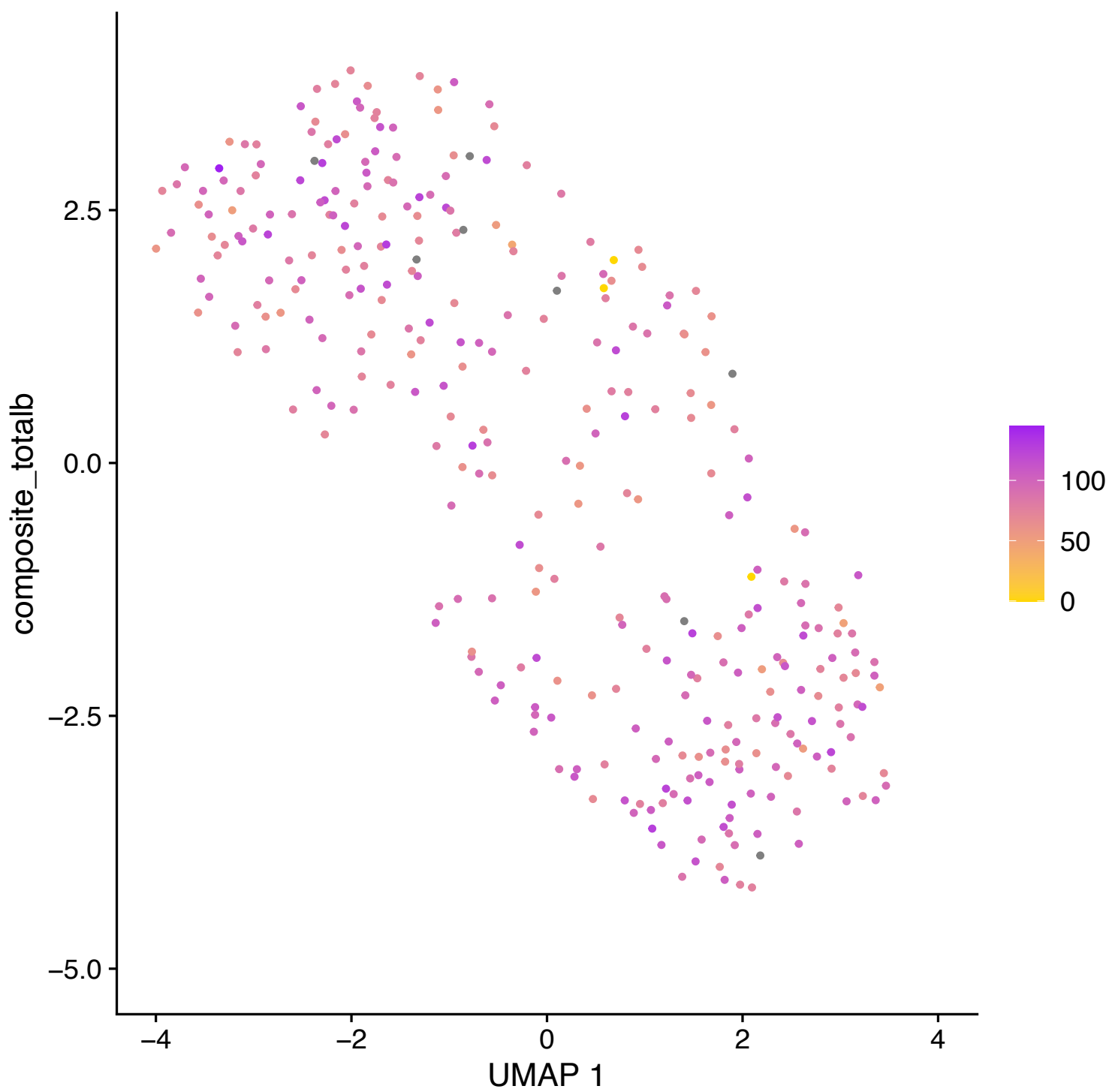

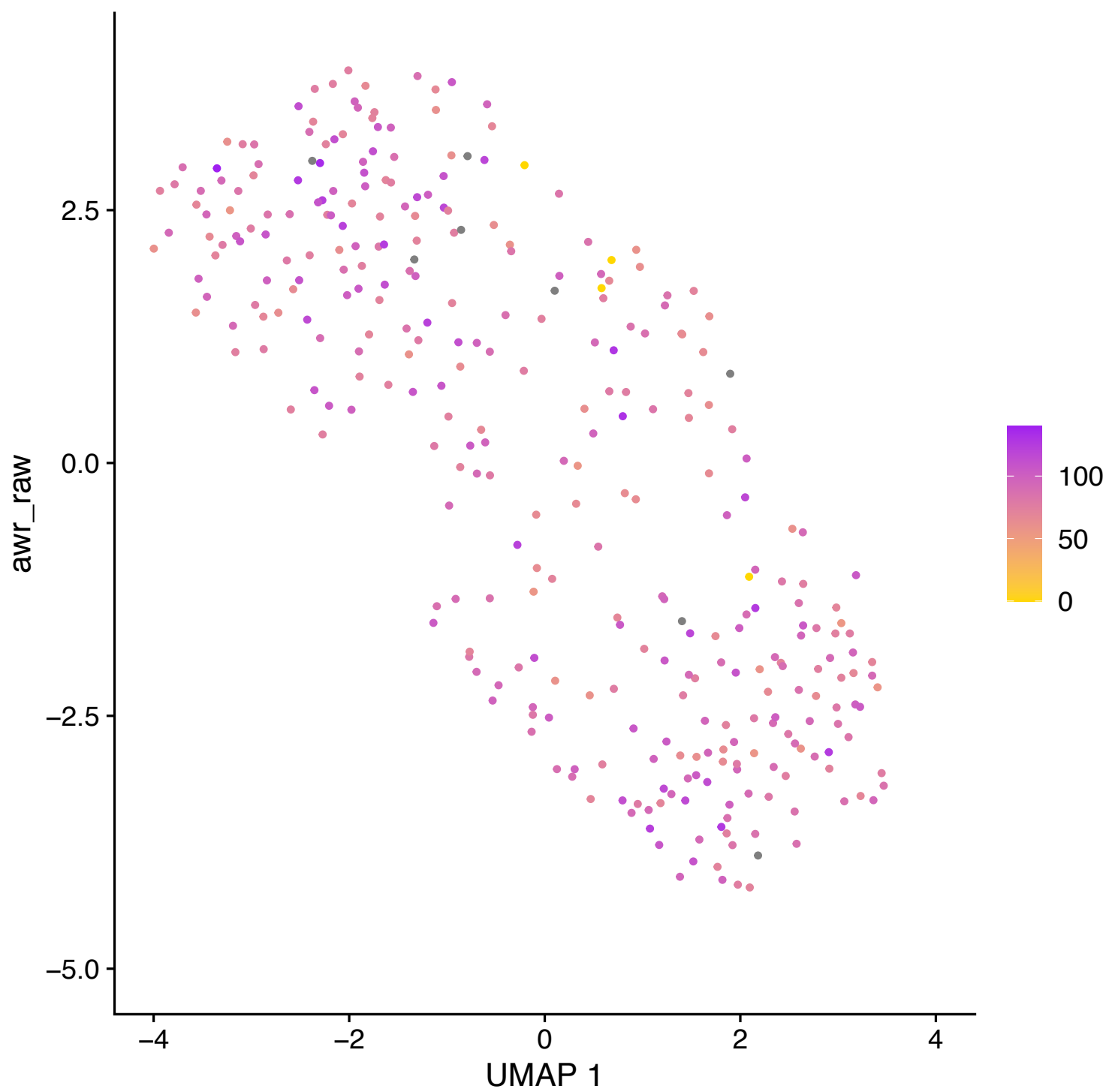

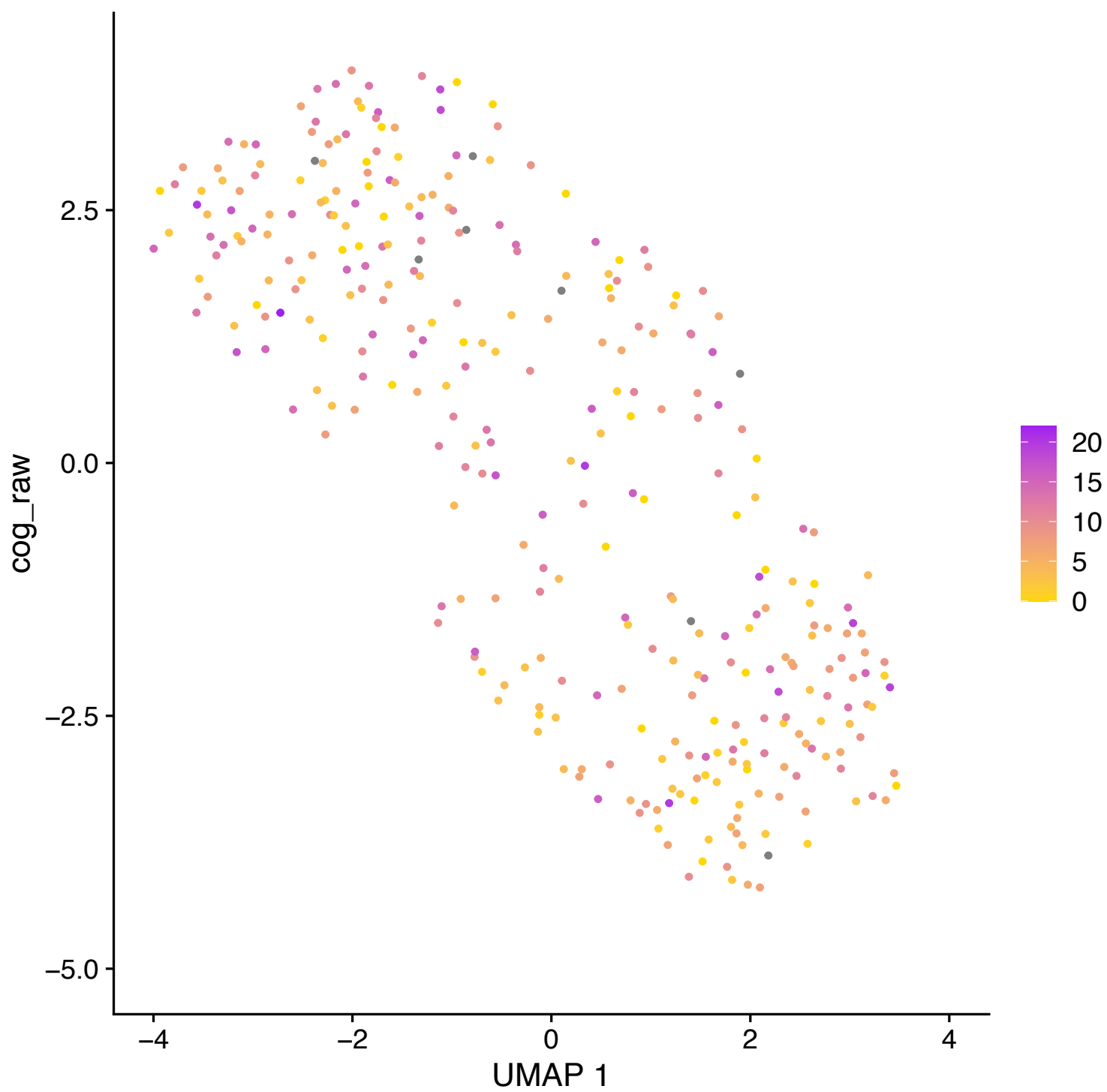

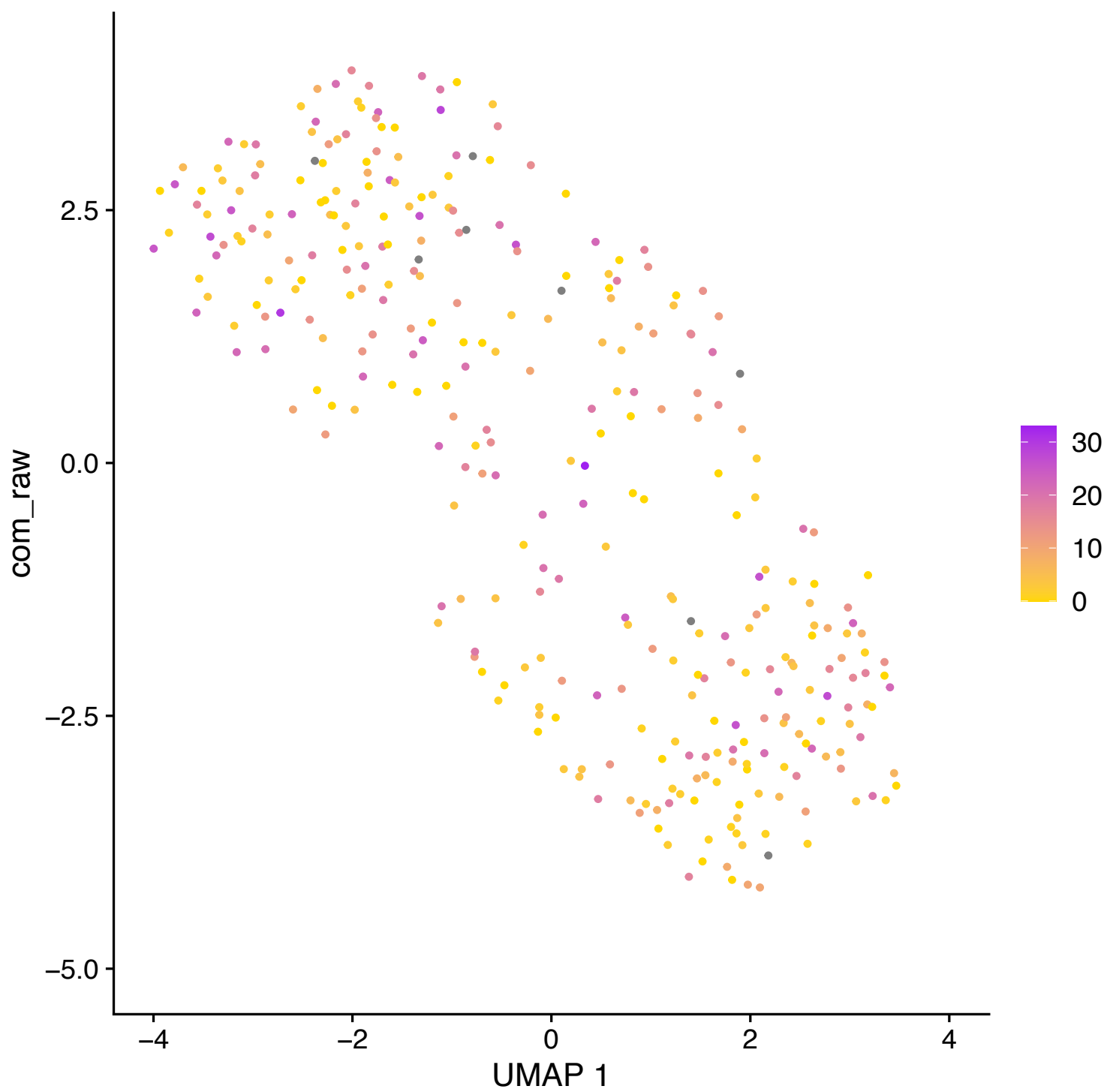

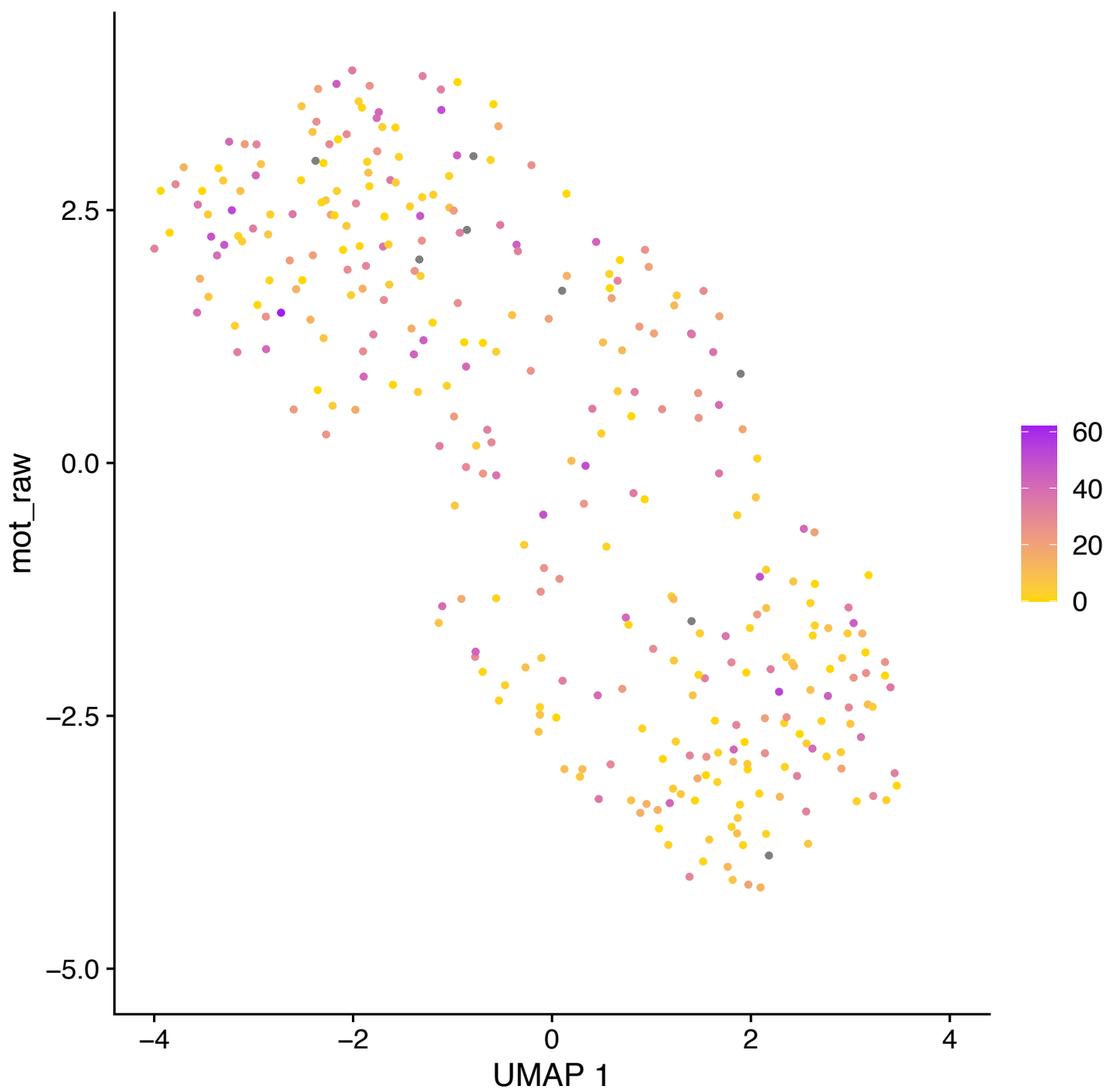

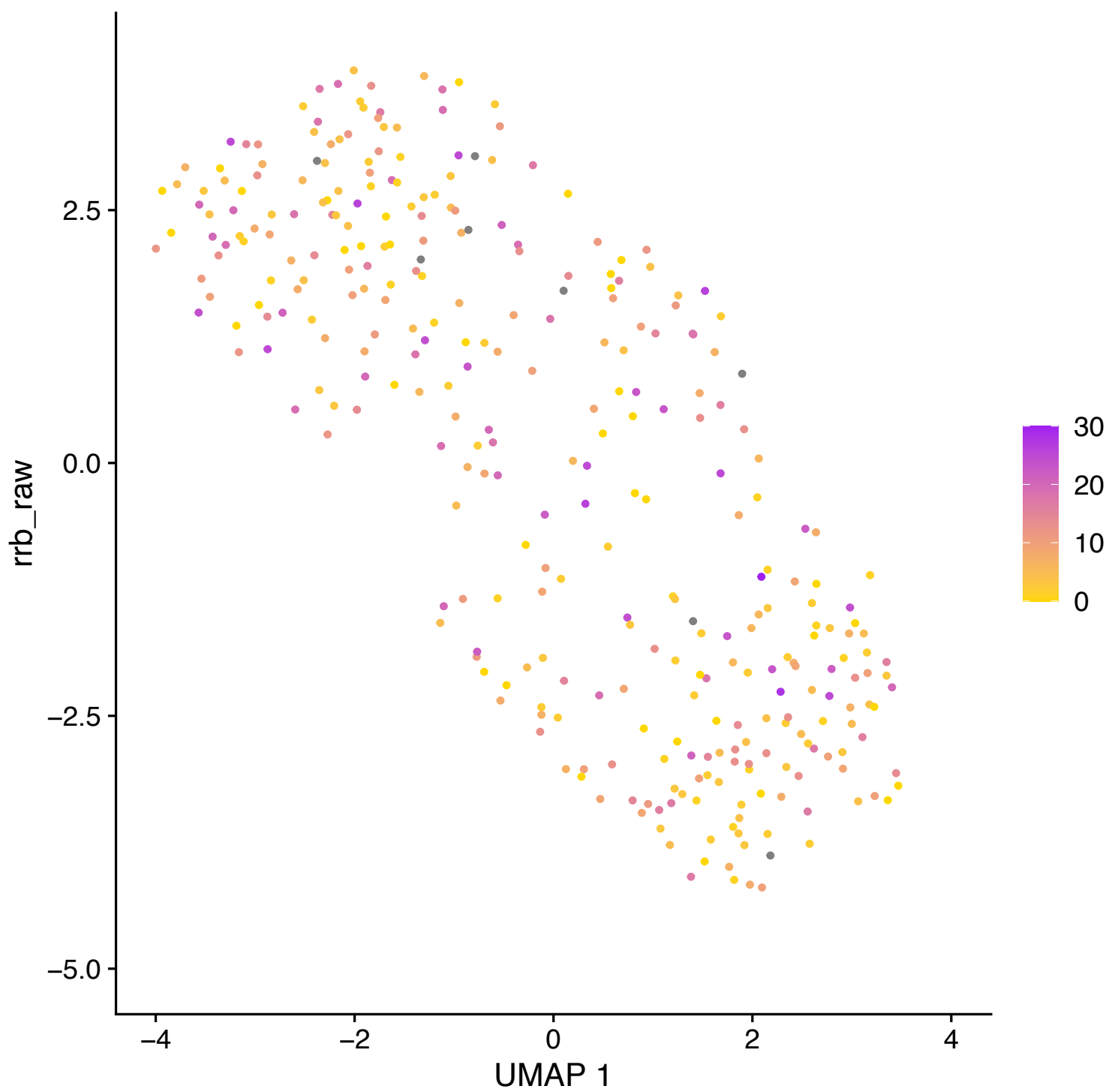

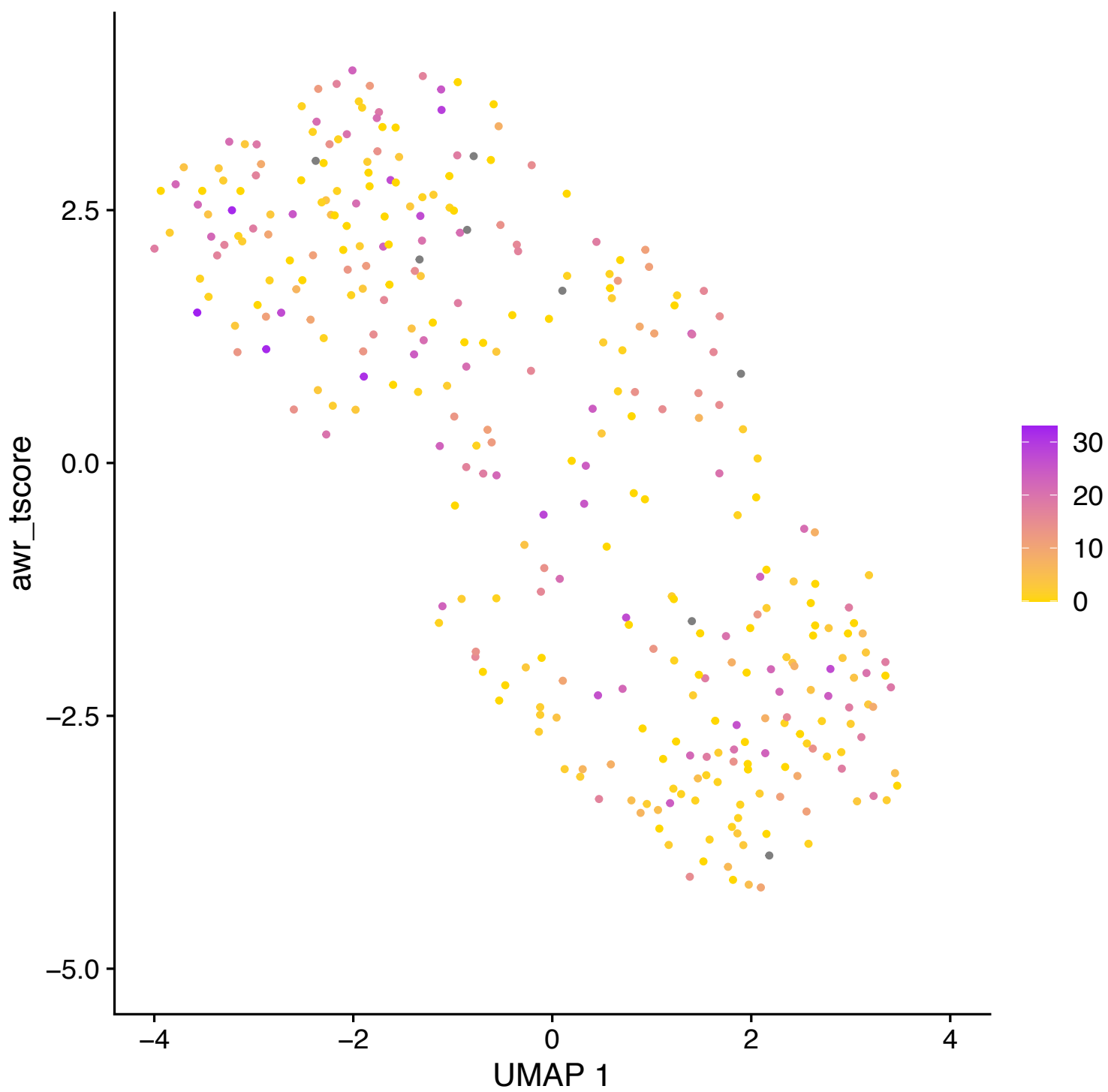

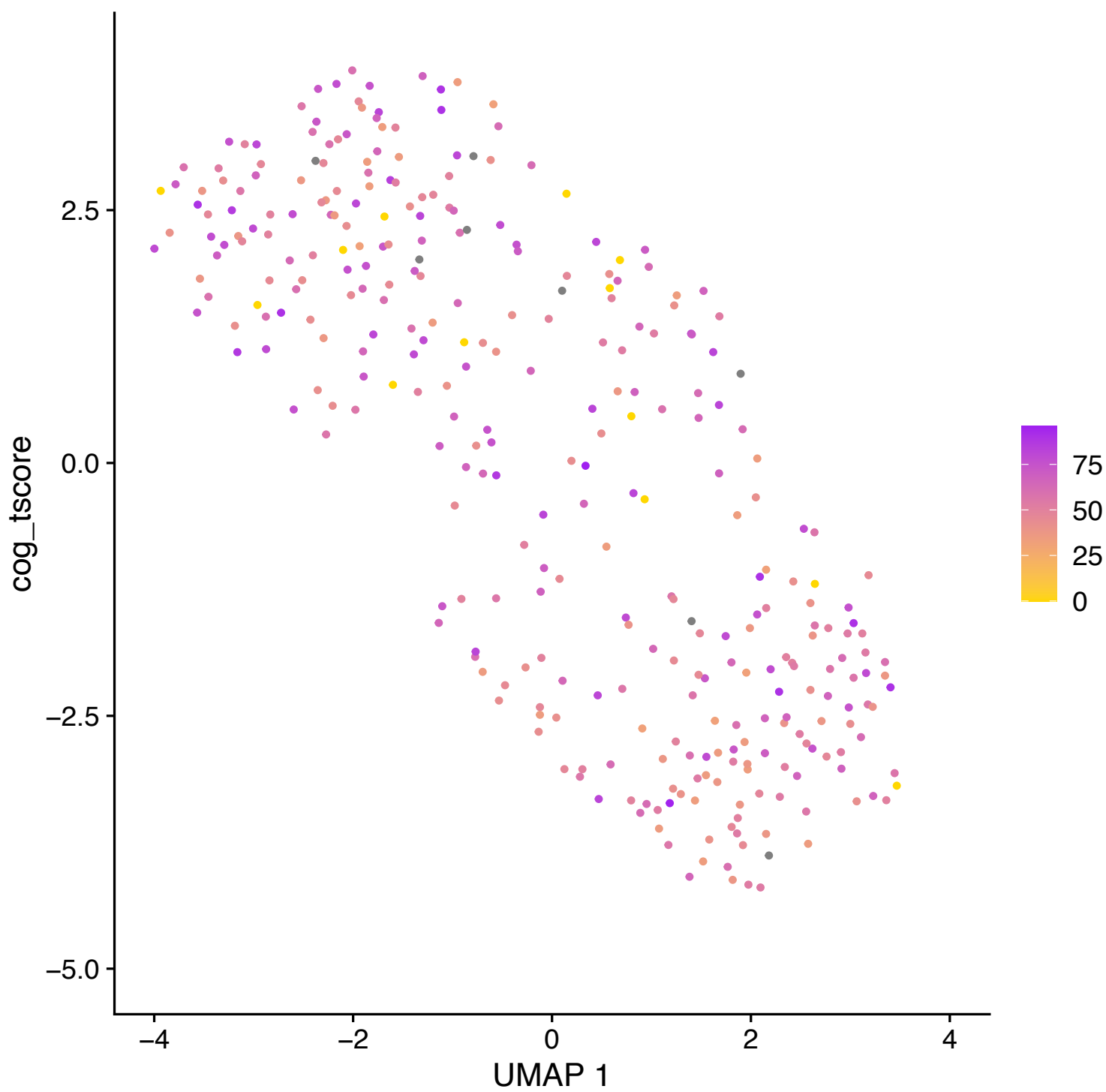

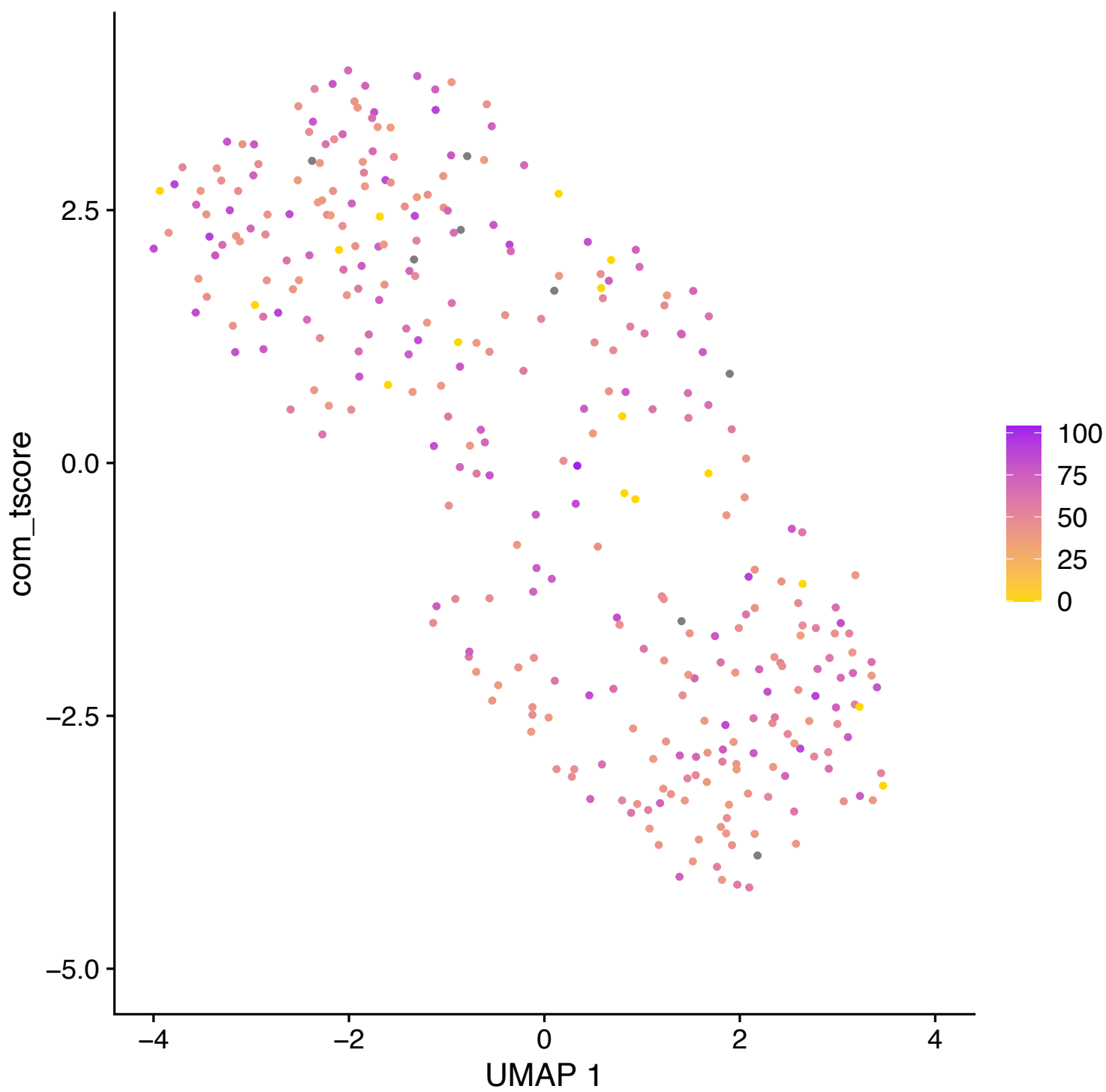

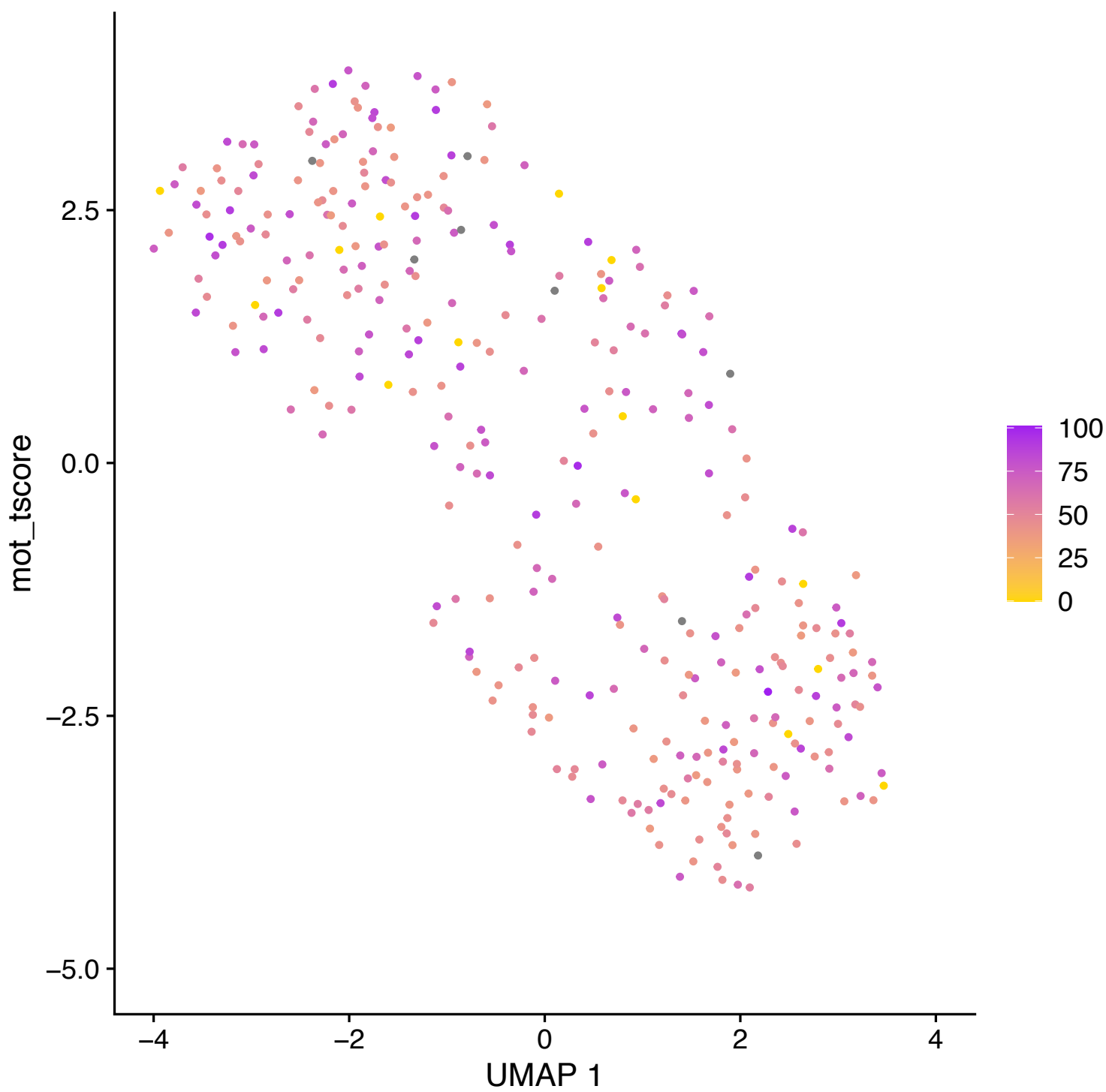

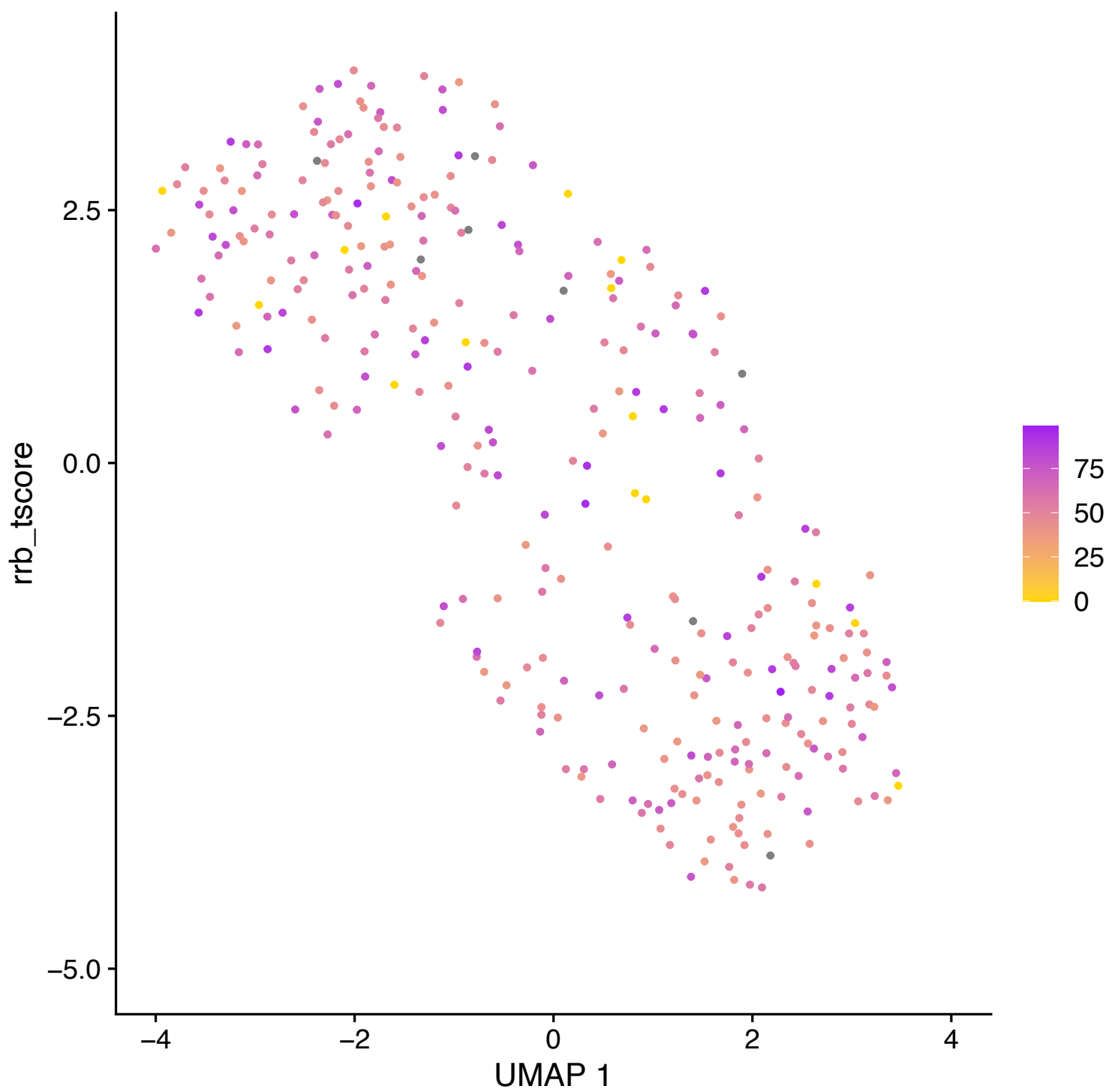

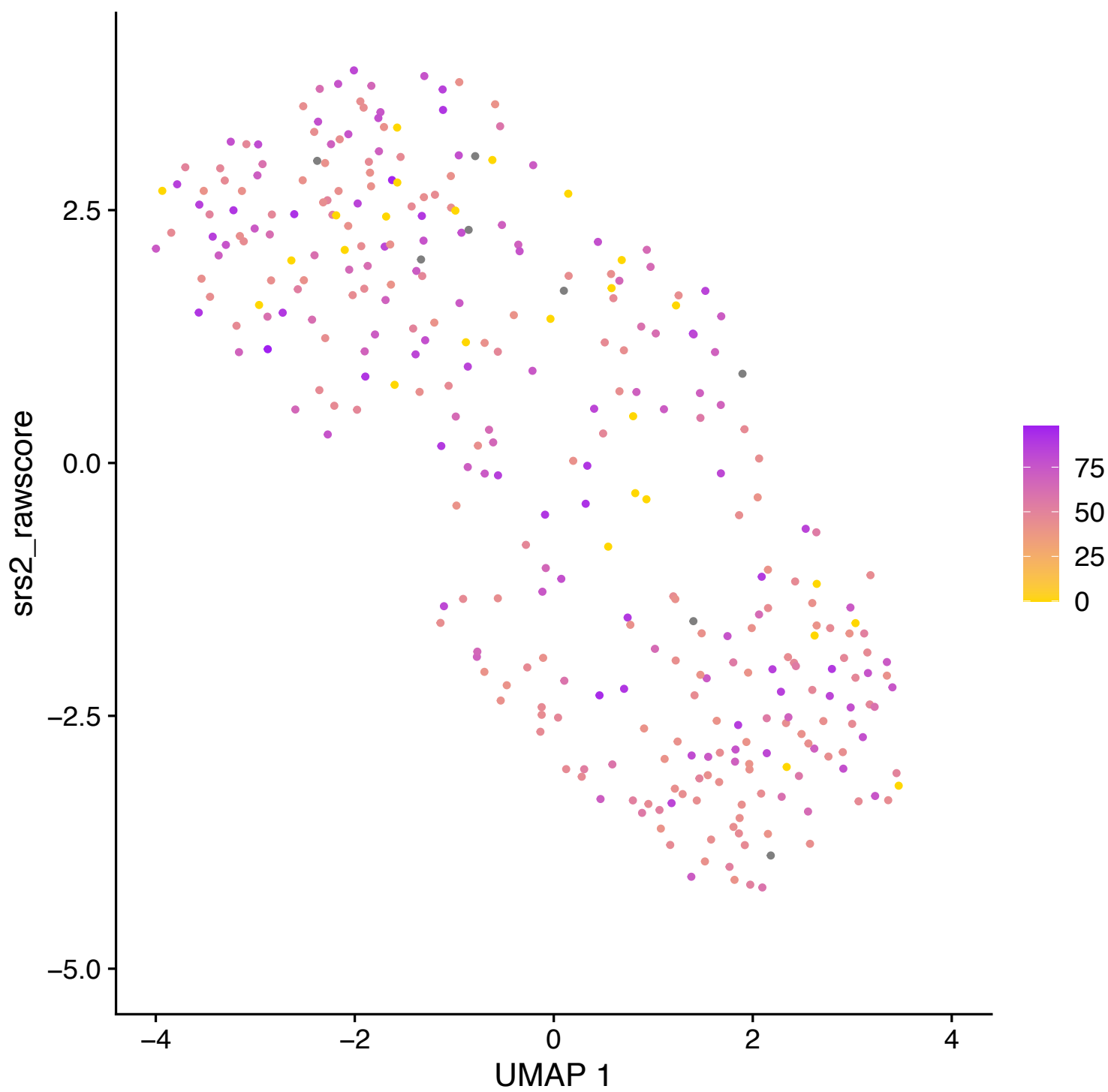

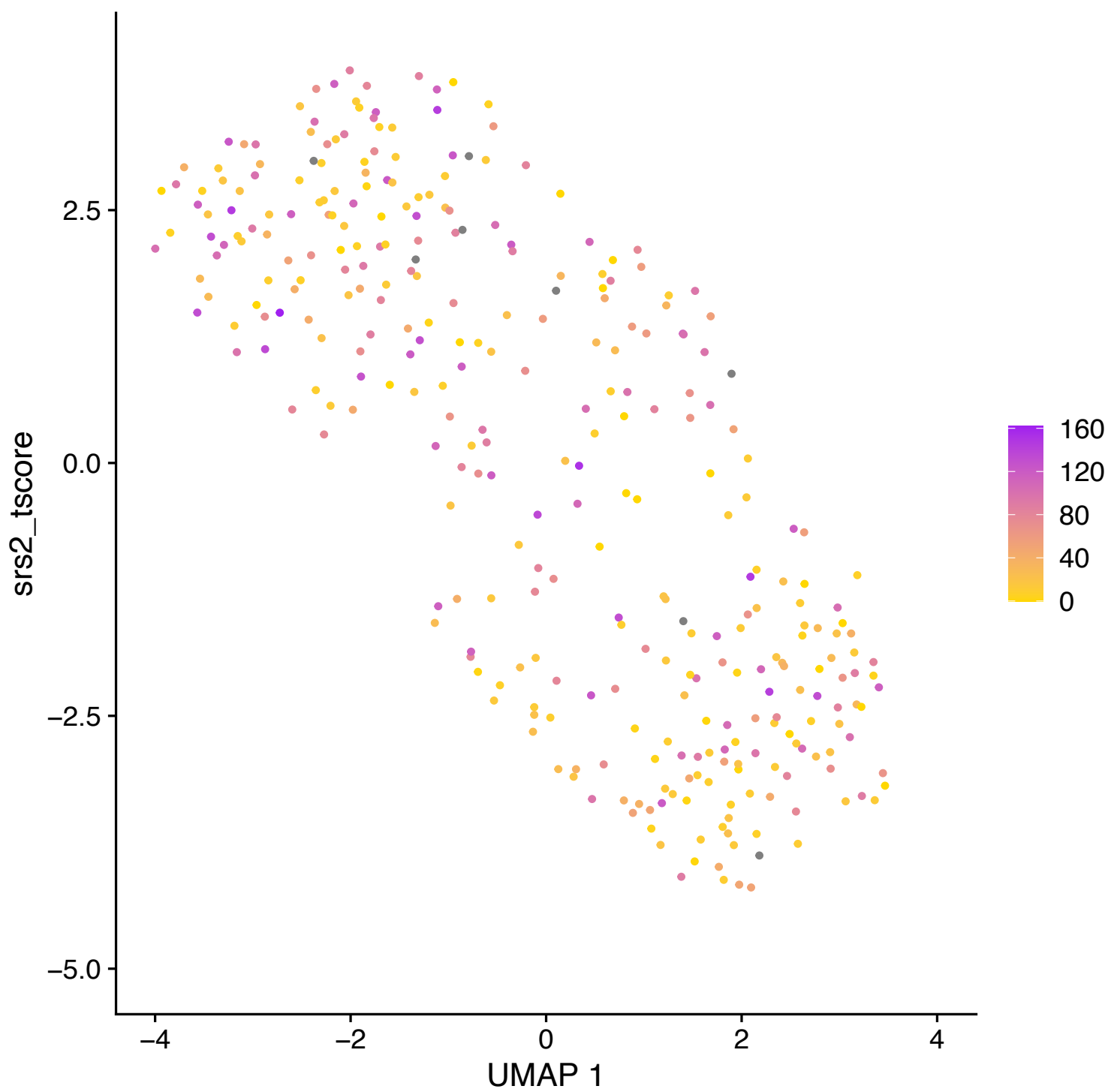

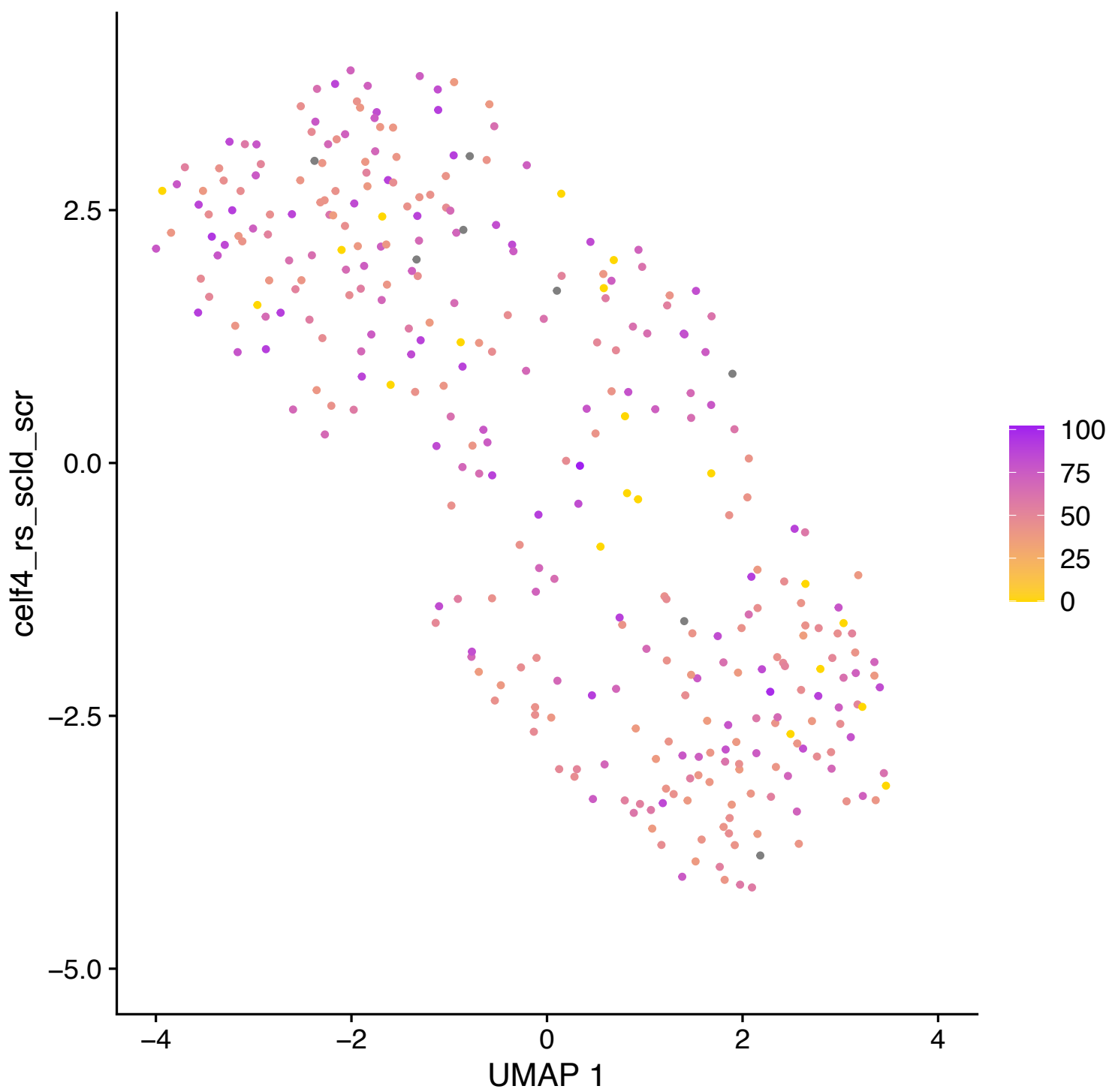

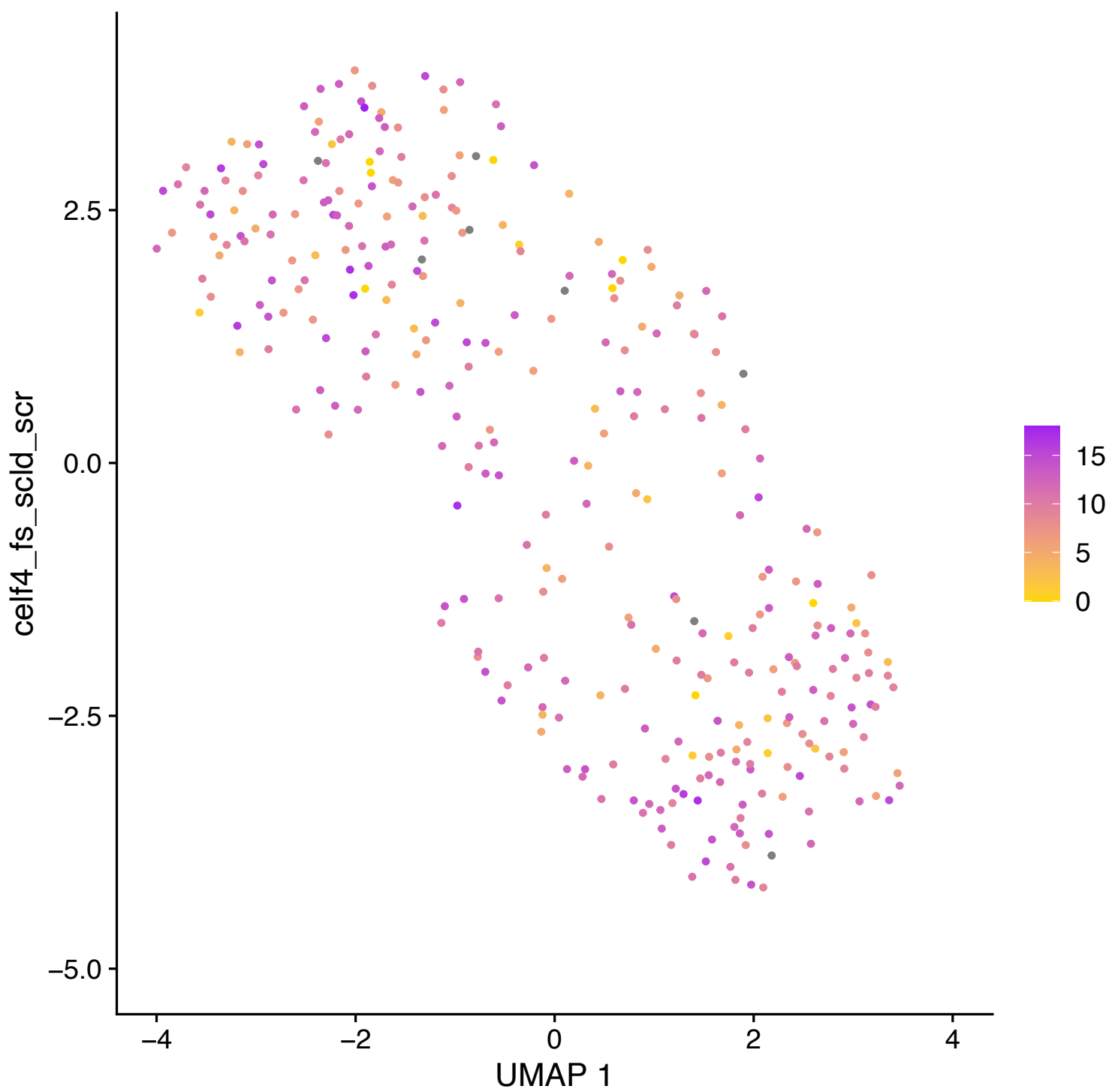

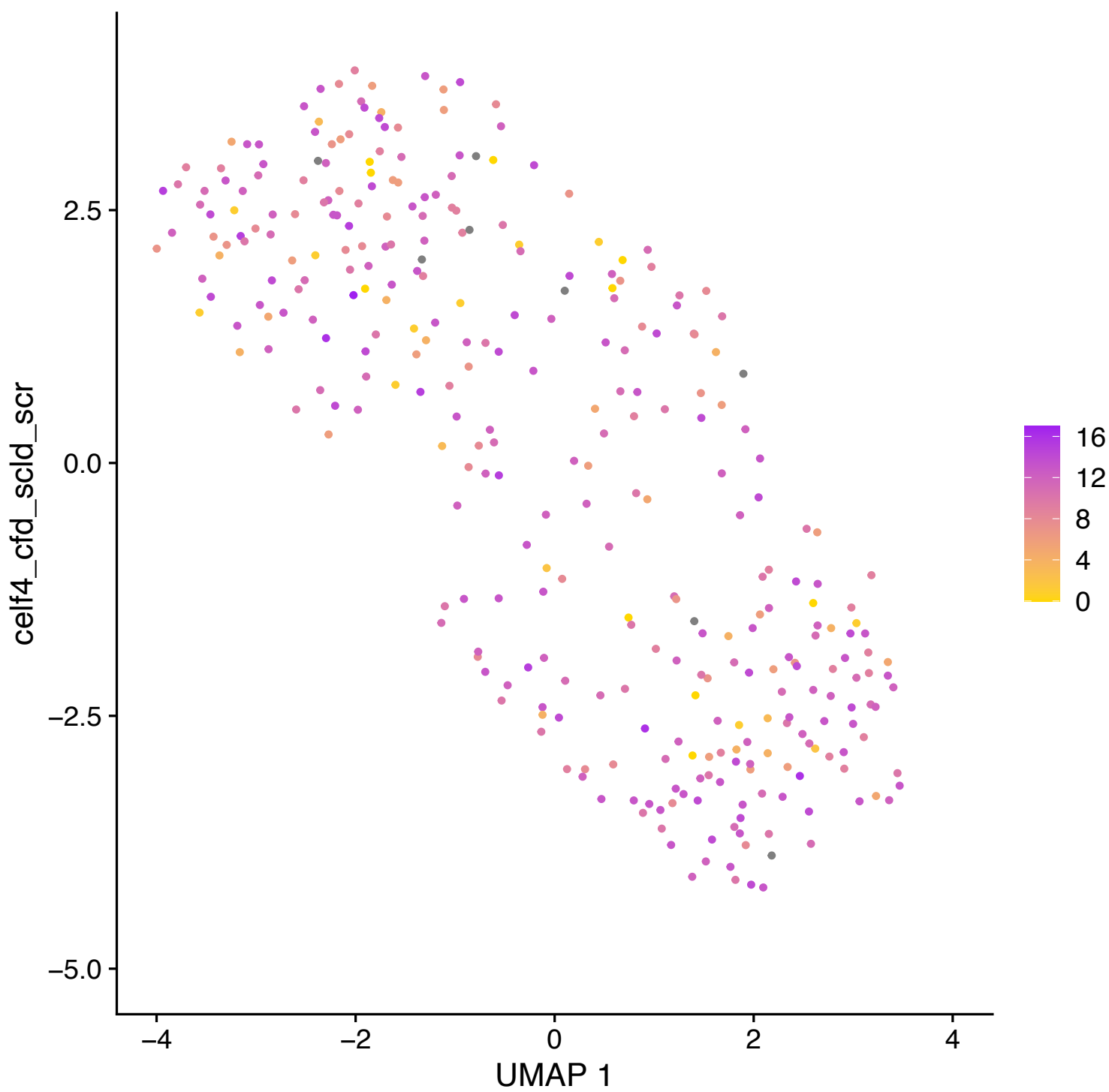

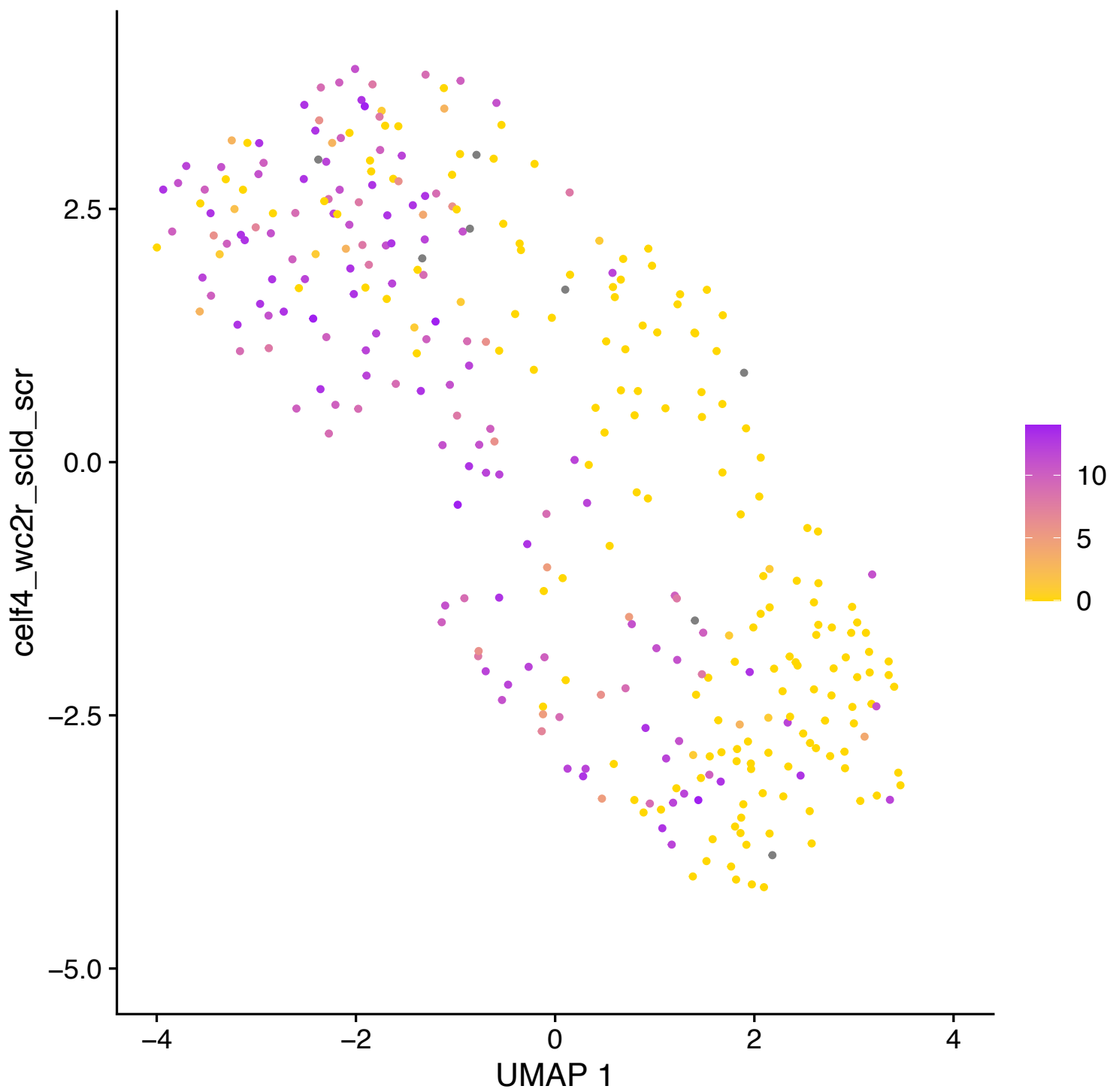

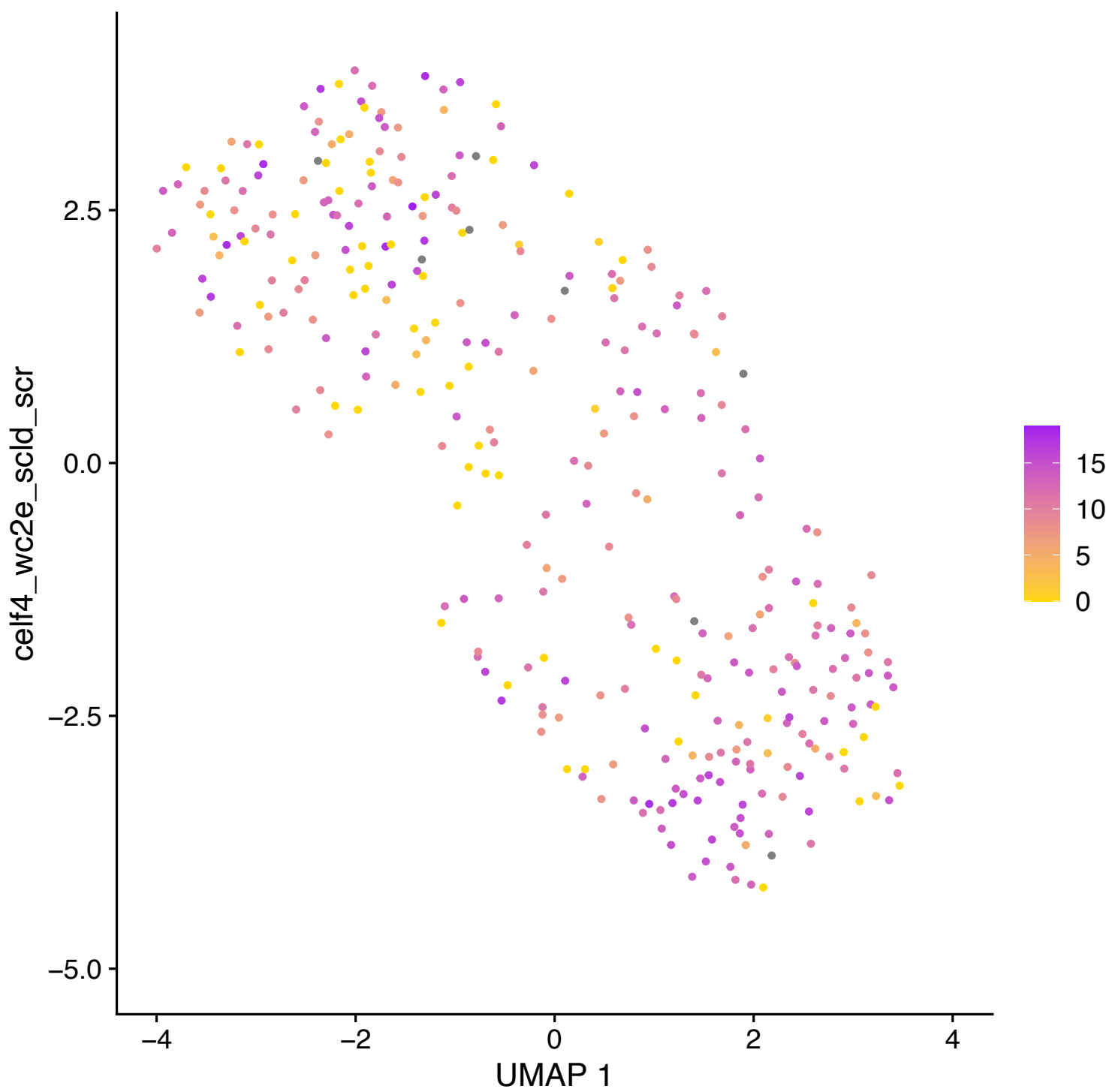

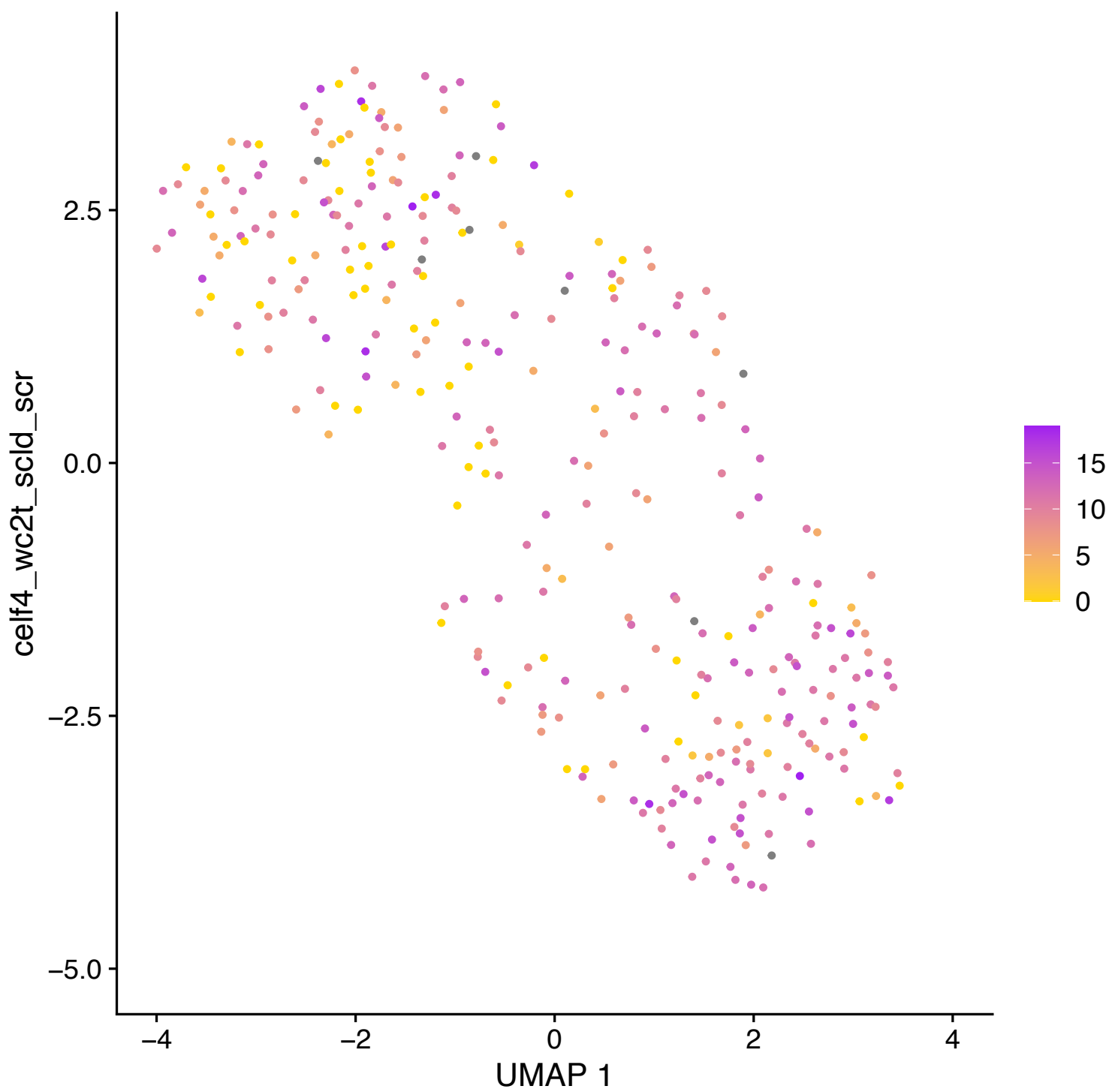

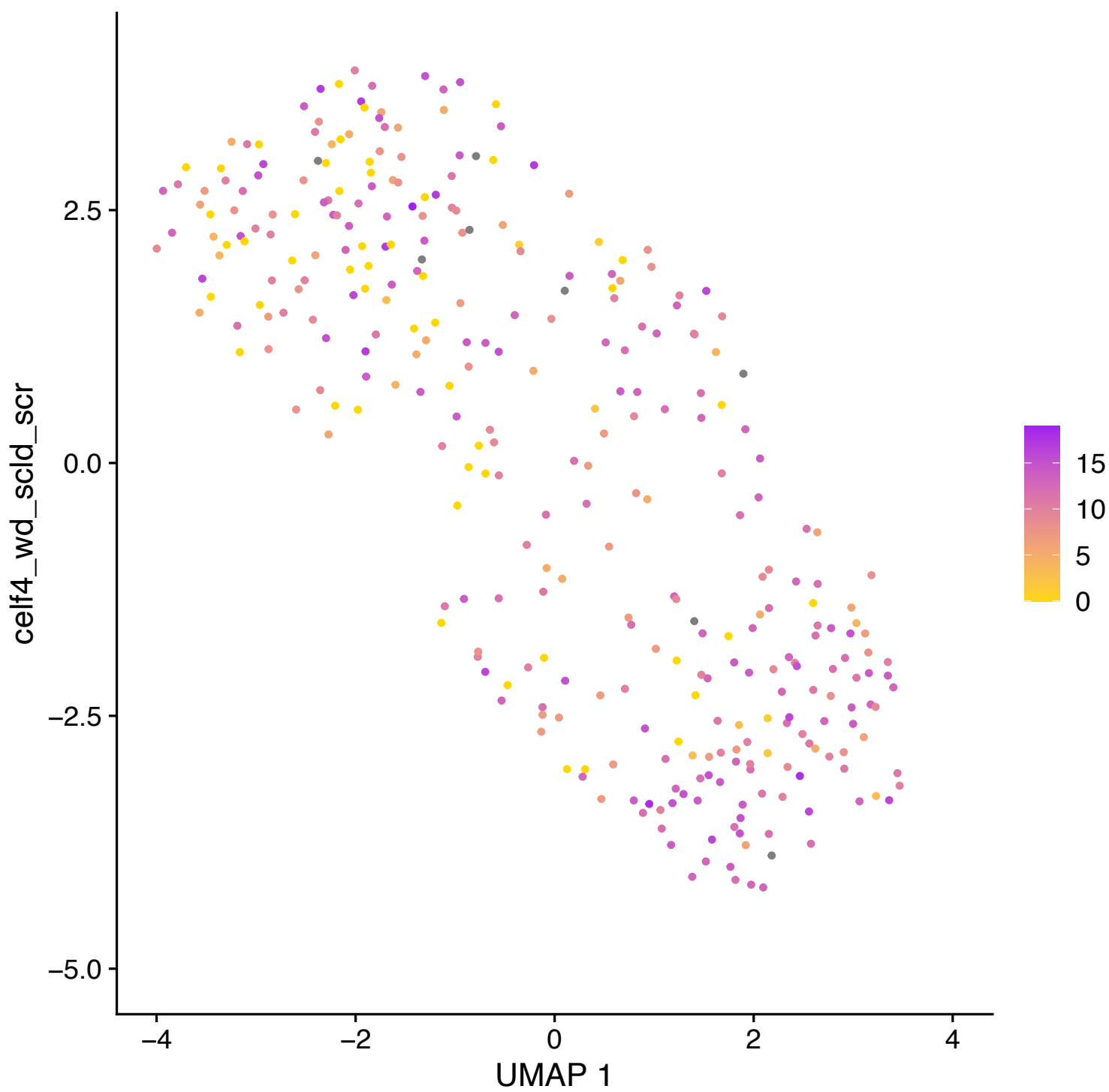

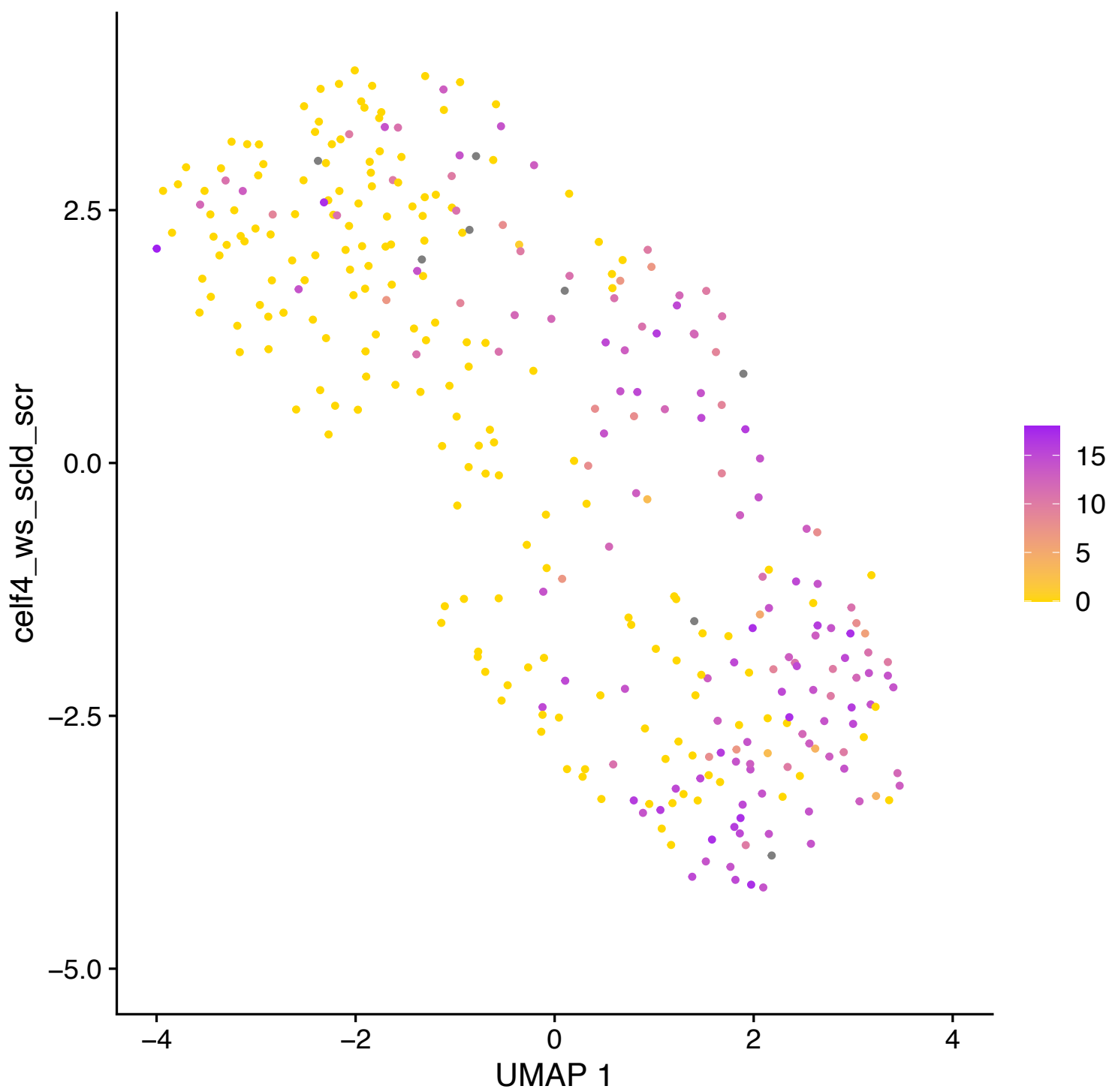

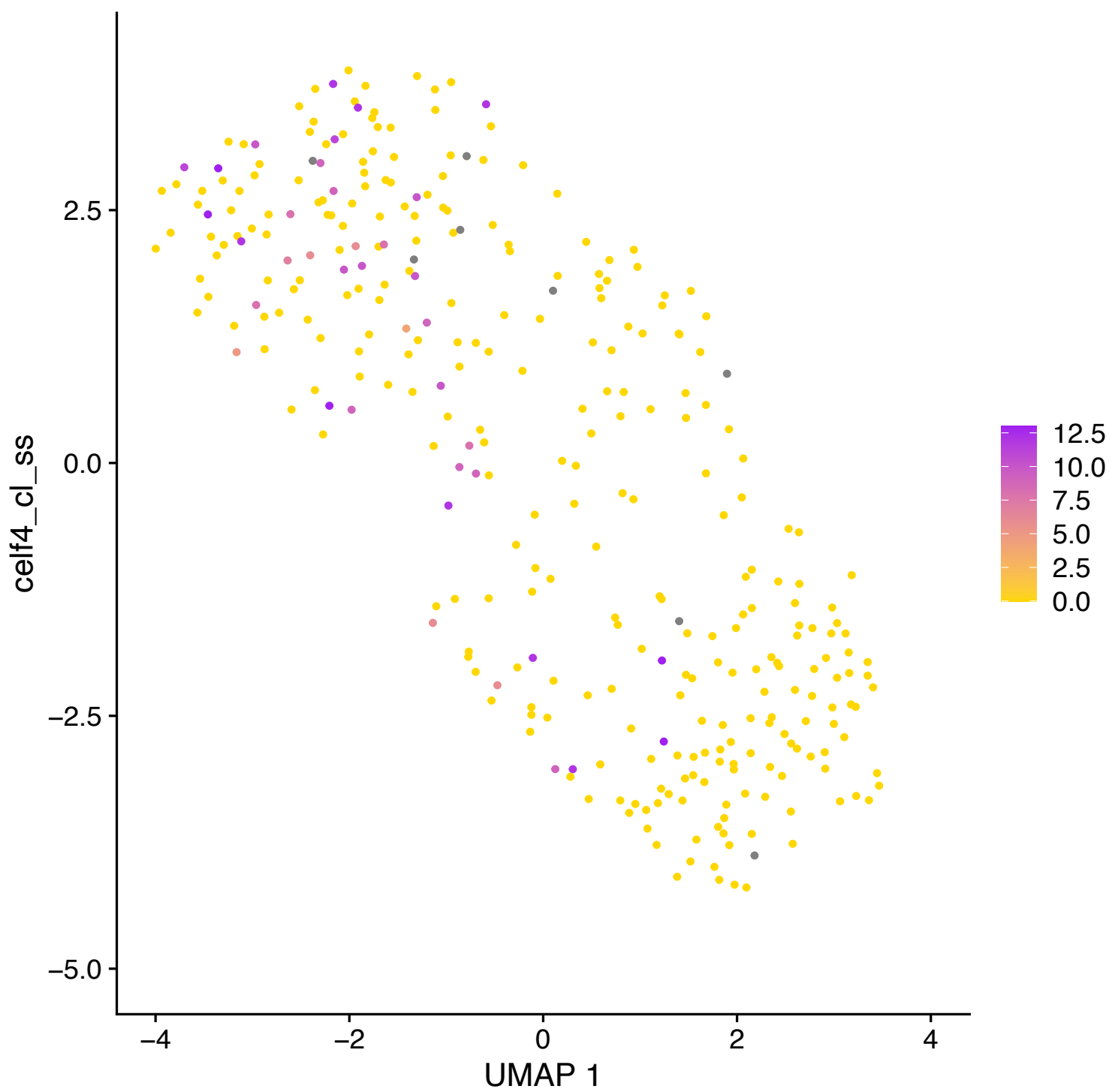

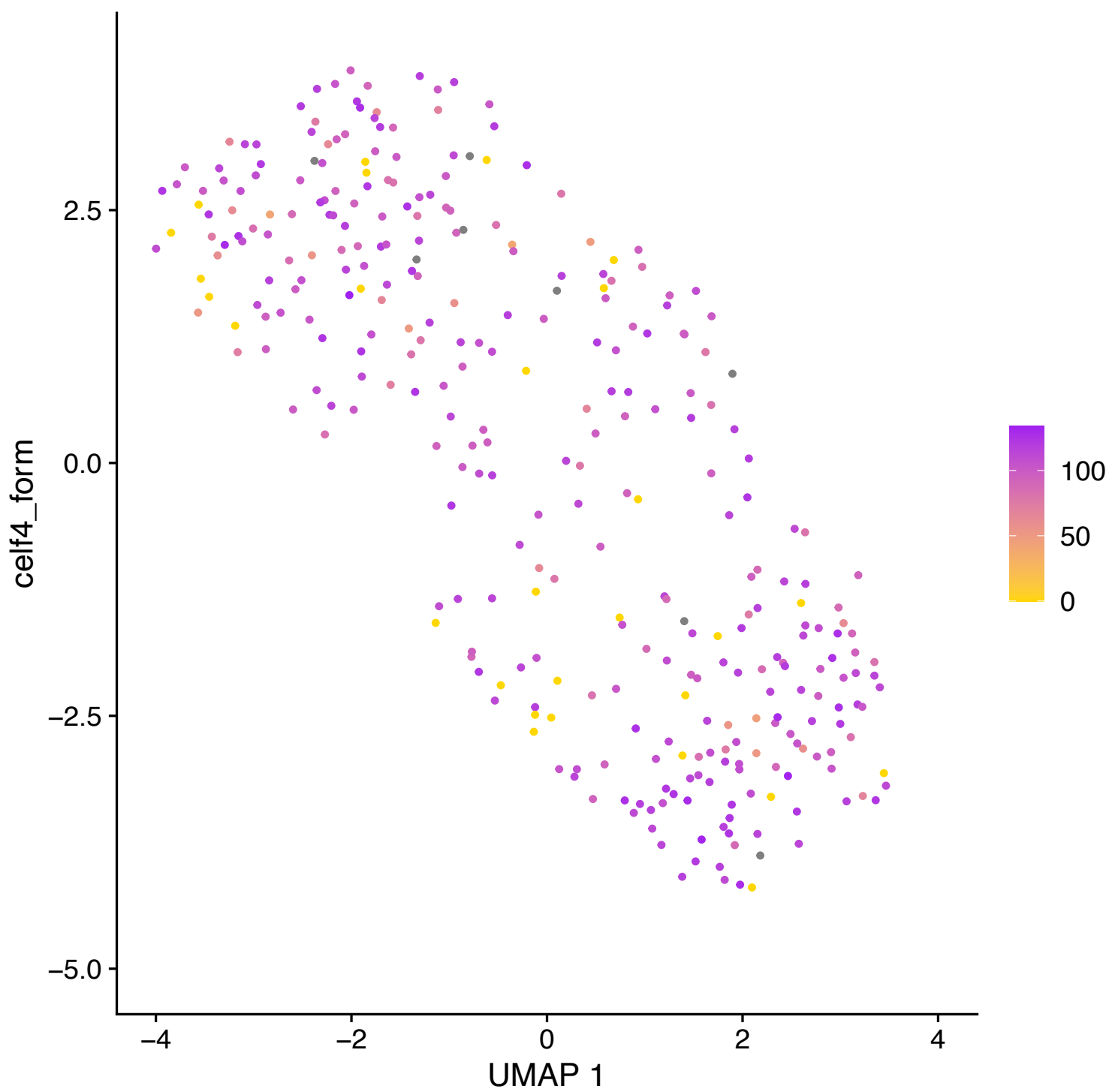

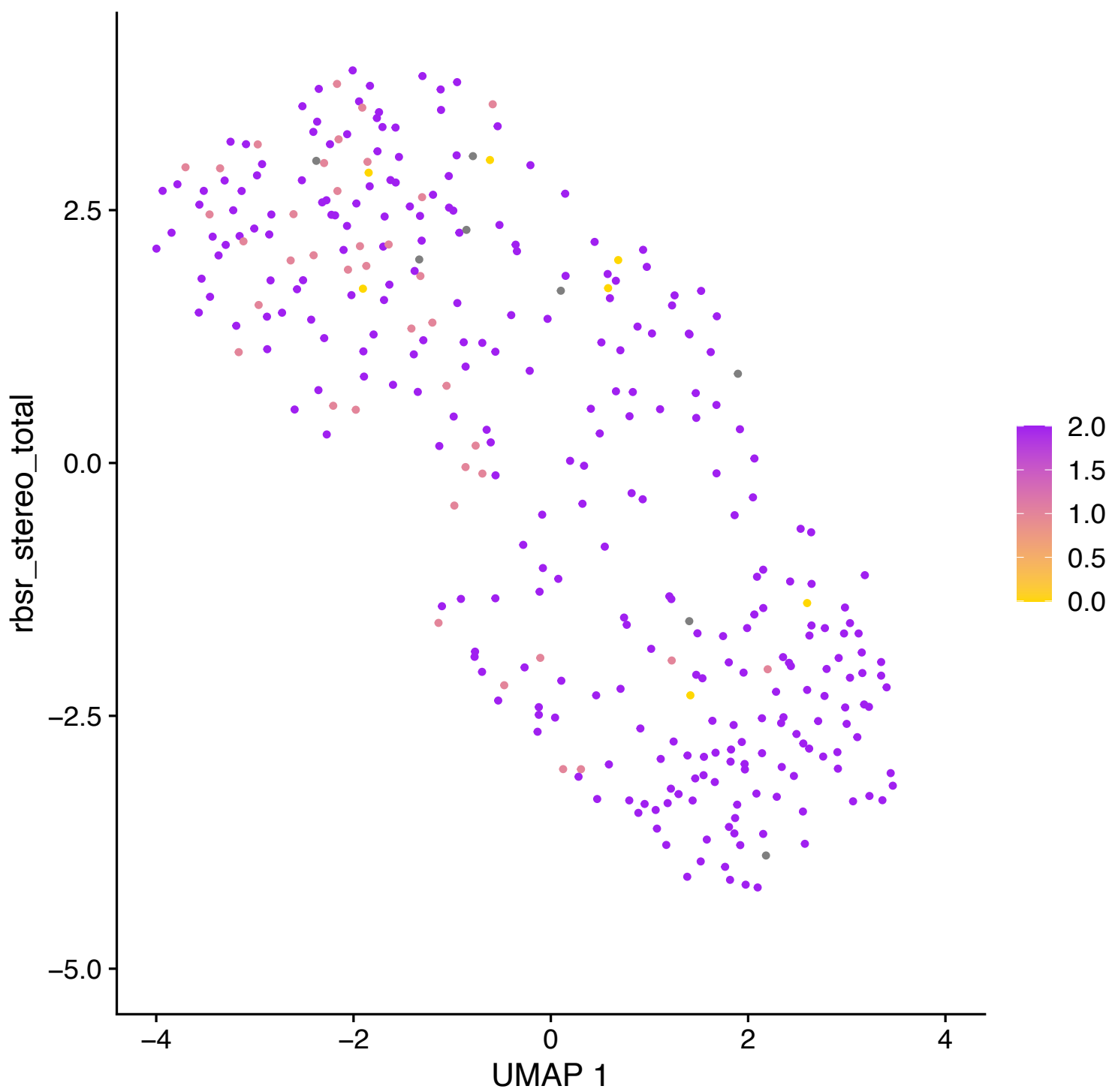

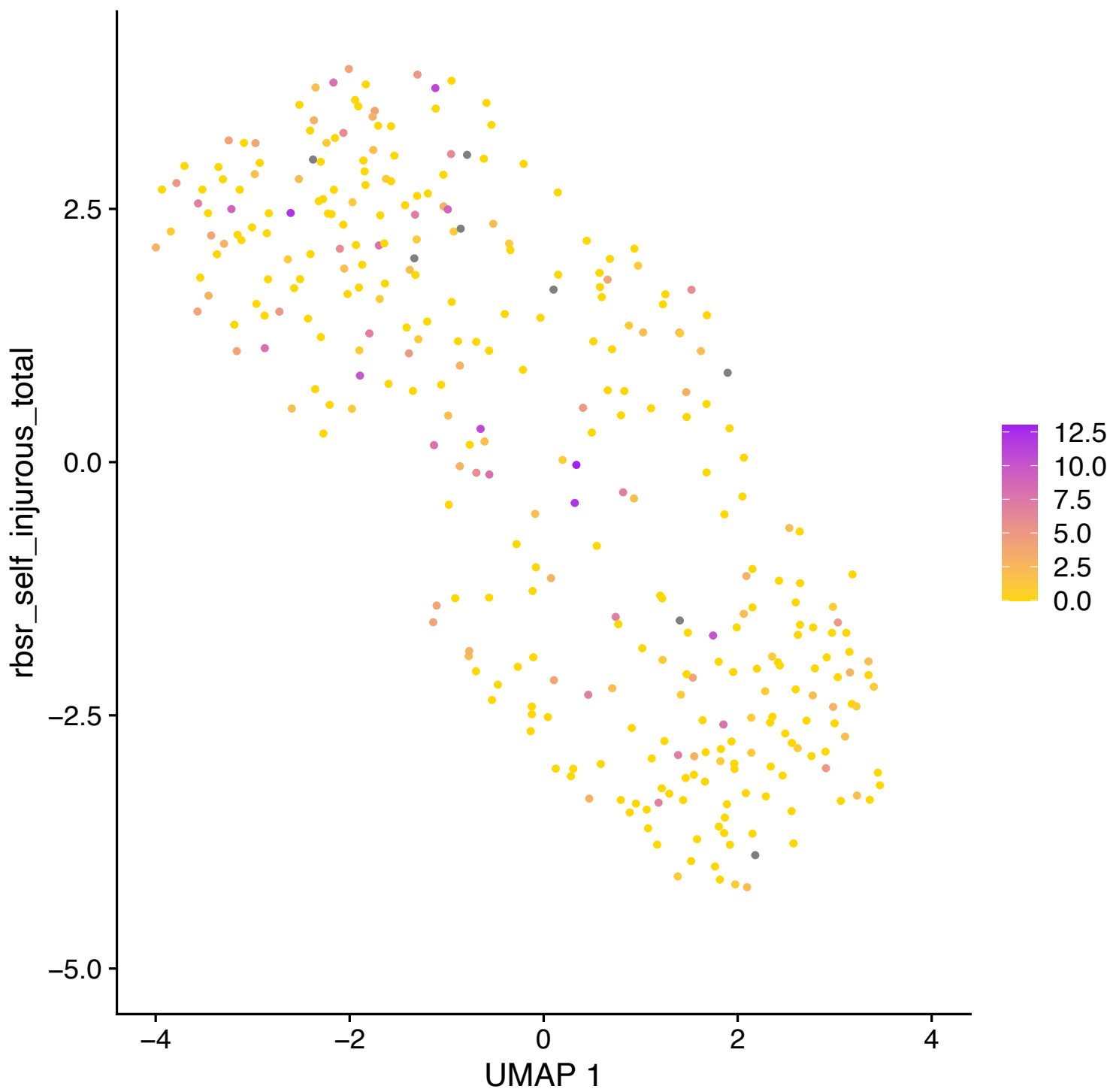

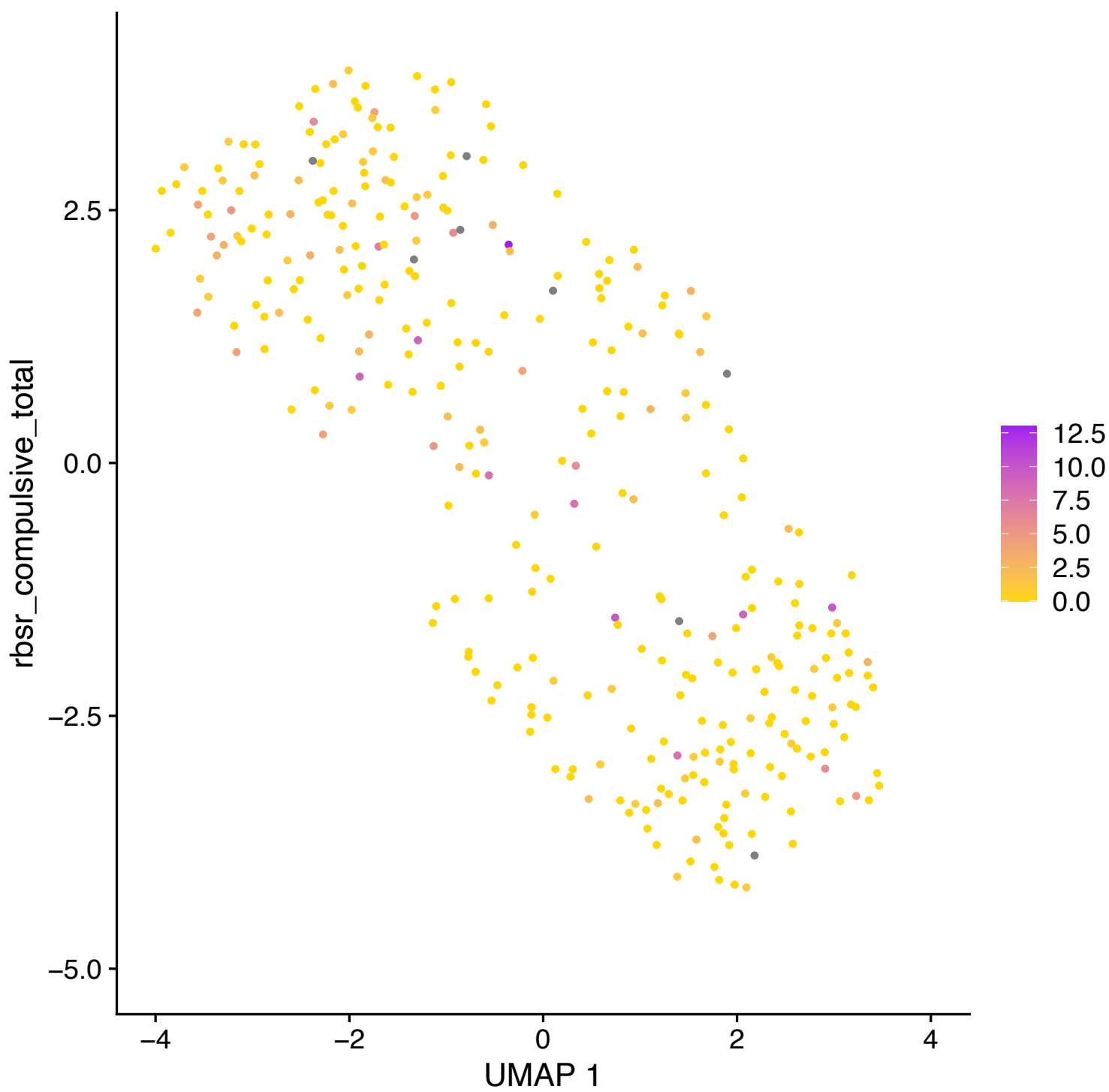

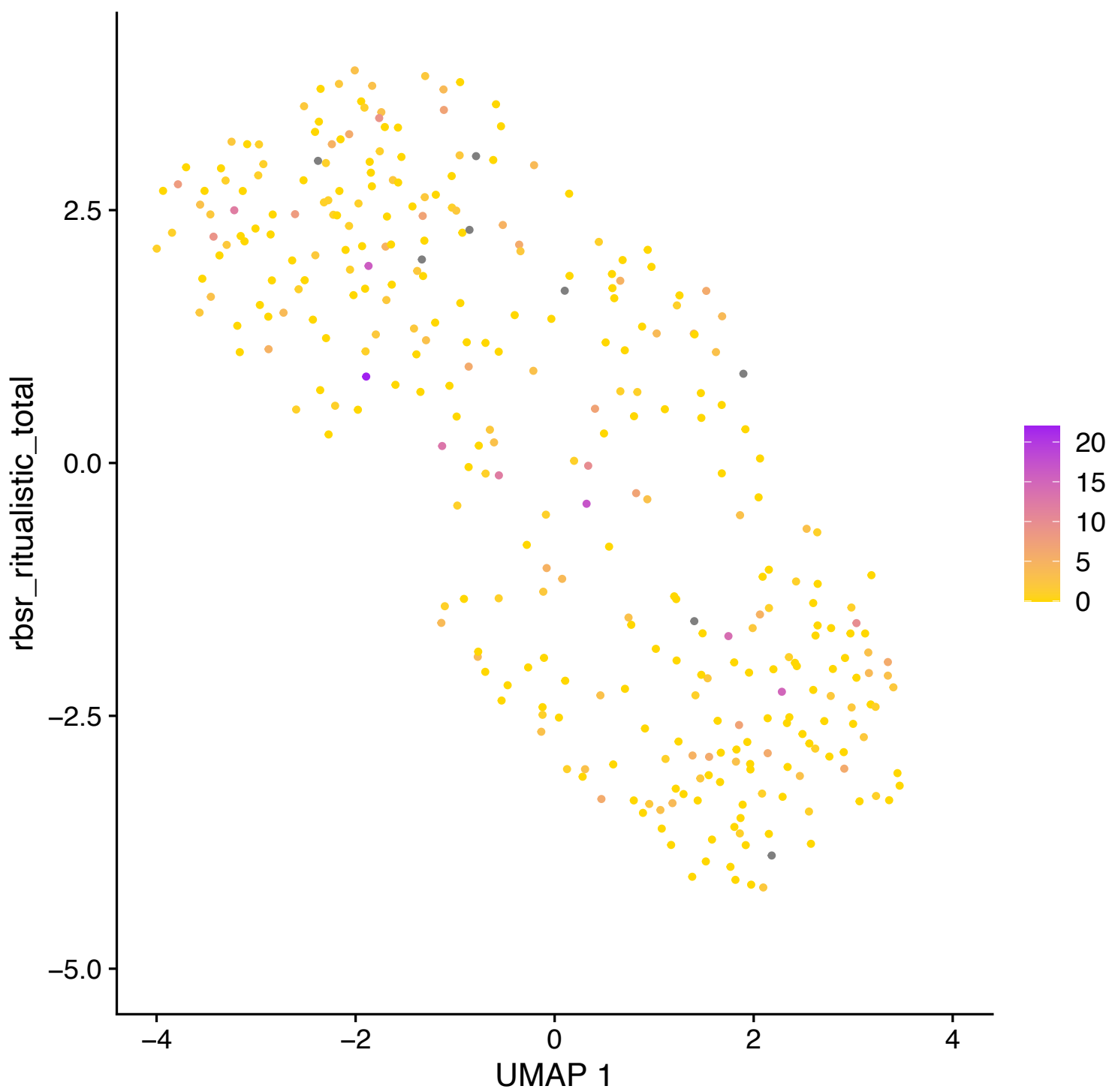

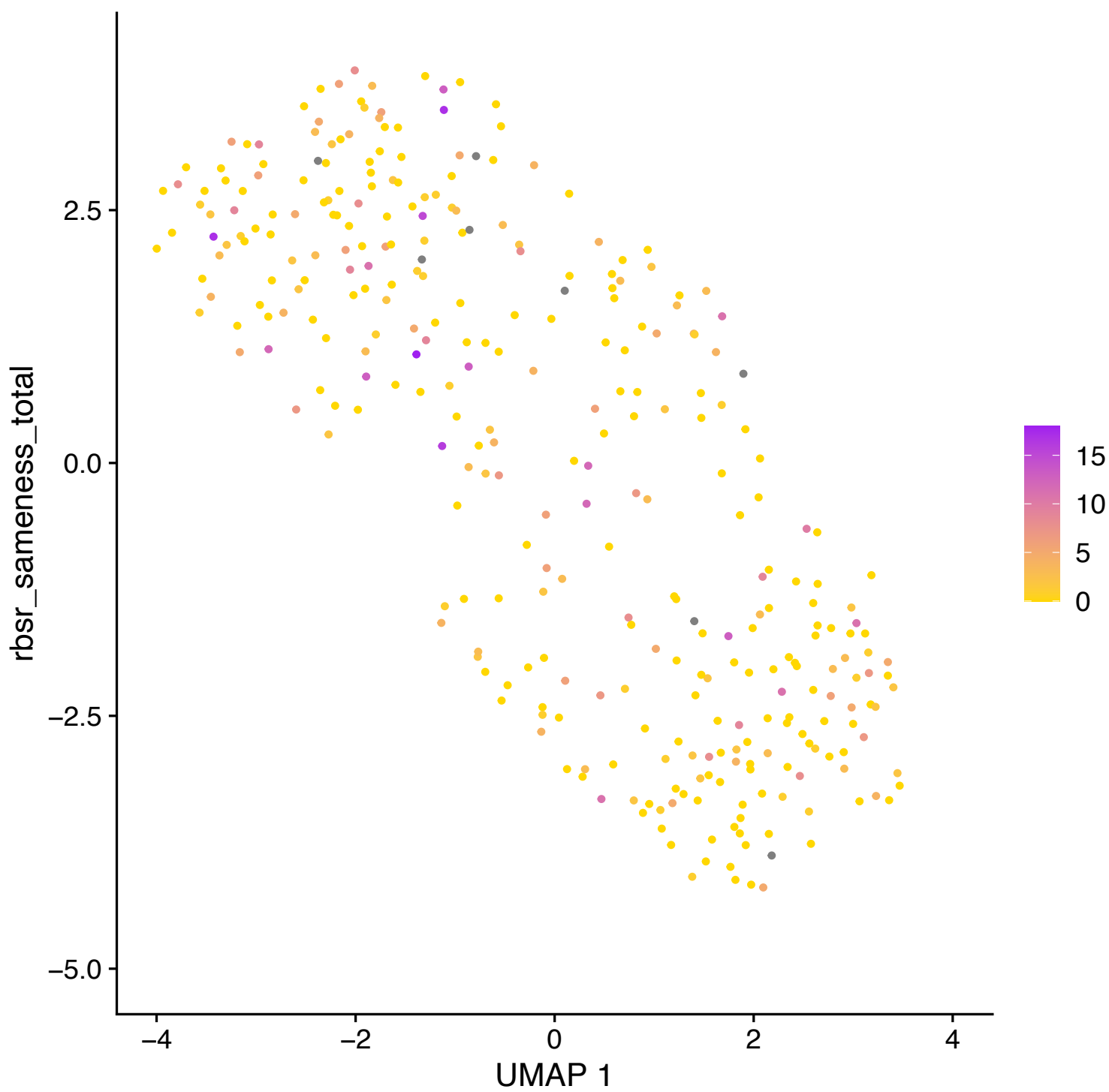

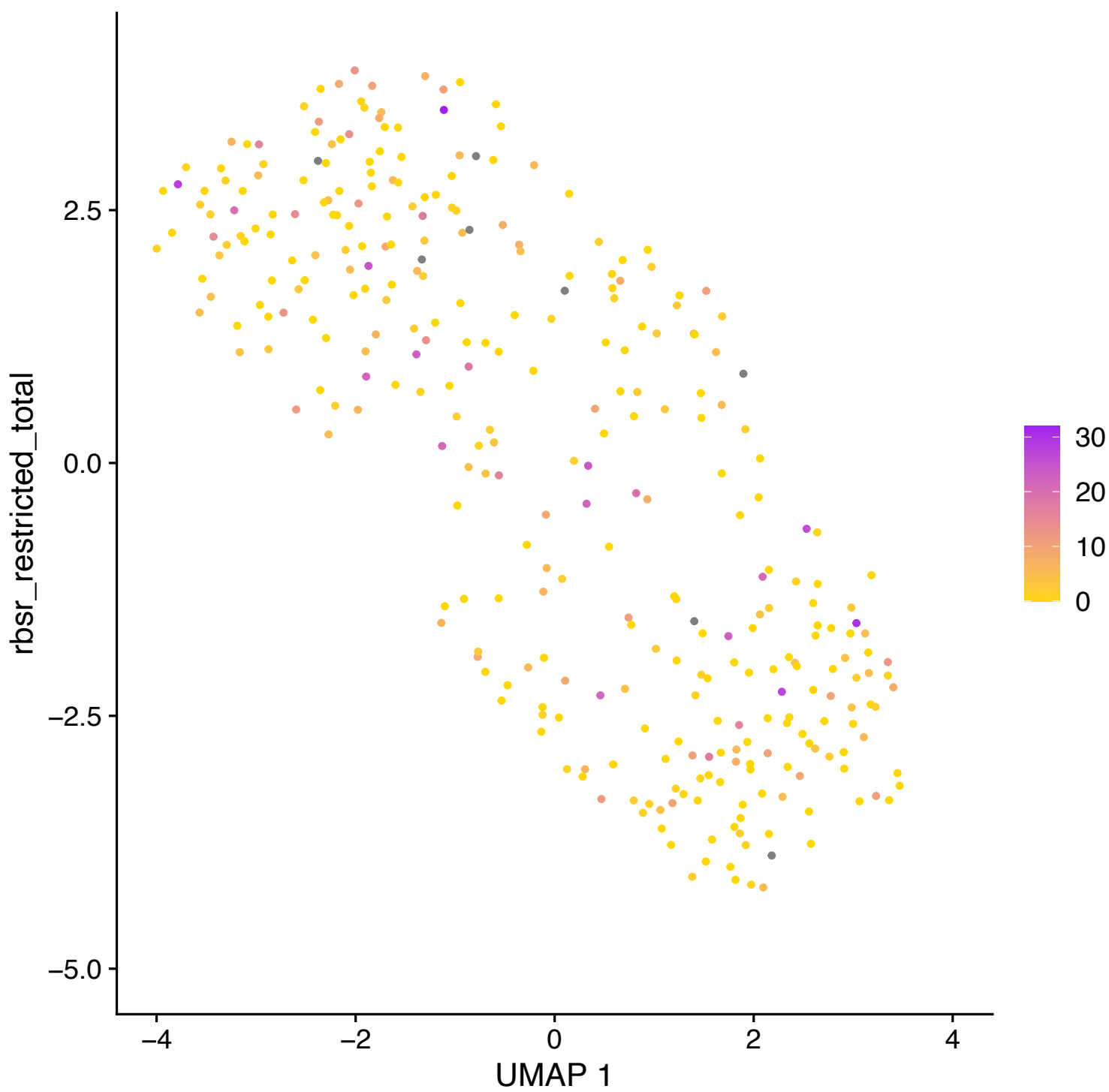

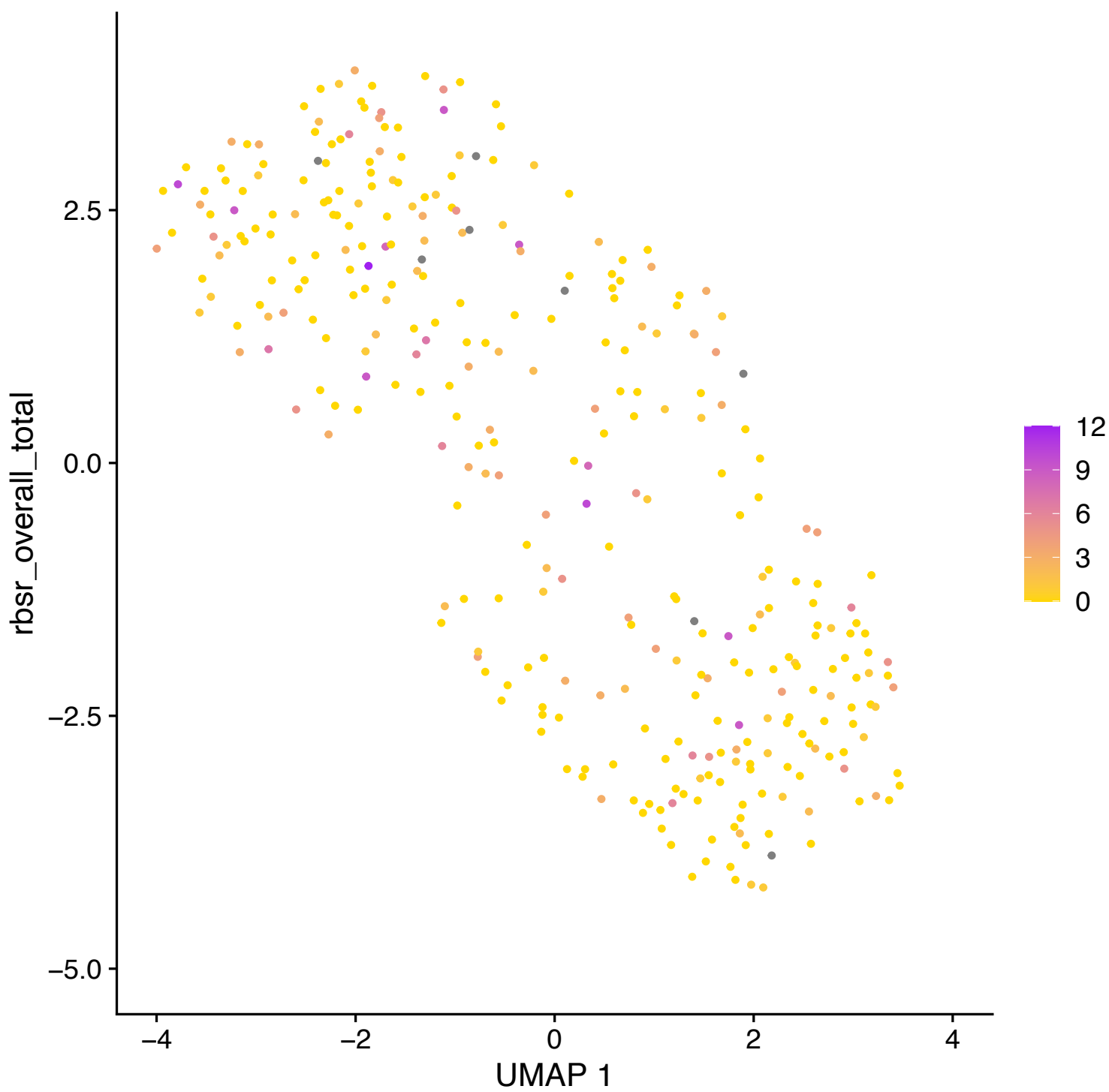

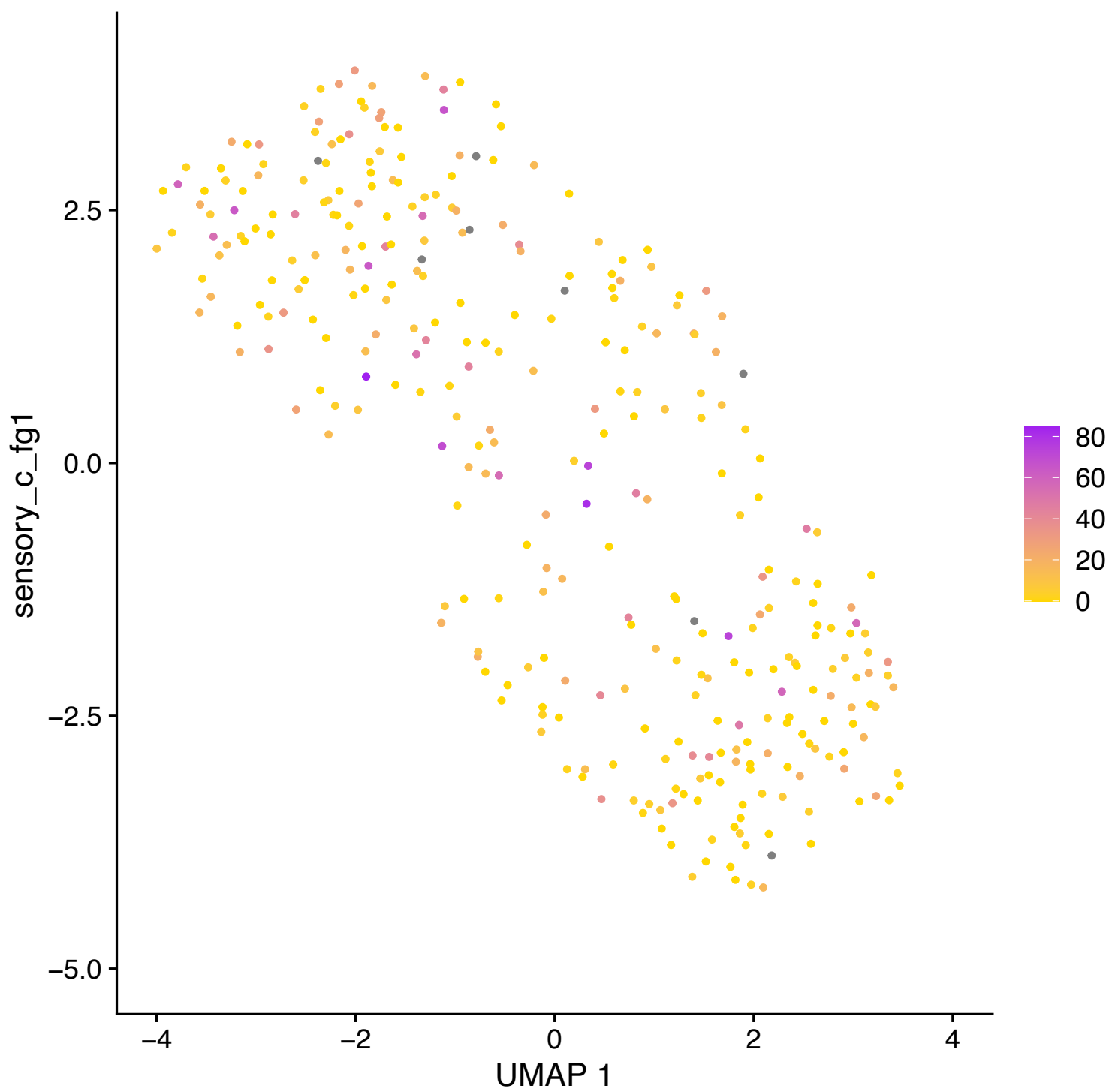

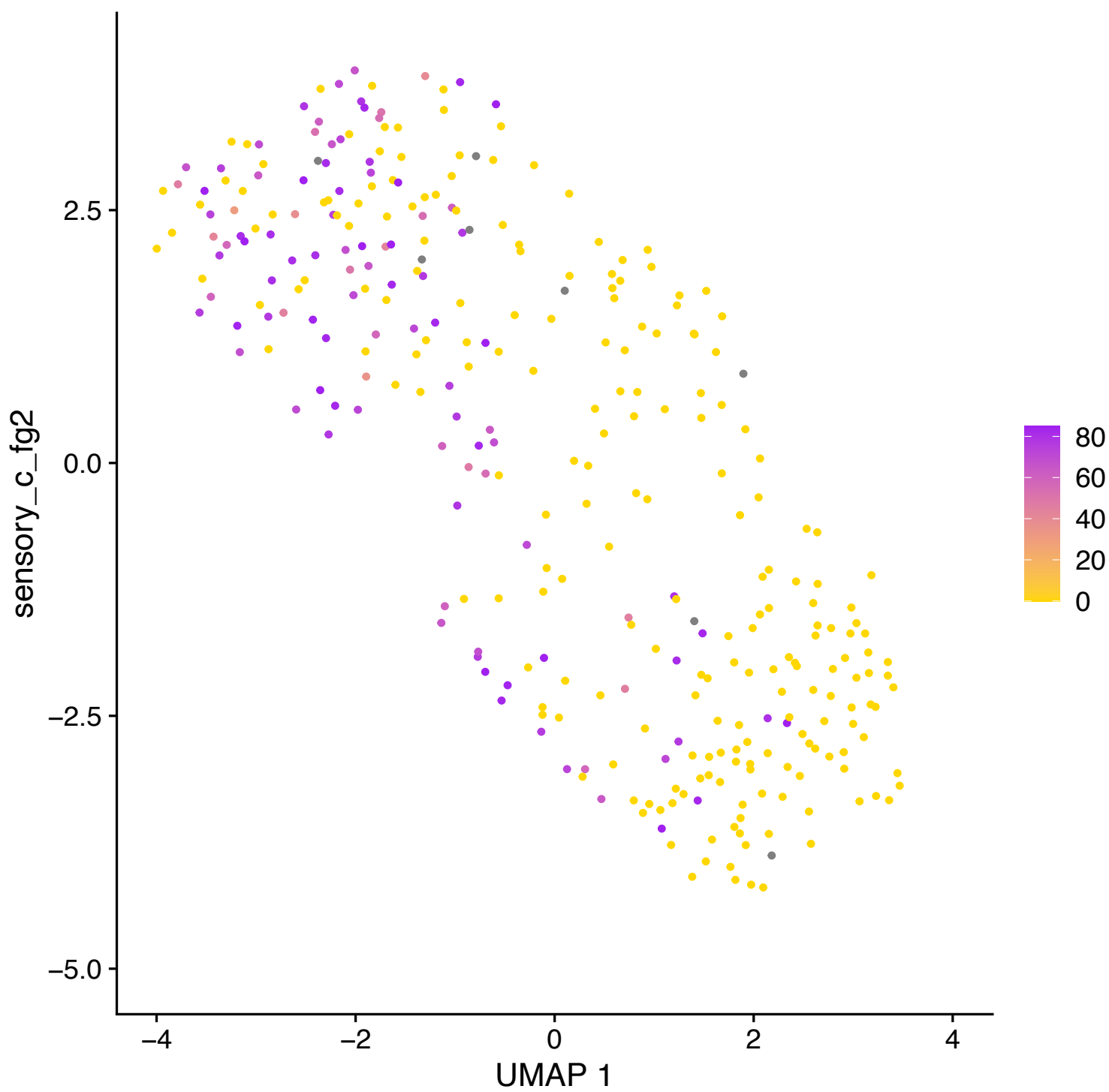

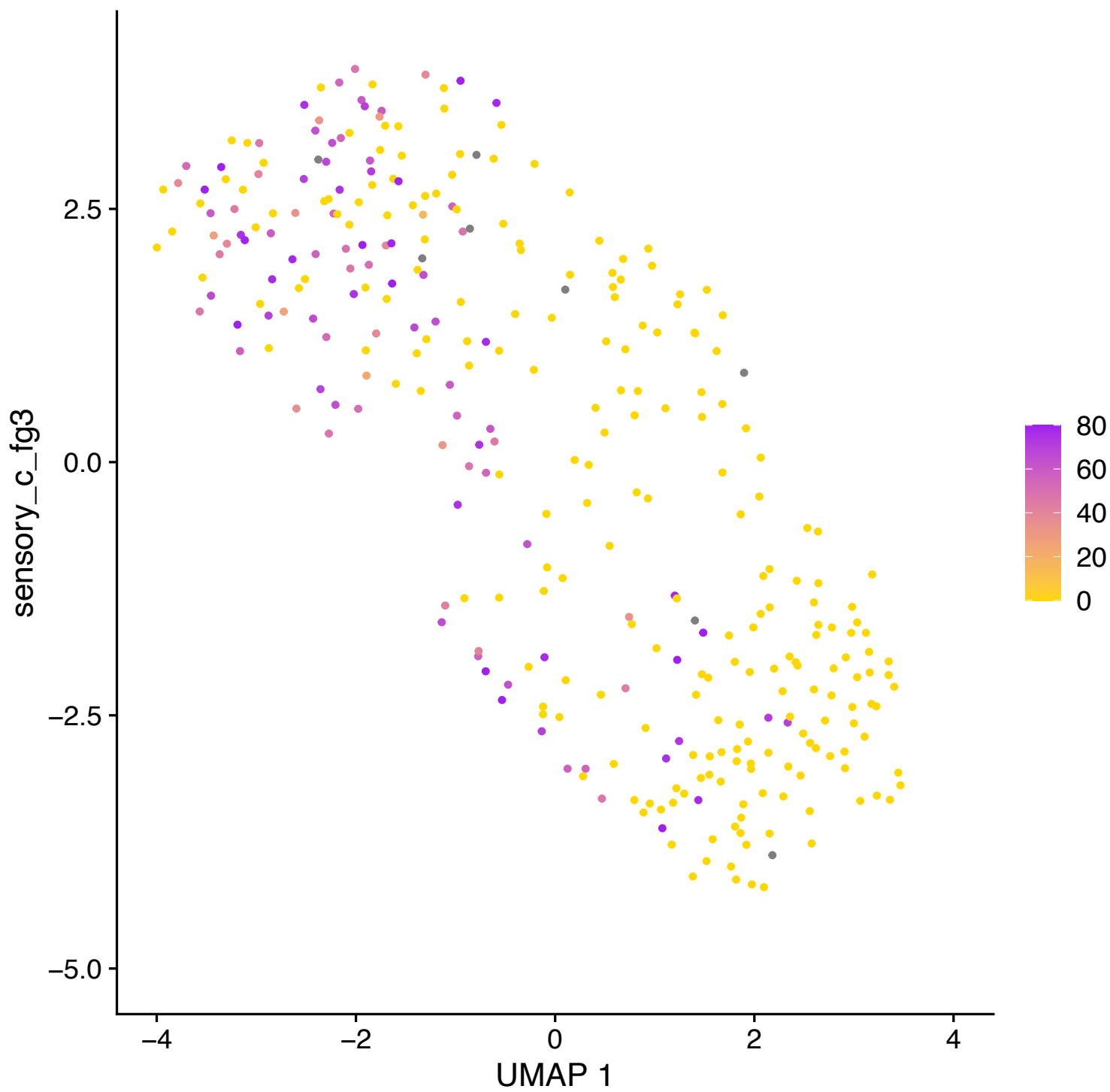

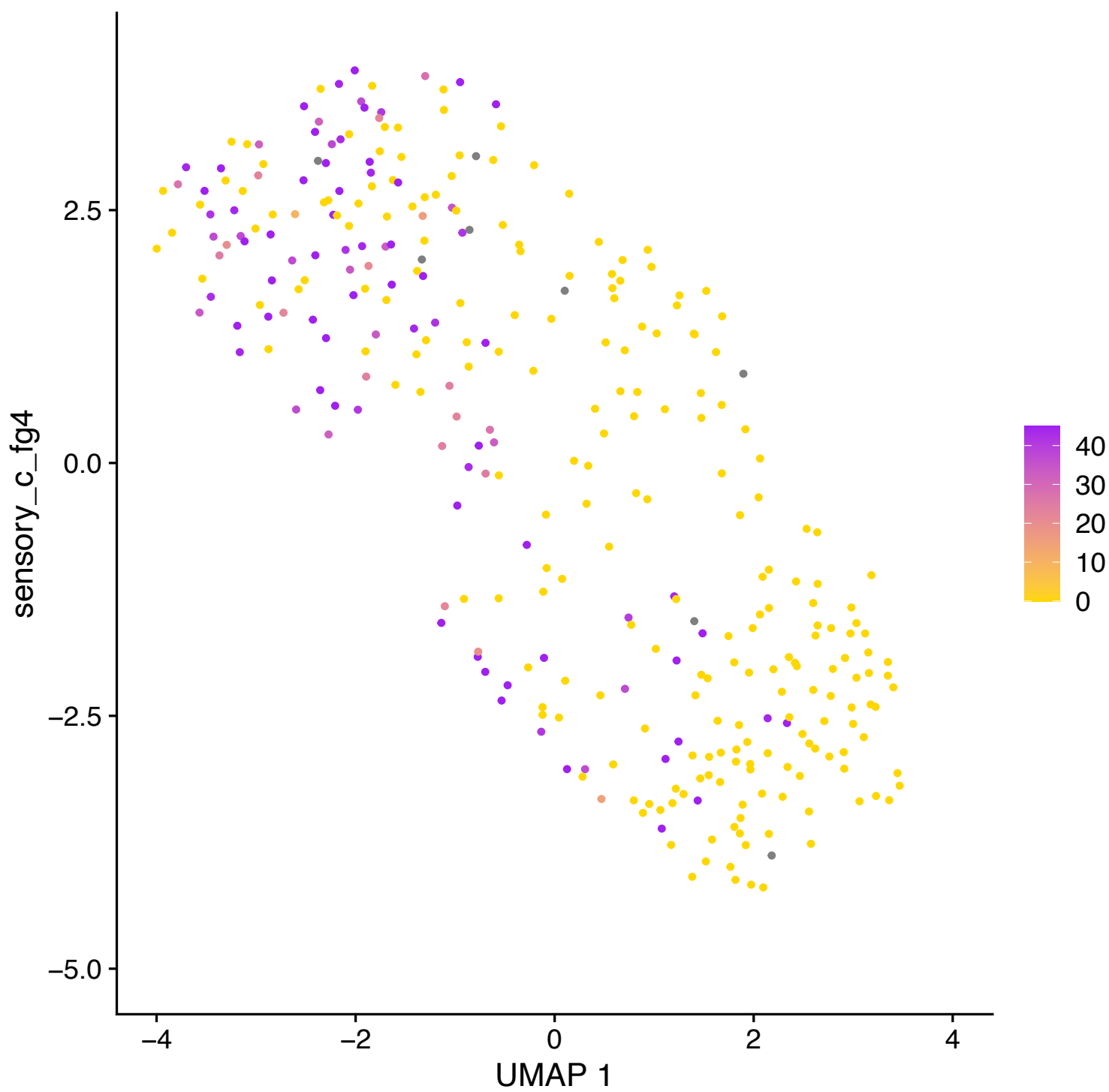

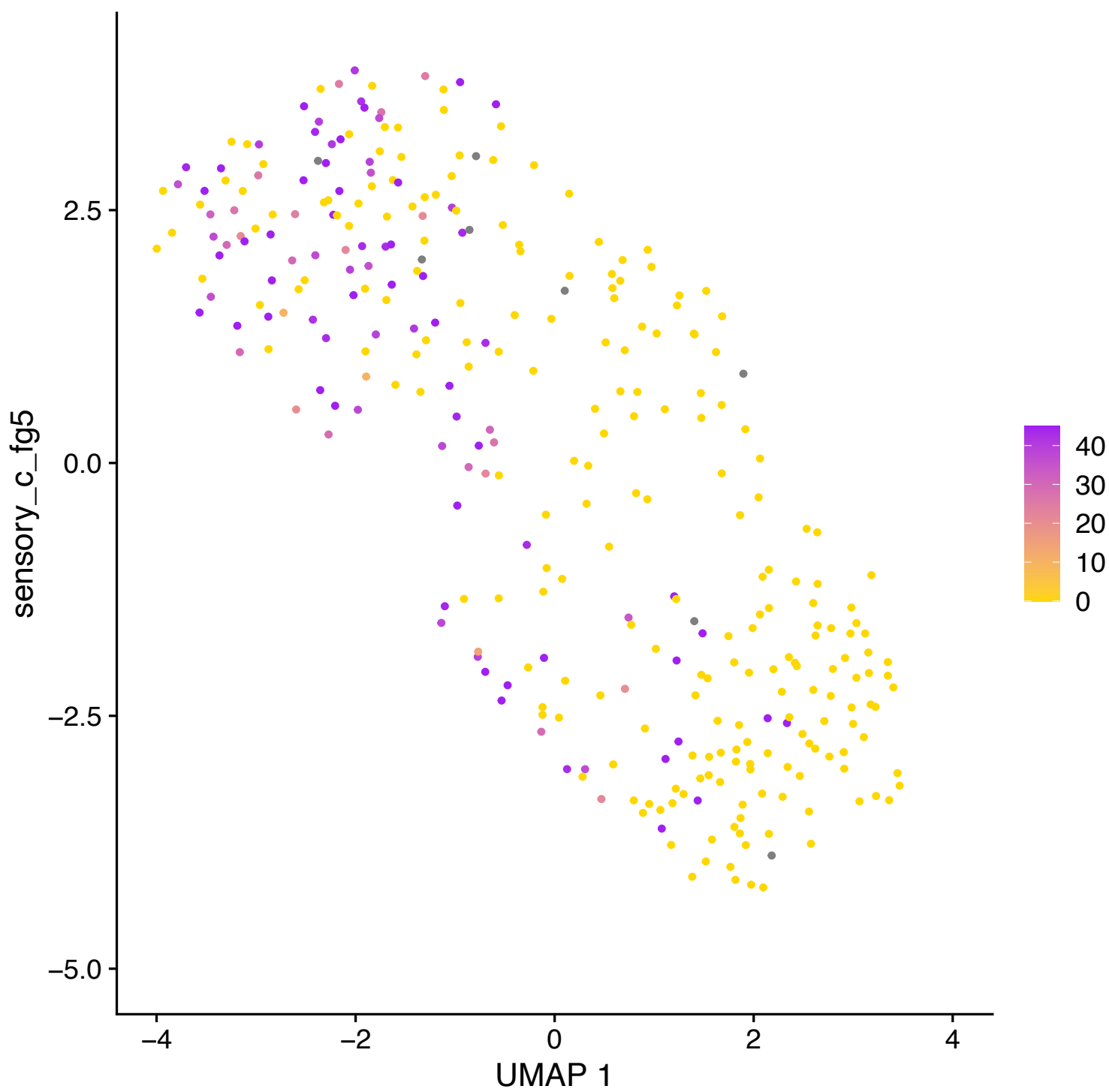

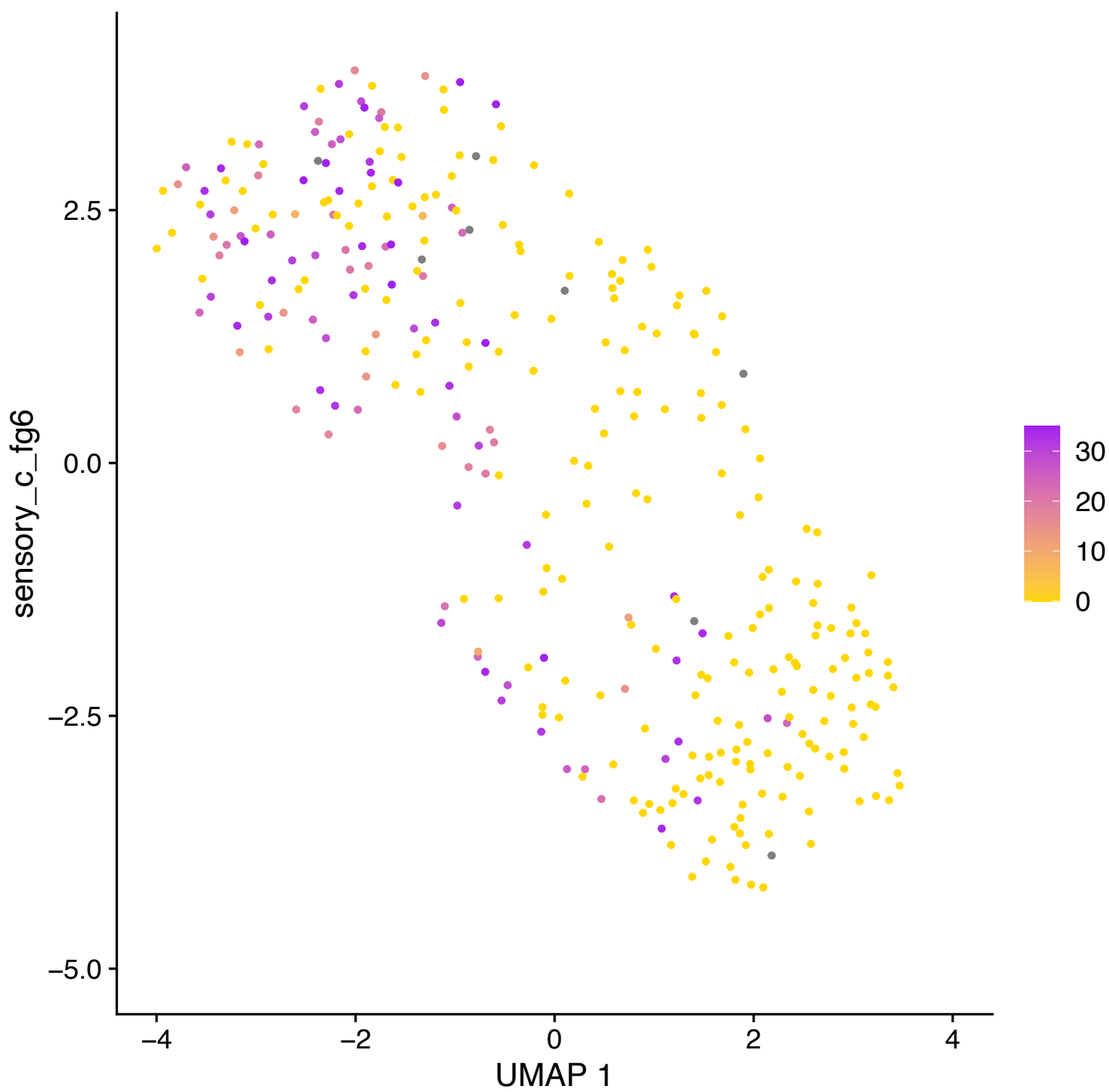

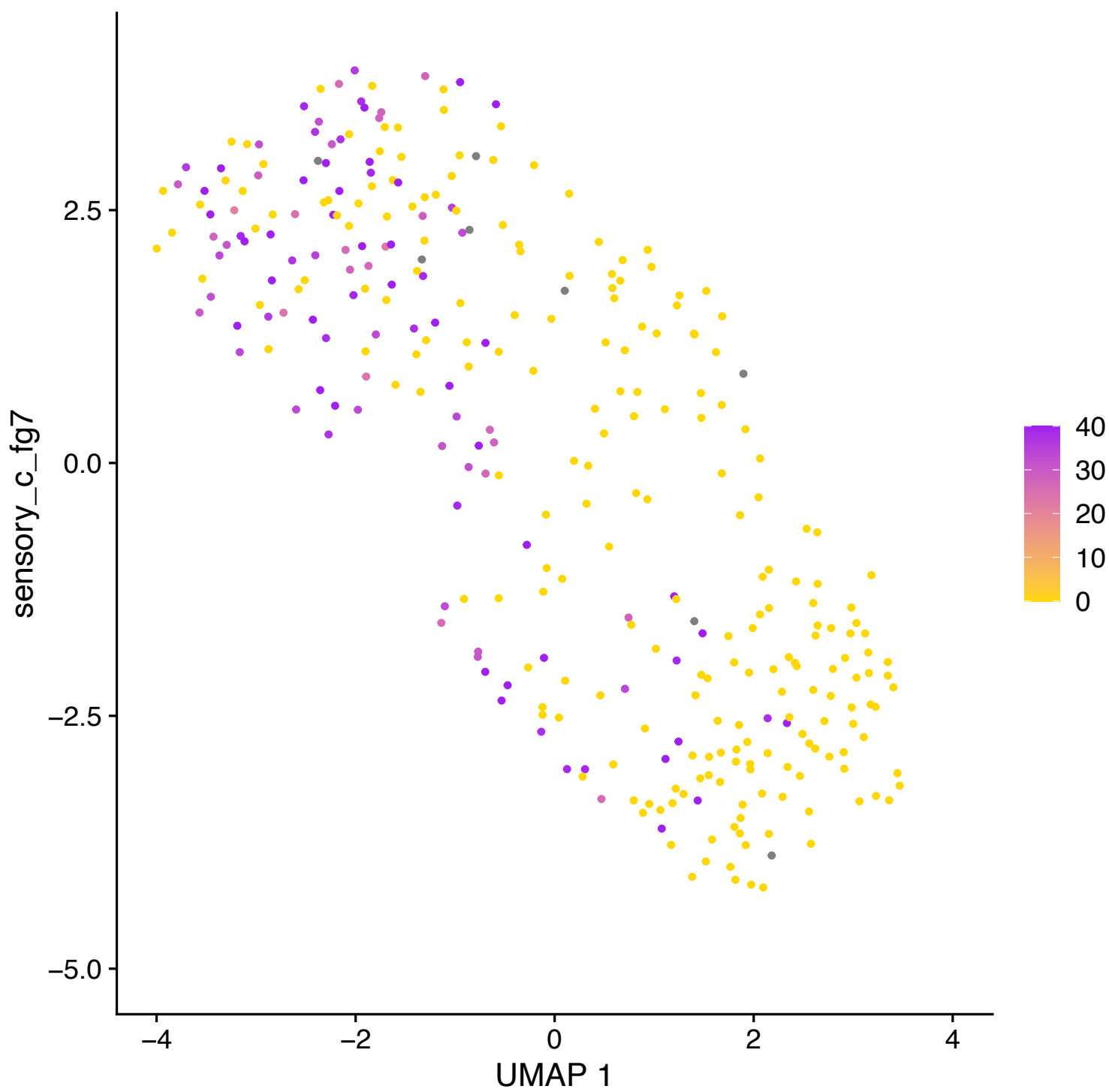

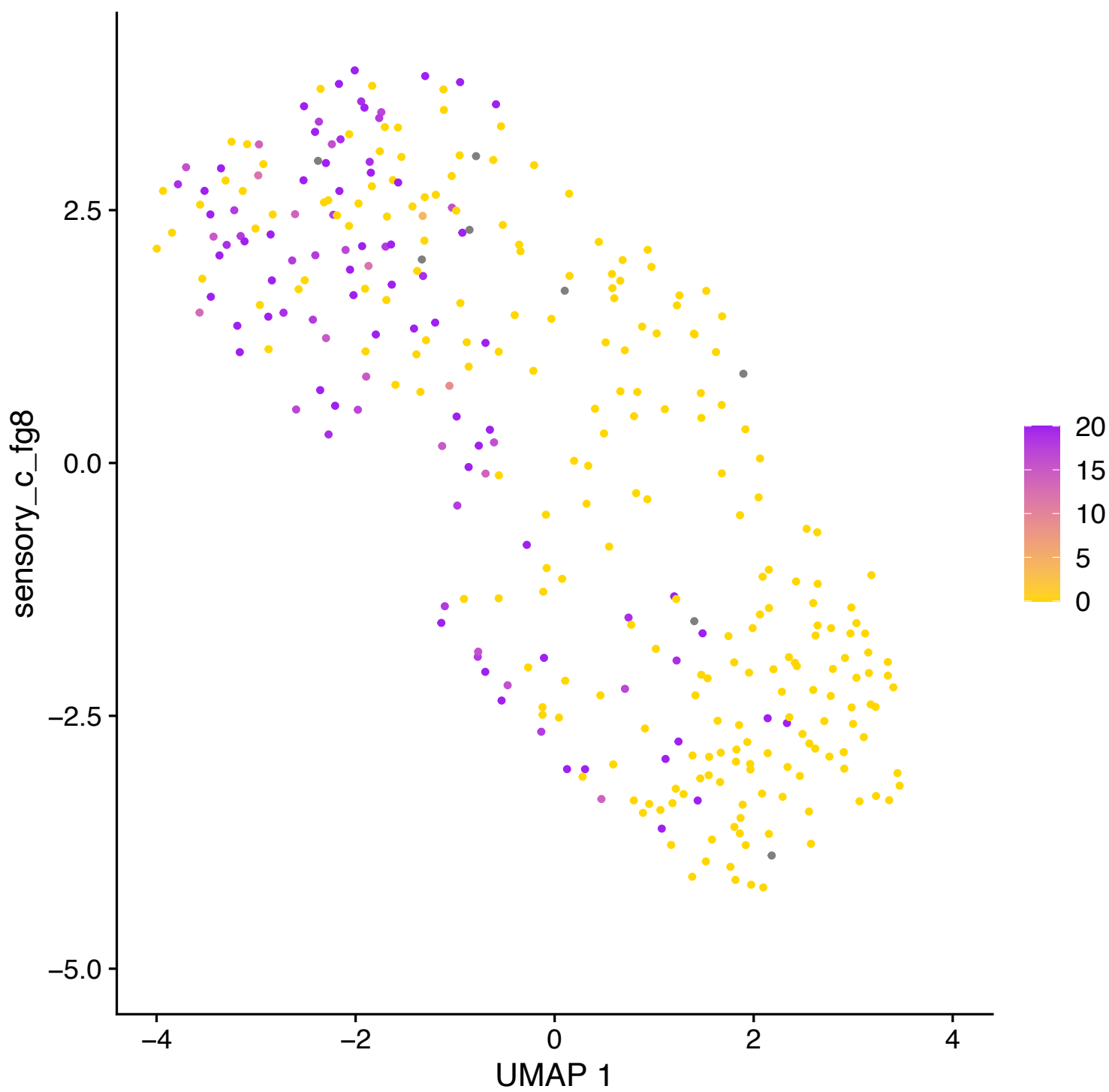

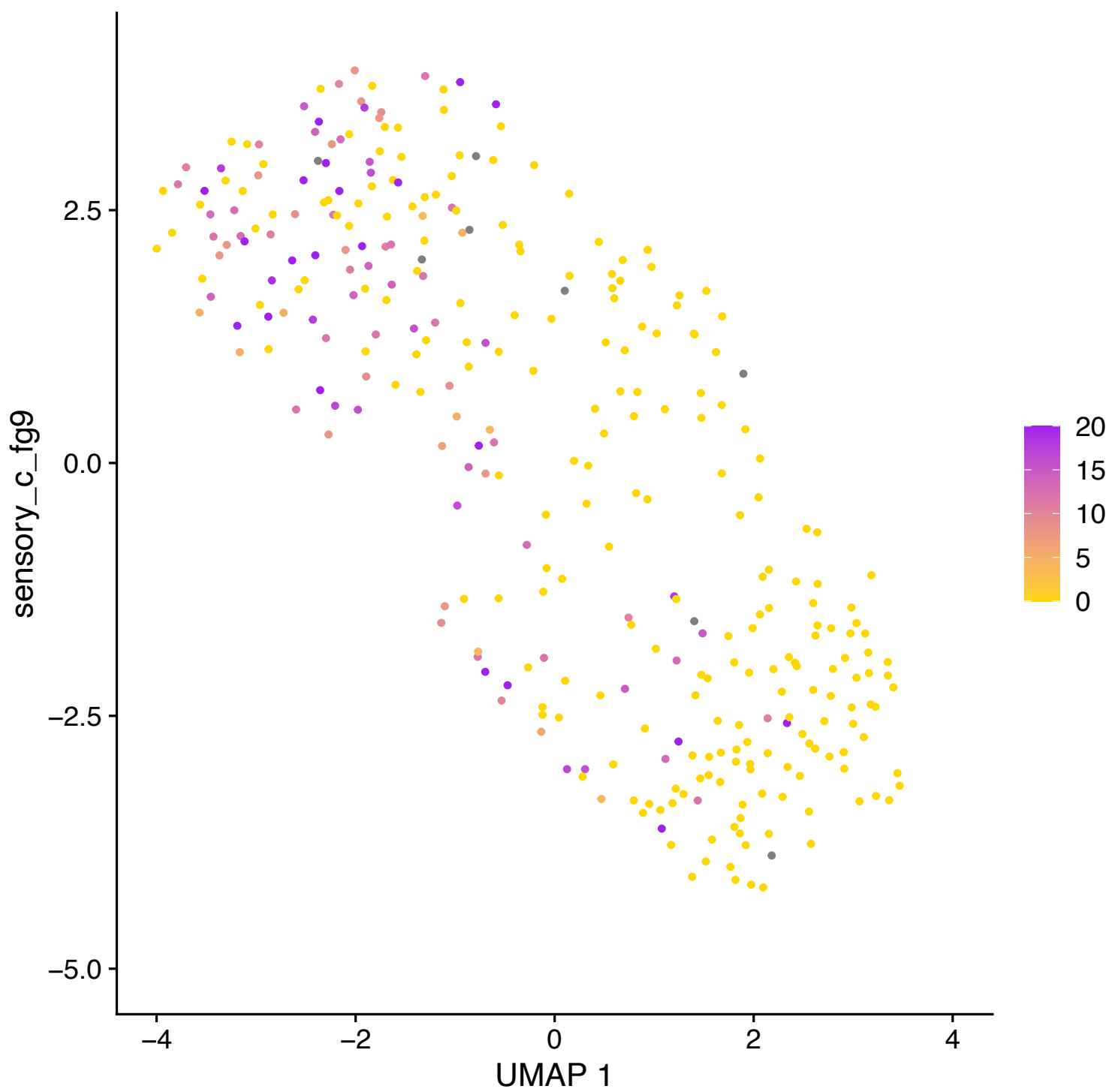

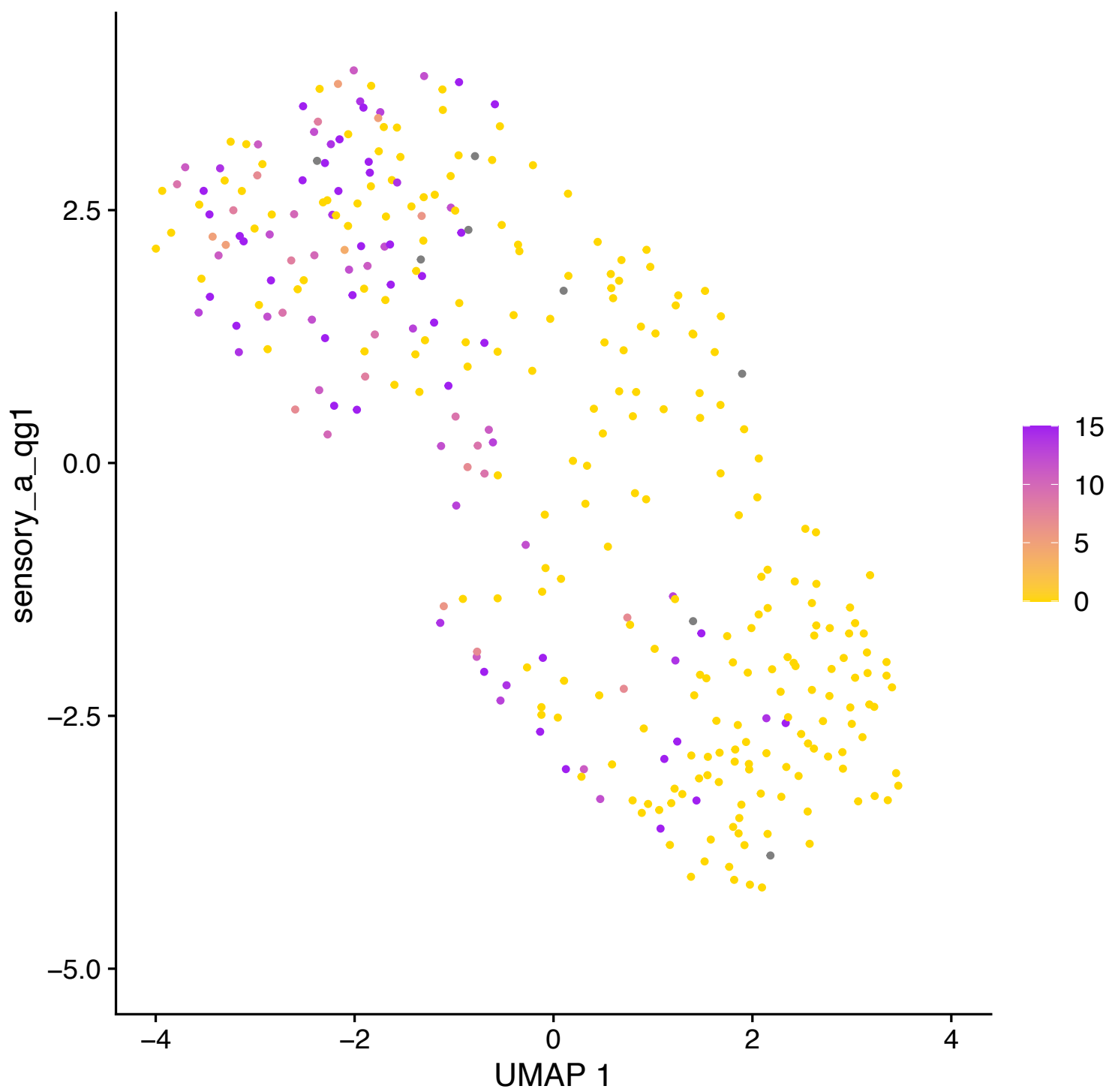

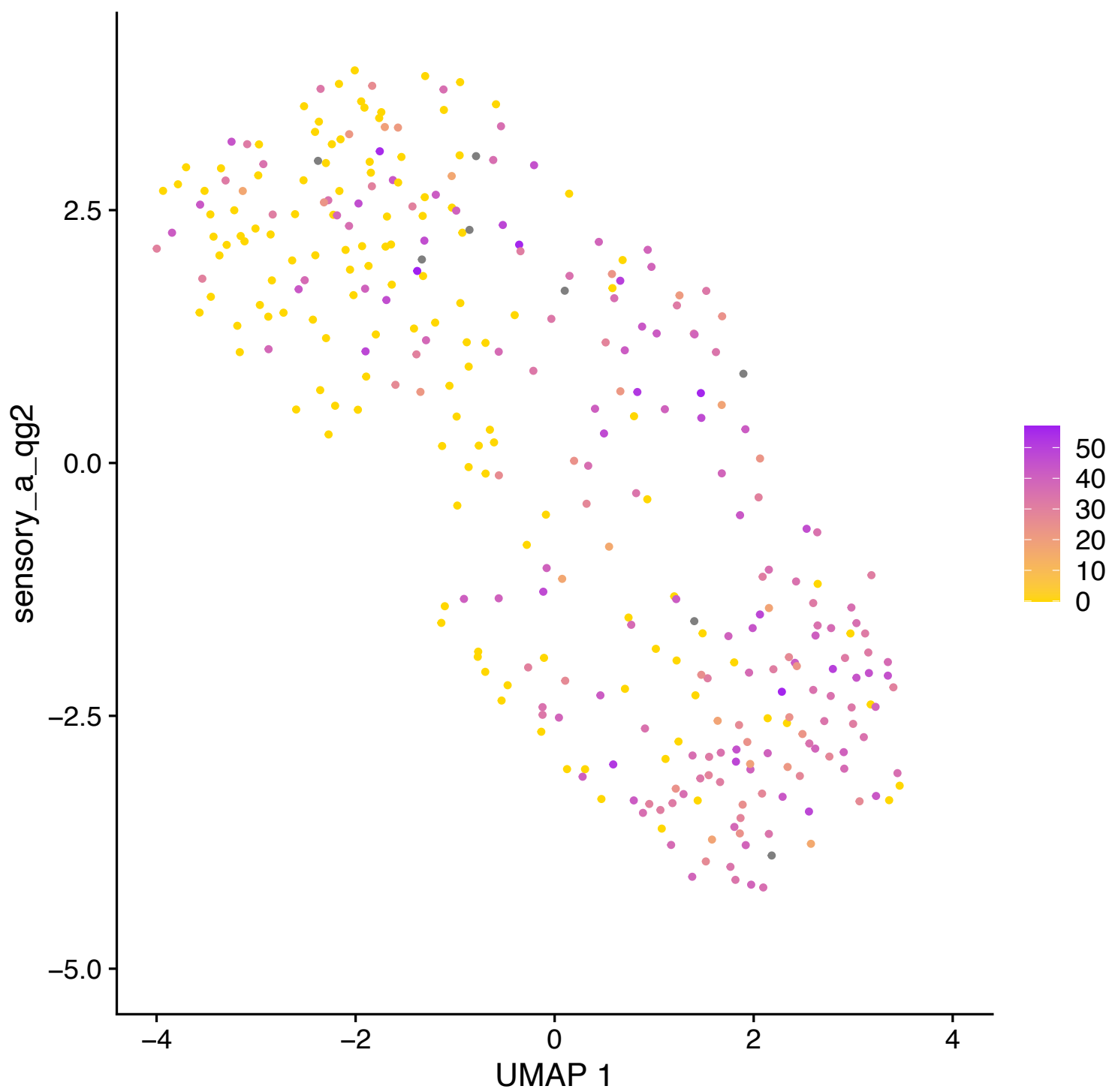

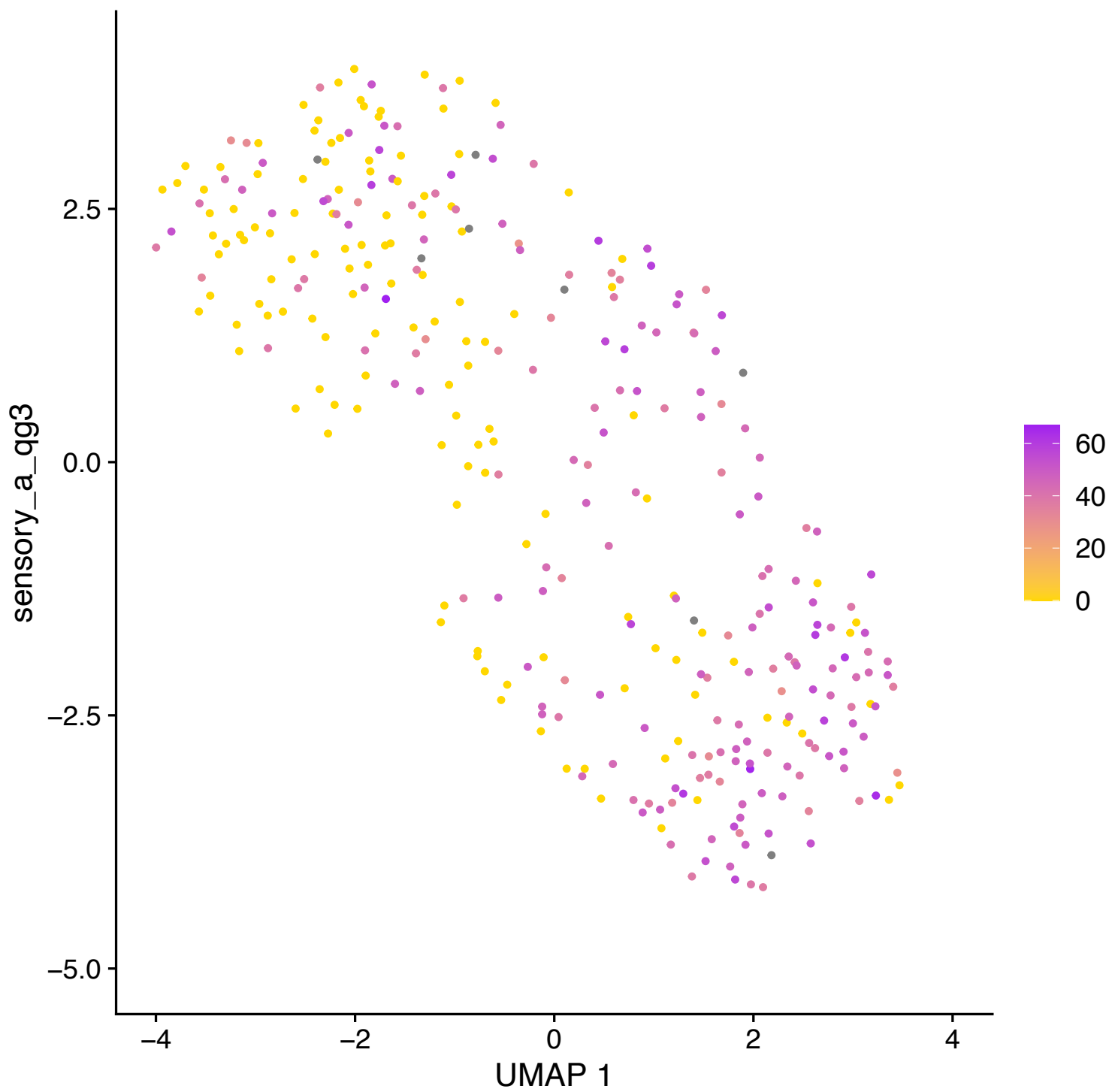

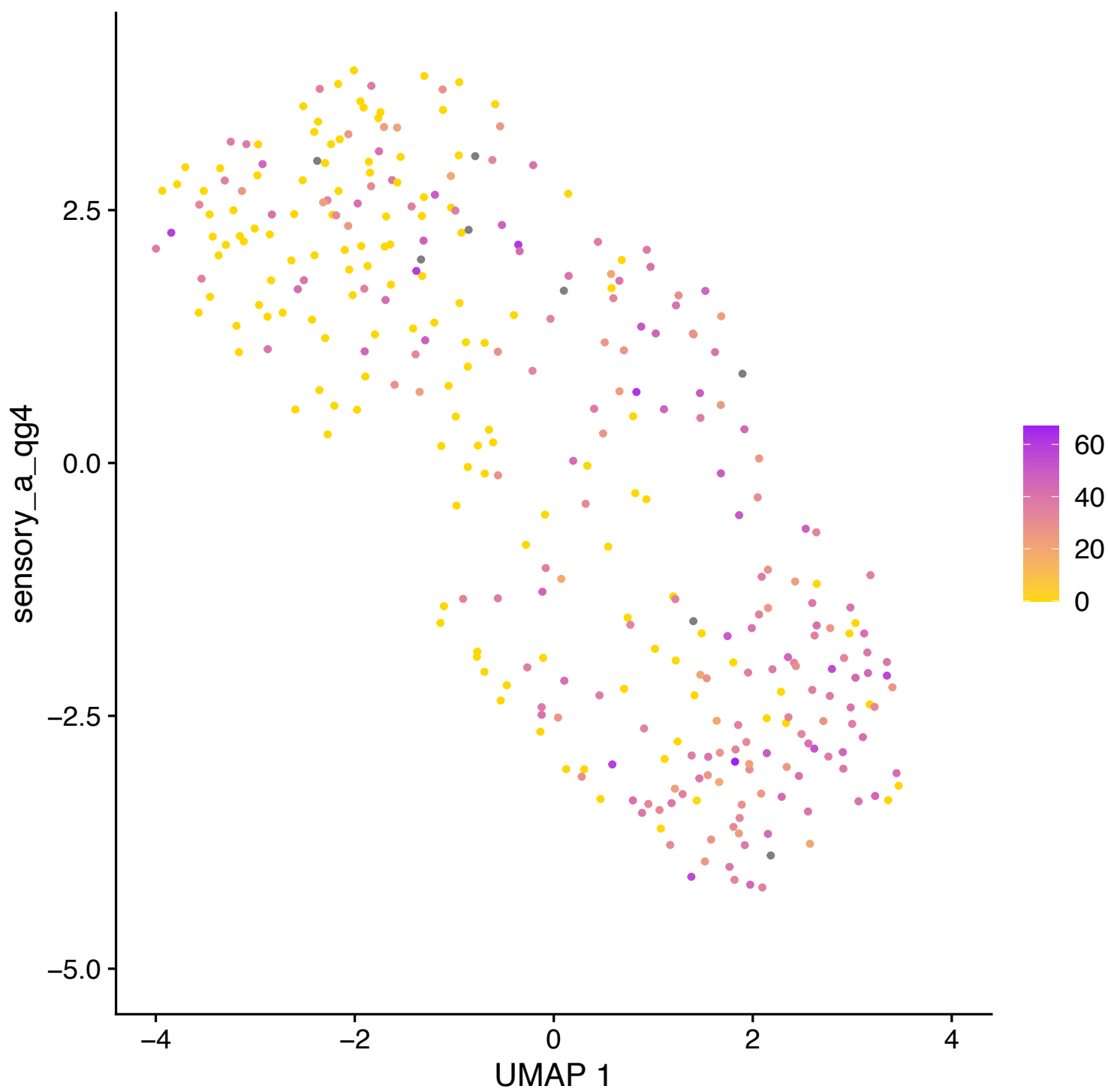

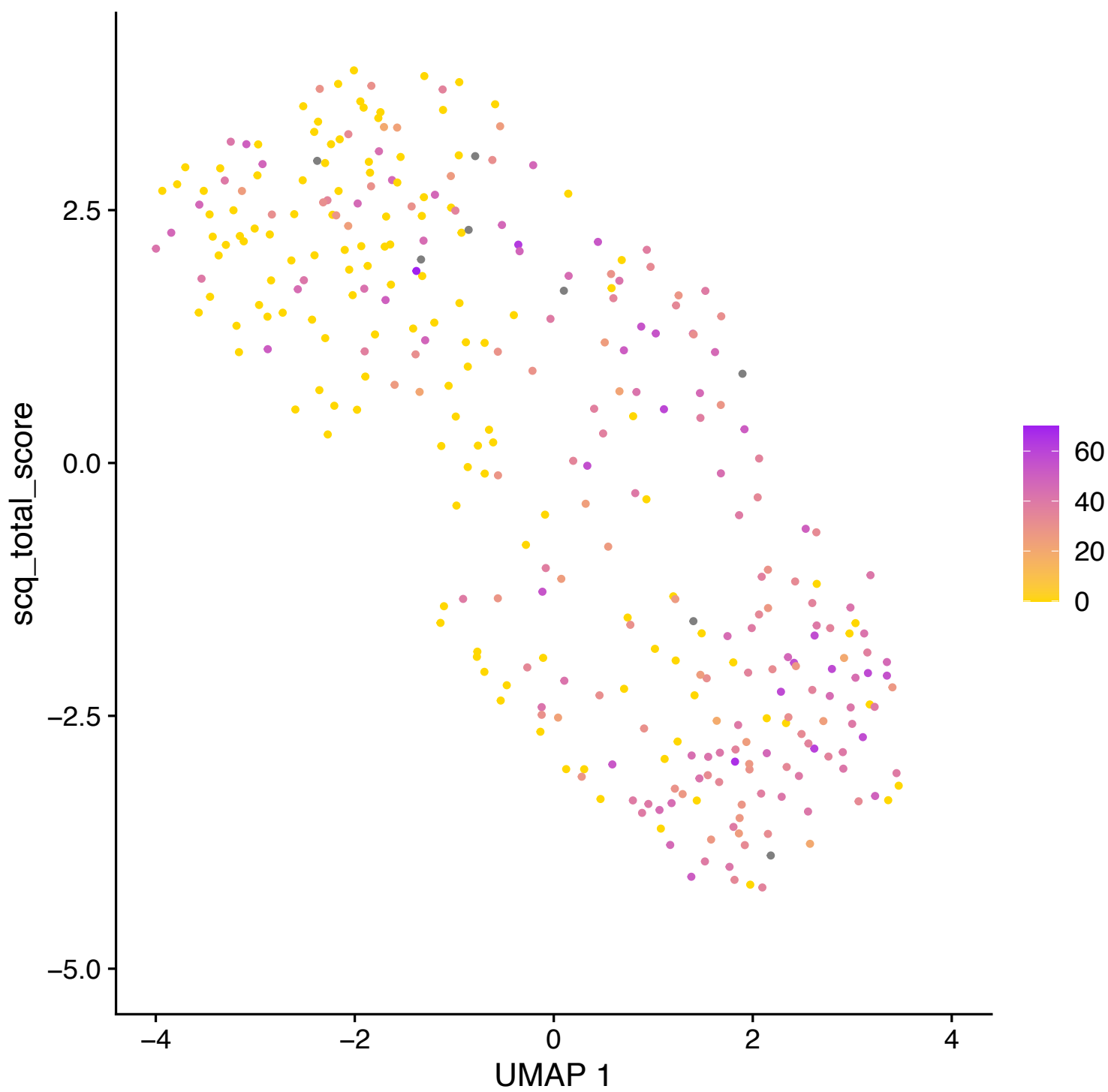

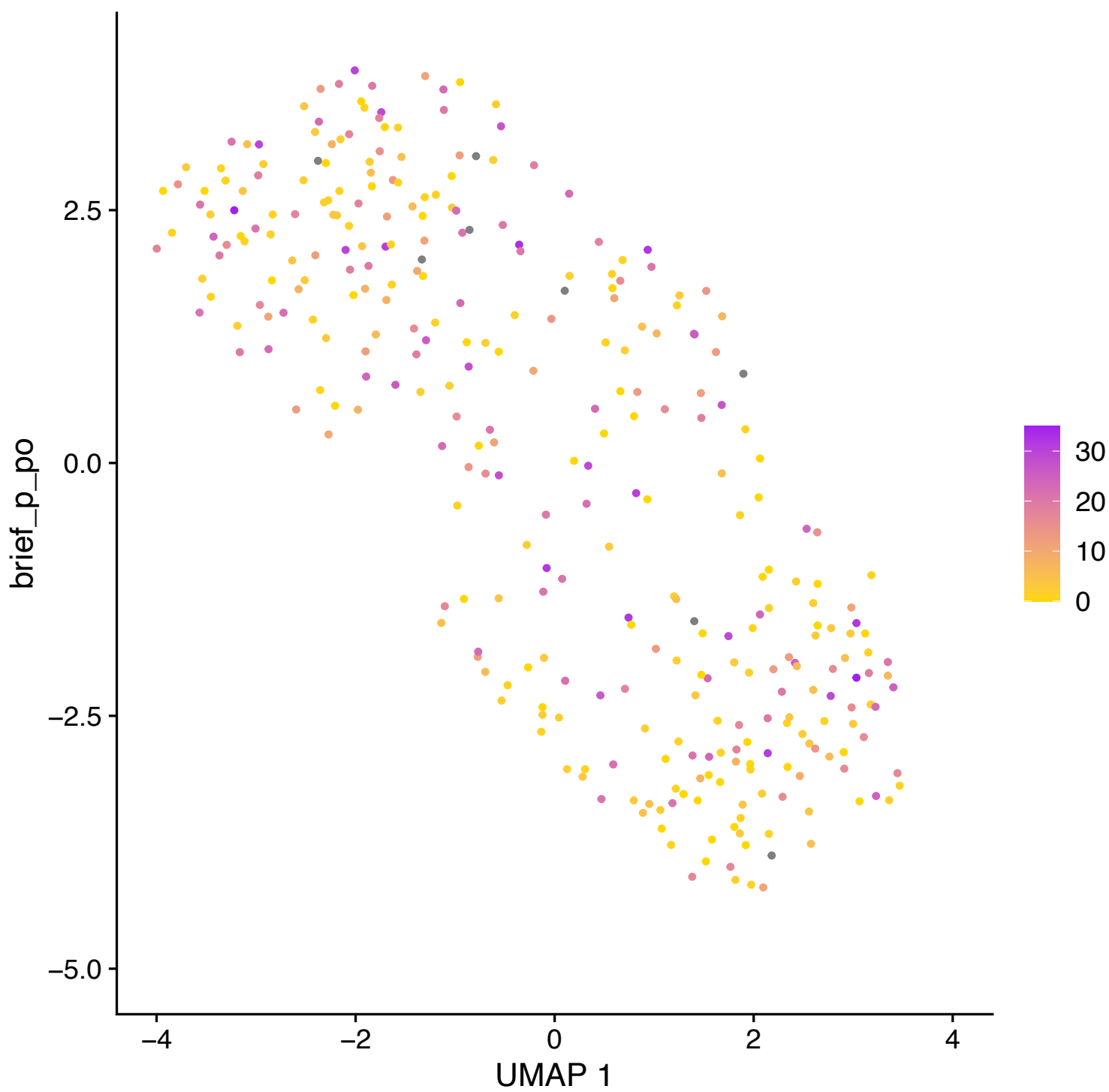

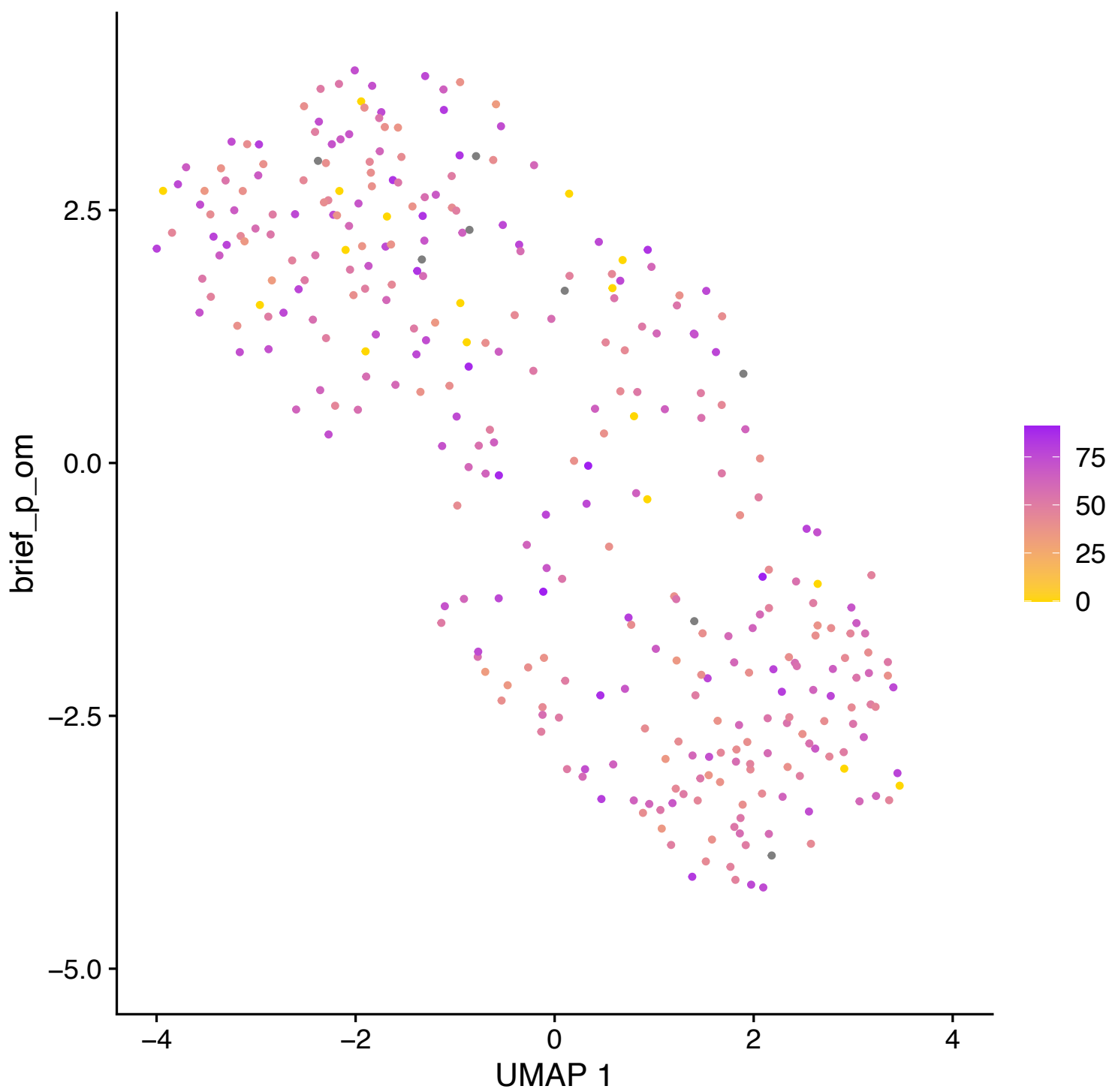

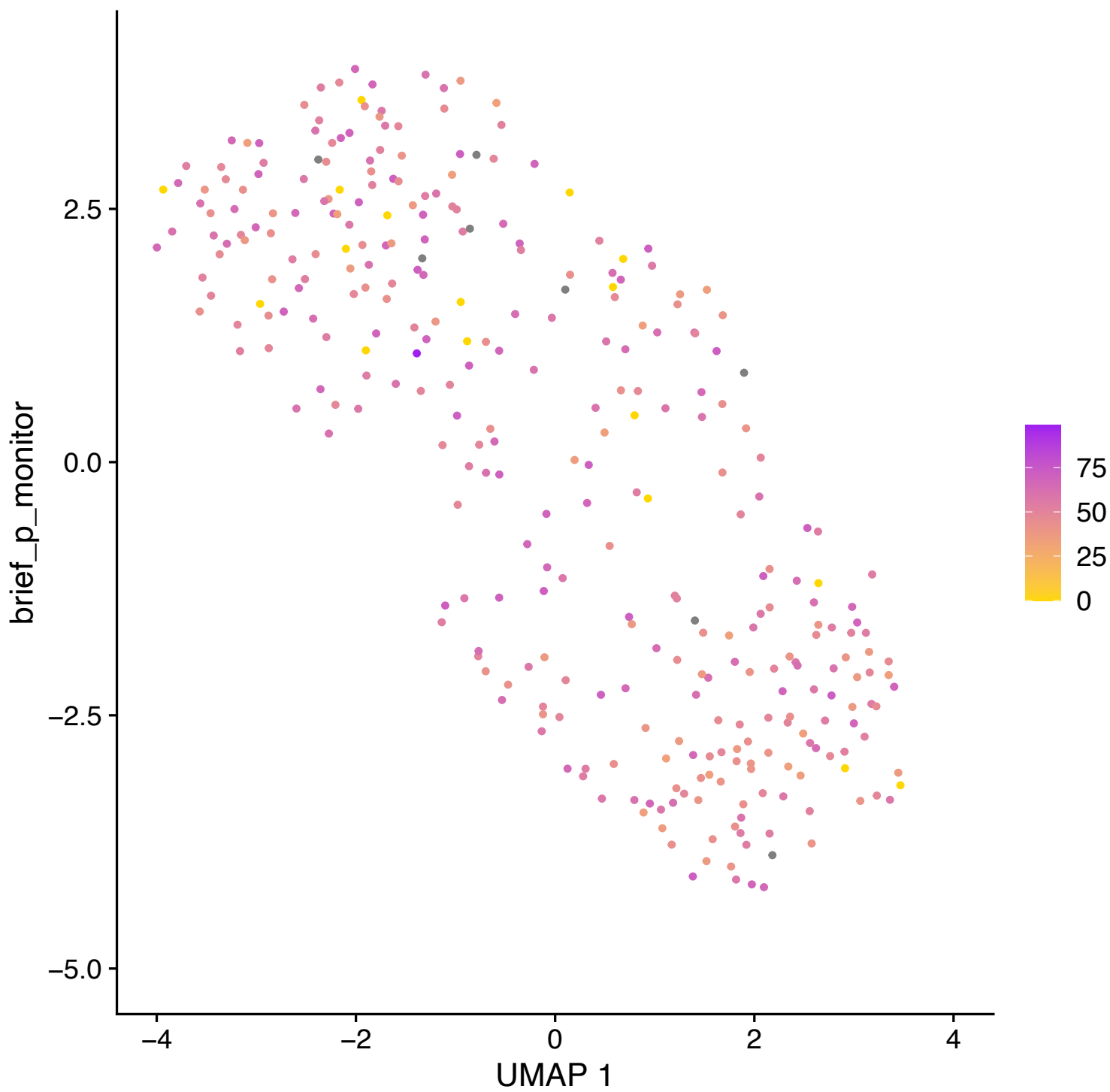

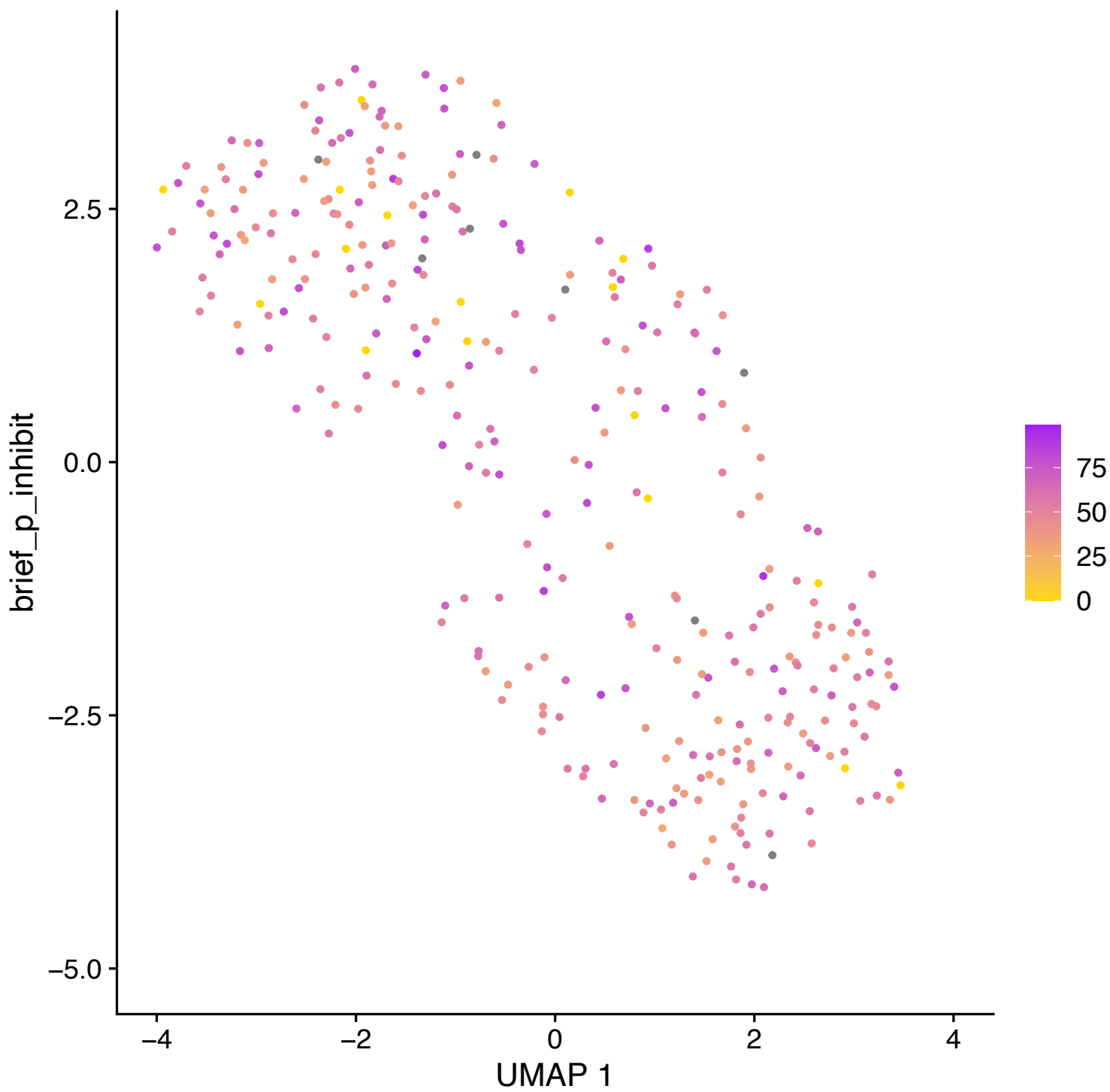

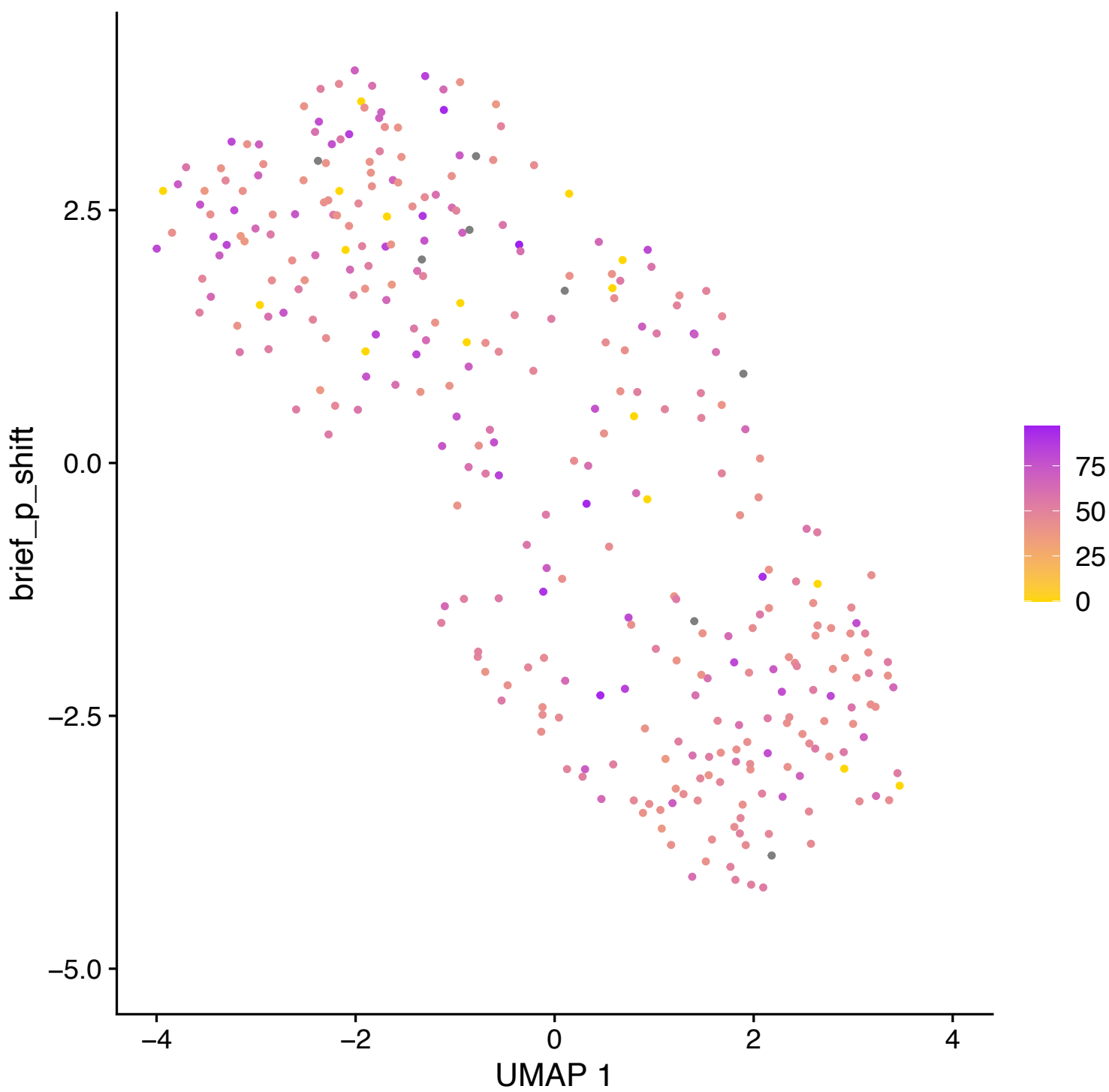

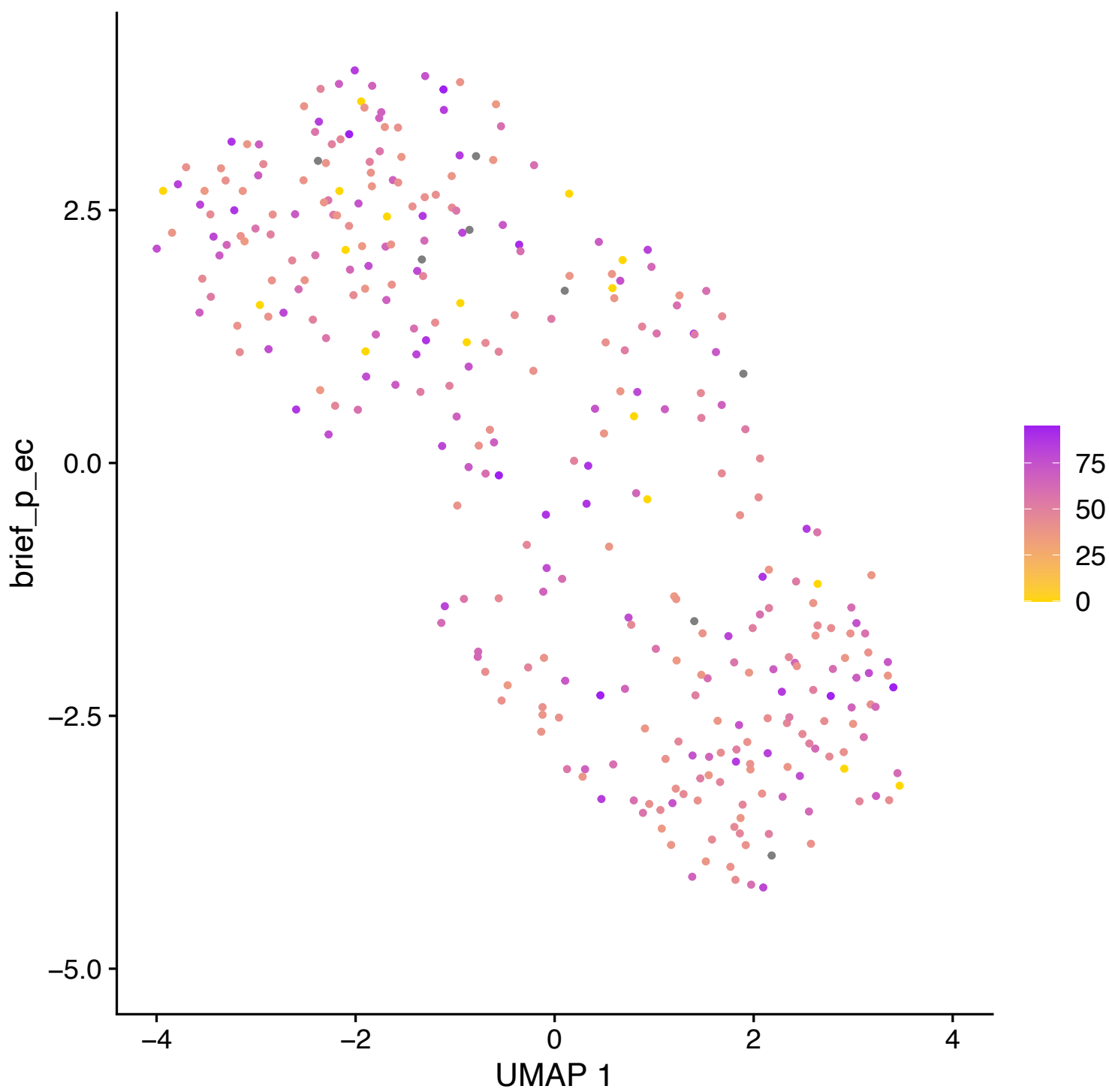

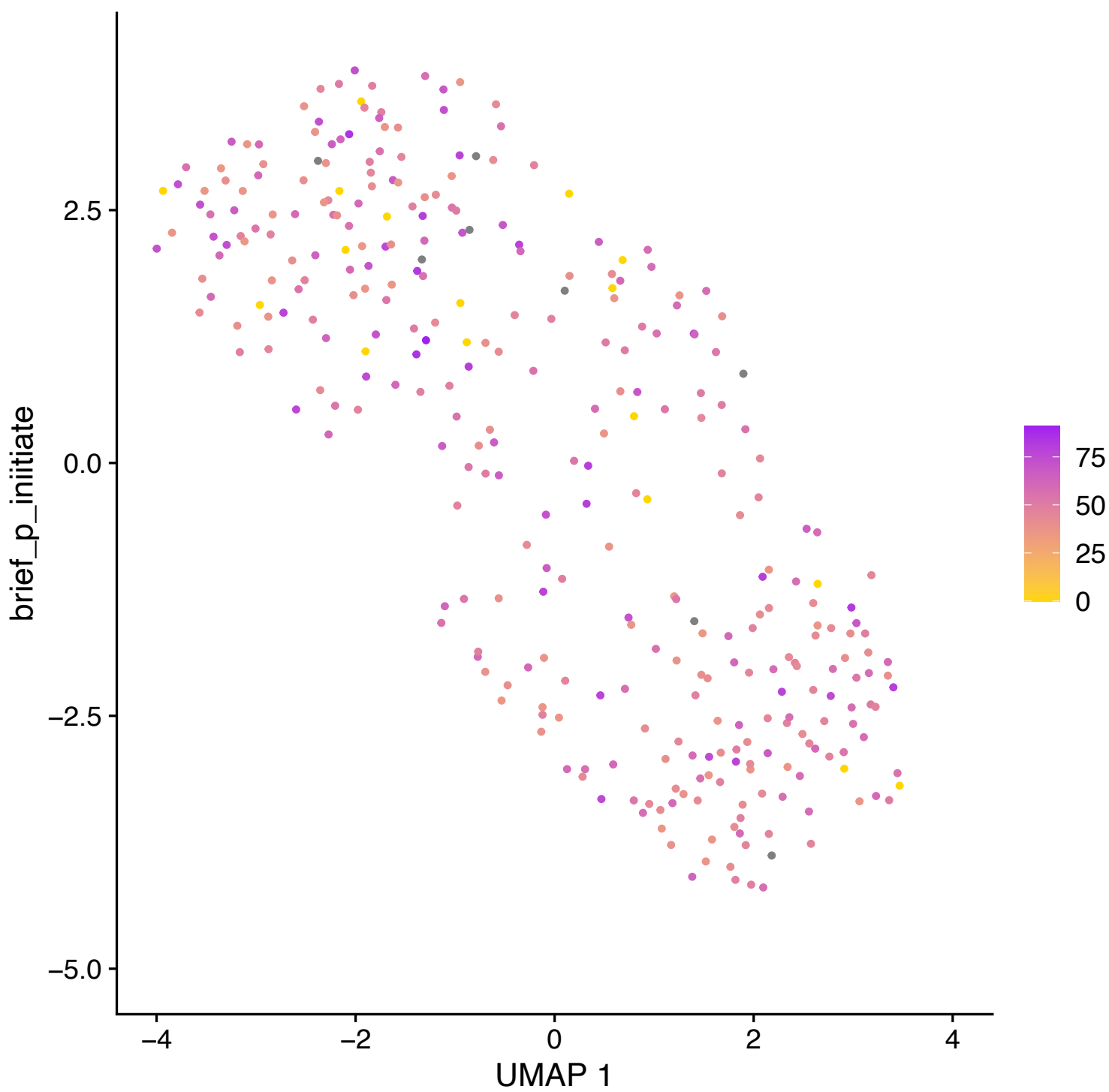

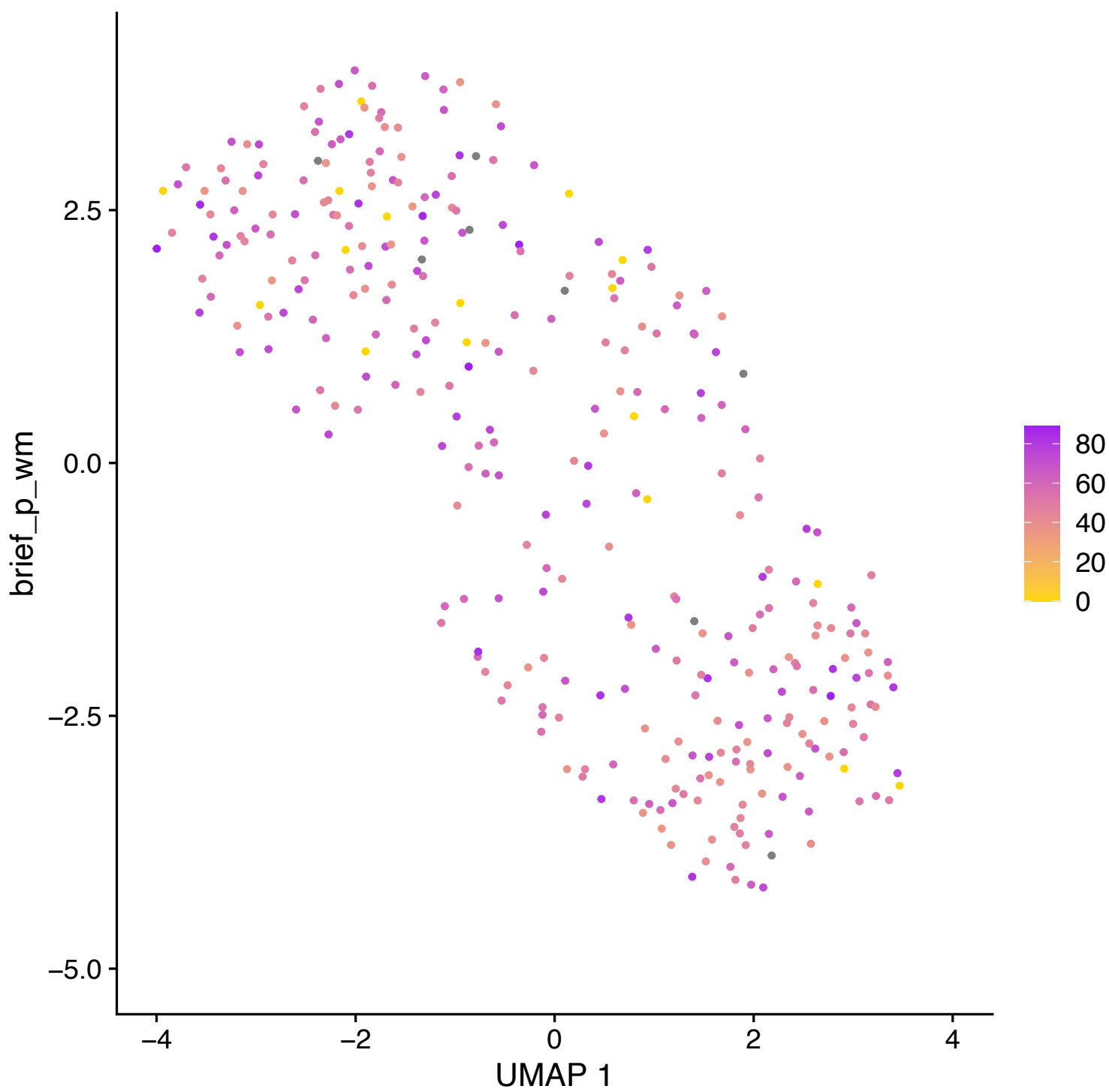

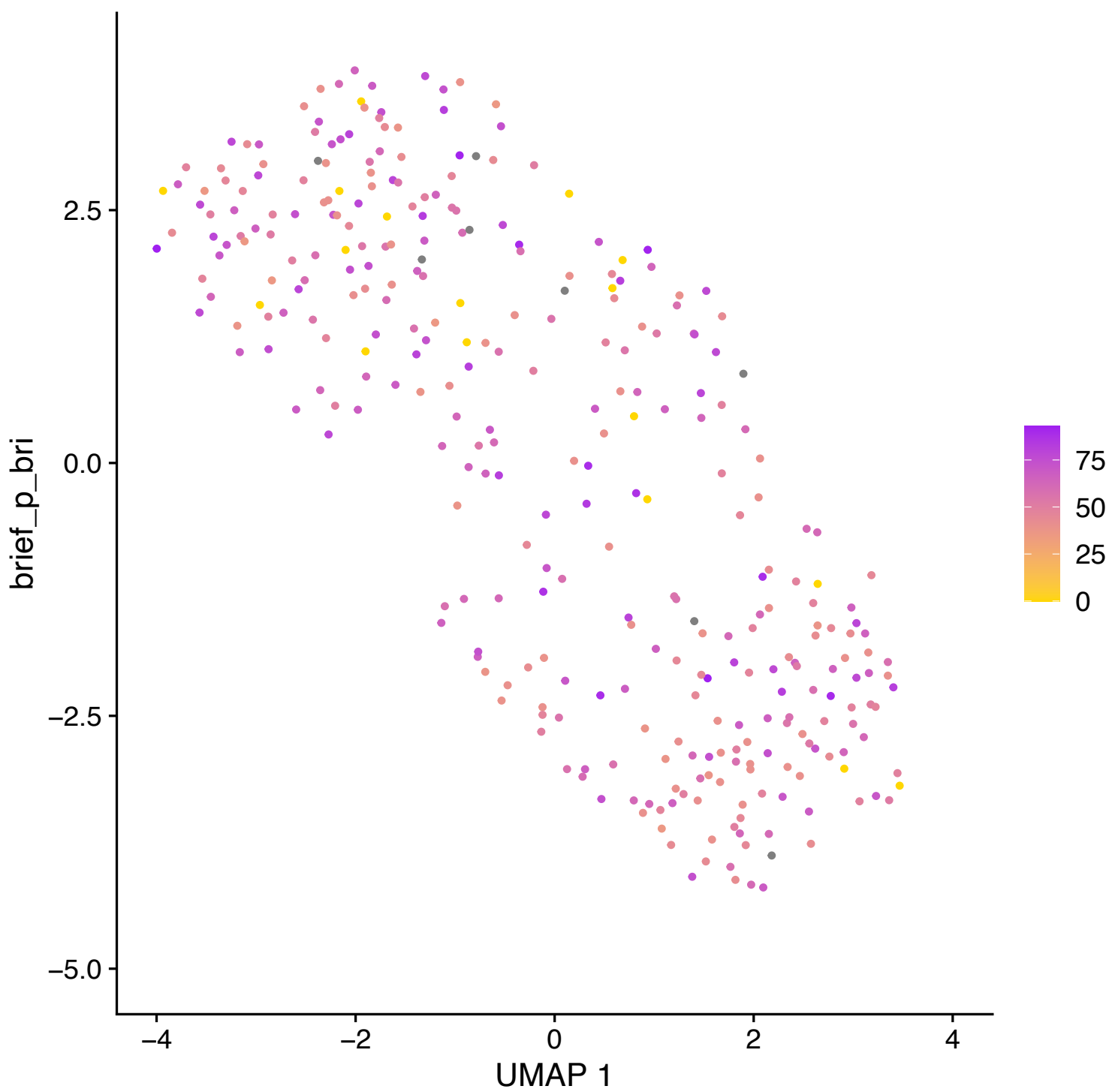

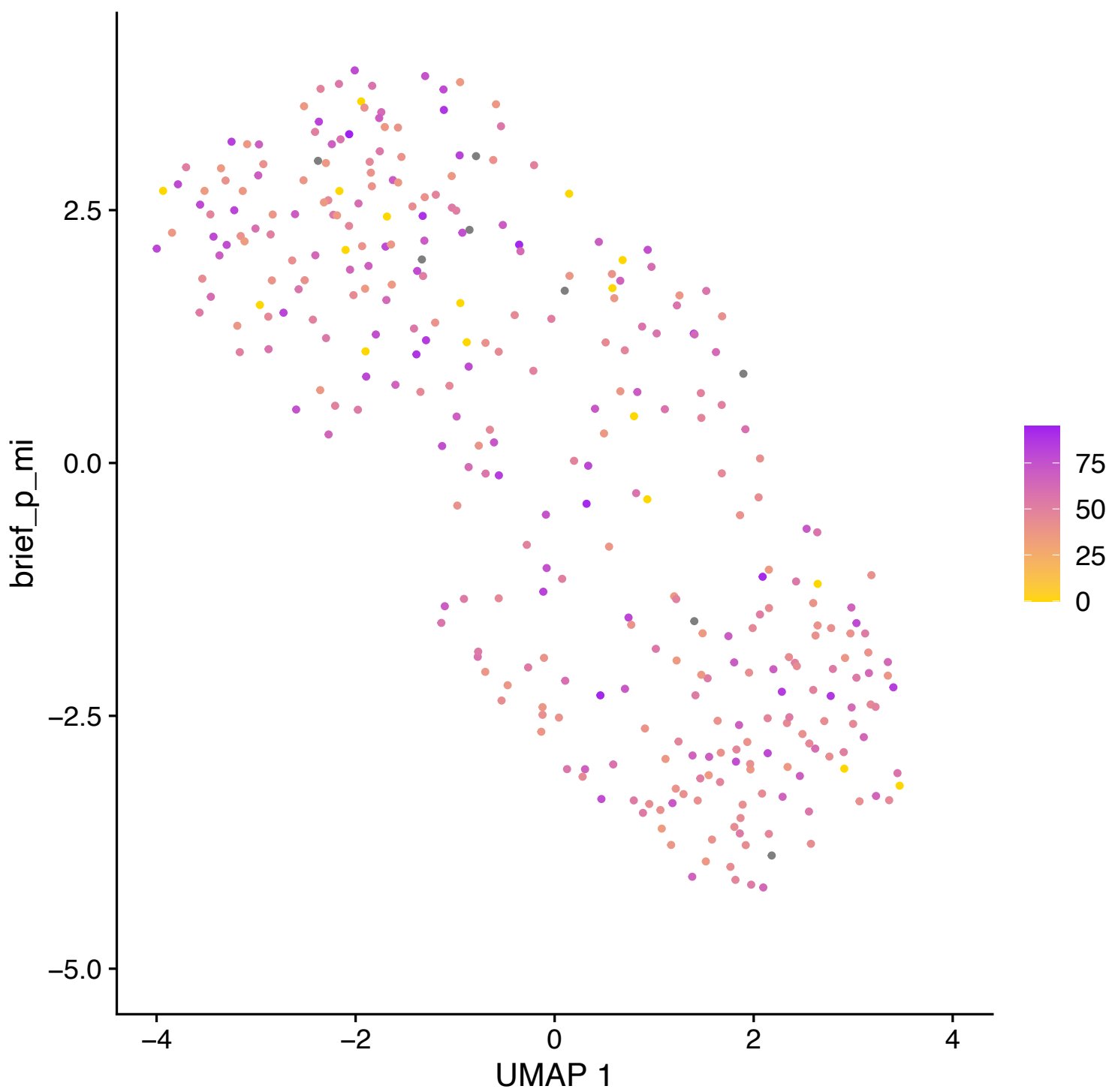

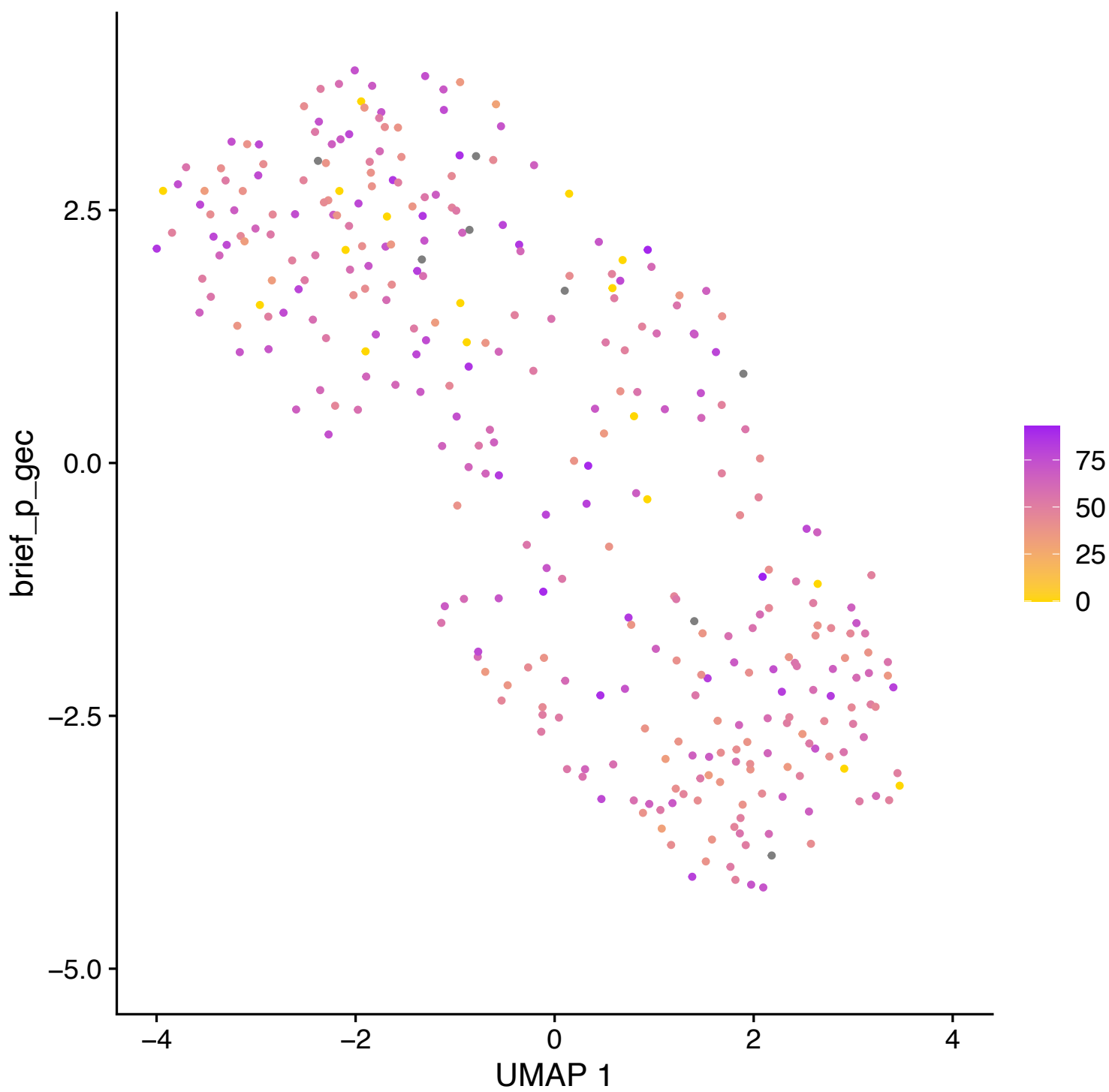

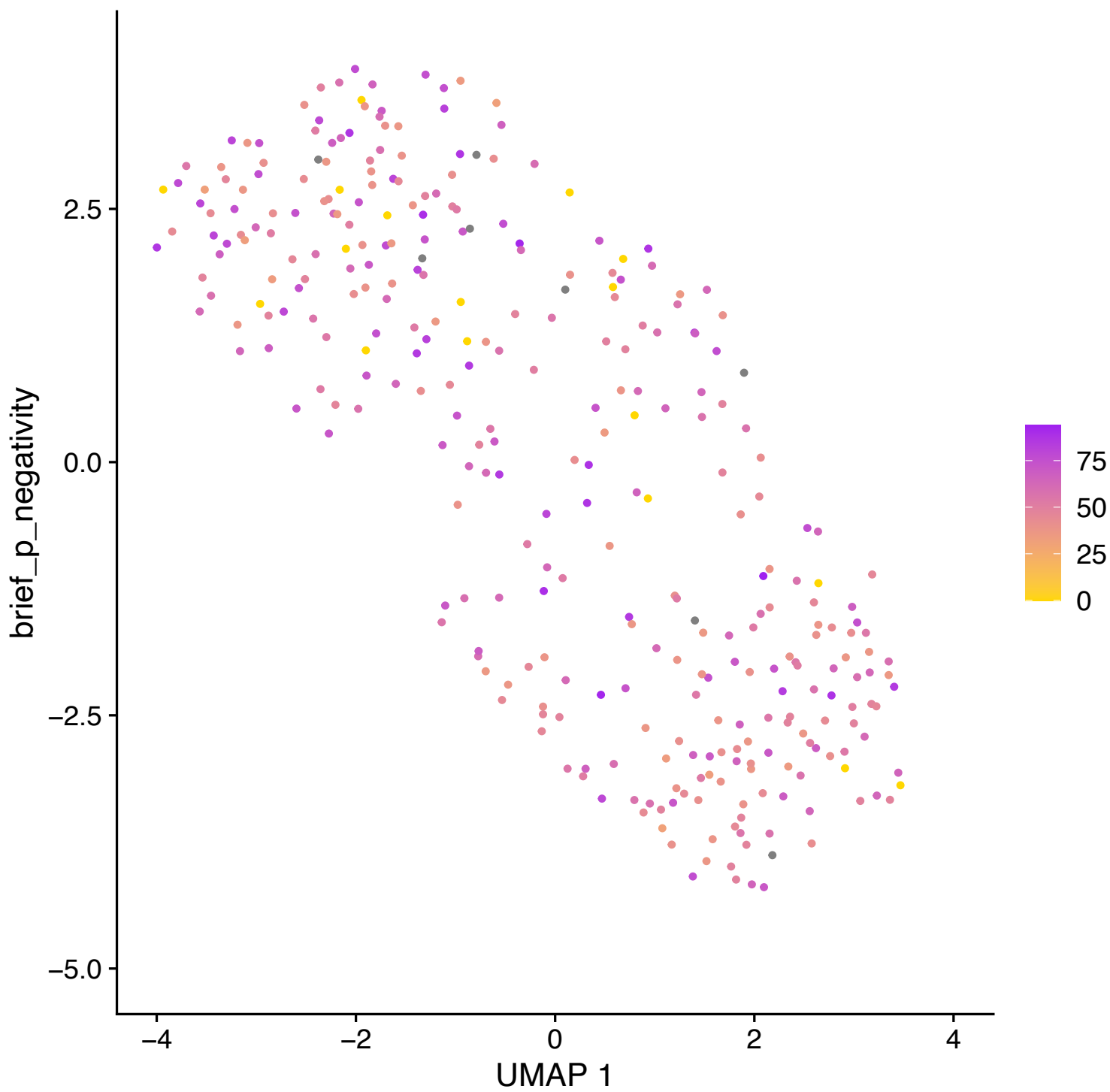

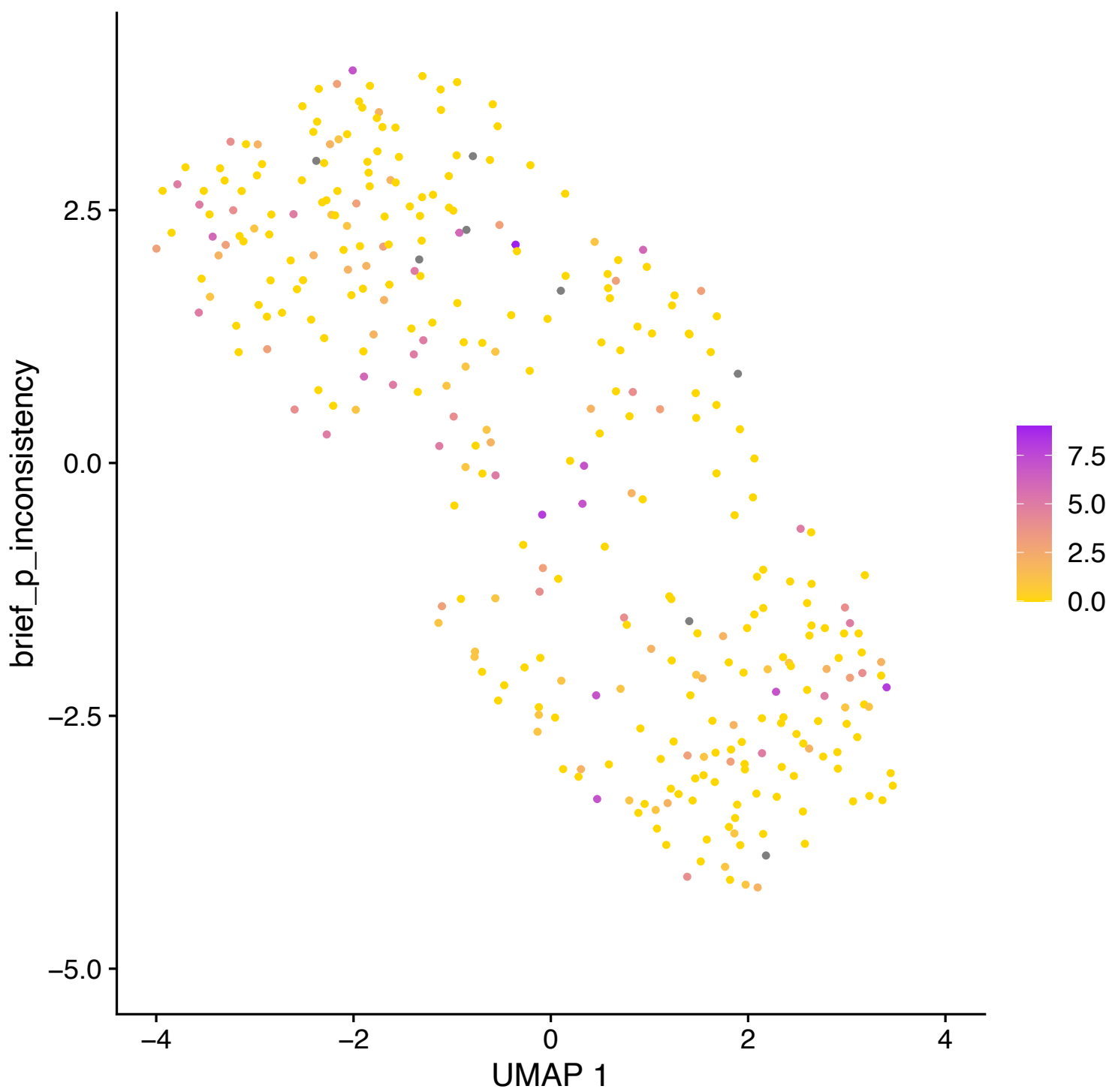

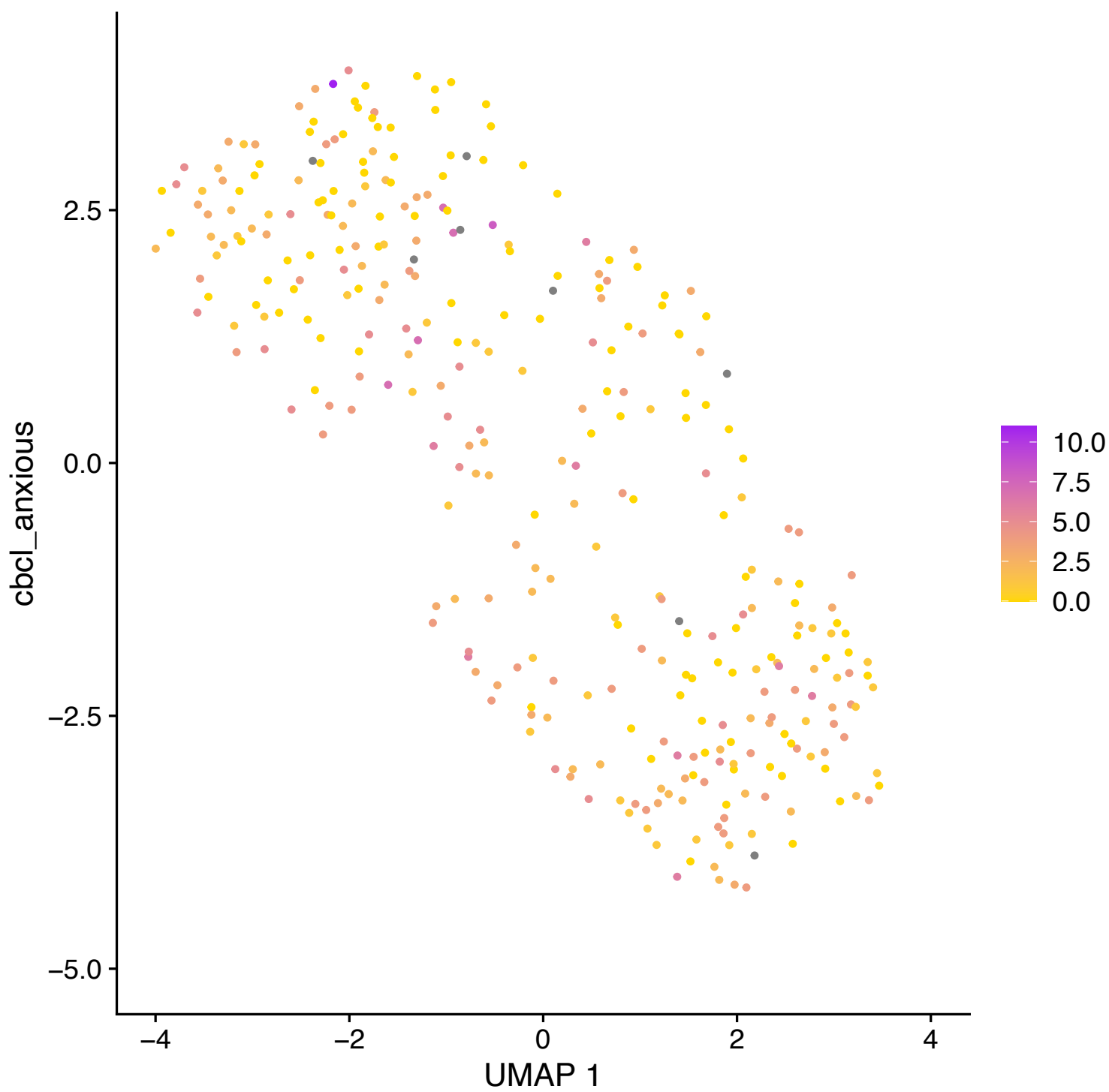

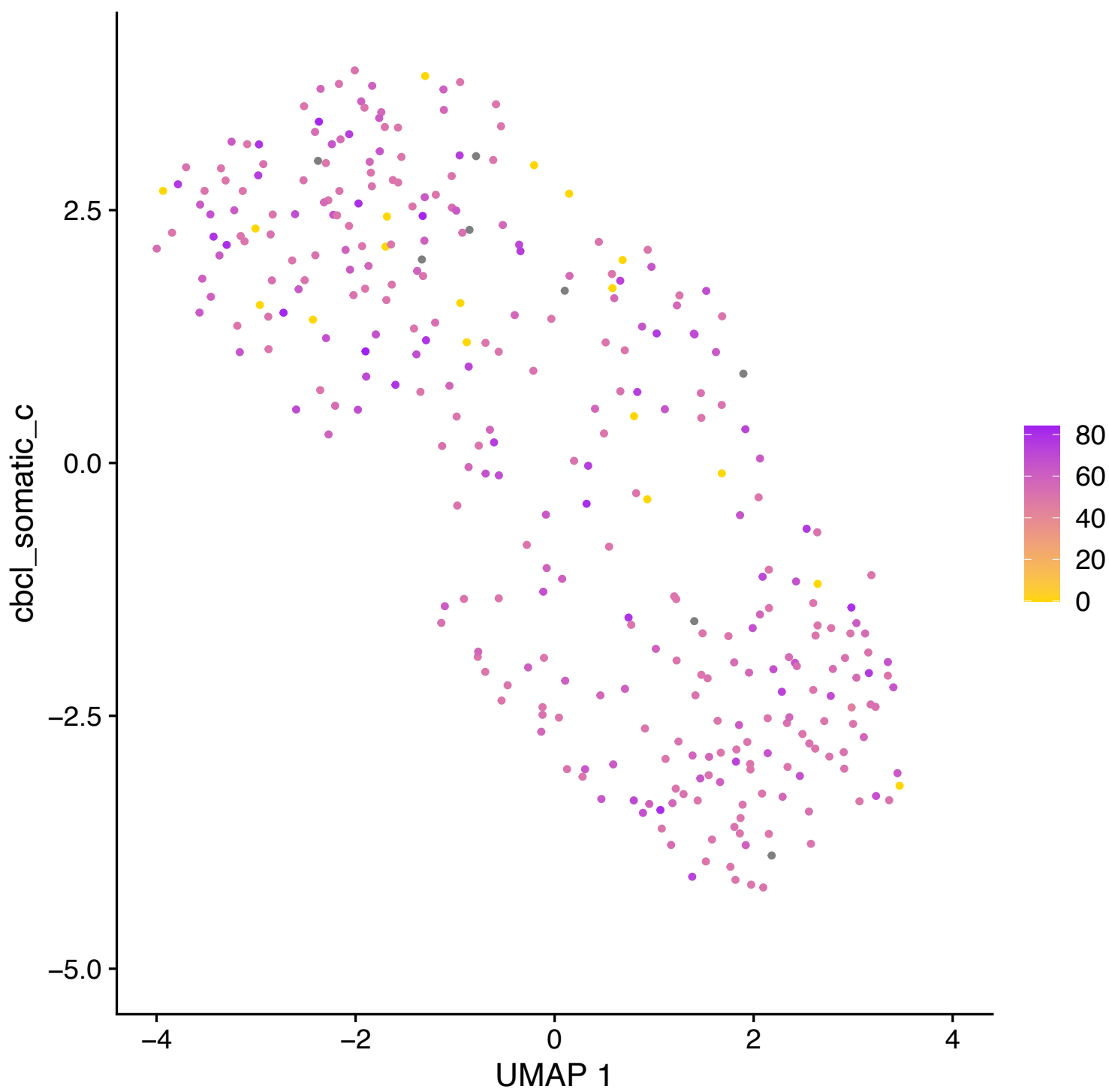

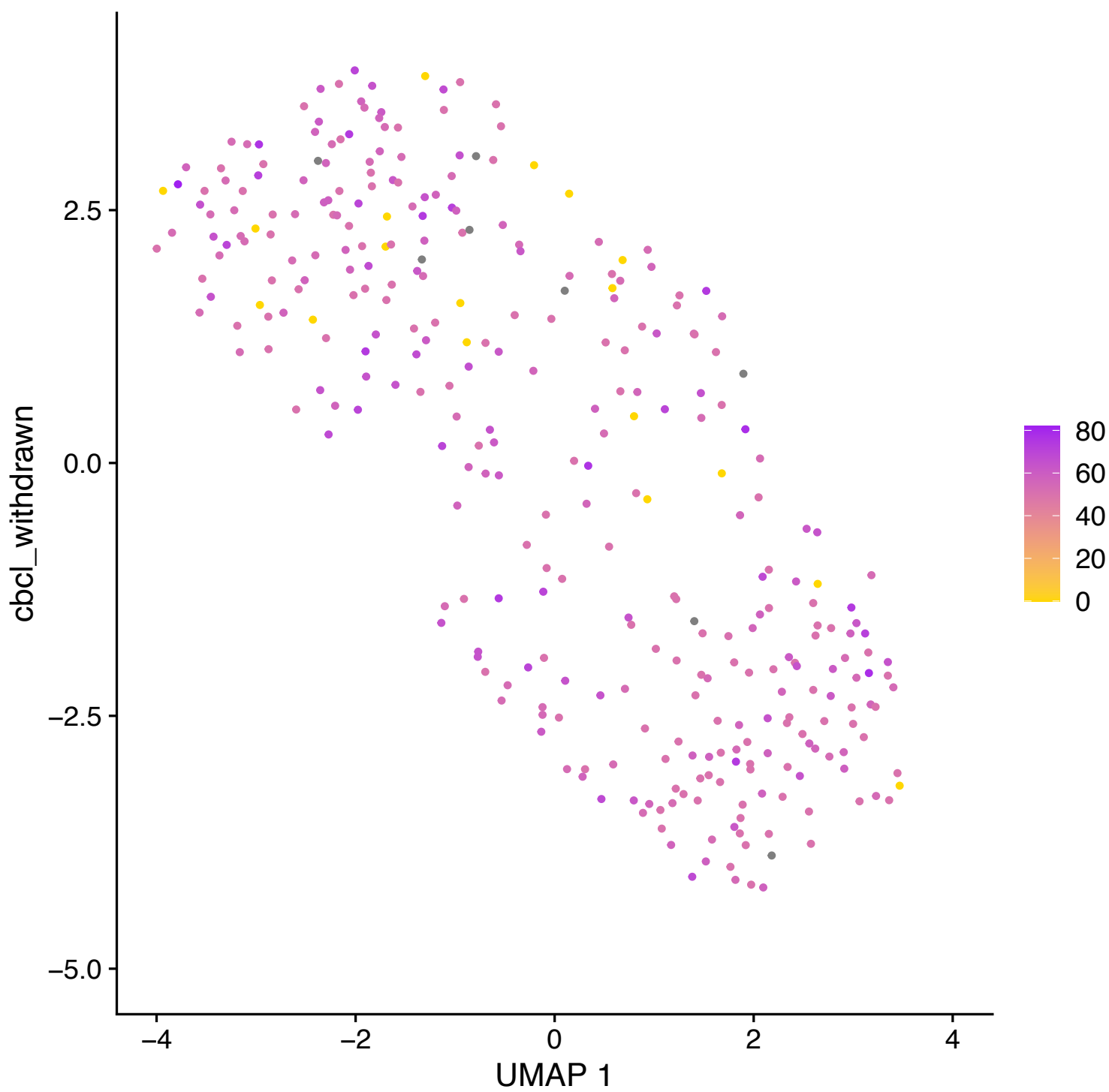

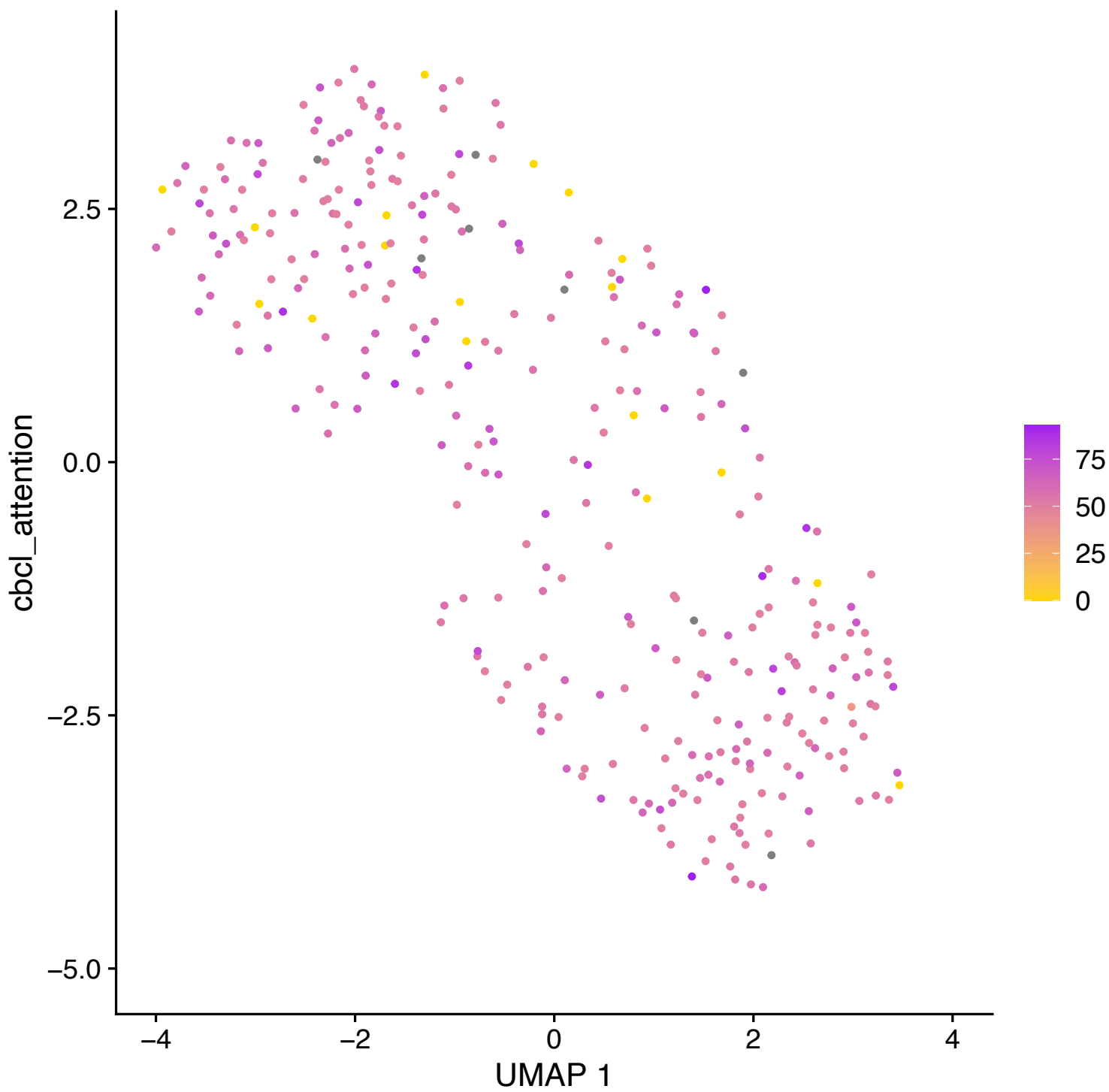

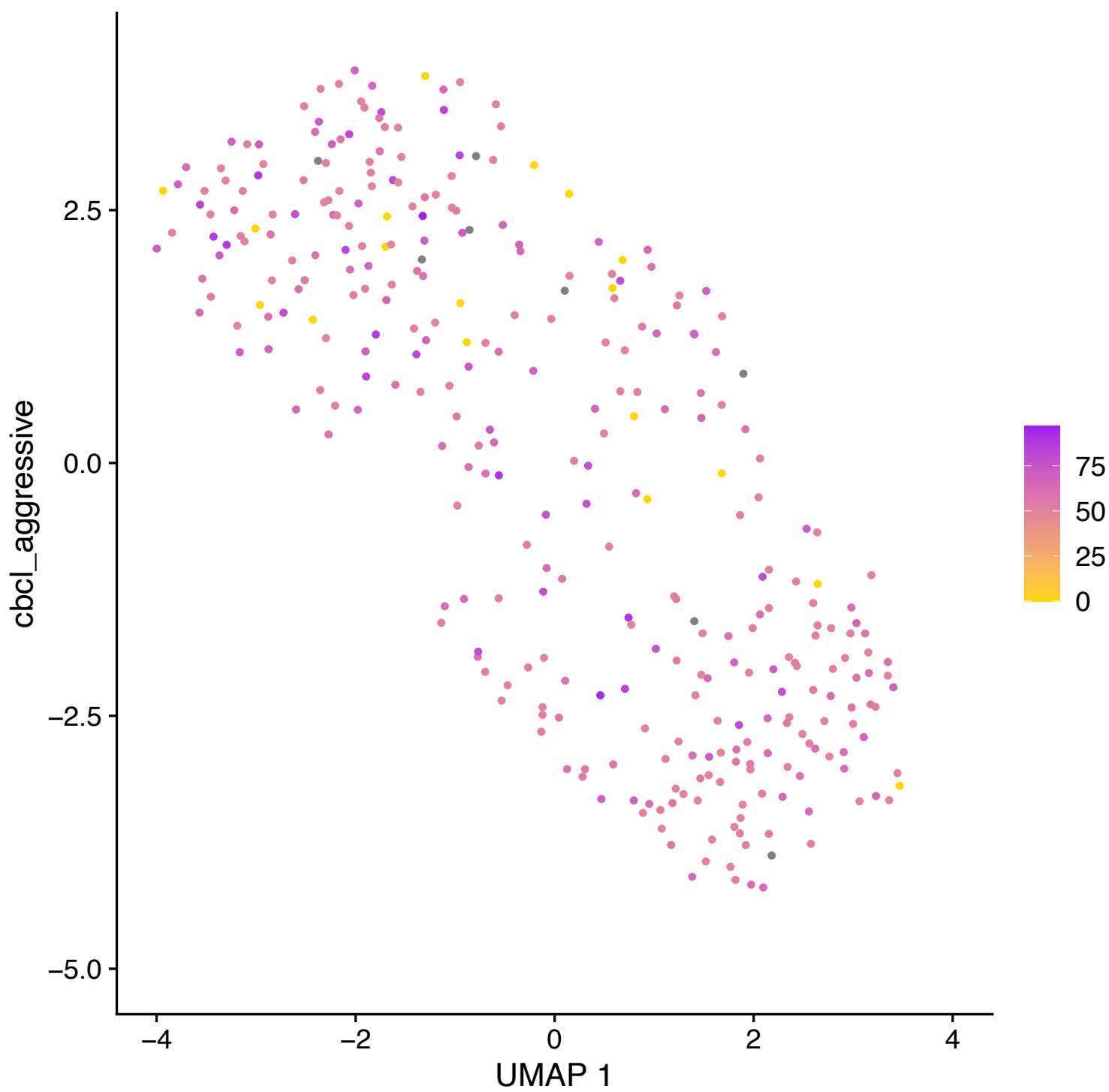

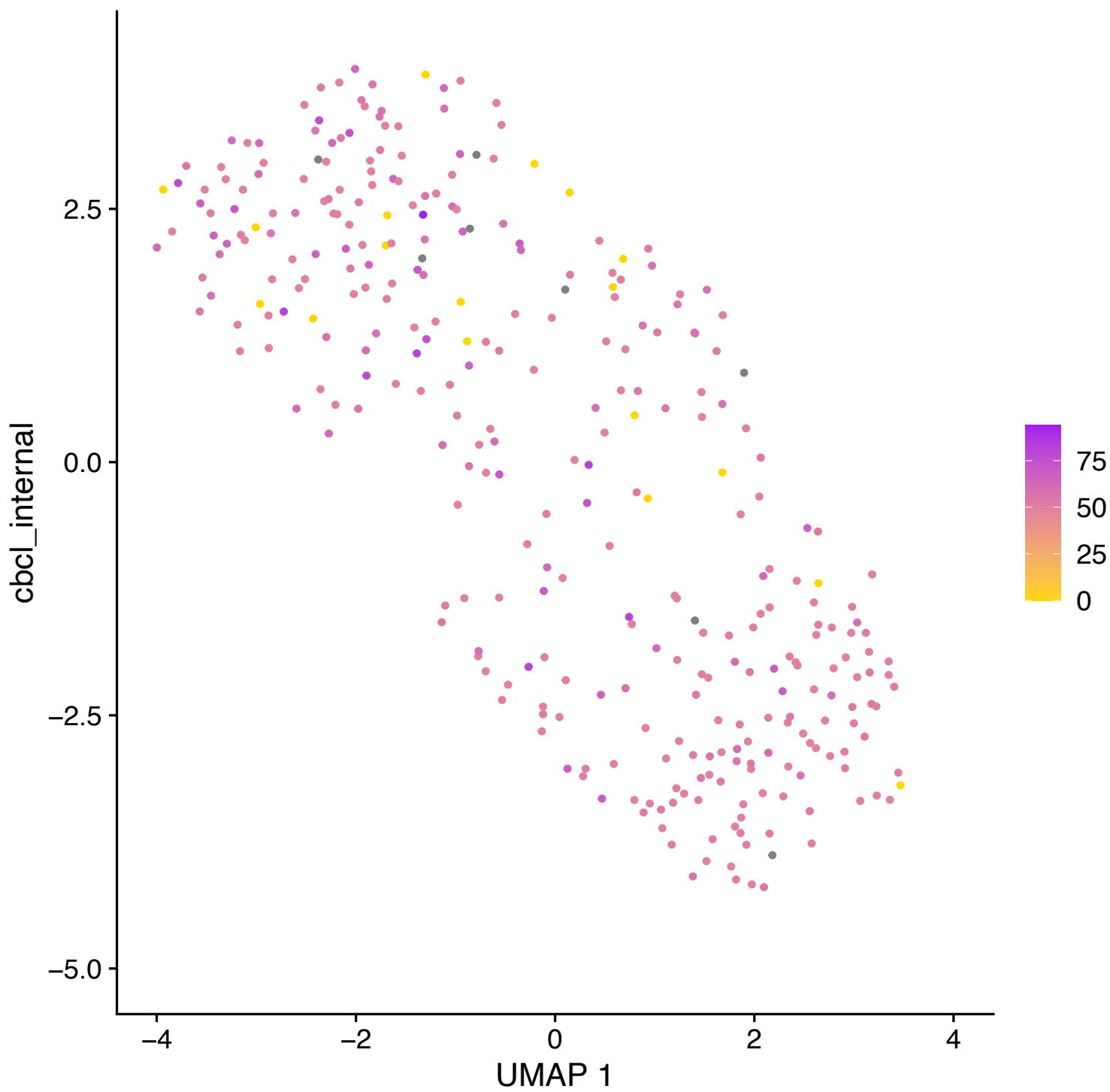

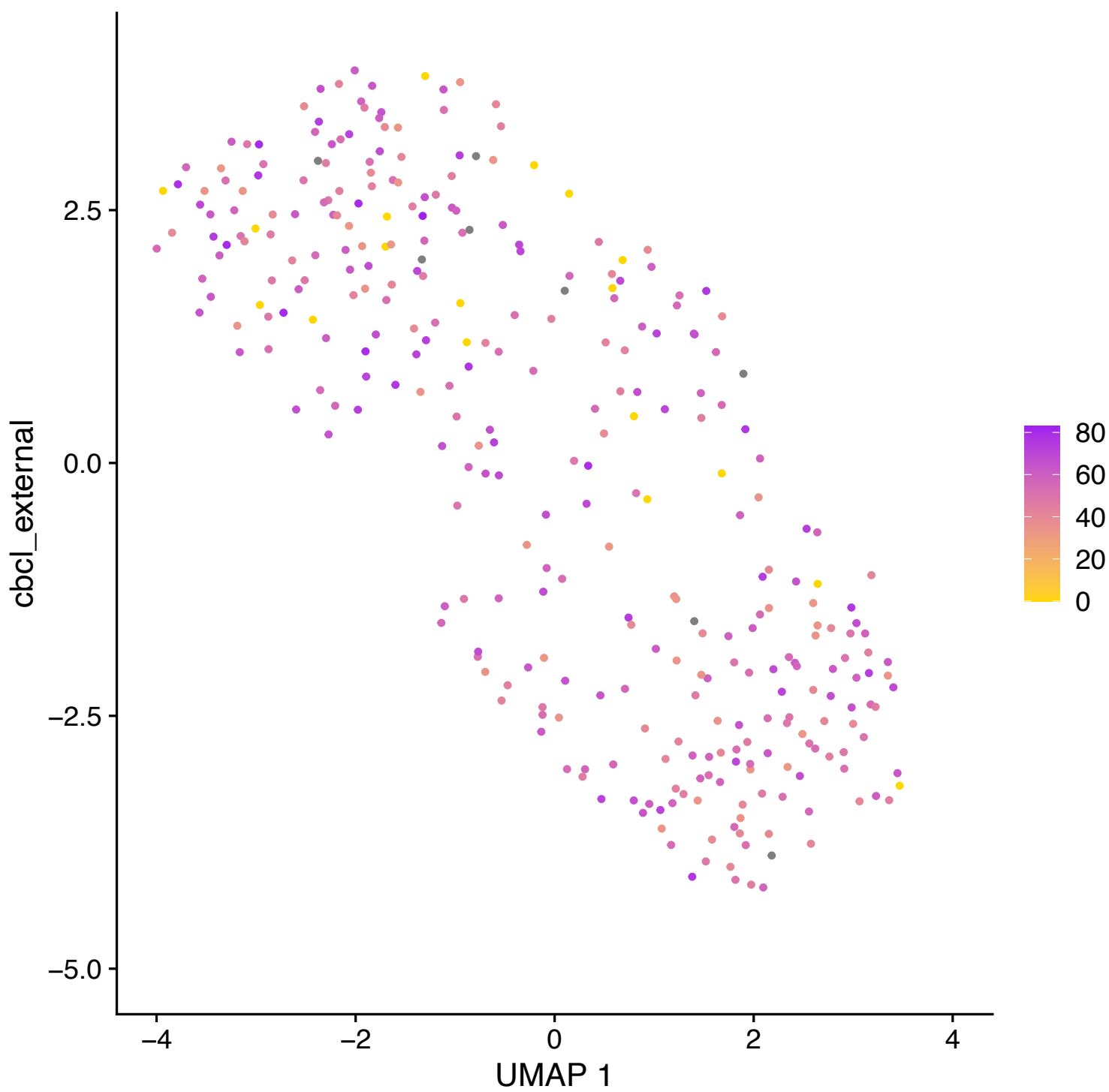

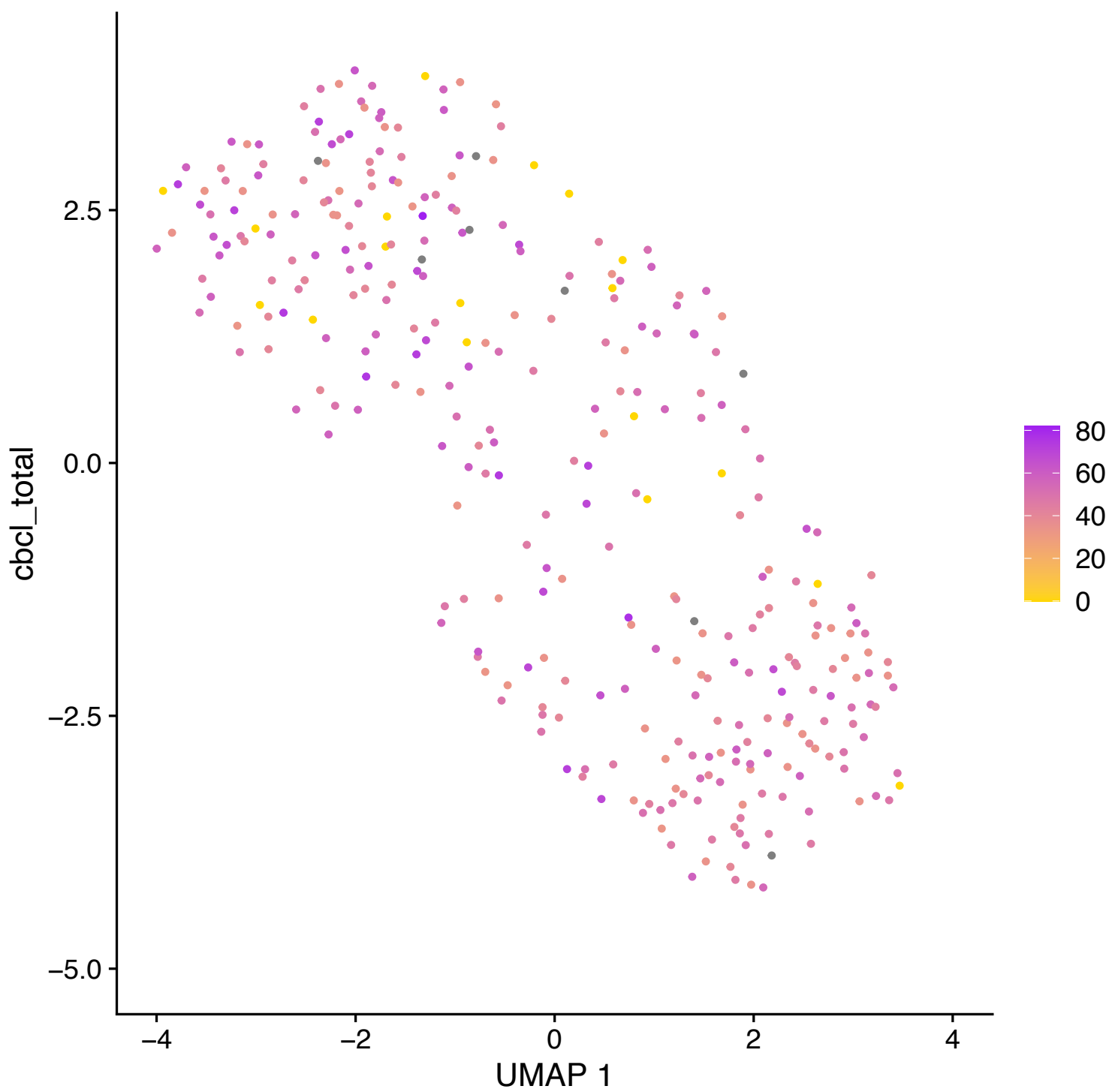

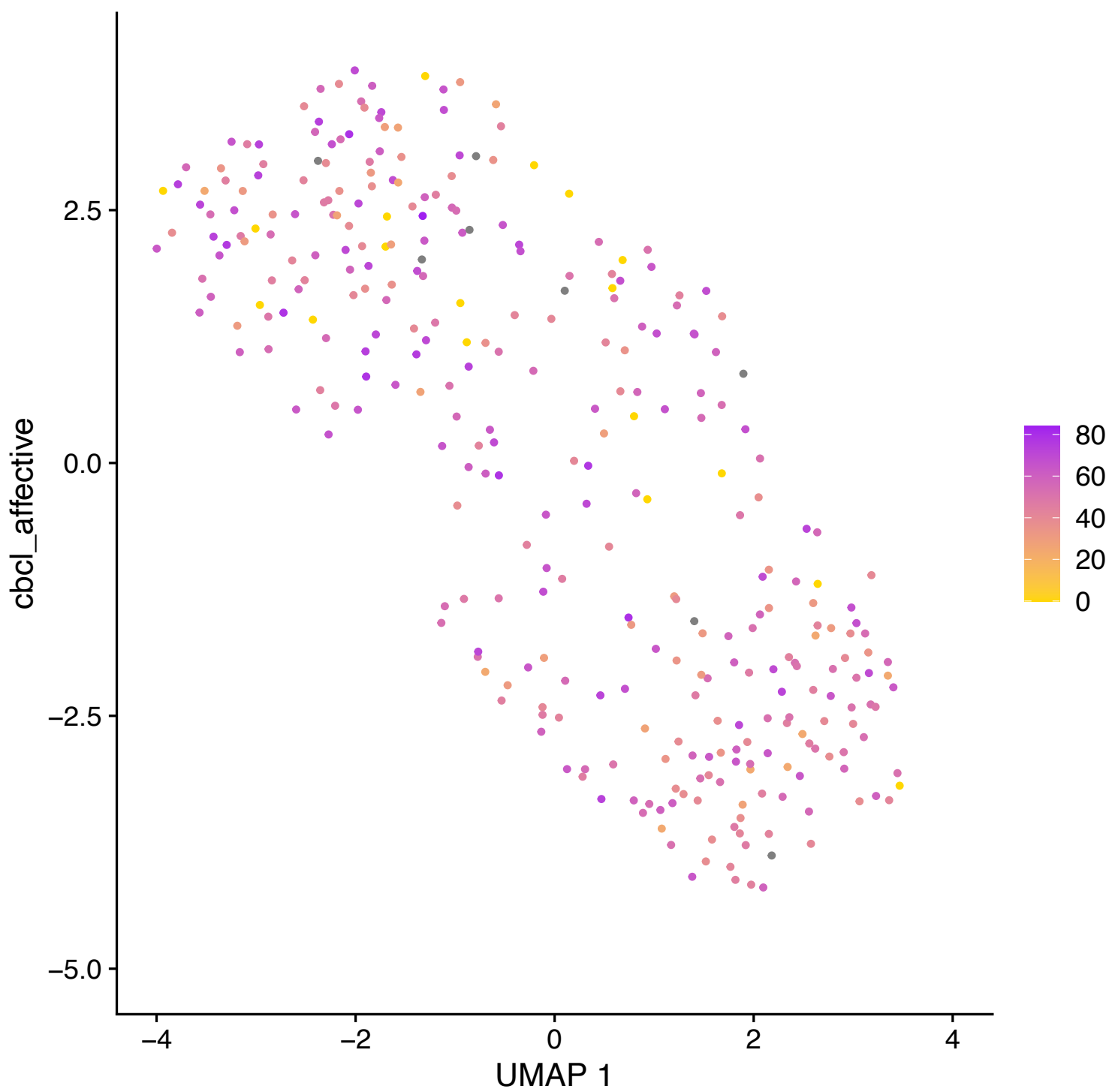

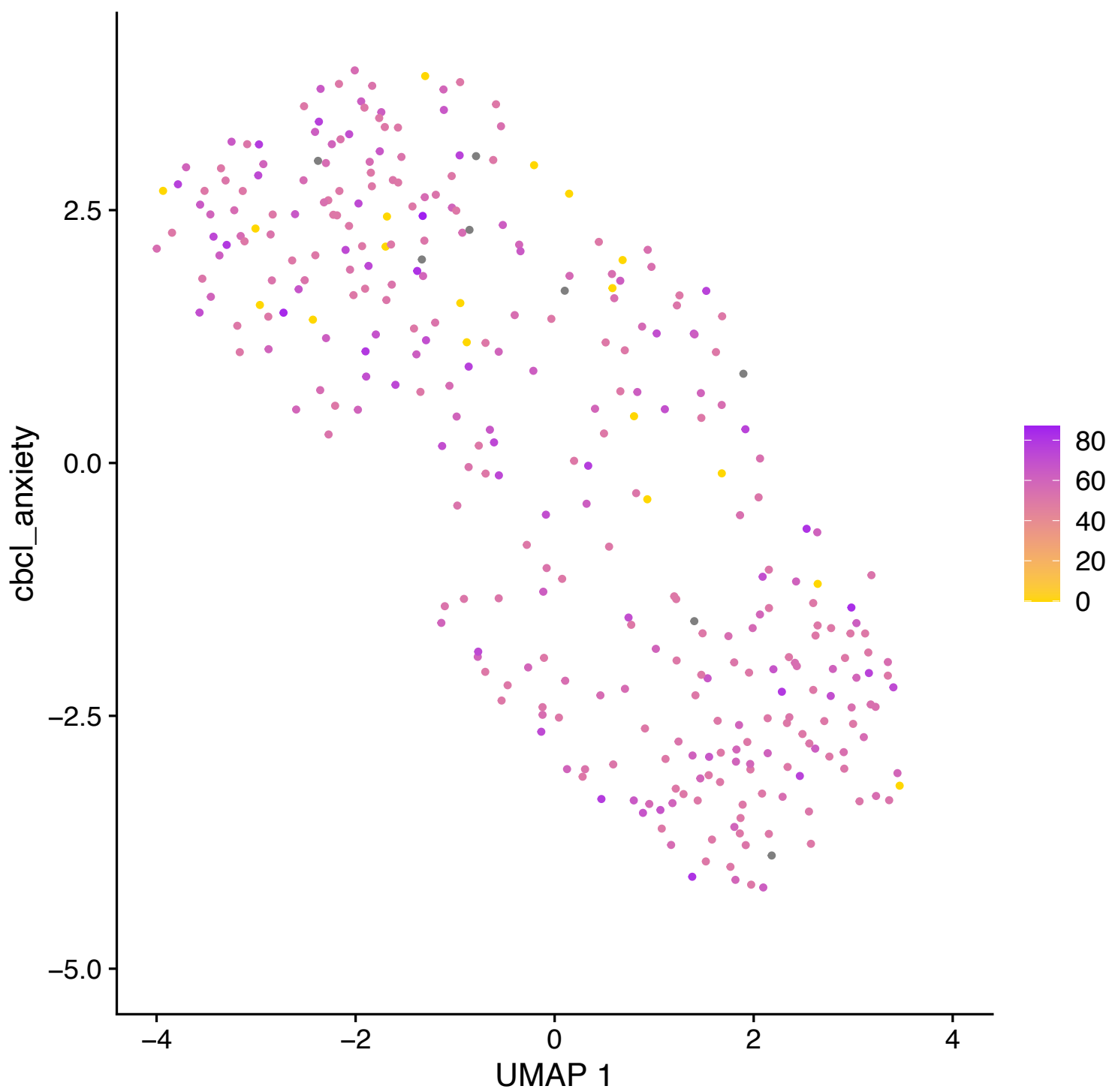

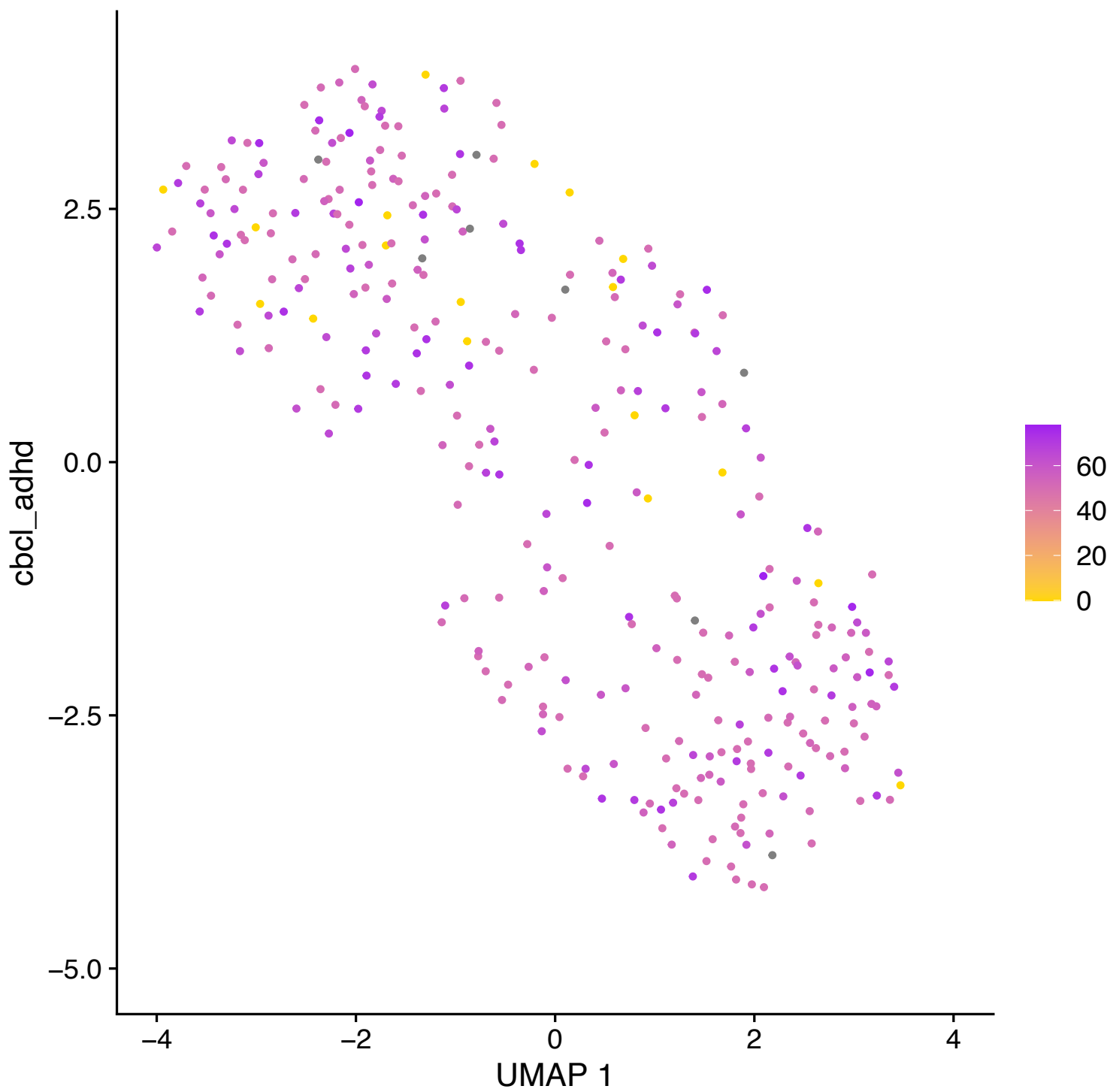

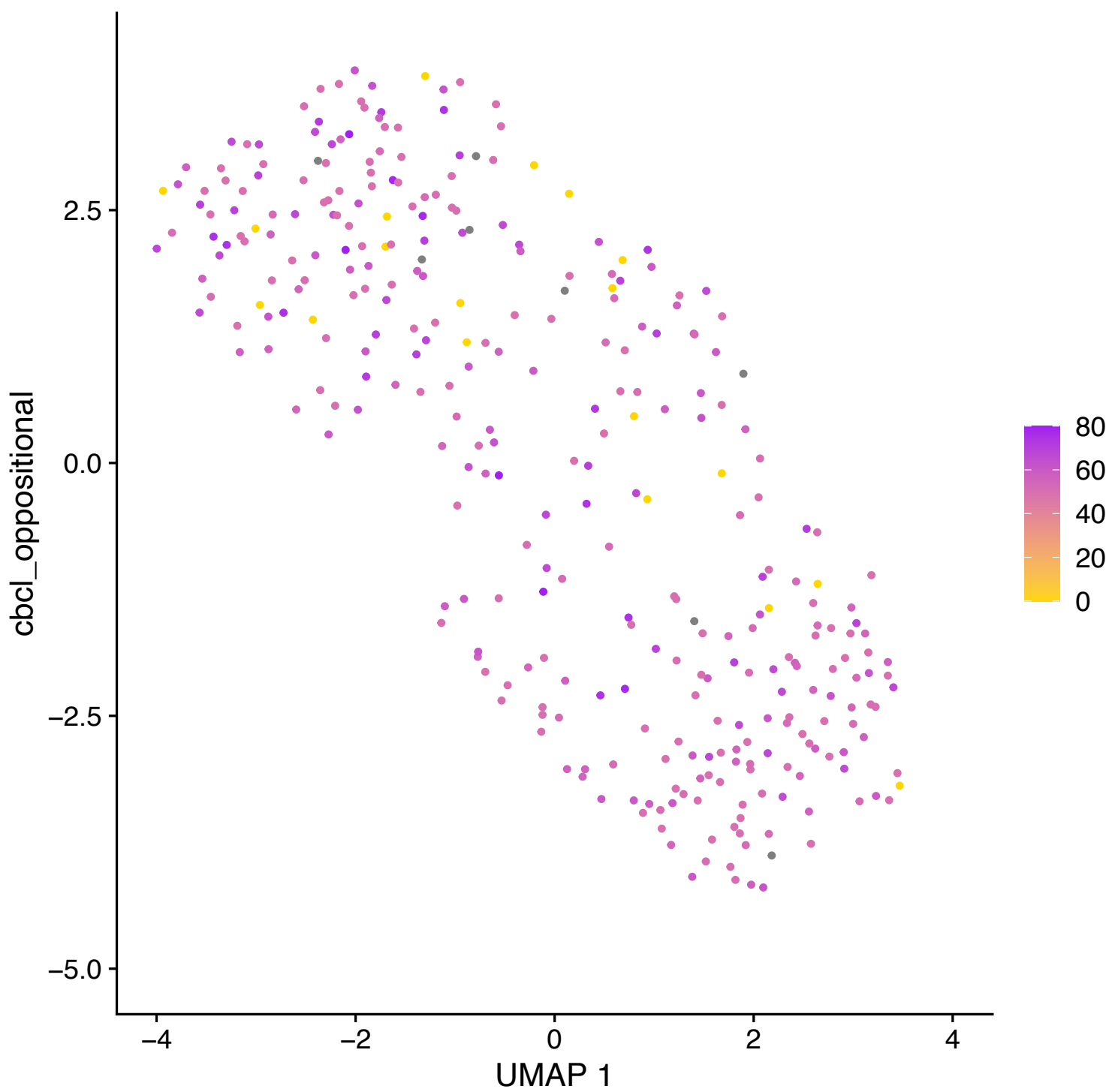

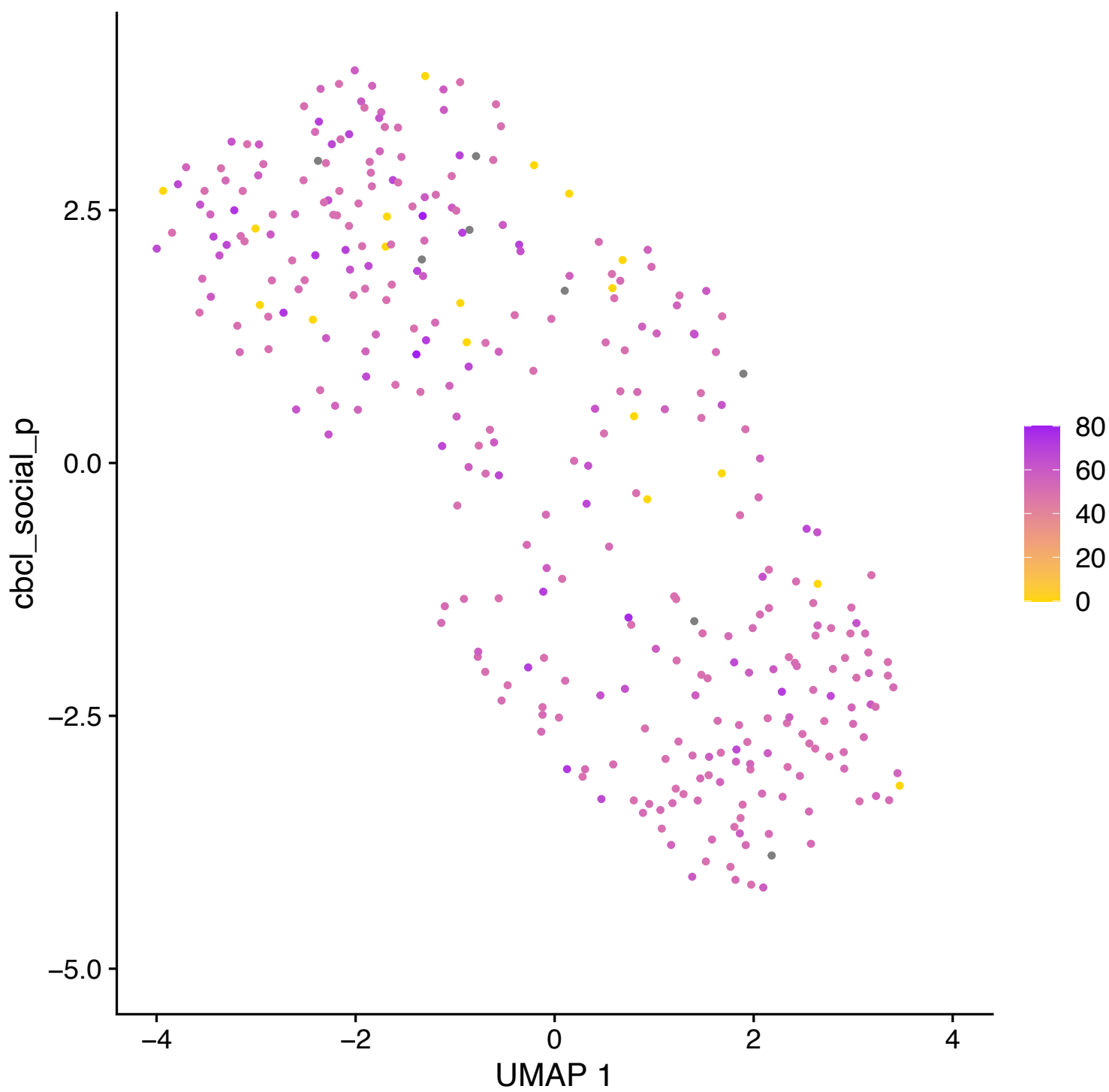

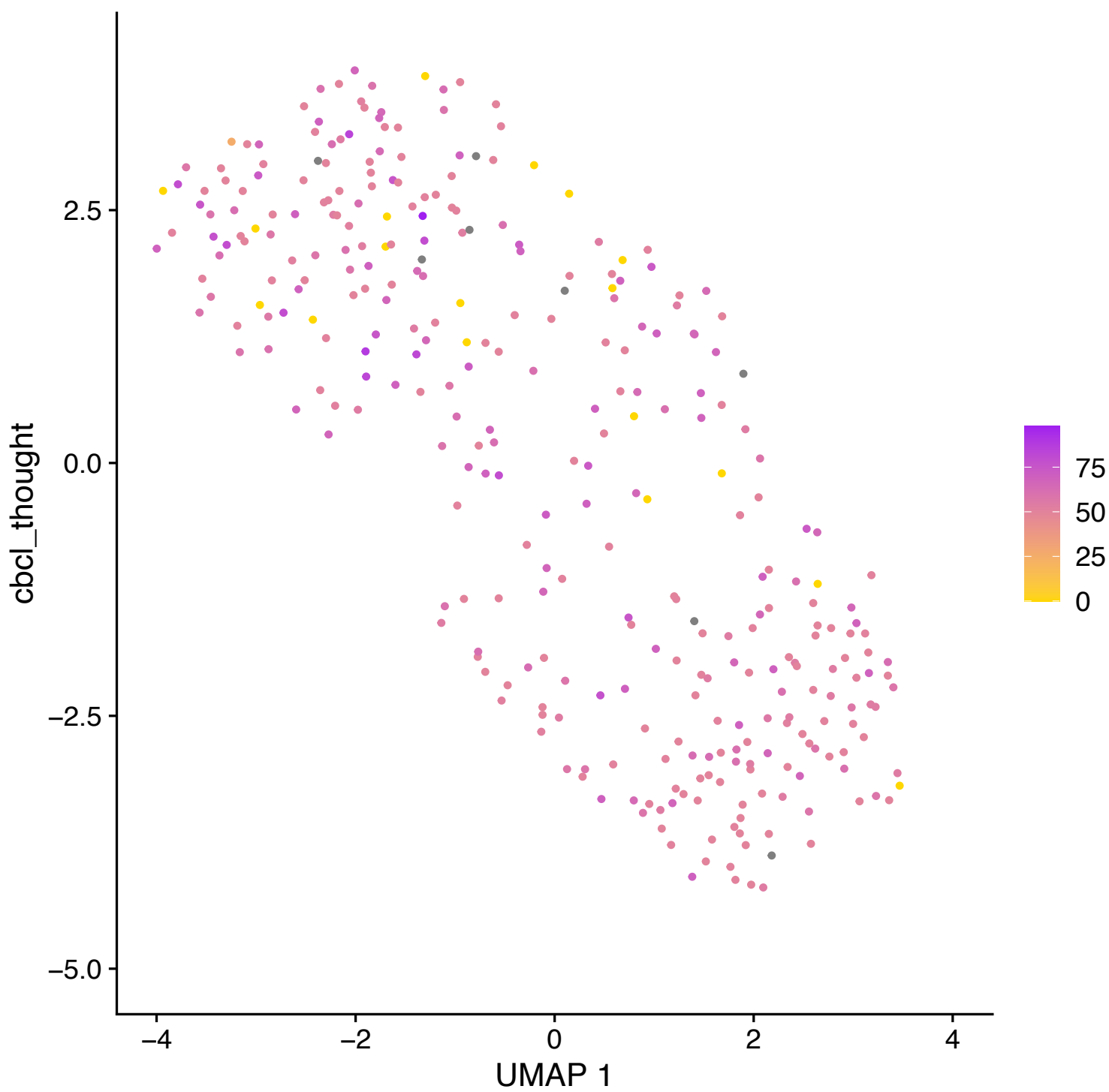

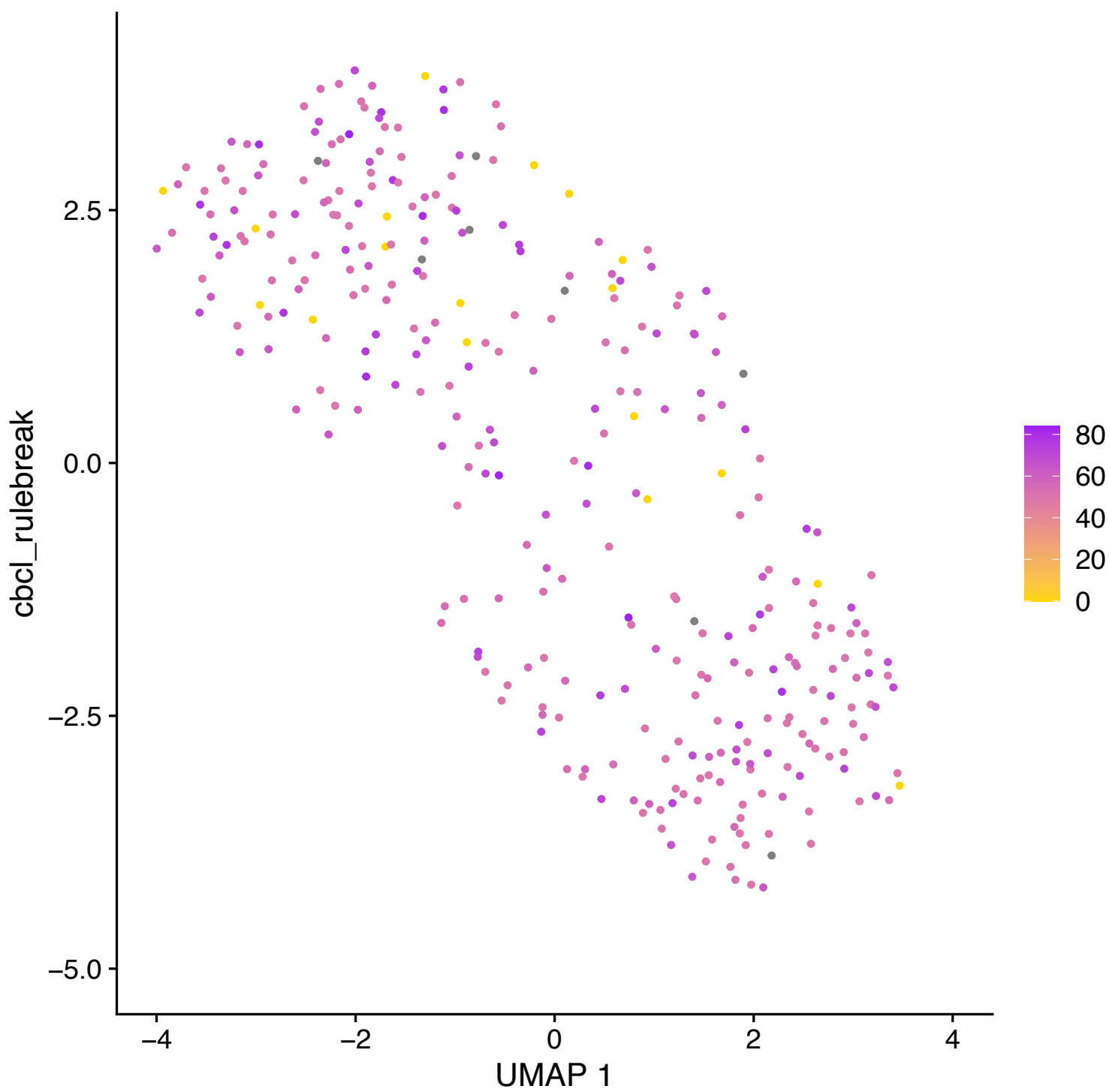

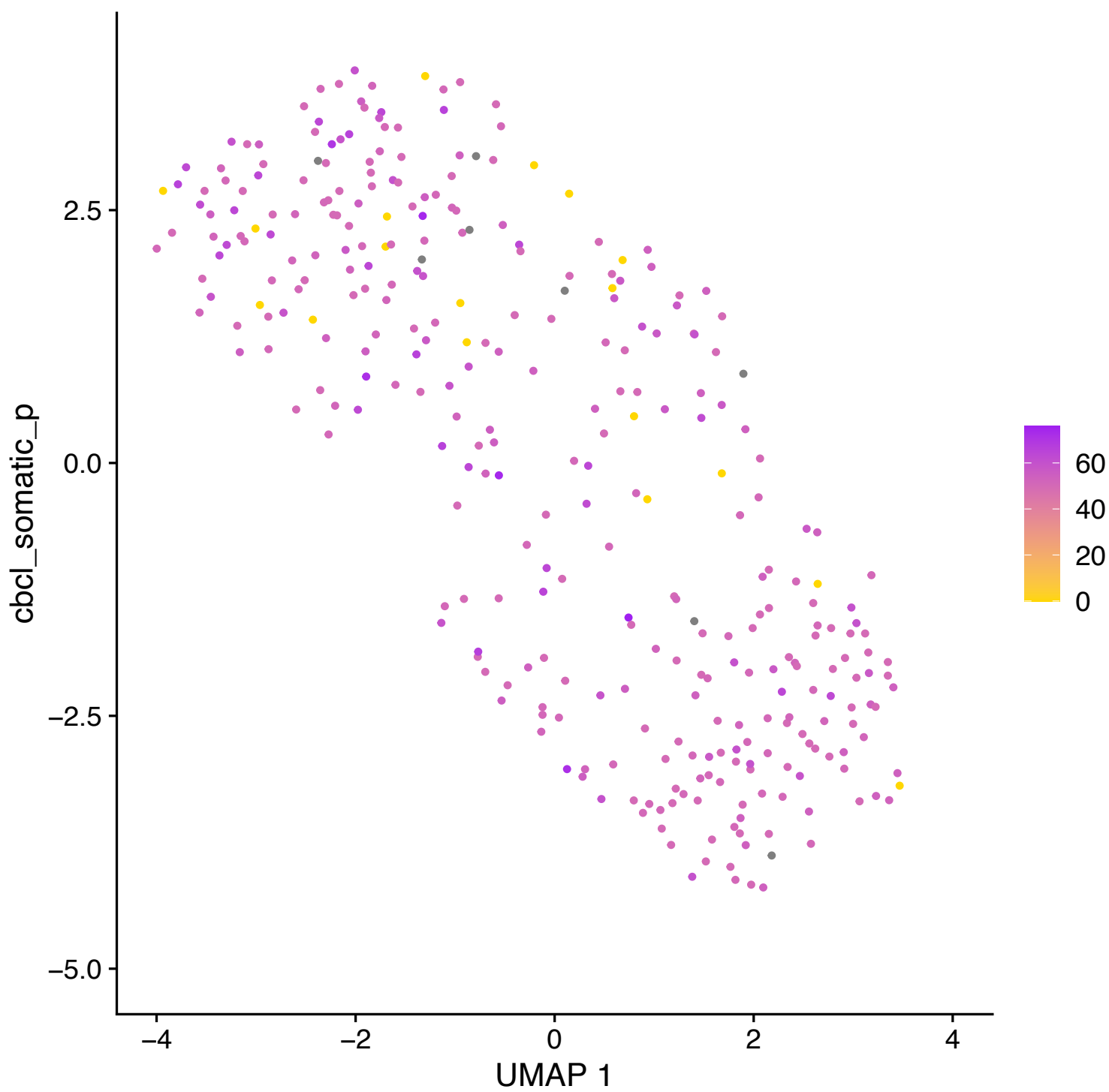

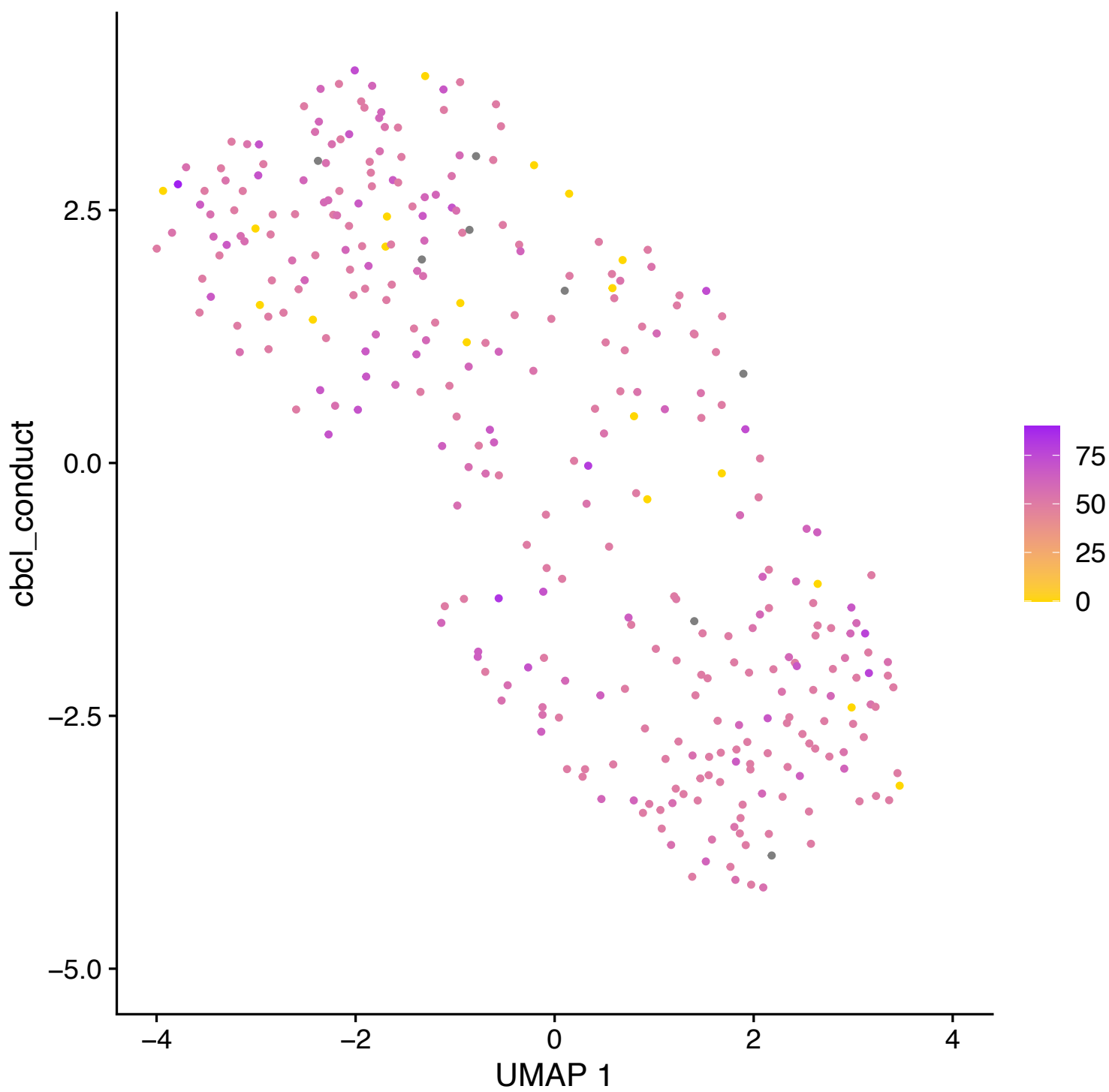

Supplement: Supplementary Material [file IMAG.a.144_supp.zip › Supplementary_Figure_S2.pdf]
